# A microporous polymer based on nonconjugated hindered biphenyls that emits blue light

**DOI:** 10.1038/s41598-024-65743-5

**Published:** 2024-06-28

**Authors:** Tamara L. Church, Lars Eriksson, Valentina Leandri, James M. Gardner, Niklas Hedin

**Affiliations:** 1 Department of Materials and Environmental Chemistry, Stockholm University, 106 91 Stockholm, Sweden; 2 Department of Chemistry, Applied Physical Chemistry, KTH Royal Institute of Technology, 10044 Stockholm, Sweden; 3 RISE Chemical Process and Pharmaceutical Development, Forskargatan 20J, 15136 Södertälje, Sweden

**Keywords:** Materials chemistry, Polymer chemistry

## Abstract

Microporous organic polymers that have three-dimensional connectivity stemming from monomers with tetrahedral or tetrahedron-like geometry can have high surface areas and strong fluorescence. There are however few examples of such polymers based on hindered biaryls, and their fluorescence has not been studied. Hypothesizing that the contortion in a hindered biphenyl moiety would modulate the optical properties of a polymer built from it, we synthesized a meta-enchained polyphenylene from a 2,2ʹ,6,6ʹ-tetramethylbiphenyl-based monomer, in which the two phenyl rings are nearly mutually perpendicular. The polymer was microporous with *S*_BET_ = 495 m^2^ g^−1^. The polymer absorbed near-UV light and emitted blue fluorescence despite the meta-enchainment that would have been expected to break the conjugation. A related copolymer, synthesized from 2,2ʹ,6,6ʹ-tetramethylbiphenyl-based and unsubstituted biphenyl-based monomers, was microporous but not fluorescent.

## Introduction

Microporous organic polymers are assembled via covalent bonds and without metal nodes, and their microporous (i.e. having pores with d < 20 Å^1^) nature has made them potentially useful in fields that include gas separation processes^2–11^, catalysis^2,4,6,7,9,11–17^ and molecular sensing^11,18,19^. General advantages of amorphous two- and three-dimensional microporous polymers with defined connectivity (e.g. conjugated microporous polymers^9,20–22^ and porous aromatic frameworks^6,23,24^) include thermal stability and tunable structures that can incorporate functionality.

**Figure 1:**
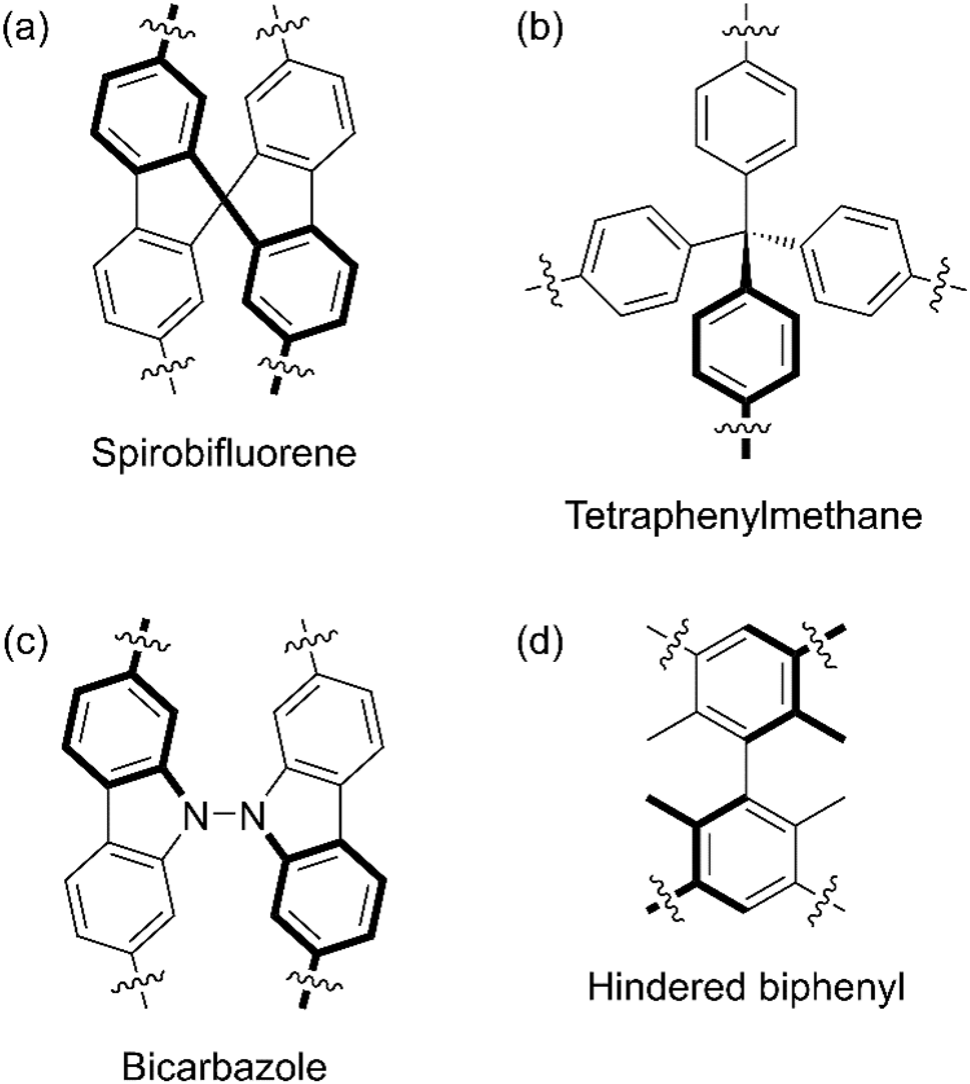
Tetrahedroid moieties that have been used as nodes in microporous polymers.

Many microporous polymers have been constructed from moieties with idealized S_4_ or D_2d_ geometry (Fig. 1). Here, we will refer to this group of structures as tetrahedroid because their enchainment points are arranged approximately in a tetrahedron or a ‘stretched’ tetrahedron in which two of the points have been drawn symmetrically away from the other two. Microporous polymers based on spirobifluorene (Fig. 1a) were described in 2008^25^; these were fluorescent and had high surface areas. Microporous polymers containing tetraphenylmethane (Fig. 1b) and even tetraphenylsilane nodes were reported by several groups in 2009^26–28^. These polymers can have extremely high surface areas (> 6400 m^2^ g^−1^)^29^. More recently, microporous polymers have been based upon the twisted but conjugated 9,9′-bifluorenylidene moiety^30^ as well as on the tetrahedroid 3,3′,6,6′-enchained *N*,*N′*-bicarbazole node^31^ (Fig. 1c), which is composed of near-orthogonal π systems (the single-crystalline monomer had a dihedral angle about the N–N bond of 87.4°). The dihedral angle between the phenyl groups in a 2,2′,6,6′-tetrasubstituted biphenyl moiety (Fig. 1d) depends on the substituent^32,33^. Thus a sufficiently hindered hypothetical biphenyl group can have a dihedral angle of 90° and be tetrahedroid. Microporous polymers have been prepared from the alkyne–alkyne coupling of 3,3′,5,5′-tetraethynyl-2,2′,6,6′-tetrasubstituted biphenyl monomers that have dihedral angles as large as 89.7° in the solid state^33^. These polymers were therefore composed of hindered biphenyl nodes connected by 1,4-enchained 1,3-butanedienyl struts, and had surface areas as high as 3420 m^2^ g^−1^.

Fluorescence has been demonstrated in microporous polymers based on tetrahedroid spirobifluorene^34–39^, *N*,*N′*-bicarbazole^31^, and tetraphenylmethane^34,40–43^ as well as tetraphenylstannane^44^ cores; however, it has not been investigated in microporous polymers with hindered biphenyl nodes. Para-enchained polyphenylenes generally have large band gaps^45,46^ and microporous polyphenylenes can therefore emit blue light^25,35,47–49^ and act as near-UV photocatalysts^50–52^. However, a microporous polymer composed solely of the tetrahedroid and hindered biphenyl in Fig. 1d would necessarily be meta-enchained, and would be expected to have similar local electronic properties to poly(*m*-phenylene), which has a broken conjugation that has been attributed to inherent electronic features of the linked *m*-phenylene units, and a HOMO–LUMO band gap that depends relatively weakly on the dihedral angle^53^. Further, unlike tetraphenylmethane- and spiro-based micropolymers, those based on tetrahedroid hindered biphenyls lack the opportunity for homoconjugation, which is particularly well known in spiro systems (as spiroconjugation^54,55^). Thus the optical properties of microporous polymer based on the tetrahedroid biphenyl moiety in Fig. 1d may be quite unlike those based on other tetrahedroid monomers. To investigate this, we constructed two crosslinked microporous polymers from a hindered biphenyl node: a dehydrogenated homopolymer, and a copolymer with non-hindered biphenyl connecting groups. We studied the surface area and fluorescence of these microporous polymers.

## Experimental section

### General

Unless otherwise stated, chemicals were used as received. Chemicals were purchased from Sigma–Aldrich, except 2-bromo-*m*-xylene purchased from abcr. Air-sensitive manipulations were performed under N_2_ using standard Schlenk techniques. Tetrahydrofuran was dried by refluxing with and then distilling from LiAlH_4_ under N_2_, and was stored under N_2_ prior to use. Anhydrous dimethylformamide was stored under N_2_ and over molecular sieves (4 Å).

### Synthesis of monomers and polymers

*(2,6-Dimethylphenyl)boronic acid* was synthesized from 2-bromo-*m*-xylene using a literature method for converting aryl bromides to arylboronic acids^56^, and purified by precipitation from Et_2_O and washing with toluene. ^1^H NMR (CDCl_3_) *δ* 7.17 (t, 1 H), 7.00 (d, 2 H), 4.59 (s, 2 H), 2.39 (s, 6 H).

*2,2′,6,6′-Tetramethylbiphenyl* (**1**) was prepared based on a literature procedure^57^. Molecular sieves (4 Å) were crushed in a mortar and dried overnight in a glassware oven. The sieves were combined with a Teflon-coated magnetic stir bar in an oven-dried two-necked round-bottom flask which was transferred to a Schlenk line and evacuated and flushed with N_2_ three times. *(2,6-Dimethylphenyl)boronic acid* (2.402 g, 16.0 mmol), KO^t^Bu (2.591 g, 23.1 mmol), and the PEPPSI-^i^Pr catalyst (0.2176 g, 0.320 mmol) were added to the flask under a flow of N_2_, and the flask was sealed with a rubber septum and evacuated and flushed with N_2_ three times before anhydrous *tert*-butanol (31.2 mL, 326 mmol) and 2-bromo-*m*-xylene (1.1 mL, 8.26 mmol) were added via syringe. Under continuous N_2_ flow, the rubber septum was replaced with a glass stopper, and the mixture was stirred at 65 ℃ for 66 h. After cooling to room temperature, the reaction mixture was filtered and the solids washed with ether. The filtrate was reduced on the rotovap and the product purified by column chromatography in hexane to give a white solid (0.7972 g, 46% yield) whose ^1^H NMR spectrum was consistent with that reported for **1**^58^.

*3,3′,5,5′-Tetraiodo-2,2′,6,6′-tetramethylbiphenyl* (**2-I**_**4**_) was prepared using a literature procedure^59^.

*3,3′,5,5′-Tetrabromo-2,2′,6,6′-tetramethylbiphenyl* (**2-Br**_**4**_) was prepared using a procedure modified from the literature method for brominating *m*-xylene^60,61^. Compound **1** (0.600 g, 2.85 mmol), I_2_ (0.0048 g, 0.019 mmol), and chloroform (9.0 mL) were combined in a 50 mL round-bottom flask. The mixture was cooled in an ice bath and allowed to stir for 10 min before Br_2_ (2.46 mL, 48.0 mmol) was added dropwise over 2 h. The mixture was allowed to warm to room temperature and stirred. After 22 h, the mixture was poured into a beaker with 150 mL of KOH(*aq*, 20 wt%). The mixture was again cooled in an ice bath, and then stirred for 3 h before being extracted with CH_2_Cl_2_, washed three times each with deionized H_2_O and brine, and dried over MgSO_4_. The solution was filtered and reduced on the rotovap to give a solid that was dissolved in hot EtOH and allowed to cool to room temperature. Crystalline **2-Br**_**4**_ (0.98 g, 65% yield) was isolated by filtration. ^1^H NMR (CDCl_3_) *δ* 7.82 (s, 2 H), 1.97 (s, 12 H).

**PTd-0** was synthesized using a modified Yamamoto procedure that was based on the methods of Ueda et al.^62^ and Colon and Kelsey^63^. Zinc powder was stirred overnight in glacial acetic acid, then filtered, washed with copious Et_2_O, and dried under vacuum. Separately, an oven-dried two-necked round-bottom flask containing an oven-dried stir bar was evacuated, then refilled with N_2_. NiCl_2_ (15.9 mg, 0.123 mmol) and bipyridine (20.7 mg, 0.133 mmol) were added under flowing N_2_, and the flask was again evacuated and refilled with N_2_. PPh_3_ (399.8 mg, 1.52 mmol) was added under flowing N_2_, and the flask was evacuated and refilled again before NaBr (1.894 g, 18.4 mmol) and finally the purified Zn (500.2 mg, 7.64 mmol) were added. The flask was then evacuated and refilled with N_2_ × 3 before dry DMF (2.5 mL) was added under flowing N_2_. The flask was lowered into an oil bath, which had been preheated to 85 ℃, and heated for 4 h, during which time the mixture became red. The flask was removed from heat while **2-Br**_**4**_ (0.5746 g, 1.09 mmol) was added under flowing N_2_, and the mixture sealed under N_2_ and returned to heat at 85 ℃. After 4 d, the mixture was red–brown. The temperature was raised to 125 ℃. After an additional 8 days, the lighter brown suspension was removed from heat. The suspension, which contained a very fine powder, was poured into a cellulose thimble to filter. The filtrate was discarded, and the solid was extracted with methanol in a Soxhlet apparatus for 5 d. The resulting beige solid was allowed to air dry, then transferred to a round-bottomed flask containing HCl(*aq*, 18 wt%) and heated to reflux overnight. The cooled mixture was filtered and the resulting yellow–beige solid was washed × 2 with deionized H_2_O and transferred to a new cellulose thimble. This was placed in a Soxhlet apparatus and extracted with deionized water for 2 d and chloroform for 3 d. The resulting pale beige powder was allowed to air dry, then transferred to a vial (m_solid_ = 76.8 mg, yield = 34% based on C_16_H_14_). ^1^H NMR (solid) *δ* 7.2, 1.7 ppm. ^13^C NMR (solid) *δ* 141, 134, 128 (shoulder), 123, 17.9 ppm.

**PTd-2** was synthesized with guidance from a published procedure for the synthesis of a porous polymer via Suzuki coupling^47^. An oven-dried flask with a magnetic stir bar was evacuated and filled with N_2_ three times before **2-I**_**4**_ (0.1908 g, 0.267 mmol) and 4,4′-biphenyldiboronic acid (0.1293 g, 0.535 mmol) were added under a flow of N_2_. The flask was again evacuated and flushed twice with N_2_ before anhydrous DMF (8.2 mL) was added via a syringe. The mixture was subjected to three freeze–pump–thaw cycles and the flask refilled with N_2_ and allowed to come to room temperature. Pd(PPh_3_)_4_ (0.0535 g, 0.0463 mmol) was added, followed by 1.0 mL of 2.00 M K_2_CO_3_(*aq*). The mixture was subjected to three more freeze–pump–thaw cycles and the flask was refilled with and sealed under N_2_. The mixture was stirred at 150 ℃ for 24 h, then allowed to cool to room temperature and poured into deionized water. The resulting suspension was filtered to give a mixture of white, yellow, beige, and darker solids, which were washed with 200 mL each of deionized H_2_O, CH_2_Cl_2_, and methanol and finally dried under vacuum at 100 ℃ overnight. The solid was then crushed and transferred to a cellulose thimble for Soxhlet extraction. The solid was extracted for 24 h in each of H_2_O, CH_2_Cl_2_, MeOH, and tetrahydrofuran, then dried under vacuum overnight to give 0.1088 g of a light beige solid. The yield calculated for a polymer of the repeat unit of C_40_H_30_ is 80%. ^1^H NMR (solid) *δ* 6.8, 1.3 ppm. ^13^C NMR (solid) *δ* 141, 131 (shoulder), 128, 106, 17.5 ppm.

### Characterization

#### Single crystal X-ray diffraction

A needle of **2-**I_4_ was affixed to a glass fibre using epoxy glue and mounted on a goniometer head for measurement in a Bruker D8 Venture diffractometer. Data was collected at room temperature using Mo Kα X-rays (λ = 0.71073 nm).

#### Scanning electron microscopy (SEM)

Powdered samples were affixed to Al stubs using carbon tape and observed as-is on a JEOL JSM-7000F electron microscope using an electron energy of 15 kV.

#### Gas sorption

Gas sorption isotherms were measured on a Micromeritics ASAP 2020 instrument. Samples were degassed by heating under vacuum for 12 h before measurement. The degas temperatures were chosen following thermogravimetric analysis to avoid thermal decomposition; thus **PTd-0** and **PTd-2** were degassed at 150 and 200 ℃, respectively. N_2_ isotherms were measured at −196 °C and *S*_BET_ was calculated over P/P_0_ = 0.009–0.10 for **PTd-0** and P/P_0_ = 0.007–0.10 for **PTd-2**; these pressure ranges gave maximum correlation coefficients for plots of 1/[n((P_0_/P)–1)] vs. P/P_0_, and the slope and intercept of the plots were both positive. Pore size distributions were derived from the adsorption branch of the isotherm using the non-linear and classical density functional theory (NL-DFT) functional developed for the adsorption of N_2_ on carbon with slit-like pores as implemented in the Micromeritics Microactive software. CO_2_ adsorption was measured at 0, 20, and 30 ℃.

#### Infrared spectroscopy

Infrared (IR) spectra were measured on a Varian 610 IR spectrometer equipped with an IR microscope. Powdered samples were placed on a Specac single reflection attenuated total reflection (ATR) accessory and measured using a deuterated triglycine sulfate (DTGS) detector. The clean diamond element served as the background.

#### ^13^C nuclear magnetic resonance (NMR) experiments

^13^C Cross-Polarization (CP) Magic Angle Spinning (MAS) NMR spectra were recorded in a 4 mm probe head under conditions of ramped (70–100%) CP (for 3–4 ms) and MAS at frequency of 14 kHz. A Bruker Avance III 400 MHz spectrometer attached to a wide-bore magnet was used for the experimentation and 3072–14,336 k transients were recorded under ^1^H decoupling using a SPINAL sequence. A recycling time of 3.5 s was used, and a minor amount of exponential appodization was used during processing. The ^13^C NMR chemical shift scale was externally calibrated to the methine signal of adamantane.

#### Steady-state photoluminescence

Emission and excitation spectra were recorded with a Cary Eclipse fluorescence spectrophotometer. 2–3 mg of **PTd-0** and **PTd-2** were dispersed into 3.0 mL of chloroform (≥ 99.8% for HPLC) for solution measurements. For measurements in the solid-state, powders of **PTd-0** and **PTd-2** were pressed in between two glass microscope slides.

#### Time-resolved photoluminescnce

Time-resolved photoluminescence measurements were acquired with excitation at 405 nm (Thorlabs NPL41B, average of 10,000 shots) and detection at 520 nm on a home-built laser system as detailed in our previous works^64,65^.

#### Thermogravimetric analysis (TGA)

Measurements were performed on a TA Instruments Discovery TG under 25 mL/min N_2_. Samples were heated at 10 ℃/min to 100 ℃, held at that temperature for 30 min, and finally heated at 5 ℃/min to 1000 ℃.

## Results and discussion

**Scheme 1:**
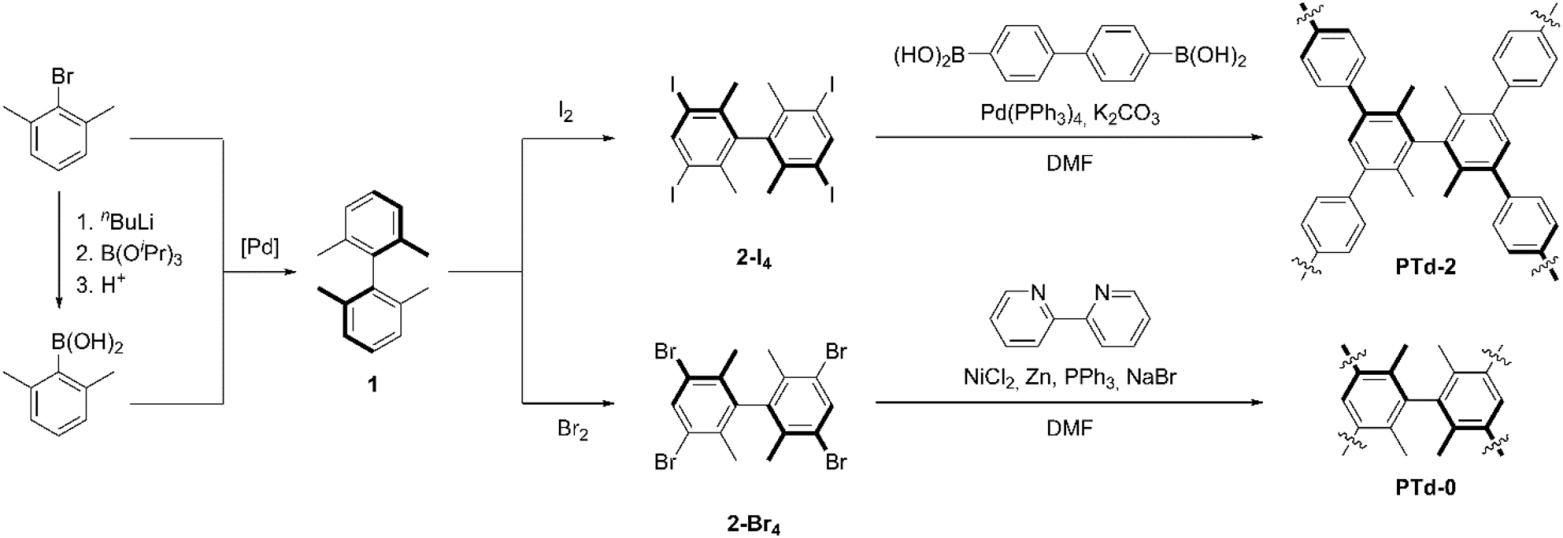
Summary of the syntheses of tetrahedroid biphenyl monomers **2**-**X**_4_ and the polymers **PTd-0** and **PTd-2**.

Microporous polymers were synthesized using monomers derived from the tetrahedroid compound **1**, composed of two π systems connected by a σ-bond, and sterically constrained by four ortho-methyl groups (Scheme 1). Compound** 1** was synthesized using Suzuki coupling chemistry^66^ and halogenated to give the two polymerizable monomers **2-X**_**4**_ (X = Br or I) as colourless solids. The IR spectra of both monomers **2-X**_**4**_ (Fig. S1) displayed sharp bands at low wavenumber (ν = 499 and 507 cm^−1^ for **X** = I and Br) that were attributed to ν(C–X). Analysis of the X-ray diffraction pattern from a single crystal of **2-I**_**4**_ revealed a monoclinic crystal (P2_1_/c; see Supporting Information for details) with a dihedral angle of 79.2° between the phenyl rings. Thus **2-I**_**4**_ did not have S_4_ symmetry in the crystalline phase, but the phenyl rings were nevertheless twisted far from co-planarity (Fig. S2), as expected from the steric hindrance exerted by the methyl groups. Similar results have been obtained for the closely related 3,3′,5,5′-tetraiodo-2,2′,4,4′,6,6′-hexamethylbiphenyl; the crystal structure of that compound contains two non-symmetry-equivalent molecules with dihedral angles of 78.3 and 88.8° between the phenyl groups^33^.

**Figure 2:**
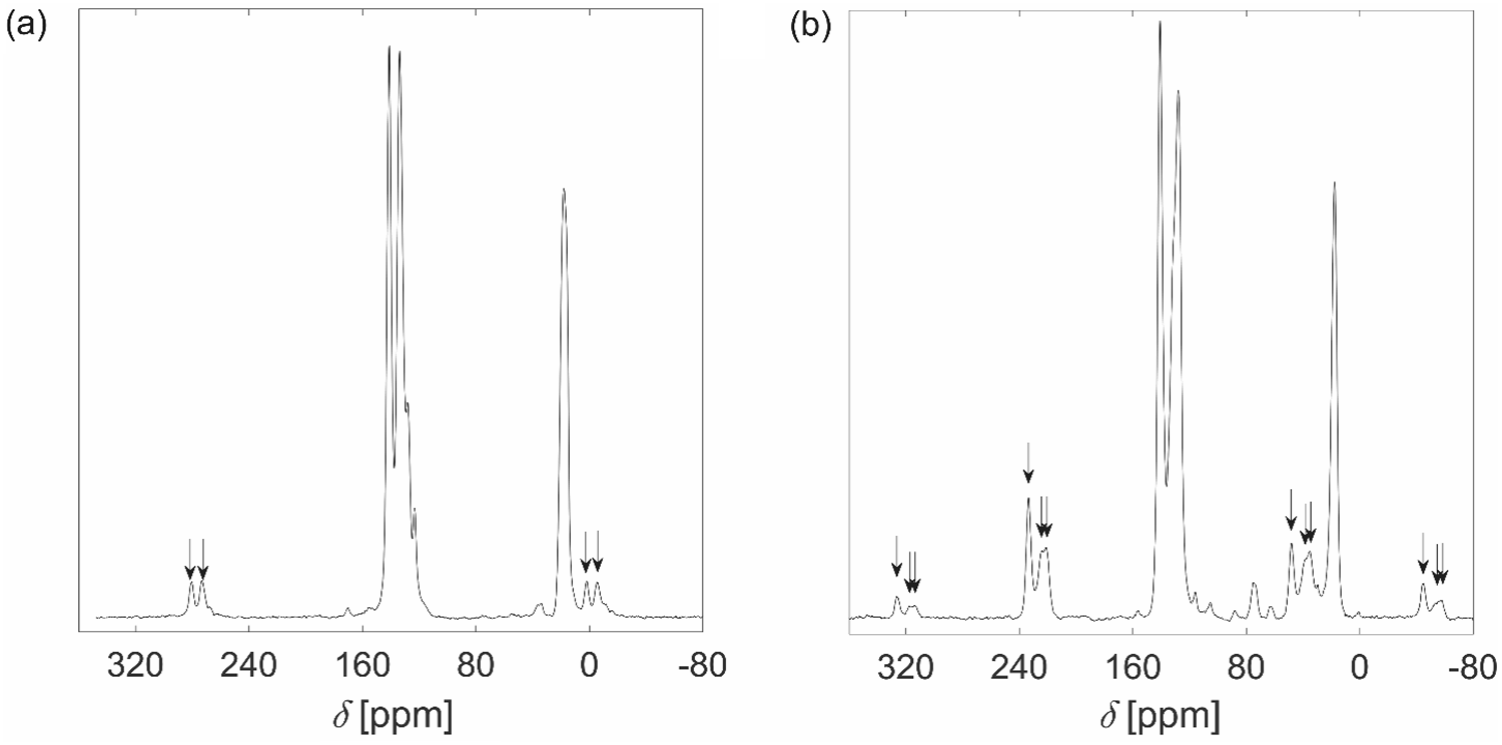
^13^C Cross-Polarization (CP) Magic Angle Spinning (MAS) NMR spectra of (**a**) **PTd-0** and (**b**) **PTd-2**, recorded under conditions of MAS and CP. Arrows indicate the positions of spinning side bands.

The tetrahedroid monomers **2-X**_**4**_ were used to form two microporous polymers (Scheme 1). The first, a dehydrogenated homopolymer of **1**, was a specifically methylated and highly crosslinked poly(*m*-phenylene), while the other was an alternating co-polymer of **1** with 4,4ʹ-enchained biphenyl units (Scheme 1). The polymers were labelled **PTd-A**, where** A** = 0 or 2 and gave the number of phenyl groups inserted as spacers between the** 1** cores. The beige polymer **PTd-0** was produced via a modified Yamamoto polymerization^67^ that used a Ni^2+^ salt as a catalyst precursor and Zn metal as a sacrificial reducing agent^63^. The monomer in this case was **2-Br**_**4**_, as this catalytic system couples aryl bromides faster than aryl iodides^63^. This method has been used to prepare poly(4-substituted-*m*-polyphenylenes)^62^; however, we used harsher conditions because of the sterically hindered aryl bromide sites in **2-Br**_**4**_. Following polymerization, the sharp band observed at 507 cm^−1^ in the IR spectrum of **2-Br**_**4**_ was replaced by a broader feature in the IR spectrum of **PTd-0**, indicating loss of C–Br bonds (Fig. S1a). The ^13^C NMR spectroscopic peak at 123 ppm (Fig. 2a) is consistent with an aromatic C atom bound to Br, and is assigned to unreacted C–Br groups. The ^13^C CPMAS NMR spectrum of **PTd-0** also contained three other peaks representing aromatic C nuclei: two large overlapping peaks at 141 and 134 ppm, and a shoulder at 128 ppm. Numerical regression analysis was used to model the aromatic region of the ^13^C CPMAS NMR spectrum of **PTd-0** with four Gaussian functions, and the peak at 123 ppm was estimated to represent ~7% of the aromatic intensity. **PTd-0** is assembled from a monomer with 12 aromatic C atoms. If each monomer retained one unreacted Br atom, we would expect ~8% of the aromatic intensity to be C atoms bound to Br. Therefore, rather than an approximately infinite tetrahedroid network, **PTd-0** had, on average, slightly less than one Br atom for every second aromatic ring, meaning that each biphenyl monomer was connected to an average of three other monomers. An incomplete polymerization is understandable in this system given that the steric hindrance around the C–Br moieties, and the hindered rotation around the C–C bonds connecting phenyl groups within and between monomers may have prevented conformational rearrangements that would have allowed coupling reactions within the growing polymer.

Br atoms were also detected in energy-dispersive X-ray (EDX) measurements of **PTd-0** (Fig. S3), consistent with some incomplete polymerization. Small (<0.5 at%) amounts of P were also detected by EDX, and likely originated from the phosphine ligand used in the catalytic coupling that formed the polymer. As **PTd-0** was purified via extended Soxhlet extraction, the detected P atoms are likely present in molecules confined within the pores of **PTd-0**. Upon heating in N_2_, **PTd-0** retained 93% of its mass up to 405 ℃, and decomposed rapidly above that temperature (Fig. S4). The pyrolysis residue could be completely combusted in air (Fig. S4).

The co-polymer of **2-I**_**4**_ and 4,4′-biphenyldiboronic acid, labeled **PTd-2** (Scheme 1) was synthesized for comparison to **PTd-0**. A closely related microporous polymer has been reported; it differed from **PTd-2** in that it bore methyl substituents *para* to the hindered biphenyl linkage^68^. **PTd-2** contained unsubstituted phenyl rings as well as the substituted, hindered rings that make up **PTd-0**. Thus **PTd-2** contained meta-enchained phenyls as in **PTd-0**, but also domains of four para-enchained phenyls. The Suzuki coupling^66^ of **2-I**_**4**_ and 4,4′-biphenyldiboronic acid gave **PTd-2** as a light beige powder. The sharp ν(C–I) band observed at 499 cm^−1^ in the infrared spectrum of **2-I**_**4**_ (which was chosen as the monomer because of the faster oxidative addition of aryl–I than aryl–Br to Pd^69^) was absent in the spectrum of **PTd-2** (Fig. S1b), though a broad feature at slightly higher energy remained. The ^13^C CPMAS spectrum of **PTd-2** (Fig. 2b) contained two large peaks in the aromatic region; a sharper peak at 141 ppm, and a peak at 128 ppm with a shoulder at 131 ppm. A single peak at 17.5 ppm represented the methyl groups. A small peak at 106 ppm may have arisen from aromatic C atoms bound to I; the signal for these atoms in the starting material **2-I**_**4**_ occurs at 99.7 ppm in CDCl_3_ solution^59^. Upon heating in N_2_ atmosphere, **PTd-2** decomposed in three steps.

**Figure 3:**
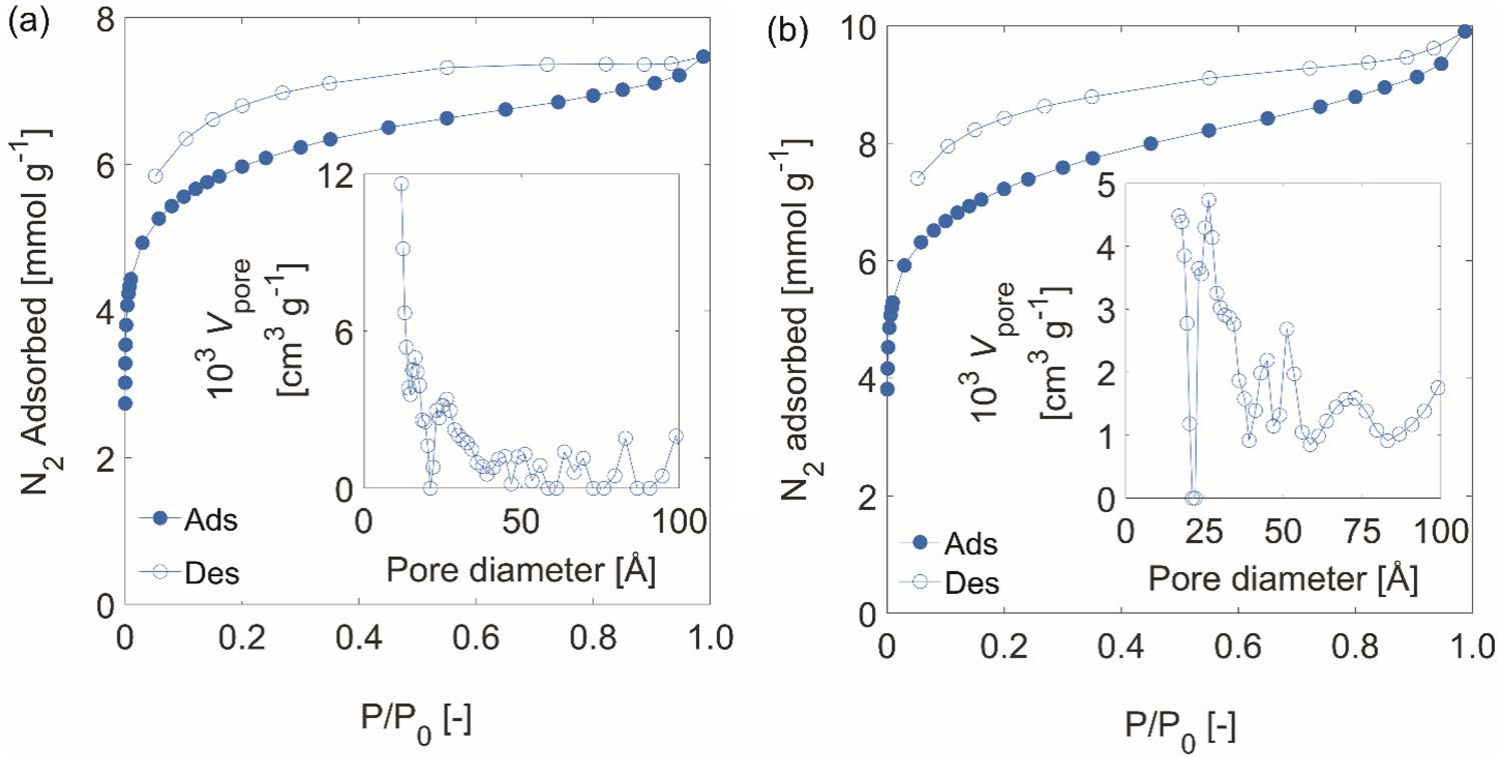
N_2_ sorption isotherms and (inset) pore size distributions for (**a**) **PTd-0** and (**b**) **PTd-2**.

The microporous natures of **PTd-0** and **PTd-2** were confirmed by N_2_ and CO_2_ adsorption. The N_2_ adsorption isotherms of **PTd-0** and **PTd-2** (Fig. 3) were both similar to the Type Ib isotherm^70^ typical for microporous polymers. However, in contrast to archetypical Type Ib isotherms, the adsorption did not reach a limiting value at relatively low P/P_0_ values, but rather continued to increase over the entire P/P_0_ range. Hysteresis was observed at all measured pressures, indicating some swelling of the polymers upon N_2_ adsorption, or the presence of restricted-access pores^71^. Hysteresis is not uncommon in microporous polymers, and has been observed in nominally two-dimensional porous polymers^72^ as well as polymers with tetrahedroid moieties like bicarbazole^31^ and hindered biphenyl^33^. The Brunauer–Emmett–Teller surface areas (*S*_BET_)^73^ of **PTd-0** and **PTd-2** were 495 and 595 m^2^ g^−1^, significantly lower than the 3420 m^2^ g^−1^ measured for a porous polymer composed of hindered biphenyl nodes connected by 1,4-enchained 1,3-butanedienyl struts^33^, and lower than the 944 m^2^ g^−1^ measured for a polymer very similar to **PTd-2**^68^. A lower surface area would be expected for **PTd-0** than for a related polymer that also incorporated rigid alkynyl linkers; however, incomplete polymerization (see above) also likely limited its surface area, as each biphenyl monomer had on average connections to three other monomers (cf. four if the polymer all C–Br bonds had been converted C–C bonds). Indeed, the surface area of hindered-biphenyl-based porous polymers can depend strongly on synthesis conditions^68^. Further, the polymer networks may be interpenetrated. The flexible biphenyl co-monomers in **PTd-2** may also allow it to exist in a more compact configuration, resulting in a lower surface area. This effect has been observed for both pyrene-^74^ and bicarbazole-based^31^ microporous polymers with biphenyl struts. Similar to the case of N_2_ adsorption, **PTd-2** adsorbed more CO_2_ than **PTd-0** did (Fig. S5). The ultramicropore size distributions for **PTd-0** and **PTd-2** could be estimated from the CO_2_ adsorption data recorded at 0 °C using a NLDFT model for carbons with slit pores (Fig. S6).

In addition to microporosity, the pore size distribution of **PTd-2** (Fig. 3b, inset and Fig. S6) also evinced mesoporosity. The N_2_-accessible pores with d ≤ 2.3 nm had a volume of 0.21 cm^3^/g, whereas those with 2.3 ≤ d ≤ 50.6 nm had a volume of 0.10 cm^3^/g, meaning that the volume in mesopores was approximately half that of the volume in micropores. We do not ascribe the mesoporosity in **PTd-2** to pores in its molecular framework, but rather its particle structure. **PTd-2** particles were composed of a loose network of intergrown particles approximately 100–150 nm in size (Fig. S3), and the mesopores detected by N_2_ sorption are likely interparticle voids in this network. **PTd-0**, on the other hand, existed as micron-sized particles built from denser collections of individual particles (Fig. S3), and had a mesopore volume (0.057 cm^3^/g) that was less than a third of its micropore volume (0.18 cm^3^/g).

**Figure 4:**
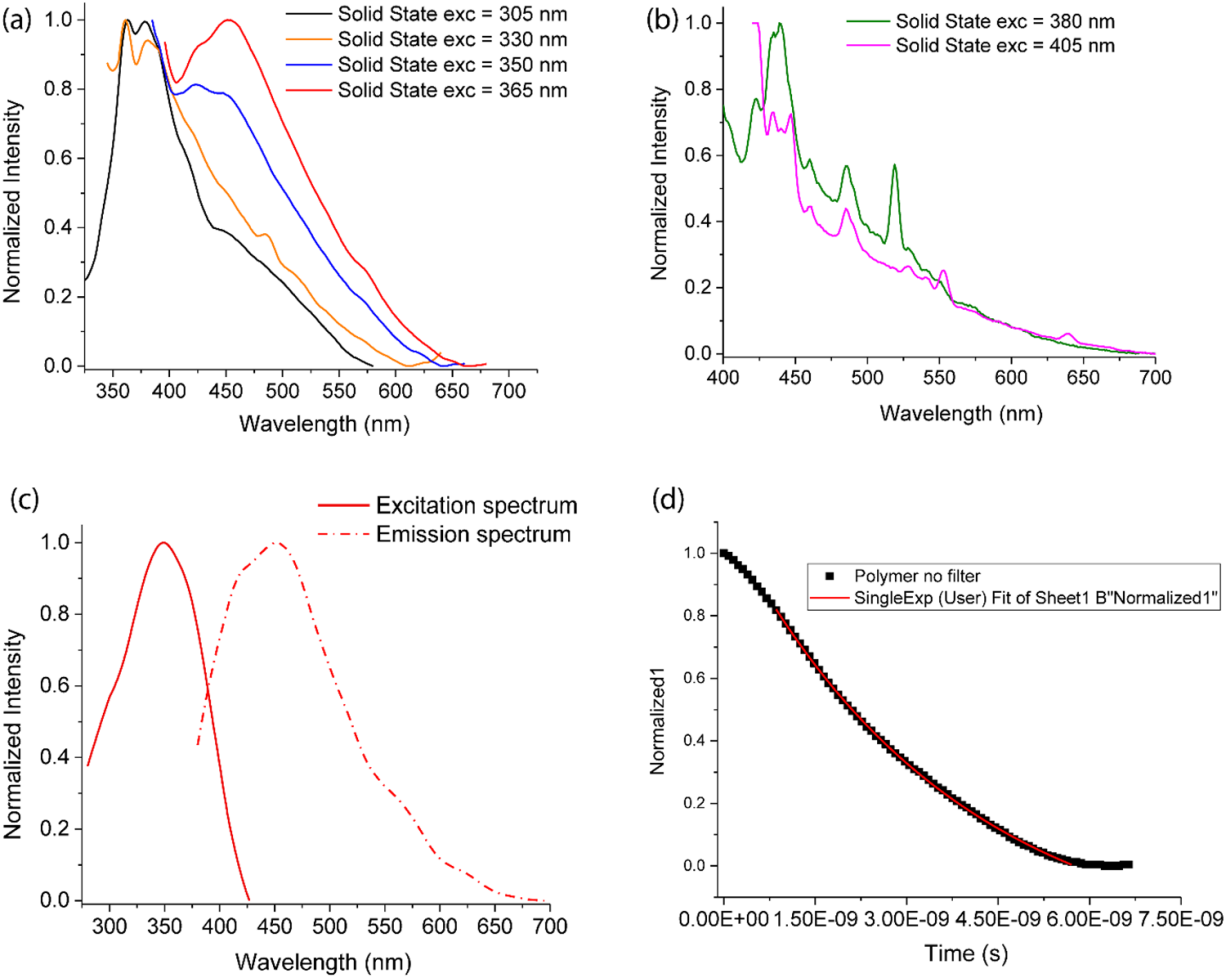
(**a**) Emission spectra of solid **PTd-0** at various excitation wavelengths. (**b**) Emission and excitation spectra of **PTd-0** in CHCl_3_ suspension. (Excitation spectrum acquired with emission at 450 nm, emission spectrum for excitation at 365 nm.) (**c**) Overlay of exictation spectra of **PTd-0** in the solution and solid states for excitation at 365 nm. (**d**) Decay of fluorescence (measured at 520 nm) from **PTd-0** in CHCl_3_ following a 450 nm laser pulse.

The microporous polymer **PTd-0** was fluorescent (Fig. 4). Light scattering by the sample in powdered form prohibited the measurement of an excitation spectrum in the solid state; when **PTd-0** was suspended in CHCl_3_, its excitation spectrum depended on the monitored emission wavelength, but consistently featured an absorption at approximately 350 nm (Fig. S7). Irradiation of powdered **PTd-0** at 305 nm produced a strong emission peak at 375 nm, as well as a shoulder at 450 nm (Fig. 4a). The latter peak occurred even when the polymer was irradiated at lower energy. Thus, in both the solid and suspended states (Fig. 4b), irradiation of **PTd-0** at λ_exc_ = 365 nm produced violet–blue fluorescence with a maximum emission at 450 nm (Fig. 4c). Steady-state photoluminescence was not observed from solid **PTd-0** with excitation at 380 or 405 nm; however, the lack of a signal after excitation at these wavelengths is likely due to the sensitivity of our instrumentation and the low absorption cross-section at the excitation wavelengths.

The absorption of higher-energy irradiation by and UV-fluorescence from **PTd-0** is readily understandable, as meta-enchained oligophenyls absorb UV light with λ_max_ close to 250 nm and behave spectroscopically as an assembly of isolated biphenyl units^75^; however, the absorption of less-energetic photons and consequent blue fluorescence is more difficult to explain. Fluorescence has not been reported previously for tetrahedroid microporous polymers based on hindered biphenyls; however, to our knowledge, it has not been studied either. Patra and co-workers^40^ and Bunz and co-workers^44,76^ have investigated analogous phenomena in microporous alternating co-polymers of (4-X-C_6_H_4_)_4_Y (Y = C, Si, Ge, Sn) and 1,4-diethynylbenzene, as fluorescence in these polymers (λ_em_ = 485–510 nm) is dramatically redshifted compared to the constituent chromophore bis(phenylethynyl)benzene (λ_em_ = 311, 360 nm). Bunz and co-workers showed that the photoluminescence properties of the microporous co-polymers of (4-X-C_6_H_4_)_4_Y (X = halogen that is removed upon polymerization; Y = C, Si, Ge, Sn) and 1,4-diethynylbenzene depend on the synthesis conditions, despite that the nominal polymers should be identical,^44,76^ and pointed out that this renders the identity of the active chromophore unclear. Similarly, **PTd-0** contained an average of one Br atom per biphenyl monomer, and can therefore not be considered an infinite network of tetramethyl-substituted biphenyls, but rather contains other moieties that could be acting as chromophores. It is also possible that the steric constraints of the **PTd-0** framework forced some phenyl groups to overlap enough to permit some through-space π-interactions and potentially excimer formation, which could produce the absorption and fluorescence at lower energy, such as in the cases of aryl-substituted germinal cyclophanes^77^, ladder-type polyphenyls^78^, bis(phenylethynyl)benzenes^79^, and poly(phenylethynyl)benzenes^80^. Excimer formation requires close contact (within 3–4 Å) between phenyl groups, which would be challenging in the sterically hindered **PTd-0**; however, π-interactions could potentially be more accessible between phenyl groups bearing unreacted C–Br moieties.

The fluorescence of **PTd-0** upon absorption of visible light allowed us to study the decay of the polymer’s excited state in CHCl_3_ suspension following 405 nm laser pulses. Fluorescence was monitored at 520 nm, which is near the emission maximum for excitation at 405 nm (Fig. S7). The excited-state decay could be modelled with a single exponential function with a fluorescence lifetime of 4 ns (Fig. 4d). However, when a long-pass filter was introduced, removing light with wavelengths below 450 nm from reaching the detector, the fluorescence decayed more rapidly, and could no longer be modelled by a single exponential function (Fig. S8). As **PTd-0** is not an infinitely dilute network of identical monomers, the excited states from the suspension may not arise from a single, uniform chromophore in a homogenous environment, but instead be self-interacting and experience local environments with a distribution of dielectric constants.

The microporous copolymer **PTd-2** contained para-enchained phenyl rings, and could thus have been expected to contain conjugated systems that would absorb and emit light at lower energies than **PTd-0**; however, **PTd-2** was not fluorescent in CHCl_3_ or toluene suspension. The powdered polymer may have been weakly fluorescent (λ_exc_ = 365 nm; λ_emission,max_ ~ 440 nm); however, the low intensity of the signal renders its assignment unclear. The unsubstituted biphenyl moieties in **PTd-2** are expected to confer higher flexibility than the hindered biphenyls (for example, the enthalpies of activation for rotational isomerization of even 2,2ʹ-disubstituted biphenyls are in the range of 100 kJ mol^−1^ even above room temperature^32^; cf. < 10 kJ mol^−1^ for unsubstituted biphenyl^81^), and may have allowed **PTd-2** to dissipate energy absorbed from irradiation through vibrations and rotations.

## Conclusions

The microporous polymer **PTd-0**, a meta-enchained and three-dimensional dehydrogenated homopolymer of a 2,2ʹ,6,6ʹ-tetramethylbiphenyl monomer, was constructed using a Yamamoto polymerization. The polymer was microporous with *S*_BET_ = 495 m^2^ g^−1^, and with approximately one unreacted Br terminus per biphenyl monomer. Despite its meta-enchained polyphenylene skeleton, **PTd-0** absorbed near-UV light and, upon irradiation at λ_exc_ = 365 nm, emitted blue fluorescence. A related alternating co-polymer, **PTd-2**, composed of the same meta-enchained 2,2ʹ,6,6ʹ-tetramethylbiphenyl core as **PTd-0** connected by 4,4ʹ-enchained biphenyl struts, had *S*_BET_ = 595 m^2^ g^−1^, but was not fluorescent.

Thus, our hypothesis that tetrahedron-like geometry of the monomers could lead to modulated optical properties was confirmed, but not in the manner we expected; rather, **PTd-0** demonstrated a significant red shift of the UV-absorption as compared to one-dimensional oligo- and poly-*m*-phenylenes, and concomitant fluorescence. In the case of **PTd-2**, the additional flexibility conferred by the biphenyl moieties led to non-radiative decay after photoexcitation.

The new monomers **2-Br**_**4**_ and **2-I**_**4**_ strengthen the arsenal available for the synthesis of microporous polymers in general. We envision future work using these monomers and related ones in the synthesis of microporous polymers with potential applications in e.g. catalysis and sensing.

### Supplementary Information

Supplementary Information 1.

Supplementary Information 2.

Supplementary Information 3.

## Data Availability

The datasets used and/or analysed during the current study available from the corresponding author on reasonable request.
