# Supplementary material for: A microporous polymer based on nonconjugated hindered biphenyls that emits blue light
Source: Sci Rep. 2024 Jun 28;14:14923. doi: 10.1038/s41598-024-65743-5 (PMC11213909; doi:10.1038/s41598-024-65743-5)
Supplement: Supplementary file 2 — Supplementary Information 2. [file 41598_2024_65743_MOESM2_ESM.docx]

**Supporting Information for**

**A Blue-Emitting Fluorescent Microporous Polymer Composed of meta-Enchained Hindered Biphenyls**

Tamara L. Church,^a^ Lars Eriksson,^a^ Valentina Leandri,^b,c^ James M. Gardner,^b^ and Niklas Hedin^a,*^

^a^ Department of Materials and Environmental Chemistry, Stockholm University, Stockholm, 106 91 Sweden

^b^ Department of Chemistry, Applied Physical Chemistry, KTH Royal Institute of Technology, Stockholm 10044, Sweden

^c^ RISE Chemical Process and Pharmaceutical Development, Forskargatan 20J, Södertälje 15136, Sweden

| Item | Page |
| --- | --- |
| S1. Infrared spectra of monomers **2-X_4_** and polymers **PTd-A** | S1 |
| S2. Crystal structure of **2-I_4_** | S2 |
| S3. Scanning electron microscopy of the **PTd-A** polymers | S3 |
| S4. Thermogravimetric analysis of the **PTd-A** polymers | S4 |
| S5. Additional gas sorption data | S4 |
| S6. Additional fluorescence data for **PTd-0** in CHCl_3_ suspension | S5 |
| S7. NMR spectra of **PTd-0** | S7 |
| S8. NMR spectra of **PTd-2** | S9 |
| S9. References | S11 |

S1. Infrared spectra of monomers **2-X_4_** and polymers **PTd-A**

**Figure S1.** Attenuated total reflectance IR spectra of (a) **PTd-0** and its monomer **2-Br_4_**, and (b) **PTd-2** and its monomer **2-I_4_**.

S2. Crystal structure of **2-I_4_**

Compound **2-I_4_** crystallized as colourless needles. Single crystal X-ray diffraction data was obtained at room temperature, solved using Shelxt,^1^ and refined using Shelxl.^2^ Details of the structure solution are given in Chart S1, and a rendering of the molecule is shown in Figure S2. The structure was solved in the monoclinic space group P2_1_/c (14); despite that α and γ varied slightly from 90°, R(int) for the Laue group 2/m was 0.0428. The asymmetric unit contained one molecule of **2-I_4_**. The structure was refined for reflections with resolution between 0.8 and 99 Å.

**Figure S2**. X-ray c
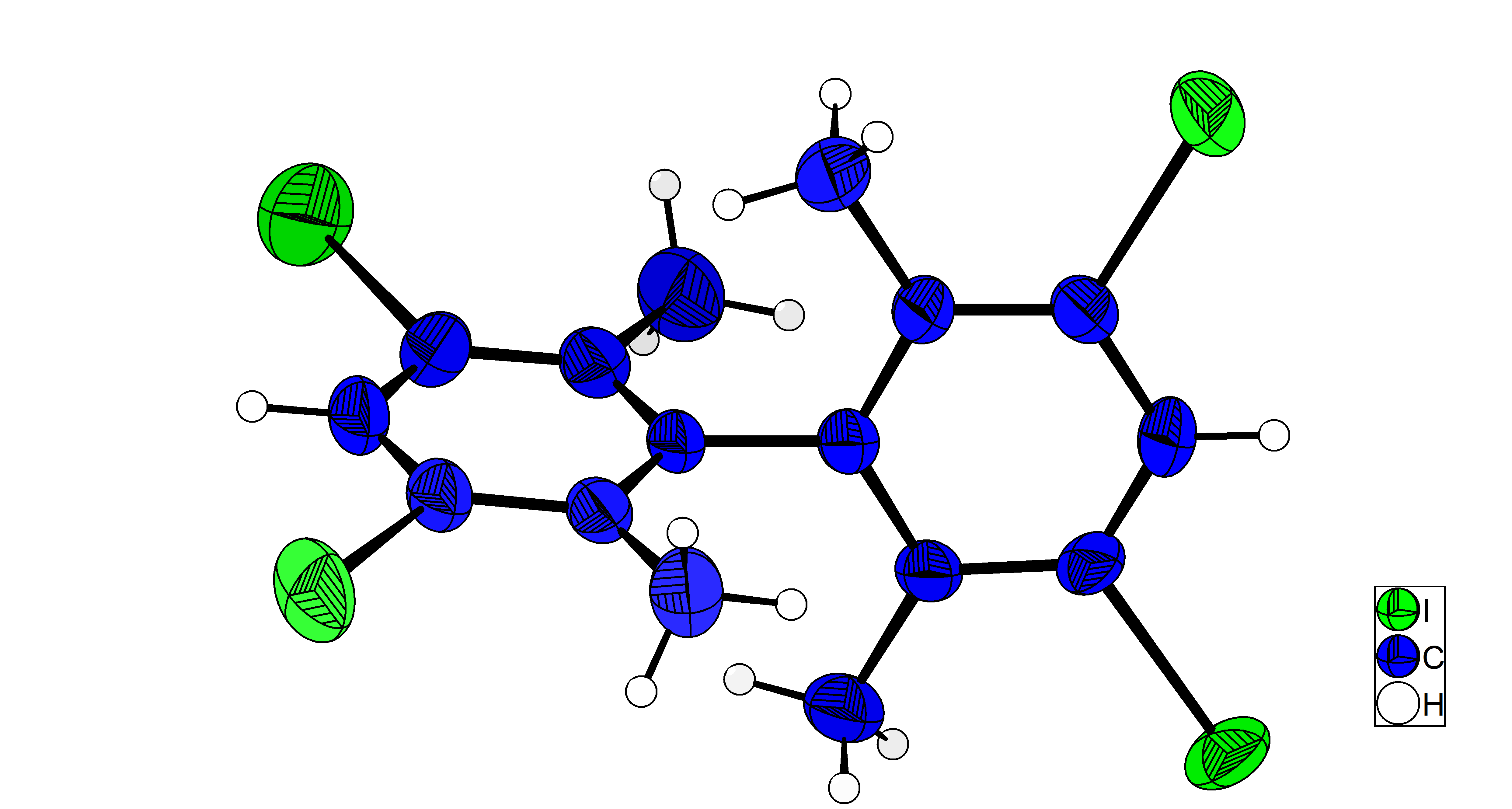
rystal structure of **2-I_4_**. Blue ellipsoids represent C atoms, green ellipsoids represent I atoms, and open circles represent H atoms.

**Chart S1**. Details of the X-ray crystal structure solution for **2-I_4_**.

| Parameter | Value |  | Parameter | Value |
| --- | --- | --- | --- | --- |
| *Data collection* | | | | |
| Temperature | Room temperature |  | Wavelength | 0.71073 Å |
| *Structure solution* | | | | |
| Space group | P2_1_/c |  | Resolution | 0.8 Å |
| a | 9.0436(5) Å |  |  |  |
| b | 12.1873(6) Å |  | Rint | 0.0428 |
| c | 17.4292(9) Å |  | R1 for F_o_ > 4σ(F_o_) | 0.0403 |
| *α* | 90.003(2)° |  | wR2 | 0.1299 |
| *β* | 98.137(2)° |  | GooF | 1.050 |
| *γ* | 89.913(2)° |  | Reflections (observed) | 3880 (3401) |
| Z | 4 |  | Parameters (restraints) | 185 (0) |

S3. Scanning electron microscopy of the **PTd-A** polymers

6


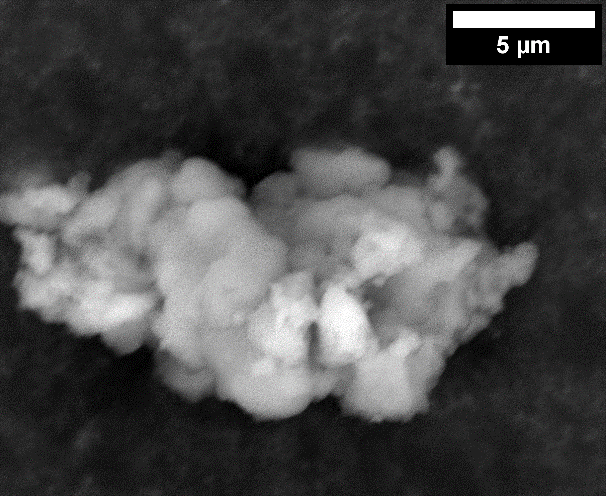


1

3

4

5

2

(a)

| Spot | Atom% | | | |
| --- | --- | --- | --- | --- |
|  | C | O | P | Br |
| 1 | 91 | 7.0 | 0.4 | 1.5 |
| 2 | 93 | 6.5 | 0.2 | 0.8 |
| 3 | 92 | 6.7 | 0.2 | 0.9 |
| 4 | 93 | 5.9 | 0.3 | 0.7 |
| 5 | 93 | 6.3 | 0.2 | 0.5 |
| 6 | 93 | 6.7 | 0 | 0.1 |


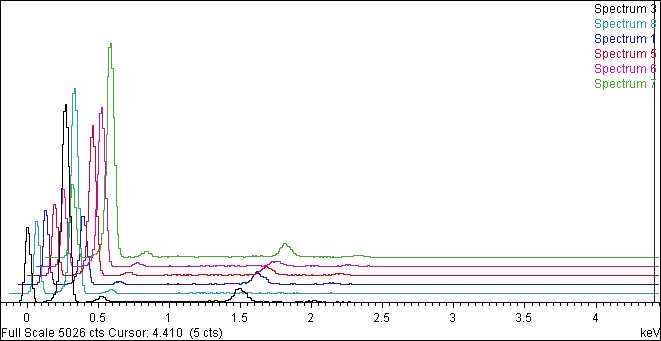


(b)

1

2

3

4

5

6

Energy [keV]


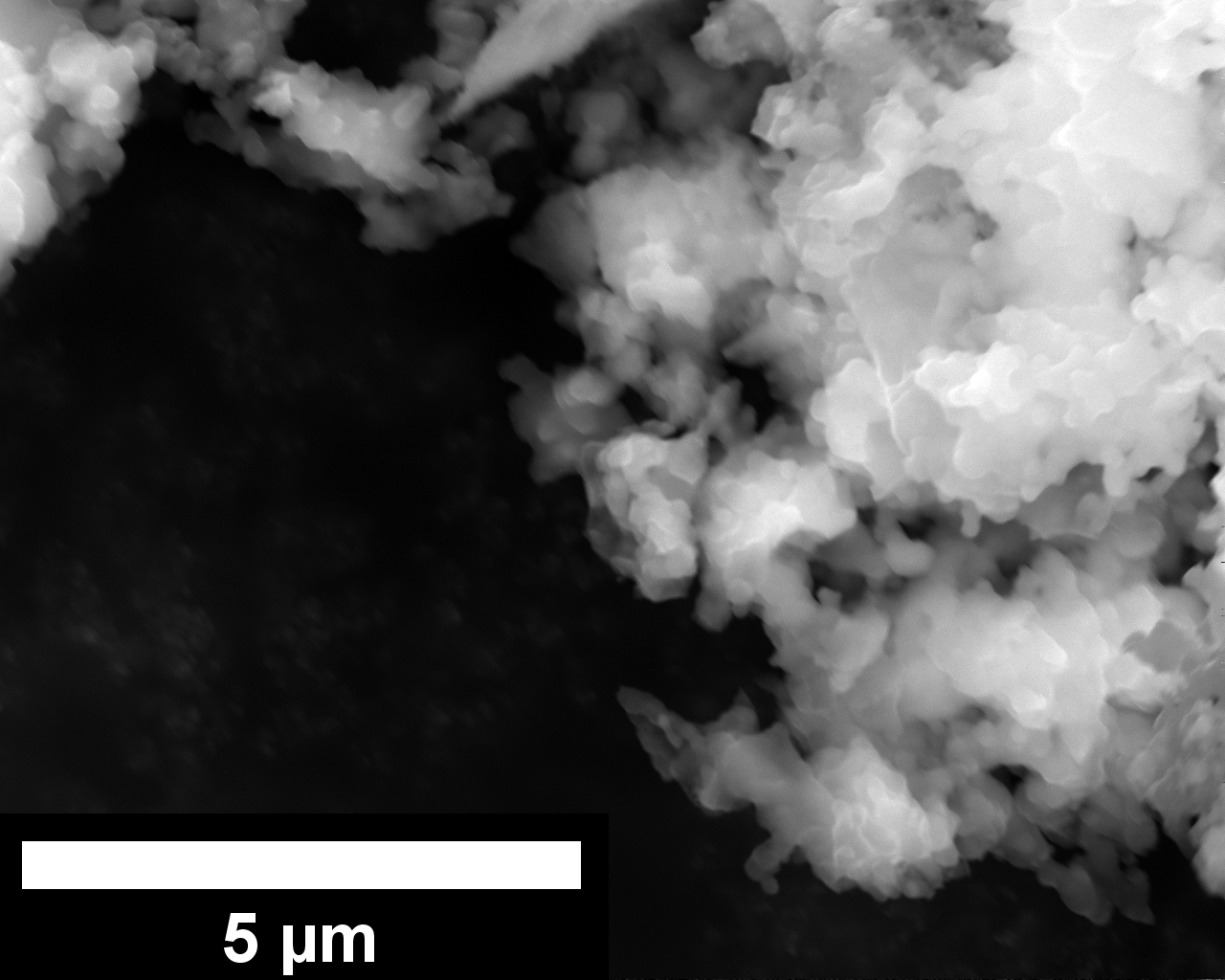

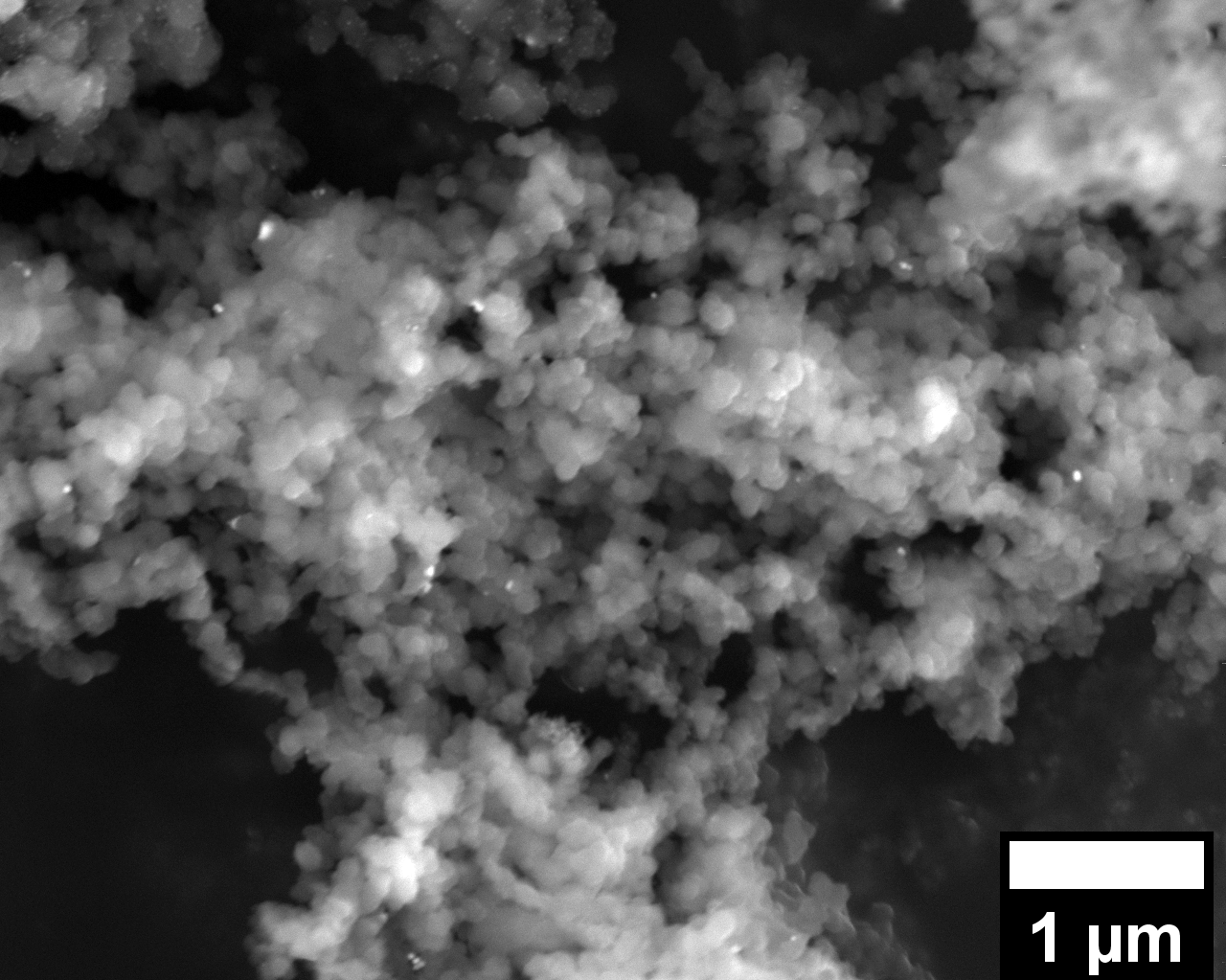


(c)

(d)

**Figure S3**. SEM images and energy dispersive X-ray spectra of the microporous polymers. (a) **PTd-0**. Numbers indicate spots analyzed using energy-dispersive X-ray spectroscopy (b); results are given at right. (c,d) **PTd-2**.

S4. Thermogravimetric analysis of **PTd-A** polymers

Atmosphere changed from N_2_

to air

**Figure S4**. (a) Thermogravimetric analysis of microporous polymers **PTd-0** and **PTd-2**. Under a flow of 25 mL min^−1^ N_2_, samples were held at 100 °C for 30 min, then heated at 5 °C min^−1^. When the temperature reached 1000 °C, the atmosphere was switched to 25 mL min^−1^ air.

S5. Additional gas sorption data


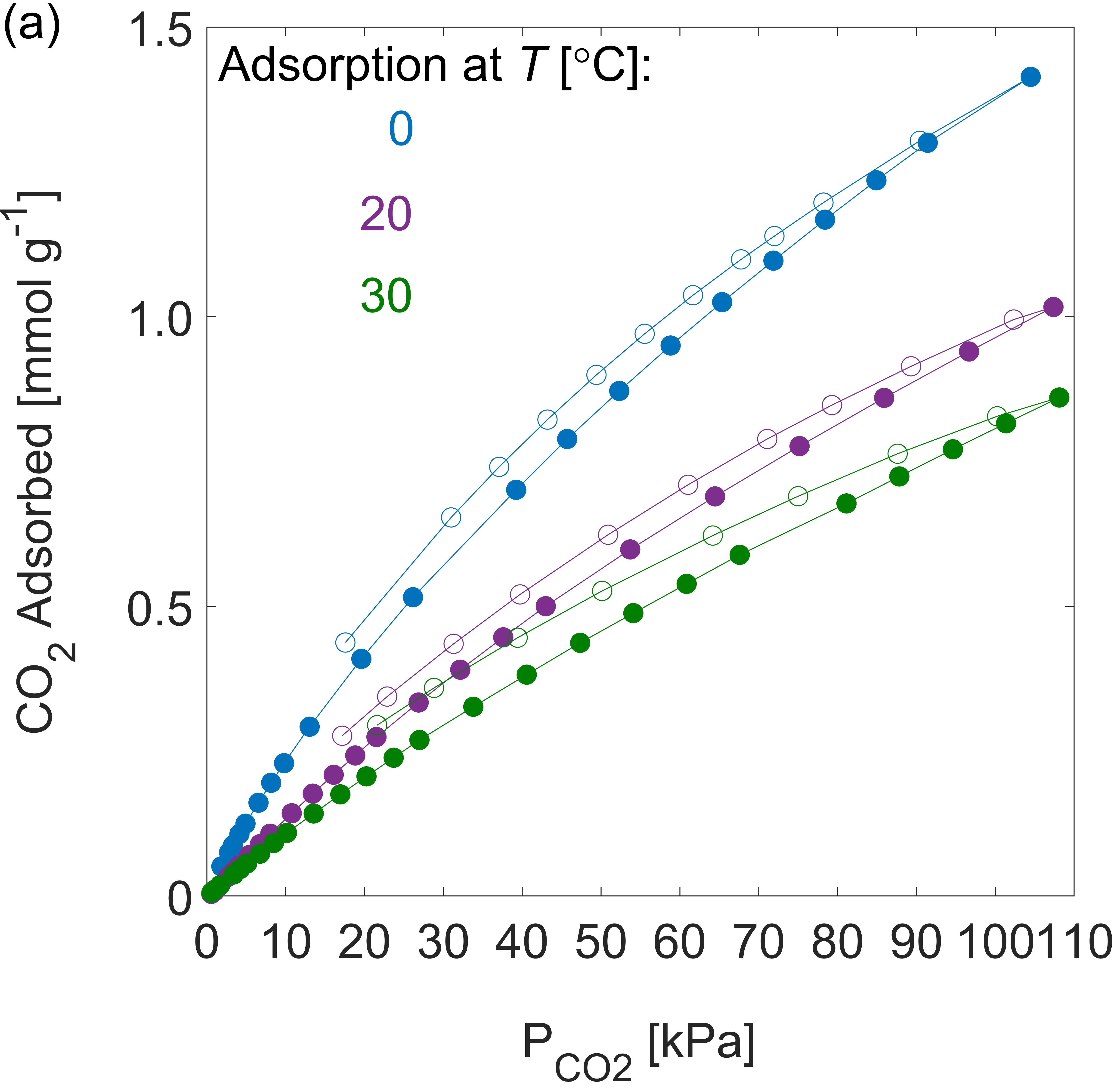

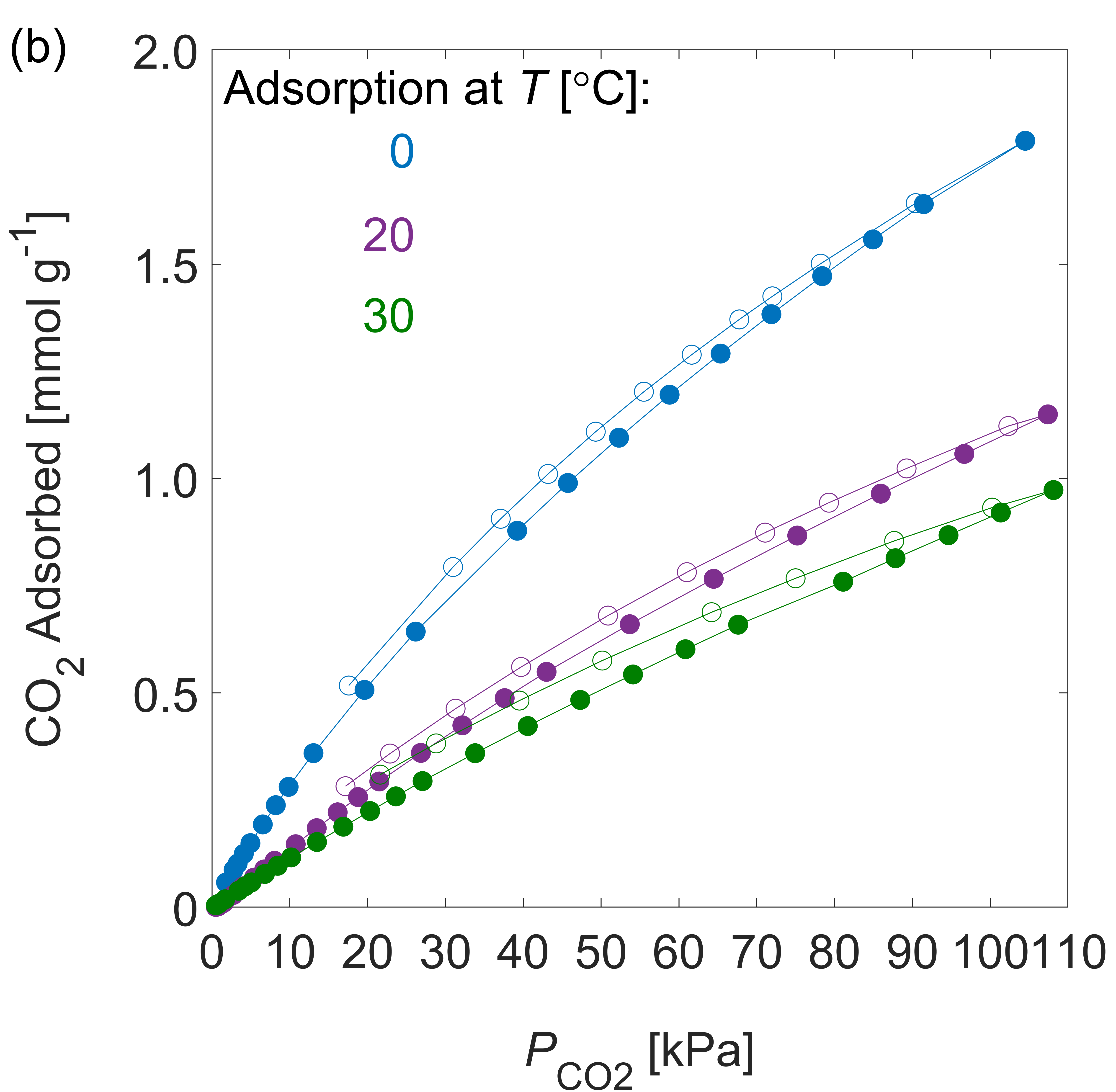


**Figure S5**. CO_2_ sorption on (a) **PTd-0** and (b) **PTd-2**, collected at 0, 20, and 30 °C.


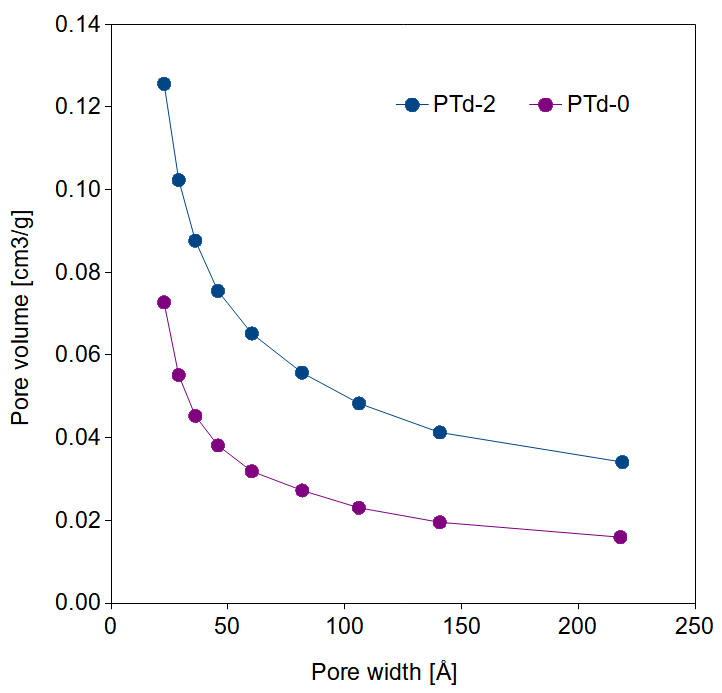

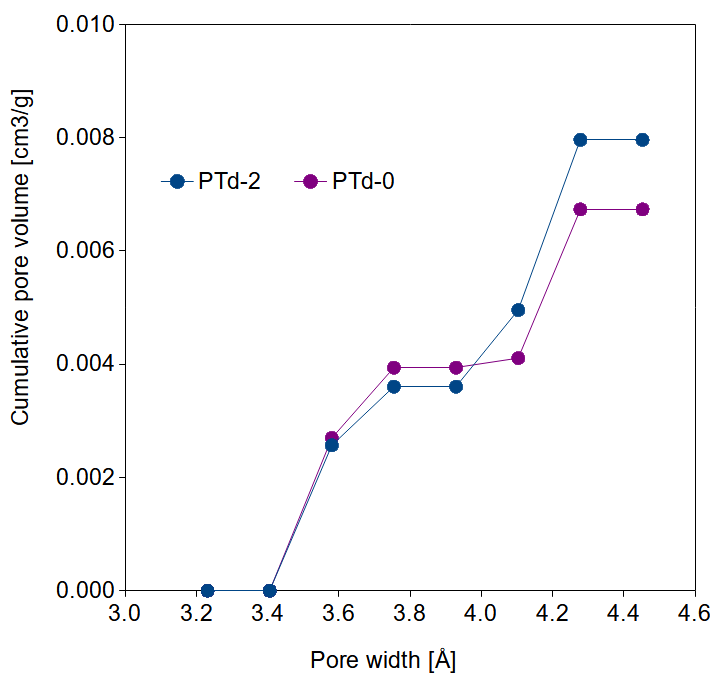


(a)

(b)

**Figure S6**. Additional pore-distribution data for **PTd-0** and **PTd-2**. (a) Barrett–Joyner–Halenda pore size distributions, calculated from N_2_ adsorption isotherms. (b) NLDFT ultramicropore distributions (cumulative) calculated from CO_2_ adsorption isotherms.

S6. Additional fluorescence data for **PTd-0** in CHCl_3_ suspension

(a) (b)

**Figure S7**. (a) Excitation and (b) emission spectra of **PTd-0** in CHCl_3_ suspension.

**Figure S8**. Fluorescence decay collected at 520 nm from a suspension of **PTd-0** in CHCl_3_ following a laser pulse at 405 nm, with an without a 450-nm long-pass filter.

S7. NMR spectra of **PTd-0**


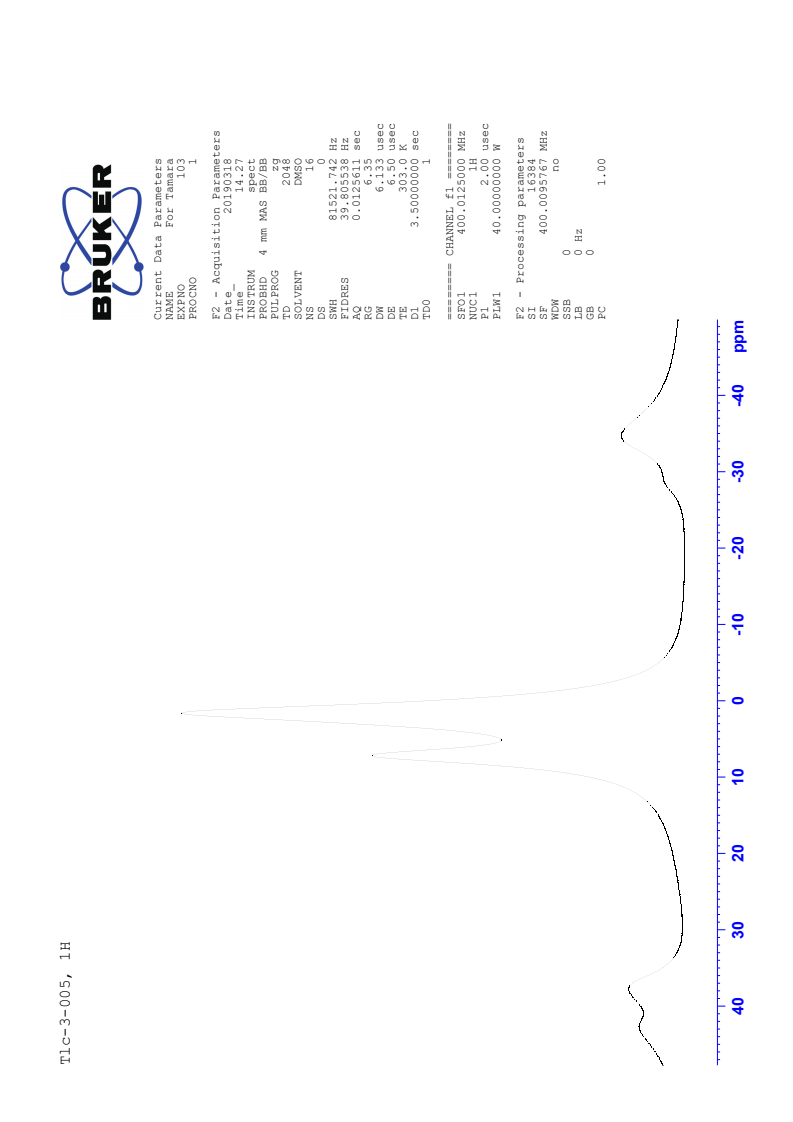


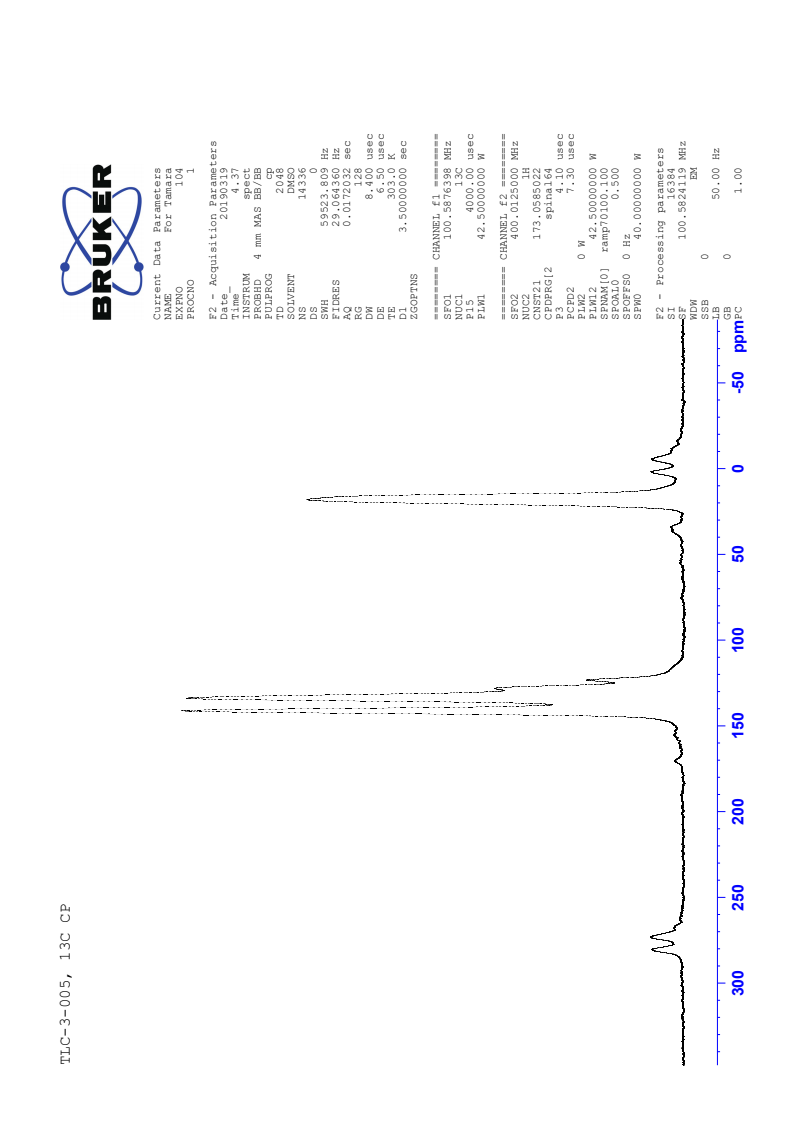


S8. NMR spectra of **PTd-2**


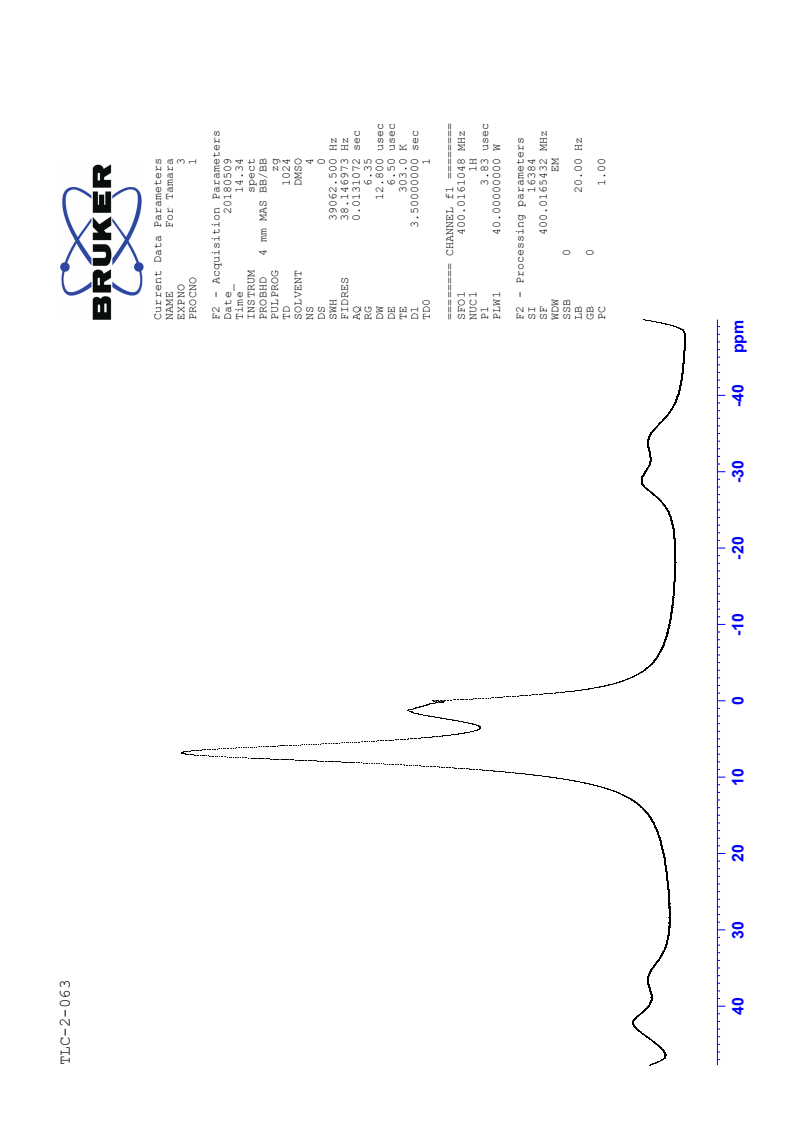


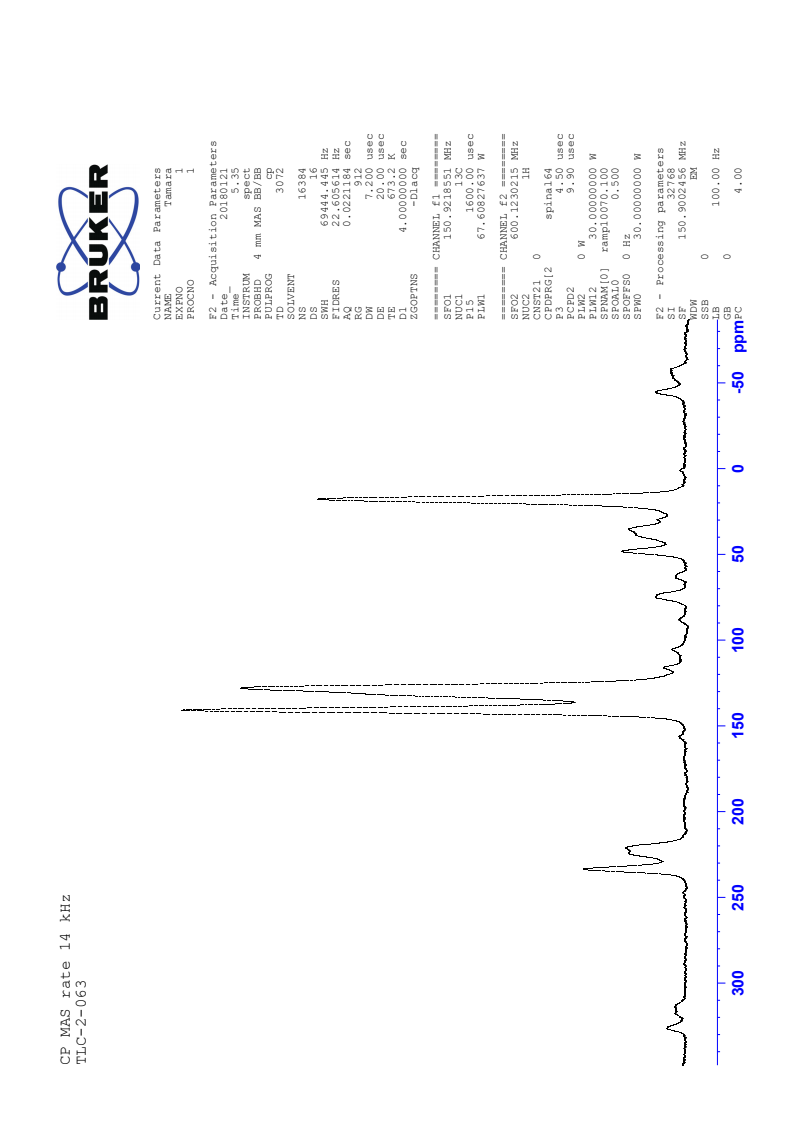


S9. References

(1) Sheldrick, G. M. A Short History of SHELX. *Acta Crystallogr. Sect. A Found. Crystallogr.* **2008**, *64* (1), 112–122. https://doi.org/10.1107/S0108767307043930.

(2) Sheldrick, G. M. Crystal Structure Refinement with SHELXL. *Acta Crystallogr. Sect. C Struct. Chem.* **2015**, *71* (1), 3–8. https://doi.org/10.1107/S2053229614024218.

(3) Langmuir, I. THE ADSORPTION OF GASES ON PLANE SURFACES OF GLASS, MICA AND PLATINUM. *J. Am. Chem. Soc.* **1918**, *40* (9), 1361–1403. https://doi.org/10.1021/ja02242a004.
