# Supplementary material for: A microporous polymer based on nonconjugated hindered biphenyls that emits blue light
Source: Sci Rep. 2024 Jun 28;14:14923. doi: 10.1038/s41598-024-65743-5 (PMC11213909; doi:10.1038/s41598-024-65743-5)
Supplement: Supplementary file 3 — Supplementary Information 3. [file 41598_2024_65743_MOESM3_ESM.docx]

data_3,3',5,5'-tetraiodo-2,2'-6,6'-tetramethylbiphenyl

_publ_contact_author_name 'Hedin, Niklas'

_publ_contact_author_address 'See below'

_publ_contact_author_email 'niklas.hedin@mmk.su.se'

_publ_requested_journal 'Materials Chemistry Frontiers'

_publ_section_title 'A Blue-Emitting Fluorescent Microporous Polymer Composed of meta-Enchained Hindered Biphenyls'

loop_

_publ_author_name

_publ_author_address

'Church, Tamara L.'

; Department of Materials and Environmental Chemistry

Stockholm University

10691 Stockholm

Sweden

;

'Eriksson, Lars'

; Department of Materials and Environmental Chemistry

Stockholm University

10691 Stockholm

Sweden

;

'Leandri, Valentina'

; Department of Chemistry

Applied Physical Chemistry

KTH Royal Institute of Technology

Stockholm 10044

Sweden

RISE Chemical Process and Pharmaceutical Development

Forskargatan 20J

Södertälje 15136

Sweden

;

'Gardner, James M.'

; Department of Chemistry

Applied Physical Chemistry

KTH Royal Institute of Technology

Stockholm 10044

Sweden

;

'Hedin, Niklas'

; Department of Materials and Environmental Chemistry

Stockholm University

10691 Stockholm

Sweden

;

_publ_section_abstract 'Microporous organic polymers that have three-dimensional connectivity stemming from monomers with tetrahedral or tetrahedron-like geometry can have high surface areas and interesting optical properties, such as the ability to transfer photoexcitation energy to other molecules. We assembled 2,2',6,6'-tetramethylbiphenyl monomers, in which the two phenyl rings are nearly mutually perpendicular, into a meta-enchained homopolymer. The polymer was microporous with SBET = 495 m2 g-1 and adsorbed 0.31 mmol CO2 g-1 at 0 degreesC and 15 kPa. Despite being a meta-enchained polyphenylene, the polymer absorbed near-UV light and emitted blue fluorescence with tau = 4 ns; however, it could not transfer photoexcitation energy to a dye adsorbed on the outside of a polymer particle. A related copolymer, in which the 2,2',6,6'-tetramethylbiphenyl monomers were separated with para-enchained unsubstituted biphenyls, was microporous but not fluorescent.'

_audit_creation_method 'SHELXL-2016/6'

_shelx_SHELXL_version_number '2016/6'

_chemical_name_systematic '3,3',5,5'-tetraiodo-2,2'-6,6'-tetramethylbiphenyl'

_chemical_name_common ?

_chemical_melting_point ?

_chemical_formula_moiety ?

_chemical_formula_sum

'C16 H14 I4'

_chemical_formula_weight 713.87

loop_

_atom_type_symbol

_atom_type_description

_atom_type_scat_dispersion_real

_atom_type_scat_dispersion_imag

_atom_type_scat_source

'C' 'C' 0.0033 0.0016

'International Tables Vol C Tables 4.2.6.8 and 6.1.1.4'

'H' 'H' 0.0000 0.0000

'International Tables Vol C Tables 4.2.6.8 and 6.1.1.4'

'I' 'I' -0.4742 1.8119

'International Tables Vol C Tables 4.2.6.8 and 6.1.1.4'

_space_group_crystal_system monoclinic

_space_group_IT_number 14

_space_group_name_H-M_alt 'P 21/c'

_space_group_name_Hall '-P 2ybc'

_shelx_space_group_comment

;

The symmetry employed for this shelxl refinement is uniquely defined

by the following loop, which should always be used as a source of

symmetry information in preference to the above space-group names.

They are only intended as comments.

;

loop_

_space_group_symop_operation_xyz

'x, y, z'

'-x, y+1/2, -z+1/2'

'-x, -y, -z'

'x, -y-1/2, z-1/2'

_cell_length_a 9.0436(5)

_cell_length_b 12.1873(6)

_cell_length_c 17.4292(9)

_cell_angle_alpha 90.003(2)

_cell_angle_beta 98.137(2)

_cell_angle_gamma 89.913(2)

_cell_volume 1901.65(17)

_cell_formula_units_Z 4

_cell_measurement_temperature 293(2)

_cell_measurement_reflns_used 9881

_cell_measurement_theta_min 2.361

_cell_measurement_theta_max 33.434

_exptl_crystal_description needle

_exptl_crystal_colour colourless

_exptl_crystal_density_meas ?

_exptl_crystal_density_method ?

_exptl_crystal_density_diffrn 2.493

_exptl_crystal_F_000 1288

_exptl_transmission_factor_min ?

_exptl_transmission_factor_max ?

_exptl_crystal_size_max 1

_exptl_crystal_size_mid 0.5

_exptl_crystal_size_min 0.5

_exptl_absorpt_coefficient_mu 6.544

_shelx_estimated_absorpt_T_min ?

_shelx_estimated_absorpt_T_max ?

_exptl_absorpt_correction_type multi-scan

_exptl_absorpt_correction_T_min 0.5076

_exptl_absorpt_correction_T_max 0.7467

_exptl_absorpt_process_details

;

SADABS 2016/2: Krause, L., Herbst-Irmer, R., Sheldrick G.M. & Stalke D.,

J. Appl. Cryst. 48 (2015) 3-10

;

_exptl_absorpt_special_details ?

_diffrn_ambient_temperature 293(2)

_diffrn_radiation_wavelength 0.71073

_diffrn_radiation_type MoK\a

_diffrn_source ?

_diffrn_measurement_device_type 'Bruker D8 VENTURE'

_diffrn_measurement_method '\w and \f scans to cover reciprocal space'

_diffrn_standards_number 0

_diffrn_standards_interval_time 0

_diffrn_standards_decay_% 0

_diffrn_detector_area_resol_mean ?

_diffrn_reflns_number 26929

_diffrn_reflns_av_unetI/netI 0.0191

_diffrn_reflns_av_R_equivalents 0.0428

_diffrn_reflns_limit_h_min -11

_diffrn_reflns_limit_h_max 11

_diffrn_reflns_limit_k_min -15

_diffrn_reflns_limit_k_max 15

_diffrn_reflns_limit_l_min -21

_diffrn_reflns_limit_l_max 21

_diffrn_reflns_theta_min 2.275

_diffrn_reflns_theta_max 26.371

_diffrn_reflns_theta_full 25.242

_diffrn_measured_fraction_theta_max 0.997

_diffrn_measured_fraction_theta_full 0.999

_diffrn_reflns_Laue_measured_fraction_max 0.997

_diffrn_reflns_Laue_measured_fraction_full 0.999

_diffrn_reflns_point_group_measured_fraction_max 0.997

_diffrn_reflns_point_group_measured_fraction_full 0.999

_reflns_number_total 3880

_reflns_number_gt 3401

_reflns_threshold_expression 'I > 2\s(I)'

_reflns_Friedel_coverage 0.000

_reflns_Friedel_fraction_max .

_reflns_Friedel_fraction_full .

_reflns_special_details

;

Reflections were merged by SHELXL according to the crystal

class for the calculation of statistics and refinement.

_reflns_Friedel_fraction is defined as the number of unique

Friedel pairs measured divided by the number that would be

possible theoretically, ignoring centric projections and

systematic absences.

;

_computing_data_collection ?

_computing_cell_refinement ?

_computing_data_reduction ?

_computing_structure_solution ?

_computing_structure_refinement 'SHELXL-2016/6 (Sheldrick, 2016)'

_computing_molecular_graphics ?

_computing_publication_material ?

_refine_special_details ?

_refine_ls_structure_factor_coef Fsqd

_refine_ls_matrix_type full

_refine_ls_weighting_scheme calc

_refine_ls_weighting_details

'w=1/[\s^2^(Fo^2^)+(0.0750P)^2^+6.9623P] where P=(Fo^2^+2Fc^2^)/3'

_atom_sites_solution_primary ?

_atom_sites_solution_secondary ?

_atom_sites_solution_hydrogens geom

_refine_ls_hydrogen_treatment constr

_refine_ls_extinction_method none

_refine_ls_extinction_coef .

_refine_ls_number_reflns 3880

_refine_ls_number_parameters 185

_refine_ls_number_restraints 0

_refine_ls_R_factor_all 0.0456

_refine_ls_R_factor_gt 0.0403

_refine_ls_wR_factor_ref 0.1299

_refine_ls_wR_factor_gt 0.1240

_refine_ls_goodness_of_fit_ref 1.050

_refine_ls_restrained_S_all 1.050

_refine_ls_shift/su_max 0.000

_refine_ls_shift/su_mean 0.000

loop_

_atom_site_label

_atom_site_type_symbol

_atom_site_fract_x

_atom_site_fract_y

_atom_site_fract_z

_atom_site_U_iso_or_equiv

_atom_site_adp_type

_atom_site_occupancy

_atom_site_site_symmetry_order

_atom_site_calc_flag

_atom_site_refinement_flags_posn

_atom_site_refinement_flags_adp

_atom_site_refinement_flags_occupancy

_atom_site_disorder_assembly

_atom_site_disorder_group

I1 I 0.82344(6) 0.58103(4) 0.05066(2) 0.05821(17) Uani 1 1 d . . . . .

I2 I 0.93217(8) 0.86423(5) 0.32852(3) 0.0794(2) Uani 1 1 d . . . . .

I3 I 0.63561(7) 0.19752(4) 0.45814(3) 0.06920(19) Uani 1 1 d . . . . .

I4 I 0.14724(6) 0.52186(6) 0.40753(4) 0.0819(2) Uani 1 1 d . . . . .

C1 C 0.6670(7) 0.5683(5) 0.2762(3) 0.0399(12) Uani 1 1 d . . . . .

C2 C 0.6828(7) 0.5437(4) 0.1991(3) 0.0394(12) Uani 1 1 d . . . . .

C3 C 0.7785(7) 0.6126(5) 0.1638(3) 0.0404(12) Uani 1 1 d . . . . .

C4 C 0.8466(8) 0.7011(5) 0.1997(3) 0.0473(14) Uani 1 1 d . . . . .

H4 H 0.907238 0.745538 0.173997 0.057 Uiso 1 1 calc R U . . .

C5 C 0.8253(8) 0.7250(5) 0.2748(3) 0.0472(14) Uani 1 1 d . . . . .

C6 C 0.7385(8) 0.6578(5) 0.3159(3) 0.0466(14) Uani 1 1 d . . . . .

C7 C 0.6019(10) 0.4486(6) 0.1586(4) 0.0564(17) Uani 1 1 d . . . . .

H7A H 0.670301 0.388803 0.156247 0.085 Uiso 1 1 calc R U . . .

H7B H 0.560306 0.469897 0.107032 0.085 Uiso 1 1 calc R U . . .

H7C H 0.523012 0.425979 0.186622 0.085 Uiso 1 1 calc R U . . .

C8 C 0.7192(11) 0.6807(7) 0.3981(4) 0.068(2) Uani 1 1 d . . . . .

H8A H 0.814336 0.697938 0.427397 0.102 Uiso 1 1 calc R U . . .

H8B H 0.678221 0.617248 0.419932 0.102 Uiso 1 1 calc R U . . .

H8C H 0.652510 0.741798 0.399765 0.102 Uiso 1 1 calc R U . . .

C9 C 0.5736(7) 0.4966(4) 0.3200(3) 0.0394(12) Uani 1 1 d . . . . .

C10 C 0.6358(7) 0.4001(5) 0.3549(3) 0.0405(12) Uani 1 1 d . . . . .

C11 C 0.5504(7) 0.3412(5) 0.4012(3) 0.0440(13) Uani 1 1 d . . . . .

C12 C 0.4113(8) 0.3753(5) 0.4133(4) 0.0483(14) Uani 1 1 d . . . . .

H12 H 0.356962 0.334664 0.444815 0.058 Uiso 1 1 calc R U . . .

C13 C 0.3528(7) 0.4691(6) 0.3788(4) 0.0487(14) Uani 1 1 d . . . . .

C14 C 0.4291(7) 0.5313(5) 0.3300(4) 0.0451(13) Uani 1 1 d . . . . .

C15 C 0.7893(8) 0.3647(6) 0.3447(4) 0.0567(16) Uani 1 1 d . . . . .

H15A H 0.854875 0.374032 0.392629 0.085 Uiso 1 1 calc R U . . .

H15B H 0.824263 0.408455 0.305149 0.085 Uiso 1 1 calc R U . . .

H15C H 0.787913 0.288879 0.329781 0.085 Uiso 1 1 calc R U . . .

C16 C 0.3626(10) 0.6340(6) 0.2886(6) 0.070(2) Uani 1 1 d . . . . .

H16A H 0.272615 0.615043 0.255135 0.105 Uiso 1 1 calc R U . . .

H16B H 0.433140 0.664789 0.258397 0.105 Uiso 1 1 calc R U . . .

H16C H 0.340088 0.686712 0.326215 0.105 Uiso 1 1 calc R U . . .

loop_

_atom_site_aniso_label

_atom_site_aniso_U_11

_atom_site_aniso_U_22

_atom_site_aniso_U_33

_atom_site_aniso_U_23

_atom_site_aniso_U_13

_atom_site_aniso_U_12

I1 0.0942(4) 0.0524(3) 0.0331(2) 0.00595(16) 0.0268(2) 0.0159(2)

I2 0.1121(5) 0.0668(3) 0.0649(3) -0.0197(2) 0.0313(3) -0.0507(3)

I3 0.0913(4) 0.0533(3) 0.0613(3) 0.0255(2) 0.0051(3) -0.0070(2)

I4 0.0586(3) 0.0930(5) 0.0983(5) -0.0095(3) 0.0255(3) -0.0061(3)

C1 0.057(3) 0.030(3) 0.034(3) 0.002(2) 0.015(2) -0.005(2)

C2 0.062(3) 0.028(2) 0.029(3) 0.001(2) 0.011(2) -0.002(2)

C3 0.065(4) 0.035(3) 0.024(2) 0.004(2) 0.015(2) 0.004(3)

C4 0.062(4) 0.046(3) 0.038(3) 0.009(2) 0.019(3) -0.010(3)

C5 0.070(4) 0.037(3) 0.037(3) -0.004(2) 0.013(3) -0.016(3)

C6 0.070(4) 0.042(3) 0.032(3) -0.003(2) 0.019(3) -0.016(3)

C7 0.089(5) 0.041(3) 0.041(3) -0.007(3) 0.013(3) -0.013(3)

C8 0.105(6) 0.067(5) 0.036(3) -0.017(3) 0.027(4) -0.035(4)

C9 0.058(3) 0.032(3) 0.031(2) -0.002(2) 0.015(2) -0.013(2)

C10 0.057(3) 0.033(3) 0.032(3) -0.001(2) 0.008(2) -0.008(2)

C11 0.059(3) 0.040(3) 0.034(3) 0.003(2) 0.009(2) -0.010(3)

C12 0.064(4) 0.045(3) 0.038(3) 0.004(2) 0.015(3) -0.017(3)

C13 0.052(3) 0.053(4) 0.044(3) -0.004(3) 0.016(3) -0.008(3)

C14 0.059(4) 0.036(3) 0.042(3) 0.000(2) 0.010(3) -0.004(3)

C15 0.057(4) 0.054(4) 0.060(4) 0.007(3) 0.015(3) -0.007(3)

C16 0.081(5) 0.047(4) 0.086(6) 0.013(4) 0.026(4) 0.011(4)

_geom_special_details

;

All esds (except the esd in the dihedral angle between two l.s. planes)

are estimated using the full covariance matrix. The cell esds are taken

into account individually in the estimation of esds in distances, angles

and torsion angles; correlations between esds in cell parameters are only

used when they are defined by crystal symmetry. An approximate (isotropic)

treatment of cell esds is used for estimating esds involving l.s. planes.

;

loop_

_geom_bond_atom_site_label_1

_geom_bond_atom_site_label_2

_geom_bond_distance

_geom_bond_site_symmetry_2

_geom_bond_publ_flag

I1 C3 2.105(5) . ?

I2 C5 2.107(6) . ?

I3 C11 2.103(6) . ?

I4 C13 2.093(7) . ?

C1 C2 1.403(8) . ?

C1 C6 1.401(8) . ?

C1 C9 1.499(7) . ?

C2 C3 1.408(8) . ?

C2 C7 1.495(9) . ?

C3 C4 1.352(9) . ?

C4 C5 1.382(8) . ?

C4 H4 0.9300 . ?

C5 C6 1.400(8) . ?

C6 C8 1.494(8) . ?

C7 H7A 0.9600 . ?

C7 H7B 0.9600 . ?

C7 H7C 0.9600 . ?

C8 H8A 0.9600 . ?

C8 H8B 0.9600 . ?

C8 H8C 0.9600 . ?

C9 C10 1.403(8) . ?

C9 C14 1.407(9) . ?

C10 C11 1.393(8) . ?

C10 C15 1.488(9) . ?

C11 C12 1.369(10) . ?

C12 C13 1.363(10) . ?

C12 H12 0.9300 . ?

C13 C14 1.392(9) . ?

C14 C16 1.525(10) . ?

C15 H15A 0.9600 . ?

C15 H15B 0.9600 . ?

C15 H15C 0.9600 . ?

C16 H16A 0.9600 . ?

C16 H16B 0.9600 . ?

C16 H16C 0.9600 . ?

loop_

_geom_angle_atom_site_label_1

_geom_angle_atom_site_label_2

_geom_angle_atom_site_label_3

_geom_angle

_geom_angle_site_symmetry_1

_geom_angle_site_symmetry_3

_geom_angle_publ_flag

C2 C1 C6 122.5(5) . . ?

C2 C1 C9 120.3(5) . . ?

C6 C1 C9 117.2(5) . . ?

C1 C2 C3 116.3(5) . . ?

C1 C2 C7 120.5(5) . . ?

C3 C2 C7 123.2(5) . . ?

C4 C3 C2 122.9(5) . . ?

C4 C3 I1 116.3(4) . . ?

C2 C3 I1 120.8(4) . . ?

C3 C4 C5 119.4(5) . . ?

C3 C4 H4 120.3 . . ?

C5 C4 H4 120.3 . . ?

C6 C5 C4 121.7(6) . . ?

C6 C5 I2 120.0(4) . . ?

C4 C5 I2 118.3(4) . . ?

C5 C6 C1 117.2(5) . . ?

C5 C6 C8 122.1(6) . . ?

C1 C6 C8 120.7(5) . . ?

C2 C7 H7A 109.5 . . ?

C2 C7 H7B 109.5 . . ?

H7A C7 H7B 109.5 . . ?

C2 C7 H7C 109.5 . . ?

H7A C7 H7C 109.5 . . ?

H7B C7 H7C 109.5 . . ?

C6 C8 H8A 109.5 . . ?

C6 C8 H8B 109.5 . . ?

H8A C8 H8B 109.5 . . ?

C6 C8 H8C 109.5 . . ?

H8A C8 H8C 109.5 . . ?

H8B C8 H8C 109.5 . . ?

C10 C9 C14 121.3(5) . . ?

C10 C9 C1 119.3(5) . . ?

C14 C9 C1 119.2(5) . . ?

C11 C10 C9 117.6(6) . . ?

C11 C10 C15 121.5(6) . . ?

C9 C10 C15 120.9(5) . . ?

C12 C11 C10 122.0(6) . . ?

C12 C11 I3 117.5(4) . . ?

C10 C11 I3 120.5(5) . . ?

C11 C12 C13 119.5(6) . . ?

C11 C12 H12 120.2 . . ?

C13 C12 H12 120.2 . . ?

C12 C13 C14 122.1(6) . . ?

C12 C13 I4 116.9(5) . . ?

C14 C13 I4 120.9(5) . . ?

C13 C14 C9 117.5(6) . . ?

C13 C14 C16 122.6(6) . . ?

C9 C14 C16 119.9(6) . . ?

C10 C15 H15A 109.5 . . ?

C10 C15 H15B 109.5 . . ?

H15A C15 H15B 109.5 . . ?

C10 C15 H15C 109.5 . . ?

H15A C15 H15C 109.5 . . ?

H15B C15 H15C 109.5 . . ?

C14 C16 H16A 109.5 . . ?

C14 C16 H16B 109.5 . . ?

H16A C16 H16B 109.5 . . ?

C14 C16 H16C 109.5 . . ?

H16A C16 H16C 109.5 . . ?

H16B C16 H16C 109.5 . . ?

_refine_diff_density_max 1.600

_refine_diff_density_min -1.965

_refine_diff_density_rms 0.158

_shelx_res_file

;

TITL YJ01423_refine4

YJ01423_refine6.res

created by SHELXL-2016/6 at 09:29:26 on 27-Jul-2018

REM Same as YJ01423_refine4 but WGHT is value suggested in refine4.res

REM See details in YJ014status.txt

CELL 0.71073 9.0436 12.1873 17.4292 90.003 98.137 89.913

ZERR 4.000 0.0005 0.0006 0.0009 0.002 0.002 0.002

LATT 1

SYMM -X, 1/2+Y, 1/2-Z

SFAC C H I

UNIT 64 56 16

shel 99 0.8

L.S. 50

BOND $H

LIST 4

FMAP 2

PLAN 20

RTAB Dih1 C6 C1 C9 C14

RTAB Dih2 C2 C1 C9 C14

RTAB DiM1 C8 C6 C1 C9

RTAB DiM2 C7 C2 C1 C9

RTAB DiM3 C16 C14 C9 C1

RTAB DiM4 C15 C10 C9 C1

ACTA

WGHT 0.075000 6.962300

FVAR 0.21201

I1 3 0.823439 0.581025 0.050662 11.00000 0.09415 0.05244 =

0.03313 0.00595 0.02678 0.01585

I2 3 0.932172 0.864228 0.328522 11.00000 0.11208 0.06684 =

0.06487 -0.01969 0.03132 -0.05073

I3 3 0.635610 0.197522 0.458143 11.00000 0.09134 0.05328 =

0.06134 0.02545 0.00505 -0.00697

I4 3 0.147239 0.521856 0.407534 11.00000 0.05857 0.09304 =

0.09828 -0.00950 0.02549 -0.00607

C1 1 0.666955 0.568300 0.276177 11.00000 0.05730 0.03042 =

0.03441 0.00167 0.01505 -0.00528

C2 1 0.682815 0.543721 0.199115 11.00000 0.06224 0.02784 =

0.02940 0.00112 0.01056 -0.00198

C3 1 0.778458 0.612564 0.163825 11.00000 0.06504 0.03478 =

0.02396 0.00377 0.01486 0.00368

C4 1 0.846580 0.701115 0.199683 11.00000 0.06150 0.04597 =

0.03794 0.00865 0.01907 -0.00967

AFIX 43

H4 2 0.907238 0.745538 0.173997 11.00000 -1.20000

AFIX 0

C5 1 0.825264 0.725015 0.274834 11.00000 0.06951 0.03687 =

0.03690 -0.00413 0.01339 -0.01559

C6 1 0.738528 0.657769 0.315910 11.00000 0.06969 0.04175 =

0.03166 -0.00328 0.01879 -0.01620

C7 1 0.601870 0.448593 0.158634 11.00000 0.08899 0.04067 =

0.04069 -0.00685 0.01324 -0.01276

AFIX 137

H7A 2 0.670301 0.388803 0.156247 11.00000 -1.50000

H7B 2 0.560306 0.469897 0.107032 11.00000 -1.50000

H7C 2 0.523012 0.425979 0.186622 11.00000 -1.50000

AFIX 0

C8 1 0.719169 0.680740 0.398093 11.00000 0.10509 0.06734 =

0.03559 -0.01691 0.02672 -0.03530

AFIX 137

H8A 2 0.814336 0.697938 0.427397 11.00000 -1.50000

H8B 2 0.678221 0.617248 0.419932 11.00000 -1.50000

H8C 2 0.652510 0.741798 0.399765 11.00000 -1.50000

AFIX 0

C9 1 0.573612 0.496574 0.320039 11.00000 0.05822 0.03182 =

0.03055 -0.00161 0.01452 -0.01262

C10 1 0.635772 0.400123 0.354940 11.00000 0.05673 0.03286 =

0.03223 -0.00078 0.00778 -0.00821

C11 1 0.550429 0.341179 0.401248 11.00000 0.05890 0.03956 =

0.03420 0.00256 0.00854 -0.01021

C12 1 0.411269 0.375270 0.413302 11.00000 0.06408 0.04483 =

0.03825 0.00432 0.01457 -0.01650

AFIX 43

H12 2 0.356962 0.334664 0.444815 11.00000 -1.20000

AFIX 0

C13 1 0.352841 0.469150 0.378794 11.00000 0.05179 0.05330 =

0.04386 -0.00436 0.01638 -0.00778

C14 1 0.429082 0.531259 0.330028 11.00000 0.05850 0.03592 =

0.04166 0.00046 0.01025 -0.00447

C15 1 0.789330 0.364733 0.344718 11.00000 0.05707 0.05448 =

0.06023 0.00736 0.01453 -0.00680

AFIX 137

H15A 2 0.854875 0.374032 0.392629 11.00000 -1.50000

H15B 2 0.824263 0.408455 0.305149 11.00000 -1.50000

H15C 2 0.787913 0.288879 0.329781 11.00000 -1.50000

AFIX 0

C16 1 0.362574 0.633959 0.288609 11.00000 0.08073 0.04710 =

0.08643 0.01342 0.02582 0.01083

AFIX 137

H16A 2 0.272615 0.615043 0.255135 11.00000 -1.50000

H16B 2 0.433140 0.664789 0.258397 11.00000 -1.50000

H16C 2 0.340088 0.686712 0.326215 11.00000 -1.50000

AFIX 0

HKLF 4

REM YJ01423_refine4

REM R1 = 0.0403 for 3401 Fo > 4sig(Fo) and 0.0456 for all 3880 data

REM 185 parameters refined using 0 restraints

END

WGHT 0.0576 7.8325

REM Highest difference peak 1.600, deepest hole -1.965, 1-sigma level 0.158

Q1 1 0.7209 0.2247 0.4754 11.00000 0.05 1.60

Q2 1 0.7467 0.5986 0.0507 11.00000 0.05 1.37

Q3 1 0.5727 0.2448 0.4708 11.00000 0.05 1.36

Q4 1 0.8811 0.6235 0.0621 11.00000 0.05 1.21

Q5 1 0.8702 0.5412 0.0703 11.00000 0.05 1.17

Q6 1 0.8448 0.8788 0.3289 11.00000 0.05 0.93

Q7 1 0.1259 0.5539 0.3561 11.00000 0.05 0.81

Q8 1 0.9788 0.8150 0.3074 11.00000 0.05 0.77

Q9 1 0.7355 0.5213 0.0329 11.00000 0.05 0.72

Q10 1 0.2630 0.5105 0.3854 11.00000 0.05 0.62

Q11 1 0.9565 0.9030 0.3054 11.00000 0.05 0.59

Q12 1 1.0176 0.8754 0.3600 11.00000 0.05 0.58

Q13 1 0.1428 0.4884 0.3581 11.00000 0.05 0.50

Q14 1 0.2599 0.5316 0.4245 11.00000 0.05 0.50

Q15 1 0.9331 0.5829 0.0297 11.00000 0.05 0.47

Q16 1 0.6191 0.4391 0.3494 11.00000 0.05 0.46

Q17 1 0.7134 0.4185 0.3697 11.00000 0.05 0.46

Q18 1 0.4992 0.5066 0.3133 11.00000 0.05 0.44

Q19 1 0.9715 0.6776 0.0952 11.00000 0.05 0.42

Q20 1 0.4923 0.3799 0.4251 11.00000 0.05 0.42

;

_shelx_res_checksum 51798

_shelx_hkl_file

;

-1 0 0 508.111 16.1906 5

-2 0 0 982.971 30.5925 4

-2 0 0 942.918 30.5185 5

-2 0 0 981.303 30.4981 1

2 0 0 906.102 30.4966 5

2 0 0 1000.81 30.7020 3

2 0 0 1019.83 30.7321 4

2 0 0 960.437 30.6126 1

-3 0 0 410.901 12.9215 1

-3 0 0 395.697 12.9078 5

3 0 0 422.941 13.0484 4

3 0 0 412.140 12.9558 1

3 0 0 409.845 12.9413 3

3 0 0 408.311 12.9250 5

-4 0 0 3753.70 118.284 1

-4 0 0 3765.14 118.465 5

4 0 0 3771.91 118.636 1

4 0 0 3771.78 118.713 3

4 0 0 3839.32 118.750 4

4 0 0 3803.64 118.364 5

-5 0 0 302.014 9.55022 5

-5 0 0 285.987 9.55882 1

5 0 0 294.501 9.67424 1

5 0 0 300.913 9.81651 4

5 0 0 278.552 9.75595 3

5 0 0 315.859 9.59838 5

6 0 0 395.209 13.0801 1

6 0 0 383.657 13.0441 3

6 0 0 436.640 13.2070 5

-7 0 0 -0.6781 0.85399 5

7 0 0 -0.4277 0.69709 3

7 0 0 0.39234 0.85160 1

-8 0 0 -0.4032 1.03351 5

8 0 0 0.68027 0.74490 3

8 0 0 0.18280 0.96128 1

-9 0 0 69.6960 3.13594 5

9 0 0 59.8498 3.15879 3

9 0 0 70.9025 3.42178 1

-10 0 0 73.6925 3.57817 5

10 0 0 74.1643 3.80164 1

10 0 0 70.2380 3.77327 3

11 0 0 -0.3226 1.61920 3

11 0 0 0.40580 1.47656 1

13 0 0 3.63060 2.25688 1

13 0 0 2.48272 2.73147 3

14 0 0 0.61981 3.20459 3

13 -1 0 2.52483 2.91276 3

13 -1 0 1.33739 2.26755 1

-12 1 0 1.28663 1.89874 1

11 -1 0 -0.4912 1.56936 3

11 -1 0 -0.0103 1.49160 1

10 -1 0 1.73453 1.41075 1

10 -1 0 0.17506 1.20171 3

9 -1 0 48.7957 3.02345 1

9 -1 0 44.8403 2.83980 3

-9 1 0 49.2328 2.64452 5

8 -1 0 13.8774 1.76175 3

8 -1 0 17.2285 2.00868 1

-8 1 0 14.0616 1.65060 5

7 -1 0 65.0893 2.88461 3

7 -1 0 64.8990 3.06216 1

-7 1 0 66.7833 2.72122 5

6 -1 0 16.0963 1.43787 5

6 -1 0 16.3944 1.44691 1

6 -1 0 12.8550 1.42554 3

-6 1 0 14.5573 1.41218 5

5 -1 0 413.524 14.2735 3

5 -1 0 487.121 14.0926 5

5 -1 0 440.301 14.3377 4

-5 1 0 445.380 14.0228 5

-5 1 0 424.441 14.1234 1

4 -1 0 13.6671 1.02028 5

4 -1 0 17.2333 1.31013 4

4 -1 0 13.9214 1.13203 1

-4 1 0 16.5123 1.11440 1

-4 1 0 11.4457 0.94608 5

3 -1 0 52.2517 1.91878 3

3 -1 0 50.2806 2.06847 4

3 -1 0 48.0931 1.98558 1

3 -1 0 55.0655 1.86820 5

-3 1 0 50.5845 1.96627 1

-3 1 0 53.9628 1.93899 3

-3 1 0 50.0047 2.11965 4

-3 1 0 50.1966 1.82685 5

2 -1 0 5312.92 164.190 4

2 -1 0 5137.33 163.924 5

-2 1 0 5150.20 164.006 1

-2 1 0 5214.60 164.280 4

-2 1 0 5456.35 163.927 3

1 -1 0 92.0487 2.96734 3

1 -1 0 91.8702 3.01325 4

1 -1 0 91.2867 2.99345 1

-1 1 0 89.0246 3.01300 4

-1 1 0 97.2810 2.99534 1

-1 1 0 95.3452 2.96838 3

-1 -1 0 93.8874 3.00001 2

-1 -1 0 95.6970 3.01274 5

1 1 0 96.7098 3.00223 2

1 1 0 90.6550 2.97177 1

2 1 0 4999.42 155.868 5

-3 -1 0 48.4640 1.75961 1

-3 -1 0 47.3138 1.83993 5

3 1 0 52.0703 1.97255 4

3 1 0 48.3299 1.85935 5

3 1 0 49.7098 1.77748 1

-4 -1 0 15.8526 1.02018 5

-4 -1 0 12.1403 0.91241 1

4 1 0 14.3636 1.21722 4

4 1 0 15.1855 1.03615 5

4 1 0 12.5468 0.96973 1

-5 -1 0 414.712 13.1239 5

-5 -1 0 402.382 13.0605 1

5 1 0 407.099 13.1492 5

5 1 0 412.925 13.3313 4

5 1 0 417.749 13.1314 1

-6 -1 0 14.2873 1.41897 5

6 1 0 13.6028 1.45265 1

6 1 0 14.8543 1.41234 3

-7 -1 0 72.7918 3.08861 5

7 1 0 72.8003 3.16366 3

7 1 0 76.9624 3.20556 1

-8 -1 0 27.7156 1.98487 5

8 1 0 25.6097 2.00872 3

8 1 0 26.0315 2.12724 1

-9 -1 0 34.7455 2.42966 5

9 1 0 34.3579 2.50959 3

9 1 0 36.6286 2.58018 1

-10 -1 0 0.16358 1.40105 5

10 1 0 0.15451 1.00146 3

10 1 0 0.35652 1.34442 1

-11 -1 0 1.00715 1.77884 5

11 1 0 0.81657 1.40351 3

11 1 0 2.14064 1.63190 1

12 1 0 1.44417 3.64694 3

12 1 0 0.64725 1.92685 1

13 1 0 2.36913 2.26509 1

13 1 0 -0.1522 2.37015 3

13 -2 0 -2.0870 2.45482 3

13 -2 0 3.21718 2.35117 1

12 -2 0 2.41350 1.86124 1

-12 2 0 3.64855 2.04358 1

11 -2 0 6.59792 1.91617 3

11 -2 0 8.41016 1.81581 1

10 -2 0 4.99814 1.54106 3

10 -2 0 5.83931 1.42193 1

9 -2 0 1.75676 1.14807 3

9 -2 0 3.30061 1.27937 1

8 -2 0 5.29856 1.25562 1

8 -2 0 3.66519 0.96686 3

7 -2 0 299.186 10.1642 3

7 -2 0 306.402 10.2644 1

-7 2 0 314.945 10.0022 5

6 -2 0 140.673 5.05097 3

6 -2 0 150.235 4.94772 5

6 -2 0 140.299 5.15719 1

-6 2 0 140.904 4.87046 5

5 -2 0 2.33509 0.73413 5

5 -2 0 1.92475 0.78117 1

5 -2 0 0.71524 1.24355 4

-5 2 0 1.38127 0.73611 1

-5 2 0 1.40830 0.61374 5

4 -2 0 143.893 5.11164 4

4 -2 0 143.747 4.92367 3

4 -2 0 155.891 4.83229 5

4 -2 0 142.557 5.01593 1

-4 2 0 145.208 4.79346 5

-4 2 0 140.635 5.17889 4

-4 2 0 151.418 4.94127 3

-4 2 0 150.881 5.00449 1

3 -2 0 2607.49 86.7768 1

3 -2 0 2756.02 86.8958 4

3 -2 0 2701.64 86.7400 3

3 -2 0 2886.43 86.3873 5

-3 2 0 2820.38 86.5750 3

-3 2 0 2735.64 86.9729 4

-3 2 0 2818.27 86.4918 1

2 -2 0 197.240 6.69754 1

2 -2 0 203.801 6.74873 4

2 -2 0 197.369 6.64529 3

-2 2 0 203.939 6.77340 4

-2 2 0 222.022 6.65338 3

-2 2 0 225.194 6.71345 1

1 -2 0 830.755 23.8375 1

1 -2 0 755.721 23.5452 3

1 -2 0 660.644 23.6003 4

-1 2 0 743.980 23.5395 4

0 -2 0 833.249 25.6049 2

0 -2 0 808.631 25.5503 5

0 -2 0 789.602 25.6971 3

0 2 0 805.455 25.6590 3

0 2 0 807.848 25.5610 2

0 2 0 846.184 25.6435 1

-1 -2 0 756.302 22.8033 1

-1 -2 0 726.517 22.8777 2

-1 -2 0 717.826 22.8651 5

1 2 0 748.064 23.0422 5

1 2 0 701.822 22.7817 1

1 2 0 723.692 22.7968 2

-2 -2 0 276.767 9.17616 5

-2 -2 0 299.976 9.11216 1

-2 -2 0 286.823 9.15907 2

2 2 0 304.500 9.19900 4

2 2 0 273.511 9.09608 1

2 2 0 297.522 9.16146 2

2 2 0 291.946 9.19305 5

-3 -2 0 2777.66 93.2293 5

-3 -2 0 3003.29 92.7850 1

3 2 0 3164.40 93.3417 4

3 2 0 2893.16 92.8598 1

3 2 0 2863.69 93.0248 5

3 2 0 3141.51 93.0624 2

-4 -2 0 163.666 5.58792 5

-4 -2 0 168.795 5.48793 1

4 2 0 164.281 5.48060 1

4 2 0 175.126 5.71139 4

4 2 0 178.469 5.57986 2

4 2 0 163.716 5.61516 5

-5 -2 0 1.30476 0.60281 1

-5 -2 0 2.39943 1.49024 5

5 2 0 1.25098 1.15030 4

5 2 0 2.26481 0.73236 2

5 2 0 3.09394 0.61360 1

5 2 0 1.69339 0.86429 5

-6 -2 0 162.350 5.55806 5

6 2 0 155.671 5.54362 3

6 2 0 164.067 5.52199 1

-7 -2 0 315.087 10.1618 5

7 2 0 293.381 10.1637 3

7 2 0 314.765 10.1566 1

-8 -2 0 3.30401 1.19930 5

8 2 0 3.59828 1.12839 1

8 2 0 3.33135 0.91075 3

-9 -2 0 3.57870 1.35020 5

9 2 0 2.58540 1.03250 3

9 2 0 2.28068 1.23565 1

-10 -2 0 9.84872 1.77365 5

10 2 0 9.10276 1.62440 1

-11 -2 0 12.5473 2.08237 5

11 2 0 7.54523 1.46403 3

11 2 0 9.50808 1.90918 1

-12 -2 0 1.55991 2.31576 5

12 2 0 0.21516 1.82076 1

12 2 0 2.20840 2.00918 3

13 2 0 0.55749 2.53860 3

13 2 0 0.32132 2.26527 1

13 -3 0 -0.7611 2.30319 1

13 -3 0 -1.7925 2.77479 3

12 -3 0 4.00158 1.96880 1

12 -3 0 -0.6691 3.62601 3

-12 3 0 1.83284 2.23741 1

11 -3 0 -0.7538 1.83533 3

11 -3 0 0.14660 1.46633 1

10 -3 0 13.0104 1.93881 3

10 -3 0 7.97193 1.64300 1

9 -3 0 19.2844 2.22114 1

9 -3 0 19.0483 2.50069 3

8 -3 0 24.5482 2.20959 1

8 -3 0 23.2249 2.21458 3

7 -3 0 16.9160 1.85251 3

7 -3 0 20.3109 1.90786 1

6 -3 0 235.174 7.39791 5

6 -3 0 221.517 7.67682 1

6 -3 0 215.006 7.55310 3

5 -3 0 17.9966 1.53708 3

5 -3 0 16.2647 1.14206 5

-5 3 0 15.5197 1.26903 3

4 -3 0 11.1649 0.90335 5

4 -3 0 12.4390 1.24207 1

4 -3 0 9.99535 1.53855 4

-4 3 0 7.82868 1.63025 4

-4 3 0 12.3332 1.06110 3

-4 3 0 14.0511 1.26211 1

3 -3 0 70.5398 2.26017 5

3 -3 0 62.4300 2.51509 1

3 -3 0 69.5356 2.61784 4

3 -3 0 64.2309 2.43972 3

-3 3 0 67.3399 2.47290 1

-3 3 0 62.8175 2.64167 4

-3 3 0 69.5202 2.39693 3

2 -3 0 52.7854 2.07432 1

2 -3 0 54.5141 2.14383 4

2 -3 0 53.9768 2.06208 3

-2 3 0 57.6996 2.03633 1

-2 3 0 56.7628 1.98166 3

-2 3 0 54.1310 2.16971 4

1 -3 0 1833.83 58.7595 6

1 -3 0 1886.07 59.1751 3

1 -3 0 1754.89 59.0021 4

1 -3 0 1958.37 59.3159 1

-1 3 0 2060.93 59.1984 1

-1 3 0 1916.42 59.0374 3

-1 3 0 1775.38 59.0625 4

0 -3 0 2.38752 0.33623 2

0 -3 0 3.75986 0.51038 3

0 -3 0 3.26803 0.47677 5

0 3 0 3.43363 0.45865 2

0 3 0 3.55730 0.43304 1

0 3 0 2.85968 0.34123 1

0 3 0 2.03028 0.32184 3

-1 -3 0 2189.29 65.7484 1

-1 -3 0 2067.94 66.0356 2

-1 -3 0 2029.14 66.0424 5

1 3 0 2088.92 65.8021 1

1 3 0 2131.50 66.0089 5

1 3 0 2144.21 65.9687 2

-2 -3 0 96.3391 3.13695 1

-2 -3 0 92.2140 3.21686 2

-2 -3 0 95.2158 3.24375 5

2 3 0 95.4719 3.16519 1

2 3 0 99.7925 3.25783 5

2 3 0 98.1564 3.21957 2

-3 -3 0 67.8529 2.31636 1

-3 -3 0 64.7606 2.44224 5

3 3 0 67.6454 2.38613 2

3 3 0 66.8774 2.44517 5

3 3 0 71.9871 2.44726 4

3 3 0 62.7960 2.27225 1

4 3 0 3.28144 0.61600 2

-5 -3 0 25.7371 1.44507 1

5 3 0 25.6183 1.34168 1

5 3 0 25.7274 1.79372 4

-6 -3 0 211.971 7.07316 5

6 3 0 210.252 7.50597 4

6 3 0 201.990 6.88814 1

-7 -3 0 21.1446 1.82922 5

7 3 0 15.8622 1.75615 1

7 3 0 15.6632 1.66942 3

-8 -3 0 14.2072 2.12406 5

8 3 0 14.6636 1.91824 3

8 3 0 18.5894 2.00183 1

-9 -3 0 19.6710 2.20206 5

9 3 0 16.1029 2.24810 1

9 3 0 17.2750 2.08589 3

-10 -3 0 19.1606 2.74099 5

10 3 0 19.9540 2.68489 1

-11 -3 0 1.46845 1.77046 5

11 3 0 -0.1406 1.22122 3

11 3 0 -3.3492 1.57103 1

-12 -3 0 -0.3680 2.79804 5

12 3 0 0.60679 1.44067 3

12 3 0 2.77444 1.89667 1

-13 -3 0 2.98297 2.98424 5

13 3 0 1.68653 2.63135 3

13 3 0 -1.2499 2.29525 1

13 -4 0 -2.1009 2.60703 3

13 -4 0 2.39504 2.42088 1

-13 4 0 4.11716 3.11300 1

12 -4 0 -0.3520 1.98989 1

12 -4 0 -1.1229 2.27173 3

-12 4 0 2.71139 2.44236 1

11 -4 0 2.41625 1.89471 3

11 -4 0 2.06899 1.70634 1

10 -4 0 5.96297 1.52877 1

10 -4 0 8.81780 1.70941 3

9 -4 0 -0.5178 1.19265 1

9 -4 0 -1.0445 1.23463 3

8 -4 0 55.2083 3.07126 3

8 -4 0 59.2827 3.13016 1

7 -4 0 212.215 7.31930 1

7 -4 0 200.264 7.16434 3

6 -4 0 174.287 6.31421 1

6 -4 0 175.452 6.19813 3

6 -4 0 183.387 5.94148 5

5 -4 0 8.10580 1.09479 1

5 -4 0 9.50576 1.01709 5

-5 4 0 12.3484 1.20408 3

4 -4 0 194.732 6.93887 1

4 -4 0 199.460 6.84818 3

4 -4 0 213.471 6.61787 5

4 -4 0 201.855 7.04727 4

-4 4 0 213.671 6.79801 3

-4 4 0 216.481 6.90415 1

-4 4 0 201.018 7.13361 4

3 -4 0 81.2857 3.10643 1

3 -4 0 82.0766 3.06874 3

3 -4 0 83.7561 3.22281 4

-3 4 0 79.2310 3.26075 4

-3 4 0 86.6293 2.95951 3

-3 4 0 91.1349 3.06511 1

2 -4 0 115.429 3.99668 4

2 -4 0 111.843 3.93431 3

2 -4 0 116.917 3.97064 1

-2 4 0 121.991 3.90292 1

-2 4 0 118.841 3.86241 3

-2 4 0 110.444 4.00846 4

1 -4 0 584.984 18.5481 3

1 -4 0 576.744 18.5411 4

1 -4 0 606.864 18.5655 1

-1 4 0 564.911 18.5399 4

-1 4 0 606.459 18.5158 1

-1 4 0 603.420 18.5293 3

0 -4 0 399.645 13.0416 5

0 -4 0 397.228 13.0669 2

0 -4 0 394.219 13.0967 3

0 4 0 420.910 13.1093 3

0 4 0 433.983 13.0747 2

0 4 0 448.011 13.0741 1

0 4 0 416.726 13.0736 1

-1 -4 0 607.956 19.4188 2

-1 -4 0 591.629 19.4112 5

-1 -4 0 646.536 19.4212 1

1 4 0 618.071 19.4071 2

1 4 0 641.555 19.4589 6

1 4 0 609.960 19.3858 1

1 4 0 625.295 19.4204 5

-2 -4 0 112.816 3.79007 2

-2 -4 0 116.721 3.67332 1

-2 -4 0 110.431 3.80787 5

2 4 0 114.899 3.77710 2

2 4 0 116.331 3.80980 5

2 4 0 110.156 3.73850 1

-3 -4 0 93.0295 3.14670 1

-3 -4 0 91.1927 3.31705 5

-3 -4 0 92.9282 3.27844 2

3 4 0 94.3261 3.17707 1

3 4 0 100.537 3.28157 2

3 4 0 95.3798 3.32953 5

-4 -4 0 185.599 5.88829 1

-4 -4 0 157.097 6.21004 5

4 4 0 192.185 6.04074 2

4 4 0 188.530 6.09514 4

4 4 0 182.439 5.86382 1

4 4 0 181.758 6.11939 5

-5 -4 0 19.3869 1.40497 1

-5 -4 0 16.5494 1.60393 5

5 4 0 19.6107 1.21245 1

5 4 0 14.3870 1.75614 4

6 4 0 193.289 6.92084 4

6 4 0 199.518 6.50650 1

-7 -4 0 242.102 8.15805 5

7 4 0 236.372 7.94698 1

-8 -4 0 57.7581 2.96720 5

8 4 0 58.0731 2.83151 1

8 4 0 48.1276 2.73084 3

-9 -4 0 -1.7312 1.31172 5

9 4 0 0.46396 1.20593 1

9 4 0 0.47315 0.96290 3

-10 -4 0 12.2311 1.98686 5

10 4 0 7.14893 1.39325 3

10 4 0 6.07142 1.57324 1

-11 -4 0 3.73322 2.04613 5

11 4 0 2.49818 1.41942 3

11 4 0 6.90708 1.87008 1

-12 -4 0 -1.3294 2.42126 5

12 4 0 0.61619 2.14147 1

12 4 0 -0.3189 1.45068 3

-13 -4 0 0.06957 3.05453 5

13 4 0 1.47147 1.76553 3

13 4 0 5.70179 2.38707 1

13 -5 0 -3.5988 3.07427 3

13 -5 0 1.58505 2.51557 1

-13 5 0 8.45115 3.08627 1

12 -5 0 4.10484 2.48522 3

12 -5 0 6.20353 2.22472 1

-12 5 0 5.45847 2.76123 1

11 -5 0 2.03331 1.86405 1

11 -5 0 4.28592 2.15210 3

-11 5 0 4.49622 2.33971 1

10 -5 0 0.04875 1.67692 3

10 -5 0 0.71336 1.50155 1

9 -5 0 9.40158 1.73768 1

8 -5 0 38.2719 2.64177 1

8 -5 0 39.0867 2.87935 3

7 -5 0 19.4386 1.64722 5

7 -5 0 24.6448 2.43952 3

7 -5 0 25.7113 2.20404 1

6 -5 0 14.0337 1.76184 1

6 -5 0 15.4026 1.88017 3

6 -5 0 14.3825 1.25438 5

5 -5 0 127.887 5.01654 1

5 -5 0 141.080 4.52056 5

5 -5 0 135.391 4.93897 3

4 -5 0 238.004 7.97156 4

4 -5 0 225.583 7.79027 3

4 -5 0 219.003 8.08749 1

-4 5 0 245.470 7.69959 3

-4 5 0 240.786 7.80269 1

-4 5 0 238.318 8.11322 4

3 -5 0 698.223 21.6343 4

3 -5 0 653.398 21.5963 1

3 -5 0 667.526 21.5224 3

-3 5 0 700.933 21.5132 1

-3 5 0 678.714 21.6700 4

-3 5 0 688.650 21.4262 3

2 -5 0 401.076 12.5759 4

2 -5 0 386.501 12.5373 3

2 -5 0 392.848 12.5772 1

2 -5 0 371.448 12.3506 6

-2 5 0 402.509 12.4619 3

-2 5 0 383.614 12.5926 4

-2 5 0 411.330 12.4916 1

1 -5 0 2.09672 0.52001 3

1 -5 0 1.54100 0.48693 2

-1 5 0 2.77273 0.49959 3

-1 5 0 0.94171 0.77532 4

-1 5 0 2.59345 0.56647 1

-1 5 0 3.22997 0.48479 1

0 -5 0 2.85700 0.44865 3

0 -5 0 3.91319 0.54139 2

0 -5 0 2.95002 0.50252 5

0 5 0 3.90531 0.51365 1

0 5 0 2.94534 0.52922 1

0 5 0 3.36483 0.52909 3

-1 -5 0 2.23159 0.48708 2

1 5 0 0.94425 0.53662 1

1 5 0 1.05430 0.41920 3

1 5 0 3.79992 0.55631 2

1 5 0 2.35234 0.52797 5

1 5 0 2.17224 0.44280 6

-2 -5 0 416.587 12.9720 1

-2 -5 0 394.961 13.1147 5

-2 -5 0 403.119 13.1072 2

2 5 0 425.547 13.0929 6

2 5 0 429.892 13.1384 5

2 5 0 420.965 13.1013 2

2 5 0 406.843 13.0581 1

-3 -5 0 747.085 24.5866 5

-3 -5 0 774.836 24.5679 2

-3 -5 0 801.883 24.4018 1

3 5 0 786.820 24.6039 5

3 5 0 804.433 24.5651 2

3 5 0 777.741 24.4646 1

-4 -5 0 274.539 9.11812 1

4 5 0 302.773 9.34308 4

4 5 0 272.720 9.13889 1

4 5 0 299.378 9.44891 2

4 5 0 286.989 9.50646 5

-5 -5 0 183.777 6.41672 5

-5 -5 0 187.689 6.16876 1

5 5 0 191.060 6.50460 4

-6 -5 0 11.8515 1.72239 5

-6 -5 0 18.9208 1.55470 1

6 5 0 19.3090 1.39792 1

6 5 0 16.0847 2.14178 4

-7 -5 0 30.3174 2.44220 5

7 5 0 36.1402 2.01418 1

-8 -5 0 44.7458 2.80541 5

8 5 0 46.1080 2.61106 1

-9 -5 0 11.5805 1.85049 5

9 5 0 9.41178 1.50761 1

9 5 0 6.85242 1.19079 3

-10 -5 0 1.65256 1.82297 5

10 5 0 1.78699 1.47956 1

10 5 0 0.75721 1.24313 3

-11 -5 0 4.40352 2.22153 5

11 5 0 2.97663 1.83151 1

11 5 0 1.55705 1.43801 3

-12 -5 0 11.2595 2.76537 5

12 5 0 3.65456 1.72212 3

12 5 0 5.15960 2.28576 1

-13 -5 0 4.60810 3.35185 5

13 5 0 2.29890 2.41269 1

13 5 0 2.22802 1.98786 3

13 -6 0 -4.6302 3.19684 3

13 -6 0 0.56750 2.80524 1

-13 6 0 1.84811 4.90973 1

12 -6 0 -1.6496 2.56506 3

12 -6 0 0.25328 2.27821 1

-12 6 0 0.30114 2.95578 1

11 -6 0 4.70826 2.18234 1

11 -6 0 0.76386 2.21964 3

-11 6 0 8.62993 2.60828 1

10 -6 0 28.9617 3.96372 3

10 -6 0 28.6966 3.36750 1

-10 6 0 38.5421 4.31086 1

9 -6 0 -0.3889 1.31465 3

9 -6 0 0.68646 1.44105 1

8 -6 0 17.4376 2.75728 3

8 -6 0 16.6521 2.53417 1

7 -6 0 5.05470 1.00736 5

7 -6 0 3.89301 1.17142 3

7 -6 0 7.24388 1.36999 1

6 -6 0 21.8430 2.25549 3

6 -6 0 16.1727 2.18449 1

6 -6 0 18.3108 1.34173 5

5 -6 0 34.9920 2.41866 3

5 -6 0 31.5952 2.33394 1

5 -6 0 34.7377 1.54110 5

4 -6 0 0.50670 0.96948 1

4 -6 0 6.38316 1.51446 4

4 -6 0 3.89480 1.03948 3

-4 6 0 5.98416 1.58999 4

-4 6 0 2.72092 0.89006 1

-4 6 0 2.86467 0.59743 3

3 -6 0 396.428 12.5586 4

3 -6 0 365.846 12.5385 1

3 -6 0 377.924 12.2793 6

3 -6 0 385.214 12.4956 3

-3 6 0 394.188 12.3454 3

-3 6 0 397.633 12.4241 1

-3 6 0 389.581 12.6165 4

2 -6 0 359.050 11.7931 3

2 -6 0 367.765 11.8498 1

2 -6 0 367.508 11.5701 6

2 -6 0 368.555 11.8054 4

-2 6 0 369.651 11.7011 3

-2 6 0 372.483 11.7148 1

-2 6 0 362.742 11.8411 4

-2 6 0 364.076 11.8290 1

1 -6 0 10.7949 1.16493 3

1 -6 0 13.1657 1.08972 2

-1 6 0 13.2623 1.19056 1

-1 6 0 14.1788 0.95442 1

-1 6 0 14.9734 1.19423 3

0 -6 0 130.330 4.42399 2

0 -6 0 128.216 4.36496 5

0 6 0 135.401 4.50118 3

0 6 0 128.347 4.45729 1

0 6 0 136.312 4.38181 1

-1 -6 0 9.80611 1.05900 2

-1 -6 0 10.6977 1.00622 5

1 6 0 15.0242 1.18984 1

1 6 0 11.8195 0.98292 2

1 6 0 10.5564 0.85135 6

1 6 0 11.8404 1.04323 3

-2 -6 0 370.002 12.2260 5

-2 -6 0 380.904 12.2340 2

-2 -6 0 383.602 12.0626 1

2 6 0 369.616 12.1781 1

2 6 0 395.700 12.2493 5

2 6 0 394.836 12.1700 6

2 6 0 391.277 12.2108 2

-3 -6 0 423.983 13.8223 2

3 6 0 416.459 13.7885 2

3 6 0 434.696 13.7304 1

3 6 0 447.101 13.8283 6

3 6 0 446.381 13.8638 5

-4 -6 0 3.61422 0.92975 2

-4 -6 0 5.06452 1.04171 5

4 6 0 2.97648 0.73796 1

4 6 0 4.79424 0.94503 6

-5 -6 0 33.6098 2.01158 5

5 6 0 33.4279 2.15778 4

5 6 0 33.3926 1.71431 1

-6 -6 0 10.3963 1.45746 5

6 6 0 13.5273 1.37286 1

6 6 0 13.7713 2.02947 4

-7 -6 0 5.66683 1.34386 5

7 6 0 6.01953 0.94898 1

-8 -6 0 11.9996 1.87937 5

8 6 0 15.7609 1.99196 1

-9 -6 0 1.73478 1.63732 5

9 6 0 -0.7163 1.02574 3

9 6 0 0.47037 1.32255 1

-10 -6 0 33.2803 3.46805 5

10 6 0 30.4895 2.93217 3

10 6 0 37.7546 3.04988 1

-11 -6 0 5.61965 2.32342 5

11 6 0 3.56988 1.56080 3

11 6 0 4.03986 1.86116 1

-12 -6 0 0.91799 2.97519 5

12 6 0 1.43954 1.82112 3

12 6 0 -2.8406 3.24240 1

-13 -6 0 -0.8545 3.36180 5

13 6 0 -2.2471 2.32744 1

13 6 0 2.19163 2.22785 3

13 -7 0 2.81342 3.20419 6

13 -7 0 -4.9339 3.56801 3

-13 7 0 2.22238 3.59379 1

12 -7 0 0.03655 2.78791 3

12 -7 0 2.61812 2.63329 1

-12 7 0 1.87477 2.99099 1

11 -7 0 4.33481 2.38129 1

11 -7 0 4.31574 2.33664 3

-11 7 0 1.24815 2.71062 1

10 -7 0 -0.7807 1.99267 3

10 -7 0 1.81165 1.87163 1

-10 7 0 3.62609 2.39236 1

9 -7 0 3.93950 1.64627 1

9 -7 0 1.92359 1.64972 3

-9 7 0 3.46102 2.01264 1

8 -7 0 2.65251 1.55357 1

8 -7 0 0.78850 1.43272 3

7 -7 0 6.50772 1.41225 3

7 -7 0 5.48108 1.03817 5

7 -7 0 4.87362 1.46957 1

6 -7 0 5.66728 1.27304 3

6 -7 0 7.57704 1.52214 1

5 -7 0 215.844 7.95690 1

5 -7 0 221.391 7.61788 6

5 -7 0 242.134 7.92847 3

4 -7 0 95.5458 3.53445 6

3 -7 0 405.989 12.9303 6

3 -7 0 411.842 13.2406 4

3 -7 0 402.984 13.1978 3

3 -7 0 398.950 13.2838 1

-3 7 0 416.988 13.1088 1

-3 7 0 402.304 13.0178 3

-3 7 0 421.112 13.3229 4

-3 7 0 396.825 13.2858 1

2 -7 0 230.298 7.58178 4

2 -7 0 233.405 7.60631 3

-2 7 0 233.534 7.46762 3

-2 7 0 225.242 7.61061 4

-2 7 0 212.658 8.06614 1

-2 7 0 228.163 7.46701 1

1 -7 0 844.287 26.7190 3

1 -7 0 815.668 26.6432 2

1 -7 0 827.057 26.5457 5

-1 7 0 890.380 26.9235 1

-1 7 0 848.516 26.6859 3

-1 7 0 867.244 26.6453 1

0 -7 0 3.14399 0.77922 2

0 -7 0 2.47608 0.71330 5

0 7 0 3.35203 0.71780 3

0 7 0 2.79122 0.81843 1

0 7 0 2.85127 0.64189 1

-1 -7 0 916.537 28.6945 2

-1 -7 0 905.174 28.6554 5

1 7 0 937.056 28.6774 2

1 7 0 905.476 28.7271 1

1 7 0 911.271 28.7235 3

1 7 0 915.283 28.5589 6

1 7 0 910.567 28.6733 1

-2 -7 0 235.533 7.91133 2

-2 -7 0 240.598 7.91043 5

2 7 0 243.414 7.93010 5

2 7 0 233.940 7.88115 2

2 7 0 251.120 7.96725 3

2 7 0 244.800 7.79802 6

2 7 0 243.216 7.88407 1

-3 -7 0 430.589 13.8166 2

-3 -7 0 416.404 14.0024 5

3 7 0 437.045 13.7645 6

3 7 0 441.468 13.7304 1

-4 -7 0 100.086 3.78825 2

-4 -7 0 98.8203 3.82517 5

4 7 0 101.632 3.76361 6

-5 -7 0 256.287 8.45802 5

5 7 0 246.818 8.30630 1

-6 -7 0 9.83612 1.48994 5

6 7 0 11.1337 1.99792 4

6 7 0 9.18894 1.39518 1

-7 -7 0 10.3193 1.60489 5

7 7 0 12.2469 1.66494 1

-8 -7 0 0.09282 1.49459 5

8 7 0 -0.2576 1.07336 1

-9 -7 0 0.93085 1.64959 5

9 7 0 4.42982 1.39017 1

-10 -7 0 -1.5082 2.07249 5

10 7 0 1.71775 1.29986 3

10 7 0 1.25745 1.55414 1

-11 -7 0 7.87256 2.67843 5

11 7 0 1.73166 1.60617 3

11 7 0 3.79375 1.83542 1

-12 -7 0 -1.5007 2.87700 5

12 7 0 2.71760 2.11555 3

-13 -7 0 5.74335 3.77666 5

13 7 0 0.04301 2.32983 3

12 -8 0 -2.7772 3.12820 3

12 -8 0 -2.8211 2.70254 1

12 -8 0 -0.4634 2.79260 6

-12 8 0 -1.9564 3.24806 1

11 -8 0 2.70224 4.44809 3

11 -8 0 1.14982 2.30315 6

11 -8 0 3.76682 2.61911 1

-11 8 0 8.71005 2.97574 1

10 -8 0 -0.1013 1.99951 1

10 -8 0 -2.2197 2.15916 3

10 -8 0 0.16929 1.94028 6

-10 8 0 -2.1160 2.47015 1

9 -8 0 3.16733 1.90702 1

9 -8 0 2.02126 1.82836 6

9 -8 0 0.13934 1.92477 3

-9 8 0 2.03327 2.23497 1

8 -8 0 6.33723 1.71489 1

8 -8 0 9.02963 1.72628 6

8 -8 0 6.41122 1.85393 3

-8 8 0 7.06775 2.06594 1

7 -8 0 1.19018 1.44300 6

7 -8 0 2.50496 1.58169 1

7 -8 0 2.76763 1.55008 3

-7 8 0 2.09096 1.73855 1

6 -8 0 133.701 5.33996 3

6 -8 0 128.646 5.41033 1

-6 8 0 131.494 5.50585 1

5 -8 0 118.095 5.08444 1

5 -8 0 125.281 4.60835 6

5 -8 0 131.250 5.08087 3

-5 8 0 122.874 5.18457 1

4 -8 0 7.36111 1.25983 3

4 -8 0 8.98887 1.42795 1

4 -8 0 9.23722 1.15390 6

-4 8 0 7.52941 1.42597 1

3 -8 0 73.1754 3.35583 4

-3 8 0 75.2836 3.48225 1

-3 8 0 69.2065 3.52328 4

-3 8 0 71.6632 2.98376 1

-3 8 0 81.2093 3.10848 3

2 -8 0 244.656 8.06601 2

2 -8 0 229.913 8.36289 3

-2 8 0 250.126 7.97960 1

-2 8 0 247.674 8.19608 1

-2 8 0 237.115 7.97781 3

1 -8 0 429.911 14.0868 3

1 -8 0 422.345 13.8841 5

1 -8 0 456.038 14.2214 2

-1 8 0 437.674 14.1262 1

-1 8 0 439.522 14.0701 3

-1 8 0 447.953 13.9930 1

0 -8 0 34.1579 1.73526 5

0 -8 0 36.8789 1.87482 2

0 8 0 39.4376 1.73407 1

0 8 0 36.9982 2.06135 3

-1 -8 0 405.574 13.1450 5

1 8 0 381.610 13.3119 1

1 8 0 419.353 13.2582 3

1 8 0 411.113 13.0266 6

1 8 0 442.328 13.1301 1

-2 -8 0 209.742 7.64331 5

-2 -8 0 227.173 7.46351 2

2 8 0 228.468 7.31699 6

2 8 0 226.592 7.49302 3

2 8 0 231.123 7.62550 1

-3 -8 0 98.1068 3.76131 5

-3 -8 0 95.1631 3.71122 2

3 8 0 101.216 3.67696 1

3 8 0 97.9844 3.56577 6

-4 -8 0 6.50028 1.26574 5

-4 -8 0 5.37545 1.04296 2

4 8 0 7.46675 1.06525 6

-5 -8 0 151.550 5.59072 5

5 8 0 157.428 5.28173 1

-6 -8 0 154.072 5.70802 5

6 8 0 156.535 5.31151 1

-7 -8 0 3.66190 1.57059 5

7 8 0 3.07197 0.98324 1

-8 -8 0 7.66357 1.84352 5

8 8 0 5.41041 1.19829 1

-9 -8 0 1.00883 1.93103 5

9 8 0 -0.7821 1.39797 1

-10 -8 0 0.74482 2.12759 5

10 8 0 -0.7421 1.42054 3

10 8 0 -0.0095 1.58466 1

-11 -8 0 6.34904 2.85679 5

11 8 0 3.89623 1.94636 1

11 8 0 3.58728 1.87597 3

-12 -8 0 0.34296 3.13566 5

12 8 0 -2.4292 2.57106 1

12 8 0 -0.0033 2.07818 3

12 -9 0 -0.2842 3.19533 1

12 -9 0 -5.3308 3.29672 3

12 -9 0 1.32127 3.12080 6

-12 9 0 4.30584 3.53657 1

11 -9 0 2.68512 2.58612 6

11 -9 0 -6.2803 2.71882 3

11 -9 0 0.03309 2.70626 1

-11 9 0 5.62200 4.12779 1

10 -9 0 3.82002 2.28869 6

10 -9 0 3.32784 2.40699 1

10 -9 0 0.20648 2.58673 3

-10 9 0 6.55120 2.75657 1

9 -9 0 -0.2894 1.99396 1

9 -9 0 -2.8723 2.16955 3

9 -9 0 -1.1393 1.83861 6

-9 9 0 -0.5715 2.33062 1

8 -9 0 40.7983 3.10663 6

8 -9 0 43.9546 3.92037 1

8 -9 0 42.1249 4.32327 3

-8 9 0 49.5296 4.39465 1

7 -9 0 25.9005 3.28058 3

7 -9 0 20.8855 2.41849 6

7 -9 0 20.9486 3.15280 1

-7 9 0 22.6880 3.46311 1

6 -9 0 122.082 5.51869 1

6 -9 0 135.443 5.54866 3

-6 9 0 130.700 5.70004 1

5 -9 0 110.327 4.14194 6

5 -9 0 115.332 4.67208 3

5 -9 0 107.345 4.78260 1

-5 9 0 103.611 4.85990 1

4 -9 0 688.459 22.0104 6

4 -9 0 718.476 22.3984 3

-4 9 0 684.527 22.5202 1

3 -9 0 199.393 6.83153 2

3 -9 0 192.967 6.99117 4

3 -9 0 203.879 7.01169 3

-3 9 0 197.870 7.12857 1

2 -9 0 598.961 19.2000 5

2 -9 0 598.033 19.4471 4

2 -9 0 636.990 19.4425 2

2 -9 0 584.342 19.6917 3

-2 9 0 623.022 19.4011 1

-2 9 0 618.250 19.6088 1

1 -9 0 35.7680 1.72287 5

1 -9 0 33.6083 1.98303 2

-1 9 0 33.8134 2.02759 3

-1 9 0 35.0392 1.80763 1

-1 9 0 31.8846 2.28148 1

0 -9 0 -1.0176 0.81428 5

0 -9 0 1.32971 0.91547 2

0 9 0 0.26334 0.79508 3

0 9 0 -0.4808 0.69241 1

0 9 0 -0.6997 0.96084 1

-1 -9 0 31.0096 2.12900 5

-1 -9 0 34.2118 1.99553 2

1 9 0 38.7514 1.75459 1

1 9 0 33.3664 2.18018 1

-2 -9 0 643.230 20.1795 5

-2 -9 0 624.225 20.2034 2

2 9 0 649.273 20.2155 1

2 9 0 637.080 20.1888 1

2 9 0 625.255 20.0393 6

-3 -9 0 229.847 7.80187 2

-3 -9 0 233.679 7.79686 5

3 9 0 237.532 7.74434 1

3 9 0 226.030 7.64840 6

-4 -9 0 775.320 24.6515 2

-4 -9 0 779.325 24.6547 5

4 9 0 774.970 24.4798 1

4 9 0 775.303 24.7175 6

-5 -9 0 129.658 4.81298 5

-5 -9 0 118.789 4.75545 2

5 9 0 127.570 4.47672 1

-6 -9 0 147.994 5.62272 5

6 9 0 146.592 5.16263 1

-7 -9 0 23.3798 2.69008 5

7 9 0 31.8991 2.03755 1

-8 -9 0 55.6325 3.75827 5

8 9 0 53.9856 2.88173 1

-9 -9 0 1.49875 2.04497 5

9 9 0 -0.7564 1.41630 1

-10 -9 0 7.12149 3.64555 5

10 9 0 2.39192 1.70694 3

10 9 0 4.18516 1.69002 1

-11 -9 0 -3.6781 2.71216 5

11 9 0 -3.2363 1.88313 3

11 9 0 -0.5207 1.85059 1

-12 -9 0 2.30965 3.45479 5

12 9 0 3.45019 2.06502 1

12 9 0 0.00113 2.36885 3

11 -10 0 -2.5573 2.95960 3

11 -10 0 2.54125 2.75813 6

11 -10 0 -1.9384 3.03484 1

-11 10 0 2.40047 3.27271 1

10 -10 0 -4.0212 3.57494 1

10 -10 0 -1.4320 2.56797 3

10 -10 0 -1.1859 2.26223 6

-10 10 0 0.76155 4.25960 1

9 -10 0 -2.3455 1.90155 6

9 -10 0 -2.5769 2.25975 1

9 -10 0 -2.3808 2.20220 3

-9 10 0 1.82839 2.49455 1

8 -10 0 20.9201 2.62006 3

8 -10 0 20.0688 2.95034 6

8 -10 0 18.4329 2.77324 1

7 -10 0 -0.2979 1.88478 1

7 -10 0 1.69883 1.78087 3

7 -10 0 -0.0110 1.60714 6

-7 10 0 1.34010 1.90790 1

6 -10 0 29.9553 3.41527 1

6 -10 0 27.5337 2.78309 6

6 -10 0 38.9414 3.31758 3

-6 10 0 37.4586 3.49239 1

5 -10 0 11.0558 1.49871 6

5 -10 0 8.42573 1.50283 3

-5 10 0 7.80112 1.68442 1

4 -10 0 236.472 7.91231 3

-4 10 0 207.725 8.06647 1

3 -10 0 15.2354 2.37564 4

3 -10 0 8.58188 1.35275 3

3 -10 0 10.6734 1.39928 2

-3 10 0 8.01875 1.59456 1

2 -10 0 96.6719 3.74827 2

2 -10 0 94.2389 3.87901 3

-2 10 0 89.0834 3.66862 1

-2 10 0 95.2586 4.15232 1

1 -10 0 73.1029 2.99280 5

1 -10 0 79.6567 3.31151 2

-1 10 0 79.0531 3.57606 1

-1 10 0 80.1924 3.13284 1

0 -10 0 118.765 4.42810 2

0 10 0 120.979 4.25769 1

0 10 0 118.737 4.60795 1

-1 -10 0 96.3623 3.77022 2

-1 -10 0 92.0160 3.57821 5

1 10 0 90.3527 3.83795 1

1 10 0 96.8549 3.51709 1

-2 -10 0 103.431 3.97559 5

-2 -10 0 104.638 4.04820 2

2 10 0 106.718 3.82495 6

2 10 0 107.530 3.84320 1

2 10 0 102.379 4.12467 1

-3 -10 0 13.0485 1.43528 2

-3 -10 0 13.3513 1.49070 5

3 10 0 12.1101 1.43254 1

-4 -10 0 238.131 8.32227 2

-4 -10 0 251.472 8.33909 5

4 10 0 242.387 8.14519 1

-5 -10 0 14.2242 1.95072 2

-5 -10 0 15.4303 1.73503 5

5 10 0 11.4981 1.32677 1

-6 -10 0 47.5081 3.11483 5

6 10 0 48.2852 2.44667 1

-7 -10 0 3.38115 1.76800 5

7 10 0 3.96909 1.06375 1

-8 -10 0 37.3136 3.60691 5

-9 -10 0 -0.2063 2.21710 5

-10 -10 0 -2.0435 2.61727 5

10 10 0 1.01458 1.62123 1

-11 -10 0 1.61195 3.35671 5

11 10 0 5.00631 2.13707 3

11 -11 0 -2.7740 3.22176 3

-11 11 0 3.89283 3.68012 1

10 -11 0 -5.0011 2.41564 6

-10 11 0 1.98250 3.13191 1

9 -11 0 6.59264 2.59281 3

9 -11 0 7.32980 2.31814 6

-9 11 0 8.20966 2.90098 1

8 -11 0 11.8518 2.33501 3

8 -11 0 10.7509 2.14560 6

-8 11 0 9.52811 2.73702 1

7 -11 0 20.9951 2.34681 3

7 -11 0 21.4092 2.25134 6

-7 11 0 26.2227 4.03650 1

6 -11 0 40.2961 3.55196 3

6 -11 0 31.5146 2.61054 6

-6 11 0 39.6967 3.70723 1

5 -11 0 46.4643 3.35944 3

-5 11 0 41.8328 3.57203 1

4 -11 0 13.5306 1.58982 2

4 -11 0 10.4011 1.50269 3

-4 11 0 8.72275 1.87772 1

3 -11 0 4.62697 1.35973 2

3 -11 0 2.49533 1.10215 3

3 -11 0 5.22720 1.84980 4

-3 11 0 0.68431 1.60151 1

2 -11 0 6.34326 1.04125 5

2 -11 0 6.60267 1.17377 3

2 -11 0 5.28723 1.22363 2

-2 11 0 3.94510 1.58584 1

1 -11 0 246.927 8.13962 2

-1 11 0 241.079 8.32255 1

-1 11 0 227.819 7.99711 1

0 -11 0 -1.0669 1.04211 2

0 11 0 -0.6817 1.26605 1

0 11 0 0.38088 0.90366 1

-1 -11 0 236.086 8.03517 2

-1 -11 0 231.508 7.88339 5

1 11 0 232.838 8.09816 1

1 11 0 243.760 7.87011 1

-2 -11 0 7.53936 1.27506 2

-2 -11 0 9.27011 1.31918 5

2 11 0 9.19994 1.11382 1

2 11 0 11.1523 1.27729 6

2 11 0 8.80947 1.43827 1

-3 -11 0 1.59487 1.17664 2

-3 -11 0 1.56255 1.18110 5

3 11 0 4.48141 1.17502 6

3 11 0 2.76262 1.30735 1

-4 -11 0 13.9428 1.64795 2

-4 -11 0 14.2736 1.71933 5

4 11 0 14.5721 1.52301 1

-5 -11 0 44.1478 2.78316 2

-5 -11 0 44.7484 2.92910 5

5 11 0 42.7215 2.56973 1

-6 -11 0 53.7558 3.38395 5

6 11 0 52.6563 2.64289 1

-7 -11 0 34.0457 3.37019 5

-8 -11 0 20.5730 2.74136 5

-9 -11 0 12.6135 3.77389 5

-10 -11 0 -2.3603 2.82512 5

-11 -11 0 -3.0014 3.44211 5

11 11 0 1.58594 2.49440 3

10 -12 0 -3.1192 2.65081 3

-10 12 0 0.42330 3.34470 1

9 -12 0 2.25535 2.36769 3

-9 12 0 0.90192 3.08609 1

8 -12 0 -2.2421 2.24542 6

8 -12 0 1.68504 2.11146 3

-8 12 0 3.03996 2.72104 1

7 -12 0 16.2967 2.27791 3

-7 12 0 14.2361 2.89573 1

6 -12 0 31.1420 3.68815 3

-6 12 0 36.8320 4.27456 1

5 -12 0 26.3900 2.65987 2

5 -12 0 29.0087 3.25934 3

-5 12 0 28.3163 3.65553 1

4 -12 0 50.0972 3.05881 2

4 -12 0 49.6450 3.43400 3

-4 12 0 51.6305 3.96406 1

3 -12 0 81.0784 3.73157 2

3 -12 0 81.7078 3.91461 3

-3 12 0 77.6452 4.36515 1

2 -12 0 23.1466 2.31331 2

-2 12 0 18.4266 2.90801 1

1 -12 0 11.6156 1.60427 2

1 -12 0 12.2798 1.47767 5

-1 12 0 9.33367 1.30636 1

-1 12 0 10.4786 1.81516 1

0 -12 0 20.0807 1.86256 5

0 -12 0 18.8030 2.20850 2

0 12 0 17.4255 1.90947 1

0 12 0 16.9110 2.64149 1

-1 -12 0 11.9562 1.57693 2

-1 -12 0 12.4585 1.79681 5

1 12 0 12.2970 1.70541 1

1 12 0 10.8258 1.64796 1

-2 -12 0 21.4689 2.08376 5

-2 -12 0 23.5218 2.26204 2

2 12 0 24.4072 2.53631 1

2 12 0 25.3589 2.00505 1

-3 -12 0 73.6873 3.51383 2

-3 -12 0 77.6084 3.45918 5

3 12 0 73.4212 3.17532 6

3 12 0 74.0984 3.20221 1

3 12 0 77.4039 3.66254 1

-4 -12 0 60.6425 3.22903 2

-4 -12 0 64.0986 3.20501 5

4 12 0 55.9093 3.14949 1

4 12 0 54.2000 2.73951 1

-5 -12 0 32.3931 2.73864 2

-5 -12 0 33.3397 2.85197 5

5 12 0 34.8294 2.44040 1

-6 -12 0 47.5511 3.47766 5

-7 -12 0 23.9701 3.40267 5

-8 -12 0 3.39644 2.47449 5

-9 -12 0 -0.1240 2.76877 5

-10 -12 0 4.06120 3.24498 5

10 -13 0 3.47255 2.87448 3

-10 13 0 0.58994 3.78048 1

-9 13 0 5.02363 3.43161 1

8 -13 0 2.60135 2.34856 3

-8 13 0 2.68313 3.10777 1

7 -13 0 1.70110 1.95249 3

-7 13 0 2.14838 2.94077 1

6 -13 0 1.91611 1.72528 3

6 -13 0 1.93099 1.64363 2

-6 13 0 -1.8245 2.53476 1

5 -13 0 9.06987 1.81407 2

5 -13 0 8.83562 1.84555 3

-5 13 0 11.4870 2.55469 1

4 -13 0 13.1087 1.75118 3

4 -13 0 14.9329 1.86528 2

-4 13 0 10.4205 2.45356 1

3 -13 0 24.6020 2.79289 3

3 -13 0 23.1949 2.68298 2

-3 13 0 22.2651 3.49220 1

2 -13 0 43.1687 2.95229 2

-2 13 0 37.8373 3.45272 1

1 -13 0 -1.0955 1.30152 2

1 -13 0 0.52723 1.12301 5

-1 13 0 2.56627 1.73899 1

0 -13 0 -2.5055 1.26279 2

0 -13 0 0.20288 1.20455 5

0 13 0 -0.7697 1.53373 1

-1 -13 0 1.26897 1.33735 2

-1 -13 0 2.35150 1.28703 5

1 13 0 3.63200 1.67928 1

-2 -13 0 45.5227 2.88315 2

-2 -13 0 42.5861 2.67995 5

2 13 0 42.4685 3.09825 1

-3 -13 0 25.2190 2.48079 5

-3 -13 0 22.0170 2.48593 2

3 13 0 20.9975 2.75793 1

-4 -13 0 14.7778 1.99560 5

-4 -13 0 13.1017 1.92159 2

4 13 0 16.9844 2.49019 1

-5 -13 0 13.6103 2.09698 5

-5 -13 0 12.9737 1.98870 2

5 13 0 8.50663 1.53915 1

-6 -13 0 -0.5121 1.91573 5

-7 -13 0 4.89686 2.32974 5

-8 -13 0 2.60444 2.82077 5

-9 -13 0 0.75305 2.97128 5

-10 -13 0 -1.6981 3.38601 5

9 -14 0 6.04050 2.77642 3

-9 14 0 3.96578 3.77940 1

8 -14 0 11.2404 2.64077 3

8 -14 0 10.3049 2.35659 2

-8 14 0 12.6557 3.62699 1

7 -14 0 7.81530 2.22491 3

7 -14 0 6.58135 2.22121 2

-7 14 0 10.5434 3.25053 1

6 -14 0 0.97294 1.78793 2

6 -14 0 -1.0745 1.70784 3

5 -14 0 9.11855 1.89616 3

5 -14 0 12.2142 2.01756 2

-5 14 0 11.4407 2.95751 1

4 -14 0 3.02185 1.51839 3

4 -14 0 2.61738 1.62605 2

-4 14 0 2.24323 2.43811 1

3 -14 0 2.26071 1.60783 2

-3 14 0 0.37743 2.21029 1

2 -14 0 -0.0532 1.58303 2

-2 14 0 -3.1778 1.98714 1

1 -14 0 18.0936 2.03633 2

1 -14 0 14.3331 1.99884 5

-1 14 0 11.9943 2.28432 1

0 -14 0 33.8926 2.40190 5

0 -14 0 36.4921 2.96398 2

0 14 0 34.7945 3.38349 1

-1 -14 0 16.0973 2.06581 2

-1 -14 0 16.5056 2.18857 5

1 14 0 14.1085 2.10015 1

-2 -14 0 1.06022 1.46188 5

-2 -14 0 -0.8236 1.54408 2

2 14 0 -0.2082 1.81662 1

-3 -14 0 3.50098 1.64901 5

-3 -14 0 1.89890 1.49203 2

3 14 0 1.04733 1.68366 1

-4 -14 0 2.28886 1.58355 2

-4 -14 0 -0.0158 1.73063 5

4 14 0 3.14964 1.65621 1

-5 -14 0 12.3477 2.18494 2

-5 -14 0 11.3408 2.26977 5

5 14 0 11.3976 1.67727 1

-6 -14 0 -0.2447 2.17396 5

-7 -14 0 7.85673 3.66287 5

-8 -14 0 19.2045 3.34700 5

-9 -14 0 7.90724 3.28677 5

8 -15 0 7.89568 2.72120 3

8 -15 0 4.90822 2.46494 2

-8 15 0 4.11472 3.64671 1

7 -15 0 10.4937 2.44219 2

-7 15 0 14.8909 3.77148 1

6 -15 0 15.7504 2.45163 2

-6 15 0 22.7740 3.65863 1

5 -15 0 1.36295 1.69374 3

5 -15 0 0.40088 1.89867 2

-5 15 0 4.16304 2.89722 1

4 -15 0 0.34260 1.86885 2

-4 15 0 1.30536 2.68475 1

3 -15 0 1.22237 1.77979 2

-3 15 0 0.61834 2.54523 1

2 -15 0 1.76136 1.71621 2

-2 15 0 -0.9276 2.22956 1

1 -15 0 8.49797 1.39636 5

1 -15 0 7.80127 1.87783 2

-1 15 0 6.10718 2.23679 1

0 -15 0 0.65972 1.60298 2

0 -15 0 1.79966 1.41356 5

0 15 0 1.28497 2.05528 1

-1 -15 0 7.94913 1.64458 5

-1 -15 0 7.70132 1.84666 2

1 15 0 5.95585 2.13270 1

-2 -15 0 0.94178 1.66882 2

-2 -15 0 1.53877 1.57045 5

2 15 0 -1.4924 1.92043 1

-3 -15 0 0.55425 1.72170 5

-3 -15 0 0.98540 1.75576 2

3 15 0 0.49527 1.90069 1

-4 -15 0 0.50859 1.73789 2

-4 -15 0 -2.4642 1.82981 5

4 15 0 -1.6684 1.72124 1

-5 -15 0 1.95571 1.88944 2

-5 -15 0 0.64291 2.19091 5

-6 -15 0 22.7074 4.15922 5

-7 -15 0 15.9524 3.24475 5

-8 -15 0 4.24362 3.20640 5

7 -16 0 10.2007 2.50737 3

7 -16 0 9.20377 2.63993 2

-7 16 0 14.1375 3.96274 1

6 -16 0 4.48640 2.93777 2

6 -16 0 4.81862 2.08879 3

-6 16 0 6.00663 3.68586 1

5 -16 0 5.60240 2.37549 2

5 -16 0 3.90533 1.88666 3

-5 16 0 0.74479 3.23110 1

4 -16 0 5.21040 2.31197 2

-4 16 0 2.24211 3.27073 1

3 -16 0 6.82249 2.22013 2

-3 16 0 12.6100 5.17599 1

2 -16 0 0.55547 2.08230 2

-2 16 0 -0.3082 2.53249 1

1 -16 0 3.06122 2.00270 2

-1 16 0 1.92171 2.37805 1

0 -16 0 4.05784 1.48039 5

0 -16 0 1.52390 1.91087 2

0 16 0 1.74341 2.29189 1

-1 -16 0 2.23687 1.91835 2

-1 -16 0 2.11417 1.57067 5

1 16 0 0.95743 2.16132 1

-2 -16 0 -0.5818 1.72560 5

-2 -16 0 1.12344 1.79141 2

2 16 0 -0.8873 2.11001 1

-3 -16 0 7.04114 2.14104 2

-3 -16 0 4.50157 2.00105 5

3 16 0 5.03321 2.20462 1

-4 -16 0 6.69317 2.17517 5

-4 -16 0 8.69316 3.36677 2

4 16 0 4.30380 1.96420 1

-5 -16 0 4.85078 2.77612 5

-6 -16 0 4.92525 2.82605 5

-7 -16 0 7.09675 3.32457 5

6 -17 0 2.41885 2.76480 2

-6 17 0 0.55455 3.91833 1

5 -17 0 8.76977 2.77770 2

-5 17 0 6.60949 3.77811 1

4 -17 0 1.18217 2.49654 2

-4 17 0 -3.3618 3.27471 1

3 -17 0 1.20538 2.39253 2

-3 17 0 3.26170 3.13077 1

2 -17 0 11.4174 2.45600 2

-2 17 0 11.4232 3.15124 1

1 -17 0 1.00001 2.22785 2

-1 17 0 1.03073 2.78094 1

0 -17 0 0.03881 2.21270 2

0 -17 0 0.57672 1.59312 5

-1 -17 0 -0.8495 1.66813 5

-1 -17 0 0.60355 2.12189 2

1 17 0 -0.1036 2.50378 1

-2 -17 0 14.2315 2.14256 5

2 17 0 7.98985 2.57964 1

-3 -17 0 0.18643 2.14184 2

-3 -17 0 1.38919 2.13511 5

3 17 0 3.33267 2.34996 1

-4 -17 0 2.56202 2.45953 2

-4 -17 0 -3.4499 2.34383 5

4 17 0 -1.9860 2.08058 1

-5 -17 0 6.43339 2.72427 5

-6 -17 0 5.43991 2.96738 5

4 -18 0 -0.7042 2.80455 2

-4 18 0 5.81872 3.91212 1

3 -18 0 1.94143 2.48365 2

-3 18 0 4.87437 3.47501 1

2 -18 0 -0.1432 2.48014 2

-2 18 0 3.23488 3.22575 1

1 -18 0 0.34745 2.45507 2

-1 18 0 -1.7087 2.85621 1

0 -18 0 21.4566 2.99060 2

0 18 0 15.5487 3.24341 1

-1 -18 0 0.68208 2.35620 2

1 18 0 -2.9668 2.66987 1

-2 -18 0 0.48545 2.40504 2

-2 -18 0 1.80074 2.07466 5

2 18 0 -4.2270 2.60617 1

-3 -18 0 0.41245 2.28741 5

-3 -18 0 2.43676 2.53849 2

-4 -18 0 4.97599 2.73859 5

-4 -18 0 4.18150 2.78168 2

1 -19 0 4.30421 2.90898 2

-1 19 0 -0.6781 3.22269 1

0 -19 0 3.30313 2.92232 2

0 19 0 1.49208 3.04389 1

-1 -19 0 3.13349 2.83726 2

1 19 0 1.36133 3.18076 1

1 19 -1 -3.7936 3.02406 1

-1 -19 1 5.30248 2.85182 2

0 19 -1 2.15384 3.10974 1

0 -19 1 3.80531 2.85117 2

-1 19 -1 3.50711 3.31327 1

-4 -18 1 3.83426 2.65948 5

-4 -18 1 0.41810 2.54064 2

3 18 -1 2.10779 4.29951 1

-3 -18 1 -0.1802 2.67384 2

-3 -18 1 0.46744 2.27878 5

2 18 -1 0.02397 2.62142 1

-2 -18 1 2.48327 2.81302 5

-2 -18 1 -1.8938 2.41708 2

1 18 -1 -1.6418 2.69181 1

-1 -18 1 -1.2768 2.34225 2

0 18 -1 -1.4406 2.82870 1

0 -18 1 -0.4952 2.54118 2

-1 18 -1 -0.7262 2.98065 1

1 -18 1 3.66605 2.52724 2

-2 18 -1 -1.7943 3.02956 1

2 -18 1 -0.1783 2.46125 2

-3 18 -1 -0.5472 3.33342 1

3 -18 1 -1.9376 2.71774 2

-4 18 -1 1.41878 3.73898 1

4 -18 1 5.25856 2.84179 2

-6 -17 1 3.69812 3.00575 5

-5 -17 1 13.7119 2.92846 5

-5 -17 1 15.5892 2.93930 2

-4 -17 1 0.87101 2.32257 2

-4 -17 1 1.86187 2.28351 5

3 17 -1 1.26129 2.28368 1

-3 -17 1 2.27533 2.17738 5

-3 -17 1 -0.3170 2.24097 2

2 17 -1 2.89249 2.42611 1

-2 -17 1 -0.2726 2.76271 2

-2 -17 1 4.11407 1.94480 5

1 17 -1 -1.5984 2.35821 1

-1 -17 1 0.02354 1.64262 5

-1 -17 1 2.31867 2.35178 2

0 17 -1 5.02438 3.02944 1

0 -17 1 9.66688 2.56649 2

-1 17 -1 -1.1238 2.81371 1

1 -17 1 0.63608 2.13705 2

-2 17 -1 5.95189 2.89828 1

2 -17 1 3.00400 2.34088 2

-3 17 -1 -2.6314 2.97926 1

3 -17 1 1.48598 2.51103 2

-4 17 -1 2.20688 3.39541 1

4 -17 1 1.23204 3.19047 2

-5 17 -1 3.88683 3.60089 1

5 -17 1 5.26114 2.67910 2

-6 17 -1 2.13299 3.73807 1

6 -17 1 4.64442 2.90503 2

-7 -16 1 13.8051 3.25730 5

-6 -16 1 10.2915 2.89083 5

-5 -16 1 5.00867 2.33595 2

-5 -16 1 5.28105 2.38238 5

-4 -16 1 22.5239 2.60746 5

3 16 -1 4.51726 2.12231 1

-3 -16 1 8.19690 2.21977 2

-3 -16 1 11.2438 2.15245 5

-2 -16 1 21.7027 2.62178 2

-2 -16 1 21.9637 2.72243 5

1 16 -1 2.25099 2.24342 1

-1 -16 1 -0.7581 1.91564 2

-1 -16 1 -1.0507 1.43583 5

0 16 -1 -2.5739 2.17136 1

0 -16 1 0.99869 1.86056 2

0 -16 1 -0.3840 1.30487 5

-1 16 -1 8.76949 2.50850 1

1 -16 1 7.29428 2.09601 2

-2 16 -1 47.0330 4.85144 1

2 -16 1 46.7994 3.94068 2

-3 16 -1 1.81438 3.75258 1

3 -16 1 -0.3508 2.07168 2

-4 16 -1 1.33681 2.94001 1

4 -16 1 -0.1410 2.12676 2

-5 16 -1 -2.5982 3.07392 1

5 -16 1 0.61023 2.32075 2

-6 16 -1 1.68811 3.35723 1

6 -16 1 5.13660 2.52660 2

6 -16 1 5.43385 1.99278 3

-7 16 -1 4.92647 3.77603 1

7 -16 1 2.70509 2.55484 2

-8 -15 1 4.22015 3.06439 5

-7 -15 1 16.4335 3.09164 5

-6 -15 1 2.35649 2.49993 5

-5 -15 1 6.19078 2.04973 2

-5 -15 1 9.91063 2.32367 5

4 15 -1 2.53915 1.64523 1

-4 -15 1 -1.4206 1.86111 5

-4 -15 1 -1.4636 1.63719 2

3 15 -1 15.7614 2.14367 1

-3 -15 1 19.6357 2.74565 5

-3 -15 1 15.6346 2.26007 2

2 15 -1 1.53977 1.89181 1

-2 -15 1 0.50530 1.48523 5

-2 -15 1 -0.7523 1.69178 2

1 15 -1 0.40471 1.88423 1

-1 -15 1 0.95329 1.65232 2

-1 -15 1 -0.3578 1.38914 5

0 15 -1 48.8465 3.96639 1

0 -15 1 54.0431 2.77509 5

0 -15 1 49.6032 3.61994 2

-1 15 -1 1.66318 2.15244 1

1 -15 1 6.96670 1.99530 2

-2 15 -1 -1.2836 2.20352 1

2 -15 1 1.74493 1.76981 2

-3 15 -1 0.01776 2.44895 1

3 -15 1 1.60536 1.85817 2

-4 15 -1 48.6991 4.97523 1

4 -15 1 34.1391 3.71206 2

-5 15 -1 -0.0364 2.78911 1

5 -15 1 0.97060 1.69179 3

5 -15 1 -1.2720 1.93268 2

-6 15 -1 4.89728 3.20264 1

6 -15 1 4.35945 1.87527 3

6 -15 1 0.56624 2.10055 2

-7 15 -1 5.51858 3.49601 1

7 -15 1 7.19463 2.16595 3

-8 15 -1 -2.0616 3.42913 1

8 -15 1 3.03906 2.47127 2

8 -15 1 4.65634 2.75312 3

-9 -14 1 2.62723 3.23611 5

-8 -14 1 2.62068 2.84511 5

-6 -14 1 1.42241 2.14429 5

-5 -14 1 68.1252 3.98074 5

-5 -14 1 59.9108 3.92964 2

4 14 -1 29.5018 2.71476 1

-4 -14 1 31.4812 2.97111 5

-4 -14 1 27.0122 3.00285 2

3 14 -1 4.54386 1.72914 1

-3 -14 1 5.13085 1.72088 2

-3 -14 1 6.78157 1.71992 5

2 14 -1 7.19958 1.84864 1

-2 -14 1 11.0172 1.69356 5

-2 -14 1 7.19173 1.67772 2

1 14 -1 137.306 5.75124 1

-1 -14 1 139.744 5.18477 5

-1 -14 1 142.791 5.62710 2

0 14 -1 8.44169 1.97371 1

0 -14 1 6.45304 1.39789 5

0 -14 1 7.31818 1.71603 2

-1 14 -1 6.76352 2.08845 1

1 -14 1 12.2183 1.87682 2

-2 14 -1 3.80486 2.05531 1

2 -14 1 5.30897 1.84280 2

-3 14 -1 55.0576 4.37464 1

3 -14 1 52.7630 3.60362 2

-4 14 -1 1.15734 2.36110 1

4 -14 1 2.28993 1.40172 3

4 -14 1 0.31052 1.67436 2

-5 14 -1 10.9712 2.83560 1

5 -14 1 10.1897 1.81766 3

5 -14 1 11.4132 2.01534 2

6 -14 1 14.2551 2.26551 2

6 -14 1 16.4150 2.26055 3

-7 14 -1 5.57353 3.14806 1

7 -14 1 5.75639 2.15713 3

7 -14 1 4.75739 2.13123 2

-8 14 -1 6.63812 3.34921 1

8 -14 1 6.11484 2.49317 3

-9 14 -1 12.8592 3.84039 1

9 -14 1 9.38672 2.76345 3

-10 -13 1 0.54820 3.56537 5

-9 -13 1 -1.6741 2.82593 5

-8 -13 1 -1.0818 2.61654 5

-7 -13 1 23.6188 2.94894 5

-6 -13 1 17.1629 2.44425 5

-5 -13 1 44.3100 3.22170 5

-5 -13 1 36.9147 3.15500 2

4 13 -1 47.5824 2.85070 1

-4 -13 1 42.1121 3.06475 2

-4 -13 1 45.9732 3.10437 5

3 13 -1 84.6883 3.94387 1

-3 -13 1 88.9150 3.86327 5

-3 -13 1 85.2900 4.04093 2

2 13 -1 2.59155 1.56359 1

-2 -13 1 3.23752 1.40644 5

-2 -13 1 3.57299 1.47624 2

1 13 -1 4.21314 1.64259 1

-1 -13 1 6.22379 1.32835 5

-1 -13 1 5.23776 1.58527 2

0 13 -1 -0.2515 1.60794 1

0 -13 1 -1.0473 1.40238 2

0 -13 1 0.33120 1.08414 5

-1 13 -1 143.758 5.82538 1

1 -13 1 140.048 5.52571 2

-2 13 -1 2.40802 1.82059 1

2 -13 1 0.45948 1.40194 2

-3 13 -1 20.6290 2.50131 1

3 -13 1 22.5161 2.67489 2

3 -13 1 24.0177 2.62186 3

-4 13 -1 -1.5854 1.93789 1

4 -13 1 0.34276 1.26423 3

4 -13 1 1.72408 1.48576 2

-5 13 -1 -2.5725 2.16068 1

5 -13 1 -0.6351 1.46631 3

5 -13 1 -2.1907 1.58880 2

-6 13 -1 0.50109 2.41929 1

6 -13 1 -0.8377 1.60411 2

6 -13 1 1.37010 1.60325 3

-7 13 -1 2.02633 3.76835 1

7 -13 1 -1.4407 1.83542 3

-8 13 -1 17.2055 3.36695 1

8 -13 1 13.2085 2.50779 3

-9 13 -1 10.4407 3.47544 1

9 -13 1 0.80156 2.98664 3

-10 13 -1 5.88104 3.79005 1

10 -13 1 1.60900 2.87037 3

-10 -12 1 -1.4064 3.22029 5

-9 -12 1 1.16234 2.65578 5

-8 -12 1 1.42030 2.33709 5

-7 -12 1 6.83376 2.18844 5

-6 -12 1 72.1201 4.01503 5

-5 -12 1 1.76355 1.45950 2

-5 -12 1 2.21385 1.60713 5

4 12 -1 17.1578 1.80169 1

4 12 -1 13.7201 2.14692 1

-4 -12 1 20.0153 2.33320 2

-4 -12 1 20.1800 2.33224 5

3 12 -1 -1.9909 1.31550 1

3 12 -1 0.85797 0.97326 1

3 12 -1 3.25764 1.13930 6

-3 -12 1 -1.7433 1.22135 5

-3 -12 1 -2.2786 1.15929 2

2 12 -1 125.339 4.80576 1

2 12 -1 115.047 4.35844 1

-2 -12 1 123.356 4.54902 5

-2 -12 1 118.439 4.77041 2

1 12 -1 1.74054 1.34721 1

1 12 -1 2.50416 0.97748 1

-1 -12 1 0.80855 1.12056 5

-1 -12 1 1.16647 1.33763 2

0 12 -1 49.3677 2.63839 1

0 12 -1 48.9645 3.19225 1

0 -12 1 55.8132 2.48018 5

0 -12 1 48.6403 2.89273 2

-1 12 -1 52.2364 3.29225 1

-1 12 -1 52.7057 2.72973 1

1 -12 1 54.1717 2.99779 2

-2 12 -1 5.65229 1.69728 1

2 -12 1 7.73686 1.52834 2

-3 12 -1 2.96639 1.79726 1

3 -12 1 5.35379 1.23049 3

3 -12 1 5.36481 1.42212 2

-4 12 -1 13.5011 2.12604 1

4 -12 1 14.4204 1.80600 2

4 -12 1 13.5127 1.68318 3

-5 12 -1 0.74781 1.90994 1

5 -12 1 4.04935 1.59705 2

5 -12 1 4.34472 1.46052 3

-6 12 -1 32.3541 4.07085 1

6 -12 1 30.5140 3.53461 3

-7 12 -1 0.68761 2.32728 1

7 -12 1 0.86472 1.72581 3

-8 12 -1 9.73003 3.20105 1

8 -12 1 7.52246 2.21836 3

-9 12 -1 3.86725 3.01891 1

9 -12 1 3.66003 2.35230 3

-10 12 -1 10.6391 3.53664 1

10 -12 1 3.93210 2.80533 3

11 11 -1 4.67199 2.37738 3

-11 -11 1 5.70926 3.37685 5

-10 -11 1 -0.9514 2.76833 5

-8 -11 1 4.21529 2.20988 5

-7 -11 1 -0.6826 1.87127 5

-6 -11 1 20.8052 2.72305 5

-5 -11 1 0.92608 1.48355 5

-5 -11 1 0.83482 1.27514 2

4 11 -1 73.4020 3.10464 6

4 11 -1 71.6633 2.95527 1

4 11 -1 77.4072 3.20348 1

-4 -11 1 74.7088 3.34343 5

-4 -11 1 69.3491 3.37600 2

3 11 -1 152.260 5.26555 6

3 11 -1 150.055 5.52305 1

3 11 -1 148.776 5.22261 1

-3 -11 1 151.396 5.61379 2

-3 -11 1 156.577 5.49865 5

2 11 -1 188.950 6.66301 1

2 11 -1 193.877 6.43264 1

-2 -11 1 191.620 6.74171 2

-2 -11 1 190.342 6.55793 5

1 11 -1 34.3967 1.95199 1

1 11 -1 29.8286 2.50225 1

-1 -11 1 35.4651 2.09700 5

-1 -11 1 35.1783 2.37342 2

0 11 -1 60.9173 3.06147 1

0 11 -1 58.6702 2.55580 1

0 -11 1 58.3259 2.84942 2

0 -11 1 51.6030 2.69204 5

-1 11 -1 0.64844 1.31938 1

-1 11 -1 0.90914 0.99703 1

1 -11 1 1.12789 1.05917 2

1 -11 1 1.66059 0.91666 5

-2 11 -1 0.25008 0.99188 1

-2 11 -1 -0.9754 1.39198 1

2 -11 1 0.77082 1.12369 2

2 -11 1 -0.3871 0.89953 3

-3 11 -1 6.95344 1.69226 1

3 -11 1 10.5157 1.50504 2

3 -11 1 8.10978 1.28550 3

-4 11 -1 -2.2026 1.44753 1

4 -11 1 -0.7715 1.11852 3

4 -11 1 1.00935 1.27588 2

-5 11 -1 80.4398 4.41392 1

5 -11 1 82.4549 4.14339 3

-6 11 -1 62.2226 4.23092 1

6 -11 1 60.5099 3.96655 3

-7 11 -1 2.74673 2.12562 1

7 -11 1 0.69685 1.65535 6

7 -11 1 -0.5023 2.18911 1

7 -11 1 -0.9526 1.67562 3

-8 11 -1 2.03542 2.43663 1

8 -11 1 -1.8208 1.64467 6

8 -11 1 1.00851 2.32427 1

8 -11 1 0.90102 1.99479 3

9 -11 1 -0.5825 2.72053 1

9 -11 1 -1.0229 2.34060 3

9 -11 1 -2.5497 2.01174 6

-10 11 -1 1.70915 3.11383 1

10 -11 1 3.75839 3.45808 3

10 -11 1 0.22916 3.08785 6

10 -11 1 1.16165 3.04407 1

-11 11 -1 0.77522 3.43157 1

11 -11 1 -1.7914 3.12573 3

11 -11 1 -1.5652 3.44730 1

-11 -10 1 11.6449 3.28627 5

10 10 -1 -0.0659 2.07344 3

-10 -10 1 -1.5981 2.58855 5

-9 -10 1 7.25070 2.31051 5

-8 -10 1 4.68521 2.02074 5

-7 -10 1 3.55511 1.77056 5

-6 -10 1 -1.4812 1.47540 5

5 10 -1 11.7907 1.20810 1

-5 -10 1 16.0282 2.08557 2

-5 -10 1 15.4351 1.76119 5

4 10 -1 147.417 5.09219 1

4 10 -1 134.782 5.03736 6

-4 -10 1 145.716 5.31925 2

-4 -10 1 145.929 5.30743 5

3 10 -1 21.3005 1.54237 6

3 10 -1 20.3244 1.51906 1

3 10 -1 16.6494 1.98764 1

-3 -10 1 19.6867 1.84469 5

-3 -10 1 17.7333 1.96209 2

2 10 -1 0.57374 1.12532 1

-2 -10 1 1.50599 1.10865 2

-2 -10 1 0.09692 1.02690 5

1 10 -1 26.9034 1.57225 1

1 10 -1 23.9077 2.20144 1

-1 -10 1 26.0087 2.07243 2

0 10 -1 56.3253 2.86441 1

0 10 -1 57.8836 2.42602 1

0 -10 1 55.3070 2.70348 2

0 -10 1 56.2017 2.42296 5

-1 10 -1 10.7107 1.47930 1

-1 10 -1 11.1247 1.48820 1

1 -10 1 9.84891 1.40868 2

1 -10 1 11.5698 1.34949 5

-2 10 -1 -0.8107 1.84147 4

-2 10 -1 3.29957 1.02369 1

-2 10 -1 1.21452 1.29104 1

2 -10 1 3.36463 1.12411 2

2 -10 1 3.02510 0.96253 3

-3 10 -1 75.0002 3.73876 1

3 -10 1 81.2311 3.47176 2

3 -10 1 79.7271 3.59396 3

-4 10 -1 44.2198 3.42277 1

4 -10 1 54.3847 3.02212 3

-5 10 -1 20.2807 2.92783 1

5 -10 1 13.3835 1.62920 3

5 -10 1 9.45828 1.84979 1

-6 10 -1 72.5459 4.27075 1

6 -10 1 69.1547 3.91971 3

6 -10 1 62.8032 4.05562 1

6 -10 1 54.2760 4.81616 6

-7 10 -1 93.5297 5.14132 1

7 -10 1 89.3511 4.94992 1

7 -10 1 87.4673 4.90365 3

7 -10 1 79.7733 3.93611 6

-8 10 -1 12.6332 2.66029 1

8 -10 1 13.5929 2.01256 6

8 -10 1 7.69767 2.31041 1

8 -10 1 8.35175 2.14069 3

-9 10 -1 4.26255 2.61286 1

9 -10 1 2.03309 2.36447 1

9 -10 1 3.47732 2.21042 3

9 -10 1 1.47765 1.98324 6

10 -10 1 -0.3682 2.22129 6

10 -10 1 -0.6003 2.71464 3

10 -10 1 3.69262 2.95181 1

-11 10 -1 5.52293 3.39134 1

11 -10 1 0.53891 2.94123 1

11 -10 1 -2.0022 2.59069 6

11 -10 1 -1.1005 3.14754 3

12 9 -1 -1.3313 2.21968 3

-12 -9 1 -0.2436 3.33223 5

11 9 -1 -1.5539 1.76966 3

-11 -9 1 3.48091 2.91589 5

10 9 -1 0.90766 1.50570 3

9 9 -1 0.42669 1.30319 1

-9 -9 1 1.07741 2.11462 5

8 9 -1 -0.6627 1.05478 1

-8 -9 1 -0.1494 1.82561 5

-7 -9 1 12.4105 1.89859 5

-6 -9 1 86.4164 3.90594 5

5 9 -1 55.8721 2.30501 1

-5 -9 1 51.0534 2.84836 5

-5 -9 1 47.3490 2.75744 2

4 9 -1 1.90659 0.93877 1

4 9 -1 1.28907 0.98579 6

-4 -9 1 2.94113 1.16372 2

-4 -9 1 -1.2714 1.12653 5

3 9 -1 20.1346 1.95978 1

3 9 -1 16.8717 1.36379 6

-3 -9 1 14.4471 1.78289 2

-3 -9 1 14.0104 1.74895 5

2 9 -1 15.2578 1.27591 6

2 9 -1 16.0653 1.30056 1

2 9 -1 11.9643 1.74867 1

-2 -9 1 14.4660 1.57893 5

-2 -9 1 16.1366 1.66542 2

1 9 -1 7.63729 1.10294 1

1 9 -1 5.49199 0.93671 3

1 9 -1 7.64933 1.09313 1

-1 -9 1 4.98728 1.10737 2

0 9 -1 0.92117 0.86052 3

0 9 -1 1.66853 0.68185 1

0 9 -1 1.25853 0.97449 1

0 -9 1 1.92577 0.81111 5

0 -9 1 0.36838 0.97723 2

-1 9 -1 51.5035 2.48474 3

-1 9 -1 50.6481 2.61615 1

-1 9 -1 53.0672 2.26667 1

1 -9 1 51.3524 2.15475 5

1 -9 1 54.8016 2.51486 2

-2 9 -1 1.22775 0.75310 3

-2 9 -1 -1.4072 0.82253 1

2 -9 1 2.20001 1.03899 2

2 -9 1 -0.4512 1.51307 4

2 -9 1 0.40150 0.87792 3

-3 9 -1 -0.2339 1.76556 4

-3 9 -1 -1.0915 1.21577 1

3 -9 1 3.19922 1.71807 4

3 -9 1 -0.3161 0.98481 3

-4 9 -1 33.8068 3.05655 4

-4 9 -1 21.6780 2.80607 1

4 -9 1 23.0567 2.64724 1

4 -9 1 28.2044 2.53282 3

-5 9 -1 6.56883 1.65212 1

5 -9 1 6.33769 1.28028 3

5 -9 1 7.78834 1.21111 6

5 -9 1 3.42146 1.39845 1

-6 9 -1 -0.3234 1.51612 1

6 -9 1 -0.5719 1.50800 1

6 -9 1 0.23678 1.41724 6

6 -9 1 0.08066 1.35756 3

-7 9 -1 6.48430 1.89923 1

7 -9 1 2.79598 1.67254 3

7 -9 1 3.97562 1.72473 1

7 -9 1 5.90572 1.51282 6

-8 9 -1 14.2065 2.43873 1

8 -9 1 11.1921 1.88349 6

8 -9 1 9.11214 2.01115 3

8 -9 1 10.7606 2.18984 1

-9 9 -1 10.0759 2.53436 1

9 -9 1 6.06925 2.12371 1

9 -9 1 1.61172 2.13467 3

9 -9 1 4.13562 1.82333 6

-10 9 -1 4.74504 2.85249 1

10 -9 1 0.93420 2.11788 6

10 -9 1 3.29373 2.35928 1

10 -9 1 -1.3071 2.36089 3

-11 9 -1 -1.0683 2.93297 1

11 -9 1 2.43166 2.39348 6

11 -9 1 -2.9985 2.54090 1

11 -9 1 -3.7702 3.00638 3

-12 9 -1 -0.7437 3.35005 1

12 -9 1 5.29899 3.17395 1

12 -9 1 2.34869 2.98859 6

12 -9 1 -5.3880 3.49349 3

12 8 -1 3.66686 2.23519 1

12 8 -1 1.27742 2.15339 3

-12 -8 1 6.69036 3.21825 5

11 8 -1 0.63115 1.66567 1

11 8 -1 -0.1507 1.55486 3

-11 -8 1 2.00997 2.48039 5

10 8 -1 3.62022 1.54640 3

10 8 -1 1.96237 1.49533 1

-10 -8 1 5.27964 2.18240 5

9 8 -1 36.0367 2.69570 1

-9 -8 1 35.6065 3.52496 5

8 8 -1 41.9353 2.50704 1

-8 -8 1 42.7846 3.13758 5

7 8 -1 12.7279 1.64117 1

-7 -8 1 10.8888 1.78244 5

6 8 -1 -0.1960 0.71889 1

-6 -8 1 0.19664 1.25351 5

-5 -8 1 25.2295 1.97329 2

-5 -8 1 21.4872 2.16029 5

4 8 -1 9.84067 1.41019 6

-4 -8 1 9.92035 1.25022 2

-4 -8 1 8.20107 1.17890 5

3 8 -1 62.0941 2.59442 1

3 8 -1 62.3408 2.43437 6

-3 -8 1 61.0731 2.73033 5

-3 -8 1 62.1339 2.69513 2

2 8 -1 205.054 7.03355 5

2 8 -1 211.392 6.88081 6

2 8 -1 221.013 6.90350 1

2 8 -1 213.395 7.09031 3

2 8 -1 204.777 7.01790 1

-2 -8 1 223.616 7.76198 5

-2 -8 1 208.244 7.10573 2

1 8 -1 31.9896 1.74315 3

1 8 -1 26.5953 1.57490 1

1 8 -1 27.3275 2.18961 1

-1 -8 1 29.5610 1.77212 2

-1 -8 1 28.8253 1.63106 5

0 8 -1 75.0019 2.69535 1

0 8 -1 74.6729 2.92587 3

0 -8 1 68.6975 2.68585 5

-1 8 -1 55.9655 2.41321 3

-1 8 -1 50.7089 2.50597 1

-1 8 -1 53.4400 2.22731 1

1 -8 1 56.2434 2.39314 3

1 -8 1 54.0641 2.09064 5

-2 8 -1 5.21218 0.86609 1

-2 8 -1 6.57429 0.82851 3

-2 8 -1 4.73575 1.13842 1

-2 8 -1 6.19590 1.56679 4

2 -8 1 5.60399 0.95789 3

2 -8 1 8.79635 1.58895 4

2 -8 1 7.15701 1.15680 2

-3 8 -1 264.796 9.07754 4

-3 8 -1 258.431 8.74090 1

-3 8 -1 260.239 8.61713 3

3 -8 1 286.913 9.09547 3

3 -8 1 254.577 8.94522 1

3 -8 1 255.621 8.94658 4

-4 8 -1 157.504 5.78117 4

4 -8 1 141.773 5.58637 1

4 -8 1 148.207 5.50839 3

4 -8 1 143.064 5.03885 6

-5 8 -1 25.5839 3.16949 4

5 -8 1 25.1305 1.90631 6

5 -8 1 30.7141 2.62419 3

5 -8 1 26.1404 2.48115 1

6 -8 1 1.23835 1.25281 1

6 -8 1 0.77463 1.17164 3

7 -8 1 36.8955 3.16843 1

7 -8 1 36.5648 3.37036 3

8 -8 1 5.08496 1.66964 1

8 -8 1 3.84716 1.60978 3

-9 8 -1 7.14859 2.26457 1

9 -8 1 3.23394 1.89591 1

9 -8 1 1.85659 2.05798 3

-10 8 -1 0.75941 2.45159 1

10 -8 1 3.16660 2.12530 1

10 -8 1 -1.0568 2.30404 3

-11 8 -1 2.08410 2.99102 1

11 -8 1 -2.1398 3.26261 3

11 -8 1 1.20495 2.39267 1

-12 8 -1 8.11602 3.32680 1

12 -8 1 3.17812 2.70606 6

12 -8 1 3.22657 3.12694 3

12 -8 1 -0.3404 2.79174 1

13 7 -1 0.40994 2.33934 1

13 7 -1 -0.1864 2.26636 3

-13 -7 1 -1.4142 3.61626 5

12 7 -1 -1.2432 1.79969 3

12 7 -1 1.71128 2.01545 1

-12 -7 1 4.24960 2.97907 5

11 7 -1 8.18144 1.82113 1

-11 -7 1 7.98013 2.84381 5

10 7 -1 -0.9534 1.41242 1

10 7 -1 0.25511 1.27905 3

-10 -7 1 -0.0831 1.87993 5

9 7 -1 3.26637 1.28776 1

9 7 -1 1.06142 1.08530 3

-9 -7 1 2.71207 1.89601 5

8 7 -1 1.72526 1.12777 1

-8 -7 1 2.40109 1.57637 5

7 7 -1 12.0648 1.62429 1

-7 -7 1 11.7858 1.68788 5

6 7 -1 98.7079 3.46080 1

6 7 -1 94.3950 4.13398 4

-6 -7 1 95.8220 3.95681 5

5 7 -1 266.185 8.35933 1

-5 -7 1 243.825 8.55581 5

4 7 -1 540.512 16.7281 1

4 7 -1 534.817 16.9602 6

-4 -7 1 535.249 16.9678 2

-4 -7 1 513.158 16.9600 5

3 7 -1 843.845 25.8442 5

3 7 -1 813.323 25.6304 6

3 7 -1 814.283 25.5501 1

-3 -7 1 790.942 25.7024 2

2 7 -1 1748.47 53.8443 3

2 7 -1 1801.37 53.9135 6

2 7 -1 1712.32 53.8022 5

2 7 -1 1697.50 53.7281 1

2 7 -1 1658.83 53.8098 2

-2 -7 1 1731.36 54.1982 5

1 7 -1 727.334 22.8611 1

1 7 -1 763.490 22.8139 1

1 7 -1 739.011 22.9162 2

1 7 -1 731.466 22.9209 3

-1 -7 1 705.731 22.9523 2

-1 -7 1 700.878 22.8513 5

0 7 -1 1492.31 48.0690 1

0 7 -1 1539.83 48.0844 3

0 7 -1 1532.56 48.0156 1

0 -7 1 1535.56 48.2041 5

0 -7 1 1603.12 48.3567 2

-1 7 -1 60.3189 2.34784 3

-1 7 -1 59.2294 2.91799 1

-1 7 -1 53.0592 2.22591 1

1 -7 1 57.1432 2.36455 3

1 -7 1 57.5662 2.36404 2

-2 7 -1 1.74402 0.58275 3

-2 7 -1 -1.0431 1.15094 4

-2 7 -1 0.36324 0.65147 1

2 -7 1 1.78404 0.72597 3

2 -7 1 1.63065 0.80721 1

2 -7 1 0.87908 1.20784 4

-3 7 -1 -0.1911 1.47315 4

-3 7 -1 1.45763 0.58980 3

3 -7 1 2.39531 0.89936 3

3 -7 1 2.67192 1.42612 4

3 -7 1 -0.5182 0.94866 1

3 -7 1 2.41452 0.70952 6

-4 7 -1 96.1611 3.40411 3

-4 7 -1 91.0374 4.07218 4

4 -7 1 89.7926 4.71261 3

4 -7 1 91.3824 3.37919 6

-5 7 -1 171.138 6.56444 4

5 -7 1 164.242 6.23651 1

5 -7 1 170.086 6.14660 3

6 -7 1 228.248 8.01495 3

6 -7 1 225.883 8.08243 1

7 -7 1 55.8668 3.37225 3

7 -7 1 56.3391 3.33121 1

8 -7 1 18.9693 2.74123 1

8 -7 1 11.2106 1.69295 3

9 -7 1 4.66522 1.74349 3

9 -7 1 4.38728 1.74654 1

10 -7 1 5.85480 1.90289 1

10 -7 1 4.13085 1.93511 3

-11 7 -1 2.42260 2.69572 1

11 -7 1 0.31298 2.13624 1

11 -7 1 -0.3159 2.27222 3

-12 7 -1 1.97722 4.77791 1

12 -7 1 3.30564 2.57098 1

12 -7 1 -2.6364 2.70775 3

13 6 -1 1.38047 2.22586 3

13 6 -1 1.32796 2.39643 1

-13 -6 1 5.21182 3.45247 5

12 6 -1 0.06391 1.86487 3

12 6 -1 1.97067 1.95955 1

-12 -6 1 4.06576 2.73547 5

11 6 -1 2.74975 1.52634 3

11 6 -1 2.71513 1.72186 1

-11 -6 1 3.73633 2.38755 5

10 6 -1 0.53716 1.34077 3

10 6 -1 2.08843 1.45268 1

-10 -6 1 3.29005 2.02260 5

9 6 -1 2.32241 1.13345 3

9 6 -1 3.95225 1.34574 1

-9 -6 1 4.67250 1.74760 5

8 6 -1 13.5566 1.96193 1

-8 -6 1 10.6621 1.69611 5

7 6 -1 1.12132 0.83439 1

-7 -6 1 0.70206 1.29880 5

6 6 -1 208.506 6.57124 1

6 6 -1 198.882 7.09251 4

-6 -6 1 191.598 6.92264 5

5 6 -1 44.9845 2.33103 4

5 6 -1 42.6916 1.81356 1

4 6 -1 893.755 27.8506 1

-4 -6 1 890.142 28.0735 2

3 6 -1 336.219 10.4683 1

3 6 -1 336.115 10.6286 5

3 6 -1 329.260 10.6012 2

-3 -6 1 325.783 10.6779 2

-3 -6 1 321.622 10.6584 5

2 6 -1 949.184 29.0085 5

2 6 -1 929.500 29.0103 2

2 6 -1 953.640 28.9418 6

2 6 -1 918.516 28.9167 1

-2 -6 1 869.659 28.9908 5

-2 -6 1 934.873 29.0436 2

1 6 -1 36.4125 1.57833 3

1 6 -1 36.7480 1.58197 2

1 6 -1 40.6642 1.45115 1

1 6 -1 38.2738 1.54202 5

1 6 -1 35.5690 1.55220 1

1 6 -1 33.1077 1.34768 6

-1 -6 1 31.2276 1.54248 5

-1 -6 1 33.1630 1.62238 2

0 6 -1 1727.68 52.0517 3

0 6 -1 1603.13 52.0540 1

0 6 -1 1700.24 52.0310 1

0 -6 1 1645.47 52.2613 2

0 -6 1 1626.24 52.0205 5

-1 6 -1 912.318 29.4470 4

-1 6 -1 964.759 29.3953 3

-1 6 -1 945.749 29.3790 1

1 -6 1 962.652 29.4622 4

1 -6 1 909.455 29.4075 3

1 -6 1 938.201 29.4214 2

-2 6 -1 376.869 11.6056 1

-2 6 -1 360.769 11.5250 3

-2 6 -1 357.251 11.7391 4

2 -6 1 361.519 11.6327 3

2 -6 1 362.642 11.7285 4

2 -6 1 353.722 11.6882 1

-3 6 -1 126.608 4.32682 1

-3 6 -1 122.551 4.56739 4

-3 6 -1 123.381 4.15409 3

3 -6 1 125.266 4.40001 3

3 -6 1 113.394 4.41740 1

3 -6 1 121.443 4.52285 4

-4 6 -1 10.7083 1.62092 1

-4 6 -1 13.7991 1.23487 3

4 -6 1 12.0786 1.78432 4

4 -6 1 9.95131 1.53553 1

4 -6 1 14.0687 1.72529 3

-5 6 -1 29.7761 2.79910 4

5 -6 1 41.2424 2.37926 3

-6 6 -1 123.996 5.48689 4

6 -6 1 129.573 4.91166 3

6 -6 1 125.033 4.95149 1

7 -6 1 17.6841 2.31766 3

7 -6 1 21.5969 2.15199 1

8 -6 1 82.2583 4.02929 3

8 -6 1 72.0506 3.80303 1

9 -6 1 -0.4181 1.33948 3

9 -6 1 3.35732 1.45735 1

10 -6 1 22.1533 3.19152 1

10 -6 1 25.0672 3.93967 3

-11 6 -1 0.64331 2.32972 1

11 -6 1 -2.7209 2.23798 3

-12 6 -1 3.93030 3.01642 1

12 -6 1 0.16351 2.24959 1

12 -6 1 0.61054 2.65276 3

-13 6 -1 -0.6911 3.28111 1

13 -6 1 -3.1413 2.58964 1

13 -6 1 -7.4544 3.23378 3

13 5 -1 4.37674 2.19032 3

13 5 -1 4.19089 2.47114 1

-13 -5 1 5.92858 3.33085 5

12 5 -1 3.24525 1.61927 3

12 5 -1 2.97873 1.97128 1

-12 -5 1 7.25558 2.66483 5

11 5 -1 0.87466 1.47453 3

11 5 -1 -0.4762 1.61909 1

-11 -5 1 2.65802 2.16081 5

10 5 -1 6.85245 1.60120 1

10 5 -1 4.89916 1.44737 3

-10 -5 1 7.80304 1.81150 5

9 5 -1 67.2651 3.49210 3

9 5 -1 75.2964 3.46652 1

-9 -5 1 73.0245 3.70057 5

8 5 -1 101.131 3.92478 1

8 5 -1 94.5811 4.04518 3

-8 -5 1 102.569 4.23786 5

7 5 -1 79.1927 3.10980 1

-7 -5 1 72.6205 3.41985 5

6 5 -1 11.9560 1.16018 1

6 5 -1 12.4011 1.75778 4

-6 -5 1 10.2502 1.35441 5

5 5 -1 126.338 4.62102 4

5 5 -1 126.722 4.20933 1

-5 -5 1 123.184 4.55186 5

4 5 -1 146.729 4.82848 1

4 5 -1 153.934 5.09111 4

4 5 -1 146.396 5.12815 5

3 5 -1 1948.52 60.8374 5

3 5 -1 1915.17 60.8004 2

-3 -5 1 1901.63 61.0827 5

-3 -5 1 2043.60 61.5917 2

2 5 -1 74.1719 2.58945 5

2 5 -1 69.1950 2.49406 1

2 5 -1 74.6339 2.58981 2

-2 -5 1 69.6874 2.60438 5

-2 -5 1 72.6063 2.60800 2

1 5 -1 799.445 24.7396 5

1 5 -1 802.354 24.7707 2

1 5 -1 780.063 24.6971 1

1 5 -1 828.202 24.6941 1

1 5 -1 807.652 24.7734 3

-1 -5 1 771.647 24.7760 2

-1 -5 1 750.083 24.7310 5

0 5 -1 365.079 11.0944 1

0 5 -1 354.427 11.1002 3

0 5 -1 350.707 11.1225 1

0 -5 1 342.664 11.1124 2

0 -5 1 338.118 11.0198 5

-1 5 -1 2930.88 97.8902 4

-1 5 -1 3312.85 97.9630 1

-1 5 -1 3178.30 97.8436 3

1 -5 1 3110.95 98.1409 1

1 -5 1 3120.55 98.1349 3

1 -5 1 3106.63 97.9487 4

-2 5 -1 242.903 8.09534 4

-2 5 -1 253.879 7.96351 1

-2 5 -1 251.890 7.87752 3

2 -5 1 243.168 8.07927 4

2 -5 1 240.241 7.96386 3

2 -5 1 240.876 8.02240 1

-3 5 -1 2719.42 80.9407 3

-3 5 -1 2484.08 80.4226 1

-3 5 -1 2647.19 81.3679 4

3 -5 1 2505.99 81.0307 3

3 -5 1 2559.09 81.3379 1

3 -5 1 2577.78 80.5431 4

-4 5 -1 65.2060 2.76392 1

-4 5 -1 67.9049 2.53436 3

4 -5 1 65.9141 3.02850 4

4 -5 1 64.8955 2.69612 3

4 -5 1 60.6007 3.00528 1

-5 5 -1 252.448 9.03920 4

5 -5 1 270.414 8.30824 5

5 -5 1 254.116 8.77535 1

-6 5 -1 69.0611 3.93542 4

6 -5 1 80.2800 2.87017 5

6 -5 1 76.9319 3.40331 1

6 -5 1 81.0610 3.34458 3

7 -5 1 233.676 8.02033 3

7 -5 1 224.585 7.61441 5

7 -5 1 235.183 8.11771 1

8 -5 1 8.68343 1.45836 1

8 -5 1 7.17761 1.34515 3

9 -5 1 3.75591 1.31747 3

9 -5 1 4.22260 1.39215 1

10 -5 1 1.11419 1.43276 1

10 -5 1 -0.5800 1.68131 3

11 -5 1 0.61154 1.96605 3

11 -5 1 1.99861 1.82741 1

-12 5 -1 1.83983 2.69981 1

12 -5 1 -2.6625 2.57023 3

12 -5 1 1.54925 2.16367 1

-13 5 -1 0.26957 3.21208 1

13 -5 1 2.41904 2.55425 1

13 4 -1 0.75088 2.37185 1

13 4 -1 -0.8298 2.45097 3

-13 -4 1 -1.8200 2.92606 5

12 4 -1 1.77328 1.93724 1

12 4 -1 0.66903 1.36532 3

-12 -4 1 -0.1301 2.51678 5

11 4 -1 5.10538 1.45414 3

11 4 -1 7.75462 1.81610 1

-11 -4 1 10.6876 2.22671 5

10 4 -1 25.7270 2.75689 1

10 4 -1 18.0218 2.42603 3

-10 -4 1 24.2067 2.86004 5

9 4 -1 7.02745 1.31733 1

9 4 -1 4.46258 1.18707 3

-9 -4 1 8.22787 1.53105 5

8 4 -1 6.00025 1.13108 3

8 4 -1 7.81026 1.20836 1

-8 -4 1 9.26829 1.50997 5

7 4 -1 38.9672 2.14629 1

-7 -4 1 33.8040 2.32345 5

6 4 -1 100.912 3.99556 1

6 4 -1 108.566 4.26583 4

-6 -4 1 114.765 3.86778 1

5 4 -1 841.934 26.9670 1

5 4 -1 866.449 27.2471 4

-5 -4 1 879.041 26.9680 1

4 4 -1 133.531 4.28970 1

4 4 -1 131.311 4.58765 5

4 4 -1 135.684 4.56544 4

4 4 -1 135.634 4.49846 2

-4 -4 1 122.465 4.57006 5

3 4 -1 23.3990 1.27044 5

3 4 -1 23.6655 1.19706 2

-3 -4 1 23.2242 1.23938 2

2 4 -1 39.8167 1.51539 1

2 4 -1 43.7751 1.64114 5

2 4 -1 43.1920 1.61253 2

-2 -4 1 40.8580 1.64646 5

-2 -4 1 39.8282 1.63478 2

1 4 -1 6546.06 201.766 5

1 4 -1 6773.90 201.889 2

-1 -4 1 6094.35 201.744 5

-1 -4 1 6499.19 201.992 2

0 4 -1 146.333 4.44932 1

0 4 -1 141.238 4.49740 3

0 -4 1 134.454 4.47560 3

0 -4 1 130.826 4.41574 5

0 -4 1 136.670 4.50731 2

-1 4 -1 1497.59 49.8820 4

-1 4 -1 1568.67 49.6371 3

-1 4 -1 1659.03 49.7247 1

1 -4 1 1572.45 49.7124 4

1 -4 1 1556.60 49.8559 3

1 -4 1 1651.97 49.9718 1

-2 4 -1 -0.9976 0.80922 4

-2 4 -1 0.54241 0.44216 1

-2 4 -1 1.11190 0.34688 3

2 -4 1 1.29284 0.50388 1

2 -4 1 0.79035 0.45568 3

2 -4 1 -0.9014 0.78484 4

-3 4 -1 271.394 8.36935 3

-3 4 -1 276.786 8.51013 1

-3 4 -1 252.503 8.64090 4

3 -4 1 247.685 8.39465 3

3 -4 1 249.712 8.50747 1

3 -4 1 263.036 8.60952 4

-4 4 -1 413.021 12.6932 3

-4 4 -1 417.458 12.8243 1

-4 4 -1 402.442 13.0231 4

4 -4 1 397.857 12.9421 4

4 -4 1 375.548 12.6932 3

4 -4 1 375.794 12.8128 1

-5 4 -1 816.487 25.6292 4

-5 4 -1 791.588 25.2493 3

5 -4 1 796.714 25.3973 1

5 -4 1 855.885 25.0720 5

5 -4 1 738.902 25.2531 3

-6 4 -1 2.02309 2.04274 4

6 -4 1 3.30666 0.80936 5

6 -4 1 3.75769 1.05246 1

6 -4 1 3.19030 0.96123 3

7 -4 1 4.18216 0.95349 5

7 -4 1 3.89599 0.96773 3

7 -4 1 1.37457 1.11796 1

8 -4 1 38.1704 2.70484 3

8 -4 1 37.9218 2.57682 1

9 -4 1 9.73127 1.42824 3

9 -4 1 9.66429 1.51347 1

10 -4 1 0.66781 1.47940 3

10 -4 1 1.57480 1.46995 1

11 -4 1 3.69907 1.67279 1

11 -4 1 0.94963 1.86907 3

-12 4 -1 3.31486 2.40794 1

12 -4 1 -1.8028 2.18641 3

12 -4 1 -0.0922 1.91057 1

-13 4 -1 -0.1097 3.01774 1

13 -4 1 -0.2887 2.70573 3

13 -4 1 -1.9551 2.29260 1

13 3 -1 2.06453 2.58048 3

13 3 -1 0.90324 2.27113 1

12 3 -1 1.39588 1.90705 1

12 3 -1 1.39102 2.18554 3

-12 -3 1 -1.2653 2.57745 5

11 3 -1 -1.2812 1.16289 3

11 3 -1 0.28301 1.58607 1

-11 -3 1 -0.1109 1.79506 5

10 3 -1 57.5225 3.43052 3

10 3 -1 72.3041 3.62513 1

-10 -3 1 65.1062 3.58381 5

9 3 -1 71.3252 3.32539 1

9 3 -1 63.5123 3.30651 3

-9 -3 1 71.0689 3.32146 5

8 3 -1 2.05186 1.00805 1

8 3 -1 0.78925 0.90663 3

-8 -3 1 -0.5606 1.17768 5

7 3 -1 -0.3440 0.76987 3

7 3 -1 -0.3073 0.87708 1

-7 -3 1 2.01285 1.05805 5

6 3 -1 653.137 20.0878 4

6 3 -1 579.485 19.6994 1

-6 -3 1 619.216 19.6660 5

-6 -3 1 616.450 19.4586 1

5 3 -1 264.449 8.70098 1

5 3 -1 266.224 8.96049 4

5 3 -1 284.186 8.96572 2

-5 -3 1 270.606 8.68676 1

4 3 -1 62.9983 2.55439 5

4 3 -1 69.7423 2.46941 2

4 3 -1 65.3535 2.59810 4

4 3 -1 68.4354 2.30580 1

-4 -3 1 58.6362 2.52667 5

-4 -3 1 70.2496 2.33434 1

3 3 -1 722.750 22.3650 2

3 3 -1 728.646 22.3612 1

3 3 -1 719.992 22.4074 5

-3 -3 1 707.317 22.3660 2

-3 -3 1 679.247 22.3933 5

-3 -3 1 724.369 22.2347 1

2 3 -1 153.813 4.79569 2

2 3 -1 150.206 4.80421 5

-2 -3 1 147.342 4.79840 2

-2 -3 1 138.069 4.79478 5

1 3 -1 55.7925 1.88815 5

1 3 -1 54.4553 1.80820 1

1 3 -1 57.1184 1.90905 2

-1 -3 1 50.6676 1.88275 5

-1 -3 1 55.1904 1.92204 2

0 3 -1 3468.16 109.688 5

0 3 -1 3776.79 109.932 1

0 -3 1 3516.82 109.902 4

0 -3 1 3359.82 109.670 5

0 -3 1 3437.14 109.935 3

0 -3 1 3428.00 110.001 2

0 -3 1 3676.79 109.915 1

-1 3 -1 383.813 11.4613 1

-1 3 -1 353.065 11.5074 4

-1 3 -1 366.975 11.4001 3

1 -3 1 345.548 11.4205 3

1 -3 1 365.811 11.5218 4

1 -3 1 364.187 11.4722 1

-2 3 -1 340.372 10.2910 1

-2 3 -1 317.808 10.3639 4

-2 3 -1 338.500 10.1876 3

2 -3 1 305.961 10.1944 3

2 -3 1 321.889 10.3531 4

2 -3 1 308.567 10.2794 1

-3 3 -1 290.828 8.97419 3

-3 3 -1 274.709 9.18725 4

-3 3 -1 296.381 9.09010 1

3 -3 1 256.593 8.94461 3

3 -3 1 286.703 9.17353 4

3 -3 1 276.724 9.07980 1

-4 3 -1 3077.71 98.2575 3

-4 3 -1 3221.94 98.5622 1

-4 3 -1 3115.80 98.8261 4

4 -3 1 3049.54 98.4466 4

4 -3 1 3382.06 98.2400 5

4 -3 1 3014.85 98.5961 3

4 -3 1 3137.15 99.0750 1

-5 3 -1 160.723 6.43613 4

5 -3 1 175.576 5.57515 5

-6 3 -1 13.0588 2.20040 4

6 -3 1 10.6620 1.58283 3

6 -3 1 9.08295 1.26474 1

6 -3 1 12.2898 1.36784 5

7 -3 1 -0.8243 0.84937 3

7 -3 1 -0.3331 0.87906 1

8 -3 1 87.0502 3.82115 3

8 -3 1 93.8811 4.01357 1

9 -3 1 -1.2013 1.15318 3

9 -3 1 0.93331 1.26224 1

10 -3 1 22.1827 3.08600 3

10 -3 1 17.3509 2.51327 1

11 -3 1 0.48582 1.99030 3

11 -3 1 2.39044 1.66464 1

12 -3 1 0.43486 2.49149 3

12 -3 1 0.71208 1.81533 1

-13 3 -1 3.17081 5.37065 1

13 -3 1 -2.7885 3.02470 3

13 -3 1 1.76814 2.29340 1

13 2 -1 2.99065 2.62730 3

13 2 -1 1.92370 2.27681 1

12 2 -1 -2.0732 2.71413 3

12 2 -1 -0.9886 1.89609 1

-12 -2 1 1.42085 2.23586 5

11 2 -1 1.60903 1.52477 1

11 2 -1 0.78127 1.23280 3

-11 -2 1 3.88117 1.80539 5

10 2 -1 0.33431 1.32377 1

10 2 -1 0.84858 0.96937 3

-10 -2 1 0.64832 1.41119 5

9 2 -1 3.55411 1.27547 1

9 2 -1 3.98380 1.12125 3

-9 -2 1 5.51795 1.40143 5

8 2 -1 75.1996 3.21891 1

8 2 -1 71.7019 3.32274 3

-8 -2 1 74.1355 3.26290 5

7 2 -1 325.347 10.4165 1

7 2 -1 303.319 10.4433 3

-7 -2 1 321.606 10.4415 5

6 2 -1 141.639 5.07319 3

6 2 -1 144.324 5.02418 1

-6 -2 1 151.296 5.03300 1

-6 -2 1 143.165 5.11437 5

5 2 -1 102.140 3.57630 2

5 2 -1 101.166 3.81840 4

5 2 -1 97.8520 3.53352 1

5 2 -1 102.175 3.76189 3

5 2 -1 97.5853 3.66275 5

-5 -2 1 98.0313 3.43852 1

-5 -2 1 99.3917 3.90511 5

4 2 -1 419.923 13.6603 5

4 2 -1 437.880 13.7313 4

4 2 -1 452.381 13.6161 2

4 2 -1 417.047 13.5355 1

-4 -2 1 420.449 13.5000 1

3 2 -1 71.7979 2.33890 1

3 2 -1 68.5679 2.49357 5

3 2 -1 72.6750 2.43124 2

-3 -2 1 68.8274 2.32400 1

-3 -2 1 64.6611 2.48815 5

2 2 -1 862.019 26.3512 2

2 2 -1 831.522 26.3732 5

2 2 -1 830.070 26.4189 1

-2 -2 1 862.335 26.4917 2

-2 -2 1 839.170 26.6557 5

1 2 -1 11.3684 0.54106 2

1 2 -1 11.2107 0.54692 5

-1 -2 1 10.7828 0.55887 2

0 2 -1 8.68858 0.37034 3

0 -2 1 7.42546 0.35386 1

0 -2 1 7.02267 0.35391 3

0 -2 1 6.46182 0.39825 2

0 -2 1 6.51722 0.48973 4

-1 2 -1 2566.33 76.7628 3

-1 2 -1 2459.33 77.1477 4

1 -2 1 2423.86 77.1923 1

1 -2 1 2376.85 76.7872 3

1 -2 1 2468.91 77.1336 4

-2 2 -1 9999.99 311.888 4

-3 2 -1 163.758 5.57115 1

-3 2 -1 163.379 5.37928 5

-3 2 -1 178.167 5.52406 3

-3 2 -1 161.737 5.70140 4

3 -2 1 174.710 5.41407 5

3 -2 1 166.827 5.49413 3

3 -2 1 172.825 5.71423 4

3 -2 1 167.603 5.60275 1

-4 2 -1 159.951 5.50252 1

-4 2 -1 157.050 5.71406 4

-4 2 -1 154.213 5.28264 5

4 -2 1 166.377 5.54079 1

4 -2 1 177.899 5.35478 5

4 -2 1 165.667 5.70625 4

-5 2 -1 42.4343 1.78991 5

-5 2 -1 43.7146 2.04516 1

5 -2 1 39.8096 2.42296 4

5 -2 1 49.6016 1.90956 5

5 -2 1 40.1864 2.11826 3

-6 2 -1 264.474 8.59681 5

-6 2 -1 259.229 9.22566 4

6 -2 1 286.219 8.69468 5

6 -2 1 266.353 8.86706 1

6 -2 1 250.685 8.73863 3

-7 2 -1 5.64257 0.99115 5

7 -2 1 5.20964 1.09884 1

7 -2 1 5.69898 1.00251 3

-8 2 -1 1.33093 0.99382 5

8 -2 1 0.21355 0.87530 3

8 -2 1 1.14454 1.08282 1

9 -2 1 6.07351 1.23883 3

9 -2 1 6.85269 1.40847 1

10 -2 1 0.07037 1.37810 1

10 -2 1 -1.3434 1.20391 3

11 -2 1 0.56514 1.45035 1

11 -2 1 0.27551 1.58569 3

12 -2 1 0.88829 1.81461 1

12 -2 1 -2.8922 3.77840 3

13 -2 1 -1.2069 3.10171 3

13 -2 1 1.08155 2.18624 1

14 1 -1 0.09895 2.79952 1

14 1 -1 -1.9375 3.03514 3

13 1 -1 4.40527 2.29723 1

13 1 -1 -0.2115 2.41040 3

12 1 -1 2.48840 1.87578 1

11 1 -1 4.85428 1.67176 3

11 1 -1 3.69514 1.57158 1

-11 -1 1 5.71821 1.94898 5

10 1 -1 23.4993 2.60261 1

10 1 -1 15.1593 2.22500 3

-10 -1 1 21.1650 2.34191 5

9 1 -1 6.83085 1.18773 3

9 1 -1 9.65866 1.44391 1

-9 -1 1 9.35378 1.50738 5

8 1 -1 24.4851 2.12700 1

8 1 -1 19.2143 1.98288 3

-8 -1 1 19.9200 1.86546 5

7 1 -1 305.240 10.3703 3

7 1 -1 318.272 10.3181 1

-7 -1 1 317.166 10.2960 5

6 1 -1 17.2799 1.48646 3

6 1 -1 15.1731 1.45893 1

-6 -1 1 17.3721 1.41689 5

5 1 -1 48.0617 2.05101 1

5 1 -1 46.8419 2.31834 4

5 1 -1 47.5050 2.09277 5

-5 -1 1 46.3131 2.04736 5

-5 -1 1 48.0317 1.94881 1

4 1 -1 396.044 12.6224 4

4 1 -1 391.932 12.4865 1

4 1 -1 390.557 12.5184 5

-4 -1 1 392.973 12.5126 5

-4 -1 1 396.146 12.4227 1

3 1 -1 1422.02 42.9249 4

3 1 -1 1342.90 42.7429 1

-3 -1 1 1375.05 42.6643 1

-3 -1 1 1307.47 42.9721 5

2 1 -1 407.288 12.2321 2

2 1 -1 376.757 12.2579 5

2 1 -1 393.045 12.2696 1

-2 -1 1 362.018 12.2559 5

-2 -1 1 411.869 12.2687 1

1 1 -1 117.333 3.78439 5

1 1 -1 124.103 3.77399 2

-1 -1 1 123.863 3.77332 2

-1 -1 1 110.853 3.78430 5

-1 1 -1 76.2408 2.44607 1

-1 1 -1 75.6314 2.50369 4

-1 1 -1 77.4524 2.39981 3

1 -1 1 76.0970 2.39550 3

1 -1 1 66.9292 2.54511 4

1 -1 1 74.7410 2.44620 1

-2 1 -1 969.144 31.3961 4

-2 1 -1 972.488 30.9955 5

-2 1 -1 981.618 31.1158 1

2 -1 1 997.026 31.1289 3

2 -1 1 1013.33 31.4532 1

2 -1 1 990.804 31.3459 4

-3 1 -1 448.630 14.5637 1

-3 1 -1 446.758 14.4630 5

-3 1 -1 453.753 14.6971 4

3 -1 1 472.312 14.5389 3

3 -1 1 469.803 14.5990 1

3 -1 1 452.848 14.4811 5

3 -1 1 485.094 14.7184 4

-4 1 -1 23.1482 1.29683 1

-4 1 -1 21.0916 1.17053 5

-4 1 -1 22.1890 1.63265 4

4 -1 1 26.9270 1.34615 1

4 -1 1 25.2021 1.57072 4

4 -1 1 22.1099 1.20246 5

4 -1 1 20.8123 1.24495 3

-5 1 -1 136.580 5.39058 4

-5 1 -1 138.210 4.56978 5

-5 1 -1 130.297 4.75312 1

5 -1 1 135.057 5.01956 4

5 -1 1 143.444 4.64444 5

5 -1 1 139.121 4.81282 1

5 -1 1 128.153 4.84379 3

6 -1 1 3.43404 0.89280 1

6 -1 1 5.27682 1.05572 5

6 -1 1 3.15123 0.75411 3

-7 1 -1 146.001 4.85449 5

7 -1 1 135.883 5.08546 1

7 -1 1 132.058 4.96625 3

-8 1 -1 90.9126 3.51834 5

8 -1 1 90.9995 3.76690 1

8 -1 1 84.6221 3.64358 3

-9 1 -1 25.7335 2.06170 5

9 -1 1 19.9706 2.10068 3

9 -1 1 21.6611 2.53116 1

-10 1 -1 16.9568 2.40368 5

10 -1 1 12.7120 1.72719 3

10 -1 1 21.8198 2.72189 1

11 -1 1 30.3395 3.53857 3

11 -1 1 26.9058 2.87110 1

12 -1 1 0.12076 1.85113 1

12 -1 1 -0.0470 3.84315 3

13 -1 1 -1.8250 2.46139 3

13 -1 1 -0.6648 2.13668 1

14 0 -1 0.61758 3.12682 3

14 0 -1 1.85213 2.93574 1

13 0 -1 3.85345 2.27083 1

13 0 -1 -2.4001 2.47537 3

12 0 -1 -0.3191 1.75672 1

12 0 -1 1.89542 3.97847 3

11 0 -1 -1.3053 1.43697 1

11 0 -1 -0.0263 1.40778 3

10 0 -1 0.88771 1.27514 1

10 0 -1 0.86405 1.16987 3

-10 0 1 0.40636 1.45472 5

9 0 -1 -0.4173 1.00334 3

9 0 -1 -0.9922 1.09060 1

-9 0 1 -1.2586 1.17990 5

8 0 -1 1.08370 1.03395 1

8 0 -1 0.07052 0.76004 3

-8 0 1 -0.3287 1.01255 5

7 0 -1 -1.0309 0.66070 3

7 0 -1 1.93323 0.92282 1

-7 0 1 0.13600 0.83227 5

6 0 -1 0.78728 0.71268 3

6 0 -1 -1.5446 0.74507 1

5 0 -1 1.18153 0.62377 3

5 0 -1 -0.0618 1.07554 4

5 0 -1 3.06329 0.76572 5

-5 0 1 -0.2319 0.63045 5

4 0 -1 3.22304 0.70373 5

4 0 -1 4.10407 1.06819 4

4 0 -1 2.58205 0.61209 3

-4 0 1 5.57632 0.86702 1

3 0 -1 -0.0134 0.58282 4

3 0 -1 0.25564 0.40094 5

3 0 -1 0.27207 0.37608 3

-3 0 1 1.18052 0.38423 1

-3 0 1 0.68225 0.38817 5

2 0 -1 1.33631 0.28788 1

2 0 -1 1.39798 0.27438 3

-2 0 1 1.11402 0.24863 3

-2 0 1 1.13262 0.32465 5

1 0 -1 2.03615 0.17551 1

-1 0 1 1.59825 0.17840 1

-1 0 1 2.14260 0.19980 1

-1 0 -1 1.54913 0.19402 1

-1 0 -1 1.20143 0.15253 5

-1 0 -1 2.03697 0.30981 4

1 0 1 1.16605 0.17612 5

1 0 1 1.28526 0.27683 4

1 0 1 1.07087 0.14256 1

-2 0 -1 2.45418 0.58576 4

-2 0 -1 2.48775 0.33882 5

-2 0 -1 1.94789 0.32700 1

2 0 1 3.84875 0.64107 4

2 0 1 2.19235 0.37309 5

2 0 1 1.68194 0.31427 1

-3 0 -1 0.52474 0.40784 5

-3 0 -1 0.17059 0.73188 4

3 0 1 0.64867 0.38730 1

3 0 1 -0.0418 0.71465 4

3 0 1 0.75434 0.47402 5

-4 0 -1 0.13142 0.55458 1

-4 0 -1 2.10567 1.14805 4

-4 0 -1 0.56565 0.61796 5

4 0 1 1.30172 0.71095 5

4 0 1 0.58571 0.55370 1

4 0 1 1.35587 0.45072 3

-5 0 -1 1.68663 1.19569 4

-5 0 -1 0.24226 0.68215 1

-5 0 -1 0.95927 0.65844 5

5 0 1 2.57292 1.23171 4

5 0 1 1.35628 0.75683 5

6 0 1 0.90212 0.70681 3

6 0 1 1.69219 0.84032 5

6 0 1 0.81945 0.80028 1

-7 0 -1 -0.6517 0.88750 5

7 0 1 -0.8870 0.75560 3

7 0 1 -0.7844 0.86809 1

-8 0 -1 2.38865 1.10671 5

8 0 1 2.07729 0.93147 3

8 0 1 1.32772 1.06909 1

-9 0 -1 -0.3449 1.17275 5

9 0 1 -0.2266 1.11937 1

9 0 1 -0.8691 0.83729 3

-10 0 -1 0.60406 1.42781 5

10 0 1 1.22639 1.30683 3

10 0 1 0.85830 1.42028 1

-11 0 -1 -1.9245 1.70937 5

11 0 1 -0.5974 1.59275 1

11 0 1 -1.9435 1.51429 3

12 0 1 3.47740 3.35154 3

13 0 1 -3.3508 2.36594 3

13 0 1 -1.6763 2.30074 1

14 -1 -1 2.92448 2.83657 1

14 -1 -1 -2.0363 3.28509 3

13 -1 -1 3.92661 2.63513 3

13 -1 -1 2.76348 2.37225 1

12 -1 -1 1.39247 2.90436 1

12 -1 -1 0.39148 3.45159 3

-12 1 1 0.69801 2.02518 1

11 -1 -1 4.39788 1.62474 1

11 -1 -1 3.14327 1.61276 3

-11 1 1 2.96619 1.71733 1

10 -1 -1 13.0894 1.57729 3

10 -1 -1 17.6150 2.29454 1

9 -1 -1 5.40287 1.22799 3

9 -1 -1 6.18860 1.31229 1

8 -1 -1 13.1740 1.81661 3

8 -1 -1 16.7499 1.97110 1

-8 1 1 14.5094 1.71429 5

7 -1 -1 275.752 9.48544 3

7 -1 -1 294.530 9.57761 1

-7 1 1 286.710 9.36810 5

6 -1 -1 18.3746 1.47566 5

6 -1 -1 17.3918 1.49464 3

6 -1 -1 20.5530 1.57820 1

-6 1 1 19.5773 1.38611 5

5 -1 -1 67.3821 2.35410 5

5 -1 -1 54.9347 2.61812 3

5 -1 -1 55.5726 2.69735 4

-5 1 1 57.4716 2.42378 1

-5 1 1 58.4006 2.28854 5

4 -1 -1 401.294 13.6895 1

4 -1 -1 423.769 13.6809 3

4 -1 -1 450.171 13.6236 5

4 -1 -1 439.411 13.8029 4

-4 1 1 416.875 13.5936 5

-4 1 1 442.938 13.6890 3

-4 1 1 437.434 13.6840 1

3 -1 -1 1348.77 42.3294 4

3 -1 -1 1287.22 42.3146 1

3 -1 -1 1394.40 42.2571 5

3 -1 -1 1318.17 42.4731 3

-3 1 1 1389.04 42.2738 3

-3 1 1 1321.08 41.9651 1

-3 1 1 1318.97 42.0758 5

2 -1 -1 406.715 13.1114 1

2 -1 -1 420.436 13.1590 4

2 -1 -1 421.554 13.0529 5

2 -1 -1 396.707 13.0981 3

-2 1 1 435.251 13.1064 1

-2 1 1 421.714 13.1012 3

1 -1 -1 111.025 3.45734 1

1 -1 -1 102.026 3.45211 3

-1 1 1 108.184 3.45023 1

-1 1 1 112.840 3.44902 1

-1 1 1 105.654 3.44769 3

-1 -1 -1 61.7179 1.96262 4

-1 -1 -1 60.1460 1.94644 5

-1 -1 -1 60.0473 1.94866 1

1 1 1 63.5473 1.98303 6

1 1 1 56.0272 1.93893 1

1 1 1 51.6379 1.96678 5

1 1 1 62.0744 1.96978 4

-2 -1 -1 1044.30 33.0048 1

-2 -1 -1 1041.83 33.0171 5

-2 -1 -1 1078.50 33.2196 4

2 1 1 1041.83 32.9650 5

2 1 1 1009.43 33.0082 1

2 1 1 1090.58 33.1614 4

-3 -1 -1 472.090 14.8094 4

-3 -1 -1 448.202 14.6826 5

-3 -1 -1 478.359 14.6759 1

3 1 1 461.195 14.7125 5

3 1 1 450.018 14.6549 1

3 1 1 491.831 14.8318 4

-4 -1 -1 31.4938 1.38815 5

-4 -1 -1 28.7451 1.69567 4

-4 -1 -1 30.1397 1.36231 1

4 1 1 28.6471 1.35583 1

4 1 1 31.1362 1.45342 5

-5 -1 -1 148.351 4.95130 5

5 1 1 144.793 5.24842 4

5 1 1 143.703 4.99667 5

-6 -1 -1 7.04704 0.99477 5

6 1 1 4.85818 0.83099 3

6 1 1 5.54614 0.96686 1

-7 -1 -1 158.346 5.38185 5

7 1 1 140.675 5.34871 3

7 1 1 155.572 5.44241 1

-8 -1 -1 97.5046 3.74642 5

8 1 1 91.9950 3.81804 3

8 1 1 93.2181 3.90564 1

-9 -1 -1 18.5487 2.15326 5

9 1 1 16.8138 2.07636 3

9 1 1 19.7153 2.29846 1

-10 -1 -1 24.7737 2.52135 5

10 1 1 16.6338 1.95977 1

10 1 1 15.8249 2.15519 3

-11 -1 -1 31.1392 3.13841 5

11 1 1 21.2176 2.66301 3

11 1 1 33.0020 3.29535 1

-12 -1 -1 1.30211 2.27577 5

12 1 1 0.71887 2.10950 3

13 1 1 1.69924 2.66587 3

13 1 1 3.16611 2.24860 1

13 -2 -1 -0.6077 2.27165 1

13 -2 -1 -1.7617 2.86287 3

12 -2 -1 1.38659 3.47122 1

-12 2 1 0.03221 2.13955 1

11 -2 -1 3.82348 1.56089 1

11 -2 -1 5.66508 1.92326 3

-11 2 1 5.47449 1.94945 1

10 -2 -1 3.62557 1.35695 1

10 -2 -1 1.41691 1.36027 3

9 -2 -1 3.18525 1.22703 3

9 -2 -1 1.94138 1.23754 1

8 -2 -1 73.6224 3.37060 3

8 -2 -1 76.0922 3.40476 1

7 -2 -1 352.970 11.4241 1

7 -2 -1 334.453 11.2997 3

6 -2 -1 150.941 5.07327 5

6 -2 -1 148.609 5.18719 3

6 -2 -1 141.956 5.26069 1

5 -2 -1 67.1498 2.85648 1

5 -2 -1 74.3477 2.63113 5

5 -2 -1 69.6793 3.07097 4

-5 2 1 70.7391 2.85965 1

4 -2 -1 446.064 14.6726 3

4 -2 -1 471.420 14.8203 4

4 -2 -1 475.658 14.5801 5

4 -2 -1 425.606 14.7147 1

-4 2 1 471.208 14.6814 3

-4 2 1 483.051 14.7323 1

3 -2 -1 104.257 3.24539 5

3 -2 -1 91.5560 3.40701 1

3 -2 -1 101.415 3.38028 3

3 -2 -1 92.4693 3.46312 4

-3 2 1 97.0592 3.36995 1

-3 2 1 99.8518 3.34076 3

2 -2 -1 875.082 26.1798 1

2 -2 -1 808.429 25.8841 4

2 -2 -1 762.938 25.6968 3

-2 2 1 837.137 25.9494 3

1 -2 -1 14.6232 0.60806 6

1 -2 -1 14.3929 0.66098 1

1 -2 -1 12.8971 0.64250 4

1 -2 -1 14.8847 0.71089 3

-1 2 1 13.0203 0.66624 3

-1 2 1 12.4746 0.66989 1

-1 2 1 11.7740 0.64662 1

0 -2 -1 9.36091 0.50765 1

0 -2 -1 9.97015 0.46773 6

0 -2 -1 9.79904 0.49679 3

0 -2 -1 10.0491 0.40569 2

0 2 1 9.47227 0.47871 6

0 2 1 8.07064 0.49128 1

0 2 1 8.54466 0.39587 2

-1 -2 -1 2554.41 79.9416 5

-1 -2 -1 2541.00 79.7066 1

-1 -2 -1 2487.73 79.7551 2

1 2 1 2619.57 80.3642 6

1 2 1 2642.32 80.0187 5

1 2 1 2467.24 79.7845 1

1 2 1 2579.22 79.7339 2

-3 -2 -1 163.033 5.21713 1

-3 -2 -1 157.843 5.25259 5

3 2 1 165.471 5.29890 5

3 2 1 168.180 5.35597 4

3 2 1 149.606 5.17981 1

-4 -2 -1 171.259 5.60168 1

-4 -2 -1 163.849 5.64394 5

4 2 1 171.017 5.70934 5

4 2 1 172.370 5.57697 1

4 2 1 178.041 5.82297 4

-5 -2 -1 36.2681 1.61077 1

5 2 1 28.0739 2.07087 4

5 2 1 26.5170 1.78021 5

-6 -2 -1 266.043 8.67941 5

6 2 1 260.281 8.61149 1

-7 -2 -1 7.10013 1.20502 5

7 2 1 3.72843 0.92452 3

7 2 1 3.91849 1.10416 1

-8 -2 -1 -0.3108 1.18351 5

8 2 1 -0.5873 0.74544 3

8 2 1 0.45247 1.00871 1

-9 -2 -1 12.0788 1.65008 5

9 2 1 9.04442 1.47657 1

9 2 1 7.77961 1.17653 3

-10 -2 -1 -0.4384 1.58378 5

10 2 1 0.82807 1.29338 1

10 2 1 -1.2853 1.09072 3

-11 -2 -1 -1.6828 1.72431 5

11 2 1 0.50500 1.23660 3

11 2 1 2.19532 1.69123 1

-12 -2 -1 2.69867 2.30979 5

12 2 1 -1.6784 2.07846 3

-13 -2 -1 1.23348 2.78550 5

13 2 1 -0.4042 2.60209 3

13 2 1 -1.1895 2.29796 1

13 -3 -1 4.72781 2.45440 1

13 -3 -1 1.95500 2.63364 3

12 -3 -1 -0.9635 1.86524 1

12 -3 -1 -0.8796 3.73432 3

-12 3 1 3.77002 2.35833 1

11 -3 -1 -0.9772 1.51563 1

11 -3 -1 -2.6591 1.78750 3

-11 3 1 2.88505 2.06196 1

10 -3 -1 62.5809 3.83351 3

10 -3 -1 51.3382 3.31783 1

9 -3 -1 56.6908 3.35621 3

9 -3 -1 59.7478 3.18622 1

8 -3 -1 2.41834 1.14162 1

8 -3 -1 1.53053 1.10129 3

7 -3 -1 0.67852 0.94957 1

7 -3 -1 -0.9146 0.80459 3

6 -3 -1 518.775 17.8247 1

6 -3 -1 558.036 17.7579 3

6 -3 -1 585.881 17.5877 5

5 -3 -1 242.161 8.32365 3

5 -3 -1 258.559 8.72357 4

5 -3 -1 261.576 8.13510 5

-5 3 1 243.887 8.25201 3

4 -3 -1 62.6234 2.80478 4

4 -3 -1 64.6501 2.61115 3

4 -3 -1 72.1398 2.39808 5

4 -3 -1 63.5066 2.67563 1

-4 3 1 72.0274 2.61838 1

-4 3 1 71.3006 2.56007 3

3 -3 -1 687.871 20.8633 5

3 -3 -1 682.588 21.0851 4

3 -3 -1 644.227 20.9942 3

3 -3 -1 644.617 21.0475 1

-3 3 1 678.185 20.9650 3

2 -3 -1 134.487 4.48507 1

2 -3 -1 130.879 4.49165 3

2 -3 -1 132.874 4.48689 4

-2 3 1 137.579 4.42710 3

-2 3 1 137.060 4.42502 1

1 -3 -1 72.8132 2.53619 6

1 -3 -1 72.4096 2.65917 3

-1 3 1 71.8323 2.59880 1

-1 3 1 79.3048 2.57376 1

-1 3 1 76.3391 2.62588 3

-1 3 1 83.5095 2.50102 6

0 -3 -1 3628.75 116.870 3

0 -3 -1 3655.87 116.528 2

0 -3 -1 3678.49 116.535 6

0 -3 -1 3841.47 116.658 1

0 3 1 3835.52 116.499 2

0 3 1 3826.86 116.784 1

0 3 1 3759.95 116.522 6

0 3 1 3681.20 116.892 3

-1 -3 -1 398.622 12.2442 6

-1 -3 -1 402.364 12.2242 1

-1 -3 -1 384.478 12.2516 5

-1 -3 -1 378.153 12.2171 2

1 3 1 398.424 12.2639 5

1 3 1 371.452 12.2409 1

1 3 1 396.191 12.2444 6

1 3 1 386.842 12.2152 2

-2 -3 -1 337.695 10.6814 1

-2 -3 -1 338.496 10.7368 5

-2 -3 -1 331.955 10.6998 2

2 3 1 346.638 10.7508 5

2 3 1 330.694 10.6939 1

2 3 1 348.075 10.7025 2

-3 -3 -1 278.454 9.34572 5

-3 -3 -1 298.341 9.29747 1

3 3 1 309.450 9.40135 4

3 3 1 280.118 9.26883 1

3 3 1 299.104 9.32763 2

3 3 1 290.081 9.38046 5

-4 -3 -1 3269.31 105.714 5

-4 -3 -1 3512.74 105.215 1

4 3 1 3440.99 105.401 4

4 3 1 3539.60 105.452 2

4 3 1 3199.82 105.255 5

4 3 1 3260.04 105.307 1

-5 -3 -1 194.673 6.41944 1

5 3 1 193.971 6.72212 4

-6 -3 -1 8.85137 1.19818 5

6 3 1 9.93011 1.33321 1

6 3 1 12.4552 1.97394 4

-7 -3 -1 -0.3248 0.97918 5

7 3 1 -1.1762 0.79542 1

-8 -3 -1 97.6648 4.01840 5

8 3 1 103.272 4.02467 1

8 3 1 91.9522 3.92587 3

-9 -3 -1 0.02493 1.28859 5

9 3 1 0.55883 0.84345 3

9 3 1 -0.1081 1.22444 1

-10 -3 -1 25.9889 2.81802 5

10 3 1 24.6869 2.58603 3

10 3 1 26.1279 2.80255 1

-11 -3 -1 3.66945 2.03983 5

11 3 1 3.30308 1.34779 3

11 3 1 4.57585 1.83417 1

-12 -3 -1 -0.5712 2.87274 5

12 3 1 -0.2629 1.38266 3

-13 -3 -1 -1.5587 2.93956 5

13 3 1 1.26498 2.29526 1

13 3 1 -4.0067 2.80092 3

13 -4 -1 1.50047 2.51786 1

13 -4 -1 -1.5959 2.80312 3

-13 4 1 4.90591 3.62413 1

12 -4 -1 -1.5836 2.25300 3

12 -4 -1 1.36609 1.98222 1

-12 4 1 -1.7788 2.34902 1

11 -4 -1 7.32973 1.88190 1

11 -4 -1 7.71103 1.92173 3

-11 4 1 6.12449 2.25839 1

10 -4 -1 19.9692 2.82275 1

10 -4 -1 25.8109 3.37119 3

-10 4 1 25.9526 3.59789 1

9 -4 -1 5.28413 1.41876 1

9 -4 -1 5.50946 1.42552 3

8 -4 -1 4.53691 1.16605 3

8 -4 -1 4.83812 1.24032 1

7 -4 -1 43.4136 2.57747 1

7 -4 -1 45.8517 2.61267 3

6 -4 -1 117.991 3.92475 5

6 -4 -1 108.784 4.24275 3

6 -4 -1 103.678 4.29781 1

5 -4 -1 834.280 24.4235 5

5 -4 -1 721.447 24.7078 1

4 -4 -1 158.882 4.97179 5

4 -4 -1 144.617 5.21103 3

4 -4 -1 147.012 5.34380 4

4 -4 -1 144.740 5.29325 1

-4 4 1 161.707 5.15488 3

-4 4 1 154.894 5.20617 1

3 -4 -1 31.2411 1.71938 4

3 -4 -1 34.9474 1.72691 3

3 -4 -1 35.1330 1.68560 1

-3 4 1 33.9786 1.47033 3

-3 4 1 35.8226 1.52891 1

2 -4 -1 29.2627 1.42461 4

2 -4 -1 31.0522 1.43156 1

2 -4 -1 30.2264 1.53911 3

2 -4 -1 30.7732 1.26020 6

-2 4 1 32.4597 1.35585 3

-2 4 1 30.1051 1.30429 1

-2 4 1 26.2675 1.45212 1

1 -4 -1 6409.15 203.164 3

1 -4 -1 6410.58 202.469 6

1 -4 -1 6114.69 202.528 4

-1 4 1 6572.16 202.919 3

-1 4 1 6890.99 202.934 1

-1 4 1 6631.09 203.093 1

0 -4 -1 129.082 4.36609 3

0 -4 -1 128.939 4.27366 2

0 -4 -1 138.136 4.29592 1

0 -4 -1 127.057 4.24522 6

0 4 1 130.505 4.34433 1

0 4 1 137.970 4.38669 3

0 4 1 139.557 4.28081 2

0 4 1 124.496 4.33039 6

-1 -4 -1 1611.34 50.0515 1

-1 -4 -1 1610.60 50.2290 2

-1 -4 -1 1587.34 50.4165 5

1 4 1 1581.25 50.1138 1

1 4 1 1617.46 50.3000 6

1 4 1 1594.76 50.0942 2

-2 -4 -1 3.55489 0.48909 1

-2 -4 -1 2.15871 0.53648 5

-2 -4 -1 2.88790 0.49548 2

2 4 1 2.28571 0.55276 5

2 4 1 2.39255 0.50618 6

2 4 1 2.87958 0.46203 2

-3 -4 -1 233.791 7.79351 5

-3 -4 -1 242.507 7.69856 1

3 4 1 253.131 7.75978 2

3 4 1 234.314 7.70852 1

3 4 1 235.140 7.83571 6

3 4 1 243.299 7.81964 5

-4 -4 -1 473.081 15.1520 5

-4 -4 -1 463.804 14.5083 1

4 4 1 436.124 14.6475 5

4 4 1 483.462 14.6829 4

4 4 1 429.921 14.4630 1

4 4 1 467.242 14.5845 2

-5 -4 -1 823.488 27.1315 5

-5 -4 -1 888.858 27.0461 1

5 4 1 849.099 27.0166 1

5 4 1 883.916 27.2755 4

-6 -4 -1 4.93864 1.16513 5

6 4 1 5.18736 0.85778 1

6 4 1 5.64178 1.58532 4

-7 -4 -1 1.47173 1.24013 5

7 4 1 1.42942 0.87220 1

-8 -4 -1 37.4850 2.54075 5

8 4 1 35.8327 2.39964 1

-9 -4 -1 15.9391 1.91475 5

9 4 1 13.3245 1.62813 1

-10 -4 -1 4.27872 1.76838 5

10 4 1 1.57547 1.52058 1

10 4 1 1.98457 1.26612 3

-11 -4 -1 2.36352 2.14556 5

11 4 1 0.54704 1.32000 3

11 4 1 2.27896 1.81102 1

-12 -4 -1 4.66732 2.49266 5

12 4 1 0.77649 1.59628 3

-13 -4 -1 1.61768 3.19684 5

13 4 1 0.39167 2.32066 1

13 4 1 0.14478 1.79990 3

13 -5 -1 1.74275 3.03808 3

13 -5 -1 1.46586 2.75232 1

-13 5 1 4.68061 3.12110 1

12 -5 -1 4.85048 2.20539 1

12 -5 -1 -0.8080 2.32646 3

-12 5 1 4.19564 2.64625 1

11 -5 -1 2.02563 1.78610 1

11 -5 -1 1.00504 2.06287 3

-11 5 1 5.48499 2.42758 1

10 -5 -1 2.95499 1.83844 3

10 -5 -1 4.14010 1.62408 1

-10 5 1 5.04876 2.13363 1

9 -5 -1 66.0553 3.89889 3

9 -5 -1 60.9259 3.71177 1

-9 5 1 66.3718 4.14176 1

8 -5 -1 84.6464 4.03281 3

8 -5 -1 82.8107 3.97115 1

7 -5 -1 68.4907 3.41432 1

7 -5 -1 68.6786 3.42600 3

6 -5 -1 9.35800 1.21355 3

6 -5 -1 10.1883 1.18480 5

6 -5 -1 7.30252 1.23423 1

5 -5 -1 117.700 4.74243 1

5 -5 -1 128.811 4.21234 5

5 -5 -1 126.751 4.69791 3

4 -5 -1 144.835 5.25191 3

4 -5 -1 156.997 4.85060 5

4 -5 -1 141.652 5.26557 4

-4 5 1 146.570 5.09791 1

-4 5 1 148.794 5.01948 3

3 -5 -1 1861.00 57.3914 6

3 -5 -1 1752.92 57.2977 3

3 -5 -1 1768.87 56.9649 4

3 -5 -1 1797.18 57.3415 1

-3 5 1 1905.36 57.1285 3

-3 5 1 1895.62 57.7232 1

-3 5 1 1787.09 56.8798 1

2 -5 -1 59.6345 2.12765 6

2 -5 -1 59.5031 2.34393 1

2 -5 -1 57.7826 2.37460 3

2 -5 -1 55.7649 2.32998 4

-2 5 1 59.2021 2.21992 3

-2 5 1 56.8940 2.36753 1

-2 5 1 58.1717 2.17349 1

1 -5 -1 762.577 24.0651 2

1 -5 -1 754.385 24.0254 6

1 -5 -1 764.355 24.1784 3

-1 5 1 751.915 24.0364 6

-1 5 1 759.627 24.1499 1

-1 5 1 802.437 24.1205 1

-1 5 1 791.599 24.1582 3

0 -5 -1 323.651 10.5180 3

0 -5 -1 319.934 10.3726 6

0 -5 -1 328.978 10.4485 5

0 -5 -1 329.367 10.4224 1

0 -5 -1 330.219 10.4330 2

0 5 1 338.147 10.4303 2

0 5 1 335.376 10.5250 3

0 5 1 334.619 10.4043 6

0 5 1 326.247 10.4889 1

-1 -5 -1 3020.48 94.0493 1

-1 -5 -1 3000.96 94.3278 2

-1 -5 -1 2961.34 94.4545 5

1 5 1 3013.49 94.1085 2

1 5 1 2951.68 94.2509 1

1 5 1 3090.19 94.2835 6

1 5 1 3058.65 94.6040 3

-2 -5 -1 308.618 10.1507 2

-2 -5 -1 324.170 10.0834 1

-2 -5 -1 304.397 10.1803 5

2 5 1 316.822 10.1632 1

2 5 1 320.819 10.1414 2

2 5 1 325.038 10.1684 6

2 5 1 329.736 10.2128 5

-3 -5 -1 3016.35 93.9450 1

-3 -5 -1 3053.79 94.7618 5

-3 -5 -1 3000.35 94.5311 2

3 5 1 3011.91 94.1375 2

3 5 1 2985.07 94.0855 5

3 5 1 3016.48 94.6285 6

3 5 1 3025.25 94.2853 1

-4 -5 -1 74.8065 3.74243 5

-4 -5 -1 79.2294 2.88797 1

4 5 1 86.3047 3.08600 4

4 5 1 80.1076 2.87505 1

4 5 1 75.3231 3.04405 6

-5 -5 -1 256.173 8.69679 5

-5 -5 -1 263.461 8.54802 1

5 5 1 268.781 8.81856 4

-6 -5 -1 93.4003 3.79264 5

6 5 1 93.7930 4.13125 4

6 5 1 98.2759 3.48199 1

-7 -5 -1 275.613 9.41345 5

7 5 1 282.411 9.18194 1

-8 -5 -1 9.69113 1.63992 5

8 5 1 7.14012 1.16452 1

-9 -5 -1 5.17570 1.61495 5

9 5 1 3.66205 1.13505 3

9 5 1 3.64783 1.43128 1

-10 -5 -1 -0.0027 1.78134 5

10 5 1 -0.6157 1.00229 3

10 5 1 -0.0504 1.49326 1

-11 -5 -1 3.60788 2.25326 5

11 5 1 3.39005 1.85470 1

11 5 1 3.90425 1.53793 3

-12 -5 -1 2.33637 2.63318 5

12 5 1 0.71011 1.70794 3

12 5 1 3.45204 4.13145 1

-13 -5 -1 0.41584 3.22286 5

13 5 1 0.48452 2.42408 1

13 5 1 -0.0140 1.94135 3

13 -6 -1 2.00502 2.80269 1

13 -6 -1 -3.4615 3.18245 3

13 -6 -1 2.32081 2.99072 6

-13 6 1 3.90504 4.85083 1

12 -6 -1 -3.6448 2.58481 3

12 -6 -1 0.30927 2.43004 1

-12 6 1 0.15953 2.81408 1

11 -6 -1 1.02470 2.00943 1

11 -6 -1 3.27333 2.27811 3

-11 6 1 4.32877 2.48536 1

10 -6 -1 1.32616 1.70467 1

10 -6 -1 0.36741 1.71500 3

-10 6 1 2.86145 2.31424 1

9 -6 -1 2.48833 1.44415 3

9 -6 -1 3.56416 1.55746 1

-9 6 1 5.08550 1.99078 1

8 -6 -1 18.3339 2.76052 3

8 -6 -1 16.8895 2.50665 1

-8 6 1 13.1490 2.00283 1

7 -6 -1 2.26718 1.23317 1

7 -6 -1 1.73904 1.14707 3

6 -6 -1 185.182 6.51993 3

6 -6 -1 167.078 6.55912 1

6 -6 -1 182.687 5.94272 5

5 -6 -1 40.6266 1.66577 5

5 -6 -1 39.7029 2.50321 3

5 -6 -1 33.1002 2.39828 1

4 -6 -1 844.397 25.7971 4

4 -6 -1 833.526 26.7312 1

4 -6 -1 783.824 25.5519 6

-4 6 1 799.178 25.7712 1

-4 6 1 794.419 25.5484 3

3 -6 -1 284.550 9.44845 1

3 -6 -1 280.165 9.16947 6

3 -6 -1 286.475 9.39202 4

3 -6 -1 281.351 9.39587 3

-3 6 1 289.928 9.22806 3

-3 6 1 287.039 9.25636 1

2 -6 -1 921.759 29.1633 4

2 -6 -1 910.661 29.0384 6

2 -6 -1 921.704 29.2273 3

-2 6 1 954.831 29.1420 1

-2 6 1 931.127 29.2392 1

-2 6 1 934.577 29.1529 3

1 -6 -1 41.7043 1.75432 2

1 -6 -1 43.9933 1.97022 3

1 -6 -1 40.0013 1.66516 6

-1 6 1 44.1540 1.93178 3

-1 6 1 47.8948 1.79276 1

-1 6 1 44.0497 1.57026 6

-1 6 1 41.0517 1.94858 1

0 -6 -1 1722.18 54.7399 2

0 -6 -1 1730.97 54.7700 5

0 -6 -1 1702.99 54.5453 6

0 6 1 1770.51 54.4106 6

0 6 1 1881.92 54.7023 1

0 6 1 1691.03 54.3748 3

0 6 1 1706.74 54.4954 1

-1 -6 -1 933.580 29.9727 5

-1 -6 -1 947.045 29.9636 2

-1 -6 -1 968.012 29.9066 1

1 6 1 957.699 29.9218 6

1 6 1 954.588 30.0361 3

1 6 1 978.800 29.9559 2

1 6 1 964.951 30.0030 1

-2 -6 -1 384.129 12.3412 1

-2 -6 -1 384.871 12.4509 2

-2 -6 -1 391.199 12.4859 5

2 6 1 381.395 12.4670 1

2 6 1 396.711 12.5039 5

2 6 1 396.576 12.4338 6

2 6 1 403.252 12.4440 2

-3 -6 -1 132.016 4.33067 1

-3 -6 -1 131.684 4.56298 5

-3 -6 -1 132.249 4.49486 2

3 6 1 128.337 4.53283 5

3 6 1 133.946 4.48911 6

3 6 1 128.681 4.46237 1

3 6 1 123.891 4.44106 2

-4 -6 -1 8.74080 1.20706 1

-4 -6 -1 10.6730 1.19686 5

4 6 1 9.16964 1.03668 1

4 6 1 10.1041 1.34169 6

-5 -6 -1 51.1254 2.29476 1

-5 -6 -1 52.0475 2.51677 5

5 6 1 48.1486 2.63897 4

5 6 1 52.4875 2.17091 1

-6 -6 -1 114.039 4.47594 5

6 6 1 113.182 4.70595 4

6 6 1 117.915 4.13017 1

-7 -6 -1 28.1187 2.30135 5

7 6 1 29.1637 1.93807 1

-8 -6 -1 77.8975 3.93503 5

8 6 1 87.3486 3.55741 1

-9 -6 -1 0.70181 1.56461 5

9 6 1 1.91008 1.38314 1

-10 -6 -1 19.3761 2.58434 5

10 6 1 23.6966 3.04153 1

10 6 1 20.0251 2.66425 3

-11 -6 -1 -2.8011 2.48264 5

11 6 1 2.37546 1.81765 1

-12 -6 -1 4.16288 2.99361 5

12 6 1 3.08524 2.38038 1

12 6 1 -0.1690 1.79751 3

-13 -6 -1 2.02159 3.58987 5

13 6 1 1.70322 2.23516 3

13 6 1 1.56377 2.51623 1

13 -7 -1 -6.9772 3.44242 3

13 -7 -1 1.53197 3.17261 1

13 -7 -1 -1.5298 3.13828 6

-13 7 1 5.07260 3.54222 1

12 -7 -1 1.64439 2.85277 3

12 -7 -1 1.31333 3.11384 6

12 -7 -1 -0.0194 2.66873 1

-12 7 1 3.13653 3.20307 1

11 -7 -1 4.20389 2.55432 3

11 -7 -1 4.67343 2.96342 1

11 -7 -1 6.75075 2.36068 6

-11 7 1 7.75568 2.75339 1

10 -7 -1 -0.6553 1.82733 3

10 -7 -1 1.08855 1.95707 1

-10 7 1 0.63991 2.45486 1

9 -7 -1 3.01989 1.71022 1

9 -7 -1 4.28238 1.85043 3

-9 7 1 4.12320 2.07247 1

8 -7 -1 1.25906 1.53150 1

8 -7 -1 1.31151 1.44620 3

-8 7 1 -1.1271 1.81130 1

7 -7 -1 8.62530 1.74388 1

7 -7 -1 15.6825 1.95041 5

-7 7 1 10.5500 1.86284 1

6 -7 -1 76.8756 3.74205 3

6 -7 -1 70.0578 3.73403 1

6 -7 -1 71.5827 3.32845 6

6 -7 -1 75.5649 2.82349 5

-6 7 1 73.5410 3.88655 1

5 -7 -1 186.924 6.53904 6

5 -7 -1 184.497 6.89434 1

5 -7 -1 195.023 6.17976 5

5 -7 -1 197.876 6.82777 3

-5 7 1 179.846 6.88969 1

4 -7 -1 531.292 16.4328 3

4 -7 -1 501.833 16.1517 6

4 -7 -1 517.770 16.4212 4

4 -7 -1 466.871 16.6983 1

-4 7 1 515.355 16.5047 1

3 -7 -1 718.334 22.7220 4

3 -7 -1 704.319 22.5146 6

3 -7 -1 740.571 22.9838 3

-3 7 1 702.588 22.7948 1

-3 7 1 713.894 22.6011 3

-3 7 1 714.164 22.6261 1

2 -7 -1 1546.04 49.0943 6

2 -7 -1 1570.08 49.2537 4

2 -7 -1 1683.74 49.5250 2

2 -7 -1 1569.97 49.3292 3

-2 7 1 1467.02 49.5081 1

-2 7 1 1613.04 49.2362 1

-2 7 1 1582.63 49.2496 3

1 -7 -1 664.093 21.2574 3

1 -7 -1 660.772 21.1315 2

-1 7 1 692.663 21.1648 1

-1 7 1 690.858 21.2360 3

-1 7 1 648.142 21.2744 1

0 -7 -1 1640.71 50.5962 5

0 -7 -1 1571.29 50.2374 6

0 -7 -1 1630.60 50.4734 2

0 7 1 1664.95 50.1529 1

0 7 1 1616.33 50.2187 3

0 7 1 1520.61 50.1986 1

-1 -7 -1 58.0415 2.37482 2

-1 -7 -1 57.3417 2.35007 5

1 7 1 60.5565 2.32316 2

1 7 1 57.4594 2.45105 3

1 7 1 60.9859 2.51002 1

1 7 1 62.4091 2.30311 6

-2 -7 -1 -0.4470 1.14734 2

-2 -7 -1 -0.2194 1.30133 5

-2 -7 -1 2.35432 0.54939 1

2 7 1 -0.1519 0.62865 2

2 7 1 1.31364 0.84650 1

2 7 1 1.46819 0.61866 3

-3 -7 -1 4.91667 0.88159 2

-3 -7 -1 4.25029 0.66908 1

-3 -7 -1 4.49537 1.03173 5

3 7 1 4.63712 1.03788 1

3 7 1 5.05461 0.84427 6

-4 -7 -1 103.570 3.83294 2

-4 -7 -1 101.181 3.91152 5

-4 -7 -1 104.606 3.57550 1

4 7 1 100.522 3.81656 6

-5 -7 -1 208.194 7.05918 5

-5 -7 -1 195.639 6.68770 1

5 7 1 208.525 6.78103 1

-6 -7 -1 259.909 8.85877 5

6 7 1 262.010 8.54908 1

7 7 1 72.4119 3.05721 1

-8 -7 -1 16.7727 1.97449 5

8 7 1 17.8241 2.11067 1

-9 -7 -1 7.20870 2.02608 5

9 7 1 7.73709 1.54305 1

-10 -7 -1 12.4373 2.33915 5

10 7 1 6.11101 1.60383 3

10 7 1 5.54800 1.77951 1

-11 -7 -1 -0.2823 2.42277 5

11 7 1 2.08528 1.95178 1

11 7 1 0.46923 1.60099 3

-12 -7 -1 -0.1720 2.97347 5

12 7 1 -0.1341 2.22750 1

12 7 1 1.90999 2.05132 3

12 -8 -1 4.03230 3.41771 3

12 -8 -1 4.51053 2.87536 1

12 -8 -1 -0.2111 2.90108 6

-12 8 1 5.87163 3.39107 1

11 -8 -1 2.45035 2.49300 6

11 -8 -1 2.37524 2.55576 1

-11 8 1 3.93009 3.03663 1

10 -8 -1 3.41794 2.32740 3

10 -8 -1 2.68419 2.11939 6

10 -8 -1 -0.3307 2.09433 1

-10 8 1 3.45503 2.43727 1

9 -8 -1 22.4242 3.63419 1

9 -8 -1 31.1649 4.09606 3

9 -8 -1 22.6181 3.00748 6

8 -8 -1 33.0836 3.34487 1

8 -8 -1 34.4862 2.82856 6

8 -8 -1 37.5185 3.75044 3

-8 8 1 34.8700 3.82060 1

7 -8 -1 8.25129 1.67507 3

7 -8 -1 9.77899 1.82016 6

7 -8 -1 5.97358 1.79791 1

7 -8 -1 9.90123 1.38696 5

-7 8 1 7.05472 1.99287 1

6 -8 -1 0.07954 1.28385 3

6 -8 -1 -1.8211 1.23439 6

6 -8 -1 -0.6116 1.34432 1

6 -8 -1 0.04374 0.74243 5

-6 8 1 -1.1893 1.48190 1

5 -8 -1 32.1485 2.77049 3

5 -8 -1 22.9586 2.69623 1

5 -8 -1 26.0355 2.14517 6

-5 8 1 26.6408 2.72035 1

4 -8 -1 11.1749 1.59969 6

4 -8 -1 9.48806 1.33500 3

3 -8 -1 57.4153 2.88434 4

3 -8 -1 58.7737 2.99775 3

3 -8 -1 60.4600 2.47144 6

-3 8 1 54.3358 2.48388 1

-3 8 1 59.0530 2.93527 1

2 -8 -1 181.245 6.74740 3

2 -8 -1 187.315 6.42587 2

-2 8 1 182.678 6.32928 3

-2 8 1 189.083 6.55570 1

-2 8 1 192.386 6.29290 1

1 -8 -1 25.5519 1.53162 5

1 -8 -1 23.9774 1.56555 2

-1 8 1 26.0364 1.92283 1

-1 8 1 25.2454 1.49777 1

-1 8 1 24.5011 1.79795 3

0 -8 -1 69.2721 3.01488 2

0 -8 -1 74.3635 2.79377 5

0 8 1 76.9631 3.05424 3

0 8 1 78.3252 2.82651 1

0 8 1 66.9820 2.98696 1

-1 -8 -1 50.0302 2.31238 5

-1 -8 -1 53.7563 2.33699 2

1 8 1 55.0383 2.24813 6

1 8 1 54.1940 2.43669 3

1 8 1 53.1297 2.23591 2

-2 -8 -1 4.46716 0.90185 2

-2 -8 -1 2.23345 1.70461 5

2 8 1 5.95694 0.91871 6

-3 -8 -1 303.427 9.57011 5

-3 -8 -1 283.814 9.26269 1

-3 -8 -1 288.595 9.49916 2

3 8 1 275.949 9.67446 1

3 8 1 297.674 9.47000 6

-4 -8 -1 154.408 5.54677 2

-4 -8 -1 158.568 5.60569 5

4 8 1 159.752 5.47402 1

-5 -8 -1 40.1151 2.54216 5

5 8 1 43.4771 2.22427 1

-6 -8 -1 0.84549 1.14789 5

6 8 1 -0.7698 0.86006 1

-7 -8 -1 39.7990 2.88952 5

7 8 1 43.7368 2.42240 1

-8 -8 -1 5.95353 1.78387 5

8 8 1 7.57912 1.25863 1

-9 -8 -1 2.55452 1.98267 5

9 8 1 1.18458 1.41790 1

-10 -8 -1 -1.8397 2.05913 5

10 8 1 0.31461 1.65456 1

10 8 1 -1.2275 1.36577 3

-11 -8 -1 0.96622 2.74014 5

11 8 1 -1.4449 1.71808 3

11 8 1 2.32725 2.01659 1

-12 -8 -1 5.96466 3.23516 5

12 8 1 3.18886 2.10968 3

12 8 1 2.49781 2.30118 1

12 -9 -1 -2.2490 3.24660 1

12 -9 -1 -3.0529 3.05600 6

12 -9 -1 -1.4594 3.49453 3

-12 9 1 4.08899 3.65982 1

11 -9 -1 -1.0755 2.89096 1

11 -9 -1 -3.1067 2.85403 3

11 -9 -1 -2.3144 2.69821 6

10 -9 -1 -1.4655 2.22012 6

10 -9 -1 -0.6449 2.62382 3

10 -9 -1 -1.7249 2.46026 1

-10 9 1 4.24870 2.85246 1

9 -9 -1 0.11061 1.94115 6

9 -9 -1 2.15663 2.17789 1

9 -9 -1 -1.5261 2.20521 3

-9 9 1 1.33718 2.46478 1

8 -9 -1 -1.7641 1.87311 1

8 -9 -1 0.94489 1.79540 3

8 -9 -1 -0.4509 1.78463 6

-8 9 1 1.46544 1.98465 1

7 -9 -1 9.30791 2.06857 1

7 -9 -1 8.15657 1.73741 3

7 -9 -1 12.4908 1.94122 6

-7 9 1 8.89104 2.05967 1

6 -9 -1 48.7192 3.58184 1

6 -9 -1 63.1170 3.73651 3

6 -9 -1 50.5316 3.02731 6

-6 9 1 55.5744 3.69151 1

5 -9 -1 36.8462 2.35471 6

5 -9 -1 37.2476 2.86998 3

-5 9 1 25.9703 2.90332 1

4 -9 -1 0.35220 1.21328 3

-4 9 1 -2.9787 1.34052 1

3 -9 -1 17.9187 2.20025 3

3 -9 -1 17.0820 1.74038 2

3 -9 -1 14.4116 2.08753 4

-3 9 1 14.4672 2.31153 1

2 -9 -1 12.0468 1.53553 2

2 -9 -1 9.12424 1.16358 3

-2 9 1 7.74720 1.46414 1

-2 9 1 8.20715 1.08232 1

1 -9 -1 3.45154 0.87466 5

1 -9 -1 5.01282 1.11607 2

1 -9 -1 2.71347 0.83993 3

-1 9 1 3.07888 0.84223 1

-1 9 1 0.77975 1.13236 1

0 -9 -1 3.91815 0.93547 2

0 -9 -1 1.81996 0.92673 5

0 9 1 1.29529 0.86091 1

0 9 1 -0.8544 1.11754 1

-1 -9 -1 69.1194 2.81355 2

1 9 1 74.4482 2.75826 1

1 9 1 64.8203 3.02912 1

1 9 1 62.0265 2.69547 6

-2 -9 -1 1.52968 1.07923 5

-2 -9 -1 2.43333 0.87926 2

2 9 1 -0.2446 1.12966 1

2 9 1 1.38679 0.98319 6

-3 -9 -1 -0.6535 0.90134 2

-3 -9 -1 1.41689 1.11021 5

3 9 1 1.52927 1.10974 1

3 9 1 2.19681 0.98461 6

-4 -9 -1 26.7752 2.14176 2

-4 -9 -1 29.4137 2.24815 5

4 9 1 24.3217 2.09975 1

-5 -9 -1 6.03440 1.46059 5

5 9 1 6.51267 1.10728 1

-6 -9 -1 -1.0472 1.29885 5

6 9 1 -1.6787 0.99937 1

-7 -9 -1 4.54005 1.78778 5

7 9 1 8.66577 1.21296 1

-8 -9 -1 16.9028 2.27873 5

8 9 1 17.8299 2.33622 1

-9 -9 -1 9.38459 2.27115 5

9 9 1 6.62428 1.54988 1

-10 -9 -1 1.64605 2.61043 5

10 9 1 5.94331 1.81139 1

-11 -9 -1 0.49209 2.83732 5

11 9 1 0.91527 2.03642 1

11 9 1 -0.8916 1.86973 3

-12 -9 -1 2.50767 3.55206 5

12 9 1 0.38572 2.19411 1

12 9 1 -1.6733 2.32508 3

11 -10 -1 3.48015 2.87669 6

11 -10 -1 1.26101 3.12909 3

-11 10 1 5.47389 3.44659 1

10 -10 -1 -2.0475 2.37127 6

10 -10 -1 -0.4828 3.65522 1

10 -10 -1 -0.8453 2.73568 3

-10 10 1 3.56856 3.06388 1

9 -10 -1 5.08896 2.19092 6

9 -10 -1 6.76669 2.56363 1

9 -10 -1 3.52251 2.25976 3

-9 10 1 7.41542 2.81021 1

8 -10 -1 2.78357 2.09132 3

8 -10 -1 0.88099 2.27112 1

8 -10 -1 0.15405 1.89954 6

-8 10 1 2.60848 2.32606 1

7 -10 -1 5.48656 1.90072 3

7 -10 -1 2.36756 1.72709 6

-7 10 1 2.20883 2.02465 1

6 -10 -1 0.06120 1.48723 3

-6 10 1 -2.2755 1.68641 1

5 -10 -1 11.3151 1.61471 6

5 -10 -1 8.58962 1.62949 3

-5 10 1 10.0895 1.89593 1

4 -10 -1 119.189 4.84011 3

-4 10 1 111.535 5.08868 1

3 -10 -1 13.4668 1.83687 2

3 -10 -1 17.1441 2.25303 4

3 -10 -1 13.1465 1.52420 3

-3 10 1 15.4410 1.84899 1

2 -10 -1 0.49164 0.93369 3

2 -10 -1 2.68396 1.00084 5

2 -10 -1 1.43634 0.98891 2

-2 10 1 0.15331 0.88728 1

-2 10 1 -0.7845 1.32202 1

1 -10 -1 32.5931 2.11020 2

1 -10 -1 29.7222 2.18859 5

-1 10 1 31.6276 2.55458 1

-1 10 1 35.9054 1.96127 1

0 -10 -1 61.2485 2.79075 2

0 10 1 63.7274 2.67292 1

0 10 1 58.6807 3.15215 1

-1 -10 -1 11.5906 1.64721 5

-1 -10 -1 11.7543 1.70378 2

1 10 1 10.6212 1.52072 6

1 10 1 12.5315 1.51781 1

1 10 1 15.3349 1.59781 1

-2 -10 -1 0.86285 1.10888 5

-2 -10 -1 0.84483 0.97633 2

2 10 1 1.29581 1.08146 6

2 10 1 1.98747 1.26664 1

-3 -10 -1 78.4526 3.27432 5

-3 -10 -1 74.3697 3.22063 2

3 10 1 74.1113 3.14536 6

3 10 1 75.1160 3.37587 1

-4 -10 -1 49.7969 2.71217 2

-4 -10 -1 47.9264 2.79139 5

4 10 1 56.0656 2.71690 1

-5 -10 -1 18.2487 2.28204 5

5 10 1 20.3008 2.10901 1

-6 -10 -1 76.4489 3.88169 5

6 10 1 81.4954 3.41139 1

-7 -10 -1 100.802 4.55929 5

7 10 1 97.8254 3.97919 1

-8 -10 -1 16.2233 2.36157 5

8 10 1 12.9412 1.61432 1

-9 -10 -1 5.74315 2.42034 5

9 10 1 2.19189 1.48995 1

-10 -10 -1 0.34308 2.83152 5

10 10 1 2.65622 1.82718 1

-11 -10 -1 -3.5467 3.19094 5

11 10 1 0.08517 1.96895 1

11 10 1 0.12382 1.93899 3

11 -11 -1 0.26911 3.22619 3

-11 11 1 9.39557 3.86504 1

10 -11 -1 -2.0894 2.37072 6

10 -11 -1 -0.5148 3.22172 3

-10 11 1 1.01978 3.15145 1

9 -11 -1 2.43172 2.62286 3

9 -11 -1 -3.8393 2.18252 6

-9 11 1 5.30791 2.99893 1

8 -11 -1 -0.2647 1.98256 3

8 -11 -1 -0.8732 1.79957 6

-8 11 1 0.87185 2.62988 1

7 -11 -1 0.99439 1.85534 3

7 -11 -1 -2.1469 1.69756 6

-7 11 1 -0.0779 2.21500 1

6 -11 -1 21.4126 3.13646 3

6 -11 -1 16.5040 2.31518 6

-6 11 1 14.8770 2.48081 1

5 -11 -1 0.93728 1.41026 3

-5 11 1 -1.2735 1.87362 1

4 -11 -1 67.9209 3.62280 3

4 -11 -1 68.9019 3.25377 2

-4 11 1 63.1520 4.03355 1

3 -11 -1 148.013 5.60127 3

3 -11 -1 142.553 5.33897 2

-3 11 1 141.296 5.90542 1

2 -11 -1 186.603 6.58664 2

2 -11 -1 189.930 6.75641 3

-2 11 1 186.516 6.97488 1

1 -11 -1 31.0723 2.19173 2

-1 11 1 28.8315 2.77745 1

-1 11 1 31.0562 2.13062 1

0 -11 -1 61.6225 2.93386 2

0 -11 -1 60.8977 2.83473 5

0 11 1 62.1639 3.29279 1

0 11 1 66.1916 2.85831 1

-1 -11 -1 3.29594 1.09703 2

-1 -11 -1 3.87453 1.15537 5

1 11 1 3.48735 1.34114 1

1 11 1 3.59038 0.98742 1

-2 -11 -1 -0.5529 1.02420 2

-2 -11 -1 -0.7468 1.07832 5

2 11 1 -1.1740 1.11473 6

2 11 1 -0.6029 0.94045 1

2 11 1 -0.1851 1.28267 1

-3 -11 -1 15.4446 1.68740 5

-3 -11 -1 14.5503 1.91587 2

3 11 1 18.9169 1.98489 6

3 11 1 13.6316 1.67618 1

-4 -11 -1 -1.2716 1.33283 5

-4 -11 -1 -0.1138 1.08060 2

4 11 1 -1.0234 1.26076 1

-5 -11 -1 88.4797 4.04382 5

5 11 1 90.0991 3.84628 1

-6 -11 -1 65.1833 3.53296 5

6 11 1 53.0924 2.92971 1

-7 -11 -1 1.99717 1.88574 5

7 11 1 -0.1234 1.23913 1

-8 -11 -1 5.49271 2.22165 5

8 11 1 0.27895 1.30317 1

-9 -11 -1 -0.9886 2.59003 5

9 11 1 -1.4705 1.58850 1

-10 -11 -1 3.34913 3.05188 5

10 11 1 3.28196 1.82035 1

-11 -11 -1 0.79379 3.58498 5

11 11 1 2.05336 2.46431 3

10 -12 -1 -1.2591 2.87295 3

-10 12 1 0.28615 3.38523 1

9 -12 -1 1.67656 2.43481 3

-9 12 1 0.48587 3.12447 1

8 -12 -1 -2.9383 1.97735 6

8 -12 -1 -0.3785 2.21077 3

-8 12 1 0.33580 2.86666 1

7 -12 -1 4.05897 1.89860 3

7 -12 -1 2.90091 1.82698 6

-7 12 1 4.23870 2.74577 1

6 -12 -1 53.5937 4.11155 3

-6 12 1 50.8612 4.81421 1

5 -12 -1 2.74050 1.52897 3

5 -12 -1 2.18661 1.38627 2

-5 12 1 1.76704 2.11855 1

4 -12 -1 19.0884 2.88725 3

4 -12 -1 16.9076 2.27521 2

-4 12 1 13.4089 2.28607 1

3 -12 -1 -0.8122 1.11077 3

3 -12 -1 -0.1559 1.30455 2

3 -12 -1 -0.0280 1.01041 5

-3 12 1 -2.7657 1.72604 1

2 -12 -1 118.263 4.67825 2

2 -12 -1 124.735 4.81416 3

2 -12 -1 113.875 4.56972 5

-2 12 1 118.632 5.19993 1

1 -12 -1 0.81955 1.14635 2

1 -12 -1 -0.4204 1.09092 5

-1 12 1 0.95660 0.97881 1

-1 12 1 0.17448 1.54287 1

0 -12 -1 48.0059 2.54840 5

0 -12 -1 49.0604 2.71472 2

0 12 1 47.6407 2.54542 1

0 12 1 47.6319 3.17246 1

-1 -12 -1 58.9832 2.95574 2

-1 -12 -1 55.4463 2.75835 5

1 12 1 56.8908 2.77225 1

1 12 1 50.7646 3.29621 1

-2 -12 -1 6.57002 1.30039 2

-2 -12 -1 6.82578 1.38140 5

2 12 1 6.41984 1.64222 1

2 12 1 5.42934 1.20634 1

2 12 1 4.91286 1.34938 6

-3 -12 -1 5.36485 1.48903 5

-3 -12 -1 4.43035 1.35927 2

3 12 1 4.45782 1.56081 1

3 12 1 5.04267 1.24121 1

-4 -12 -1 16.9574 2.30965 2

-4 -12 -1 15.9411 2.39902 5

4 12 1 19.6422 2.54510 1

-5 -12 -1 1.41320 1.58446 5

5 12 1 3.05432 1.32137 1

-6 -12 -1 36.3907 3.22557 5

6 12 1 30.8081 2.60256 1

-7 -12 -1 0.99270 2.09179 5

7 12 1 1.82196 1.31131 1

-8 -12 -1 12.5105 2.88896 5

-9 -12 -1 3.51405 2.87675 5

-10 -12 -1 11.7049 3.46408 5

10 -13 -1 2.91356 2.97670 3

-10 13 1 5.31158 4.05165 1

9 -13 -1 1.65449 2.54585 3

-9 13 1 -0.4012 3.49417 1

8 -13 -1 0.21325 2.25819 3

-8 13 1 4.42546 3.18211 1

7 -13 -1 14.9397 2.42017 3

7 -13 -1 13.3991 2.08056 2

-7 13 1 12.7615 3.16172 1

6 -13 -1 11.8619 1.97001 2

6 -13 -1 13.9519 2.12883 3

-6 13 1 9.43813 2.92204 1

5 -13 -1 28.2285 2.85848 2

5 -13 -1 30.0027 3.62908 3

-5 13 1 31.4486 4.42923 1

4 -13 -1 38.8577 2.94661 2

4 -13 -1 47.1220 3.54137 3

-4 13 1 38.6596 4.18554 1

3 -13 -1 89.7214 4.32440 3

3 -13 -1 83.2225 4.00200 2

-3 13 1 85.7903 4.81597 1

2 -13 -1 5.46571 1.24867 5

2 -13 -1 4.10571 1.44953 2

-2 13 1 1.95551 1.88569 1

1 -13 -1 6.10356 1.50817 2

1 -13 -1 6.00688 1.34416 5

-1 13 1 1.89262 1.81274 1

0 -13 -1 0.94940 1.35704 2

0 -13 -1 -1.2279 1.21078 5

0 13 1 0.42428 1.63608 1

-1 -13 -1 151.995 5.44626 5

-1 -13 -1 145.871 5.58137 2

1 13 1 143.443 5.85403 1

-2 -13 -1 1.69249 1.43890 2

-2 -13 -1 0.13984 1.36855 5

2 13 1 2.11057 1.67943 1

-3 -13 -1 25.0385 2.44956 2

-3 -13 -1 24.8263 2.50518 5

3 13 1 21.0203 2.87323 1

-4 -13 -1 -0.5628 1.60061 5

-4 -13 -1 -0.4974 1.46503 2

4 13 1 2.08923 1.53759 1

-5 -13 -1 1.17362 1.70909 5

5 13 1 -2.8125 1.35811 1

-6 -13 -1 -2.2175 1.91143 5

6 13 1 -0.3666 1.27721 1

-7 -13 -1 0.49873 2.22376 5

-8 -13 -1 28.4767 4.32967 5

-9 -13 -1 15.1775 3.49208 5

-10 -13 -1 4.86529 3.65466 5

9 -14 -1 2.65797 2.33784 2

9 -14 -1 0.07915 2.70676 3

-9 14 1 1.68298 3.92199 1

8 -14 -1 4.89455 2.77441 3

-8 14 1 4.09809 3.61486 1

7 -14 -1 2.78991 2.15899 3

7 -14 -1 0.95097 1.92619 2

-7 14 1 5.52940 3.28476 1

6 -14 -1 1.35517 1.77540 2

6 -14 -1 0.71169 1.86659 3

5 -14 -1 60.4330 4.33851 3

5 -14 -1 56.7578 3.65961 2

-5 14 1 57.1923 5.34703 1

4 -14 -1 33.6492 3.45234 3

4 -14 -1 27.6839 3.03866 2

-4 14 1 24.7581 3.11800 1

3 -14 -1 4.01021 1.66619 2

-3 14 1 3.69764 2.35646 1

2 -14 -1 5.03511 1.64755 2

-2 14 1 4.56819 2.20424 1

1 -14 -1 148.590 5.35819 5

1 -14 -1 143.936 5.77836 2

-1 14 1 147.952 6.32872 1

0 -14 -1 6.36363 1.74331 2

0 -14 -1 6.34115 1.48384 5

0 14 1 6.70070 1.98321 1

-1 -14 -1 15.0304 1.78734 5

-1 -14 -1 10.9177 1.85885 2

1 14 1 12.7928 2.10906 1

-2 -14 -1 2.28226 1.55485 2

-2 -14 -1 4.92606 1.62331 5

2 14 1 0.95681 1.85172 1

-3 -14 -1 57.9584 3.35152 5

-3 -14 -1 56.4567 3.40199 2

3 14 1 52.6334 3.70602 1

-4 -14 -1 1.32451 1.66957 2

-4 -14 -1 -0.7182 1.71433 5

4 14 1 0.87905 1.67658 1

-5 -14 -1 10.7979 2.22752 5

5 14 1 9.43058 1.72211 1

-6 -14 -1 24.3576 3.60115 5

-7 -14 -1 5.46475 2.65371 5

-8 -14 -1 14.3877 3.37194 5

-9 -14 -1 7.70683 3.55054 5

8 -15 -1 1.58196 2.62519 2

8 -15 -1 5.84774 2.86325 3

-8 15 1 4.77929 3.84252 1

7 -15 -1 9.40879 2.42776 3

7 -15 -1 9.71905 2.32725 2

-7 15 1 8.79457 3.76882 1

6 -15 -1 2.11623 2.01157 2

6 -15 -1 1.51739 2.05502 3

-6 15 1 0.90699 3.19759 1

5 -15 -1 5.91008 2.06418 2

5 -15 -1 6.59657 1.92328 3

-5 15 1 5.00065 3.08020 1

4 -15 -1 1.37556 1.59406 3

4 -15 -1 1.52536 1.83405 2

-4 15 1 -0.6772 2.78176 1

3 -15 -1 19.1088 2.28232 2

-3 15 1 13.7333 2.98210 1

2 -15 -1 1.36464 1.78691 2

-2 15 1 -0.0844 2.35267 1

1 -15 -1 -1.7280 1.34042 5

1 -15 -1 -1.1730 1.55380 2

-1 15 1 0.35681 2.14408 1

0 -15 -1 51.1777 3.00280 5

0 -15 -1 53.0997 3.51212 2

0 15 1 54.3083 4.17906 1

-1 -15 -1 8.41000 1.81013 2

-1 -15 -1 11.7505 1.75249 5

1 15 1 6.26736 2.11801 1

-2 -15 -1 2.52047 1.76087 2

-2 -15 -1 3.32920 1.71315 5

2 15 1 1.75896 2.07222 1

-3 -15 -1 0.21446 1.64474 2

-3 -15 -1 -0.1827 1.77526 5

3 15 1 -1.3912 1.94152 1

-4 -15 -1 42.3186 3.56854 2

-4 -15 -1 41.9835 3.54969 5

4 15 1 39.9558 3.48447 1

-5 -15 -1 -1.8222 2.12341 5

5 15 1 1.07051 1.72787 1

-6 -15 -1 5.54837 5.08730 5

-7 -15 -1 3.12697 2.88798 5

-8 -15 -1 4.52485 3.39856 5

7 -16 -1 5.42106 2.67739 2

7 -16 -1 12.8175 2.66891 3

-7 16 1 16.6948 4.13123 1

6 -16 -1 5.35705 2.31070 3

6 -16 -1 7.41689 2.55828 2

-6 16 1 2.02716 3.66374 1

5 -16 -1 5.00609 2.32482 2

5 -16 -1 4.36781 1.96721 3

-5 16 1 1.99006 3.37042 1

4 -16 -1 17.8692 2.58775 2

-4 16 1 18.7189 3.54371 1

3 -16 -1 7.09869 2.19328 2

-3 16 1 4.32311 5.36860 1

2 -16 -1 19.1836 2.39915 2

-2 16 1 17.7844 3.07021 1

1 -16 -1 -0.1969 1.39770 5

1 -16 -1 0.58780 1.81695 2

-1 16 1 -0.0464 2.41194 1

0 -16 -1 -0.0333 1.81110 2

0 -16 -1 -0.0157 1.50421 5

0 16 1 -2.4956 2.18145 1

-1 -16 -1 9.27909 2.11039 2

-1 -16 -1 11.1162 1.84755 5

1 16 1 6.28908 2.42163 1

-2 -16 -1 49.7552 3.42266 5

-2 -16 -1 49.6188 3.69624 2

2 16 1 43.7236 4.10382 1

-3 -16 -1 -0.2903 1.85804 2

-3 -16 -1 2.18300 1.94528 5

3 16 1 -0.4247 2.08288 1

-4 -16 -1 -0.0300 2.05988 2

-4 -16 -1 -1.0380 2.14056 5

4 16 1 -0.0282 1.98846 1

5 16 1 -0.4567 1.89625 1

-6 -16 -1 0.43168 2.79803 5

-7 -16 -1 3.36972 3.21854 5

6 -17 -1 9.70793 2.86182 2

6 -17 -1 8.17351 2.49545 3

-6 17 1 8.90504 4.05489 1

5 -17 -1 8.84236 4.23760 2

-5 17 1 3.87649 3.81977 1

4 -17 -1 -0.0246 2.41111 2

-4 17 1 -2.6861 3.31590 1

3 -17 -1 2.88773 2.31997 2

-3 17 1 2.84092 3.18809 1

2 -17 -1 3.21992 2.24093 2

-2 17 1 3.68113 2.98267 1

1 -17 -1 -0.6805 2.15665 5

1 -17 -1 2.56727 2.15703 2

-1 17 1 0.55682 2.59380 1

0 -17 -1 8.51830 2.25753 2

0 -17 -1 7.48699 1.78204 5

-1 -17 -1 -1.2175 1.87269 2

-1 -17 -1 1.16566 1.81794 5

1 17 1 2.76561 2.80919 1

-2 -17 -1 5.13955 2.11869 5

-2 -17 -1 0.66856 2.08663 2

2 17 1 0.43568 2.44237 1

-3 -17 -1 1.63398 2.26251 2

-3 -17 -1 0.50637 2.14599 5

3 17 1 0.78369 2.42618 1

-4 -17 -1 4.25794 2.55268 2

4 17 1 -0.6211 2.23056 1

-5 -17 -1 -0.0938 2.70281 5

-6 -17 -1 -3.3944 2.95370 5

4 -18 -1 0.94380 2.67813 2

-4 18 1 0.44575 3.73170 1

3 -18 -1 -1.7070 2.43316 2

-3 18 1 3.76874 3.56590 1

2 -18 -1 1.70369 2.62987 2

-2 18 1 0.34403 3.29891 1

1 -18 -1 0.03571 2.56359 2

-1 18 1 2.06434 3.01236 1

0 -18 -1 -0.0794 2.40901 2

0 18 1 -1.1560 2.83092 1

-1 -18 -1 2.11527 2.30912 2

-1 -18 -1 2.79928 2.18292 5

1 18 1 -1.7255 2.83852 1

-2 -18 -1 -0.7693 2.08464 5

-2 -18 -1 -0.7604 2.30655 2

2 18 1 3.14622 2.86908 1

-3 -18 -1 -0.0541 3.38500 5

-3 -18 -1 2.34289 2.48628 2

3 18 1 -2.5004 2.71444 1

-4 -18 -1 6.26590 2.82039 5

4 18 1 -0.2886 2.42173 1

1 -19 -1 4.75048 2.82166 2

-1 19 1 1.44353 3.50204 1

0 -19 -1 9.83328 2.82541 2

0 19 1 3.36957 3.39556 1

1 19 1 -1.5689 3.19542 1

0 19 -2 1.40325 3.19735 1

0 -19 2 0.79919 2.98485 2

-4 -18 2 2.22508 2.63290 2

-4 -18 2 -0.2226 2.41140 5

3 18 -2 0.23522 2.59607 1

-3 -18 2 1.30182 2.46898 2

-3 -18 2 3.24494 2.49761 5

2 18 -2 4.94773 3.02379 1

-2 -18 2 11.3267 2.76189 2

1 18 -2 2.38173 2.69682 1

-1 -18 2 4.05243 2.55570 2

0 18 -2 6.12133 2.90458 1

0 -18 2 12.3828 2.89175 2

-1 18 -2 -0.3691 2.88797 1

1 -18 2 -2.0653 2.40260 2

-2 18 -2 0.00271 3.09393 1

2 -18 2 5.40843 2.68214 2

-3 18 -2 -0.8421 3.27486 1

3 -18 2 2.47799 3.01394 2

-4 18 -2 12.7537 3.93515 1

4 -18 2 18.1091 3.22505 2

-6 -17 2 4.00970 2.79089 5

-5 -17 2 12.4755 2.98436 2

-5 -17 2 11.3807 2.74507 5

-4 -17 2 9.98496 2.35856 5

-4 -17 2 2.72655 2.39963 2

3 17 -2 6.65293 2.27952 1

-3 -17 2 8.71686 2.12978 5

-3 -17 2 3.99871 2.28944 2

2 17 -2 -2.7733 2.23132 1

-2 -17 2 0.20129 2.27587 2

-2 -17 2 -1.0884 1.66973 5

1 17 -2 0.66445 2.33425 1

-1 -17 2 2.30406 1.76522 5

0 17 -2 0.58949 2.64217 1

0 -17 2 -2.5650 2.36214 2

-1 17 -2 3.76145 2.92801 1

1 -17 2 3.68391 2.51685 2

-2 17 -2 4.31328 2.95256 1

2 -17 2 6.70226 3.80586 2

-3 17 -2 7.96762 3.22133 1

3 -17 2 7.30548 3.00450 2

-4 17 -2 9.82684 3.48212 1

4 -17 2 10.7115 2.90291 2

-5 17 -2 5.32512 3.67074 1

5 -17 2 8.18064 2.84912 2

-6 17 -2 0.30383 3.64373 1

6 -17 2 2.83029 2.85463 2

-7 -16 2 3.49469 2.77021 5

-6 -16 2 -1.2245 2.45279 5

-5 -16 2 0.90879 2.21697 2

-5 -16 2 -0.2245 2.16473 5

-4 -16 2 2.11152 2.23001 2

-4 -16 2 5.14657 2.21675 5

3 16 -2 15.3660 2.28295 1

-3 -16 2 18.5835 2.47685 2

-3 -16 2 21.5202 2.32075 5

2 16 -2 -0.8839 2.05324 1

-2 -16 2 0.44304 1.53104 5

-2 -16 2 0.09445 1.83595 2

1 16 -2 1.71047 2.11647 1

-1 -16 2 2.89107 1.93565 2

-1 -16 2 0.93986 1.43672 5

0 16 -2 -0.9565 2.22439 1

0 -16 2 0.95107 1.92988 2

-1 16 -2 17.7780 2.75620 1

1 -16 2 22.3283 2.72486 2

-2 16 -2 -2.3463 2.53052 1

2 -16 2 3.33846 2.19269 2

-3 16 -2 0.74433 3.61312 1

3 -16 2 -0.9497 2.05146 2

-4 16 -2 -5.2615 2.73531 1

4 -16 2 1.45564 2.26783 2

-5 16 -2 10.7375 3.30504 1

-6 16 -2 3.74286 3.45598 1

6 -16 2 1.65845 2.48841 2

-7 16 -2 -3.1187 3.51477 1

7 -16 2 0.85589 2.46923 2

7 -16 2 8.33212 3.03020 3

-8 -15 2 1.12389 2.94724 5

-7 -15 2 -1.5720 2.56350 5

-6 -15 2 -1.2899 2.29709 5

-5 -15 2 6.81449 2.24554 5

-5 -15 2 6.16902 2.46410 2

-4 -15 2 9.50121 2.01718 5

-4 -15 2 6.11716 2.01757 2

3 15 -2 18.2285 2.12234 1

-3 -15 2 18.1484 2.74392 5

-3 -15 2 18.8366 2.45659 2

2 15 -2 -0.2475 1.80868 1

-2 -15 2 2.12670 1.46769 5

-2 -15 2 -0.3327 1.69783 2

1 15 -2 12.7150 2.15710 1

-1 -15 2 12.5636 1.64636 5

-1 -15 2 10.0058 1.94933 2

0 15 -2 -0.1242 1.96194 1

0 -15 2 0.65414 1.76418 2

-1 15 -2 11.3993 2.42067 1

1 -15 2 9.03514 2.04000 2

-2 15 -2 6.61364 2.34075 1

2 -15 2 9.70321 2.08125 2

-3 15 -2 6.85188 2.50270 1

3 -15 2 7.28605 2.06508 2

-4 15 -2 -0.3067 2.61239 1

4 -15 2 2.09716 1.97469 2

-5 15 -2 1.70241 2.75109 1

5 -15 2 5.95397 1.64381 3

5 -15 2 1.57074 2.18056 2

-6 15 -2 2.65927 2.91786 1

6 -15 2 3.02041 1.70771 3

6 -15 2 -0.2640 2.08959 2

-7 15 -2 1.20131 3.17197 1

7 -15 2 1.54673 1.91932 3

7 -15 2 0.05535 2.23946 2

-8 15 -2 2.04343 3.45142 1

8 -15 2 -1.2437 2.56411 2

-9 -14 2 2.14987 3.19248 5

-8 -14 2 -0.6946 2.63439 5

-7 -14 2 1.47738 2.73409 5

-6 -14 2 4.04259 2.26310 5

-6 -14 2 2.87781 2.00236 2

-5 -14 2 45.5142 3.55858 5

-5 -14 2 41.2113 3.48414 2

-4 -14 2 6.84484 1.82694 2

-4 -14 2 10.5141 1.96450 5

3 14 -2 1.46790 1.56085 1

-3 -14 2 0.16528 1.53637 5

-3 -14 2 1.01940 1.70870 2

2 14 -2 4.51539 1.74581 1

-2 -14 2 5.50517 1.52233 5

-2 -14 2 5.14567 1.65502 2

1 14 -2 -1.6809 1.72754 1

-1 -14 2 0.31867 1.30668 5

-1 -14 2 2.34512 1.54189 2

0 14 -2 2.13267 1.77668 1

0 -14 2 0.67339 1.72402 2

-1 14 -2 2.44537 1.91101 1

1 -14 2 2.25489 1.56164 2

-2 14 -2 9.16053 2.19461 1

-3 14 -2 32.3736 4.03559 1

3 -14 2 40.0272 3.34067 2

-4 14 -2 7.21521 2.39531 1

4 -14 2 7.07095 1.51855 3

4 -14 2 9.00466 2.04521 2

-5 14 -2 1.31024 2.42719 1

5 -14 2 5.29897 1.54949 3

5 -14 2 1.30117 1.85626 2

-6 14 -2 -1.2333 2.95612 1

6 -14 2 0.45435 1.69078 3

6 -14 2 1.43776 1.96551 2

-7 14 -2 4.01095 3.00443 1

7 -14 2 0.96054 2.10229 2

7 -14 2 4.96398 2.07208 3

-8 14 -2 -3.0021 3.03664 1

8 -14 2 4.26121 2.27321 3

8 -14 2 -0.3010 2.18380 2

-9 14 -2 0.34573 3.42534 1

9 -14 2 4.40277 2.55472 3

10 13 -2 3.23068 2.37609 3

-10 -13 2 -0.6210 3.19209 5

-9 -13 2 5.90175 2.93589 5

-8 -13 2 1.89427 2.57489 5

-7 -13 2 15.0331 2.57458 5

-6 -13 2 3.14403 1.92913 2

-6 -13 2 5.61159 2.03771 5

-5 -13 2 5.37508 1.80868 5

-5 -13 2 6.05305 1.77206 2

4 13 -2 0.51992 1.28739 1

-4 -13 2 0.75841 1.43738 2

-4 -13 2 0.78151 1.60506 5

3 13 -2 41.6564 2.90858 1

-3 -13 2 45.9342 2.82469 5

-3 -13 2 42.6591 3.06003 2

2 13 -2 14.2163 1.75289 1

-2 -13 2 13.6269 1.60315 5

-2 -13 2 12.2031 1.75374 2

1 13 -2 46.4333 3.13261 1

-1 -13 2 48.5576 2.51939 5

-1 -13 2 43.7613 3.09523 2

0 13 -2 9.01299 1.73858 1

0 -13 2 10.9794 1.78117 2

0 -13 2 9.29965 1.29278 5

-1 13 -2 0.62108 1.67902 1

1 -13 2 1.88453 1.47775 2

-2 13 -2 2.06950 1.86530 1

2 -13 2 5.14557 1.68450 2

-3 13 -2 1.62170 1.90060 1

3 -13 2 3.15511 1.18020 3

3 -13 2 2.49542 1.67503 2

-4 13 -2 8.33899 2.15606 1

4 -13 2 10.1878 1.79202 2

4 -13 2 10.7141 1.53335 3

-5 13 -2 2.37689 2.12001 1

5 -13 2 2.01626 1.41355 3

5 -13 2 2.11308 1.77695 2

6 -13 2 11.2427 1.89209 3

6 -13 2 10.0351 2.04768 2

7 -13 2 11.2456 2.13535 3

-8 13 -2 2.29604 2.89426 1

8 -13 2 6.04379 2.29680 3

-9 13 -2 0.80024 3.13480 1

9 -13 2 -0.5713 2.26994 3

-10 13 -2 1.01146 3.54901 1

10 -13 2 1.04509 2.72903 3

10 12 -2 5.98689 2.28489 3

-10 -12 2 12.3390 3.36056 5

-9 -12 2 -0.7571 2.70488 5

-8 -12 2 4.42262 2.27225 5

-7 -12 2 0.15739 1.98833 5

-6 -12 2 0.36805 1.77496 5

-6 -12 2 0.32224 1.56987 2

-5 -12 2 1.71702 1.67523 5

-5 -12 2 0.05289 1.49236 2

-4 -12 2 -0.2240 1.34681 2

-4 -12 2 0.22447 1.39641 5

3 12 -2 146.599 5.28992 1

3 12 -2 133.750 4.93697 1

-3 -12 2 142.546 5.50960 2

-3 -12 2 148.555 5.33007 5

2 12 -2 115.099 4.56663 1

2 12 -2 130.167 4.95166 1

-2 -12 2 131.989 5.06081 2

-2 -12 2 137.306 4.78022 5

1 12 -2 13.5896 1.77837 1

1 12 -2 13.4440 1.65865 1

-1 -12 2 14.6047 1.75420 5

-1 -12 2 12.9447 1.60943 2

0 12 -2 28.2034 2.07565 1

0 12 -2 30.1414 2.62316 1

0 -12 2 30.0950 2.45436 2

-1 12 -2 167.330 6.32274 1

-1 12 -2 187.196 6.73747 1

1 -12 2 186.321 6.59874 2

-2 12 -2 23.3811 2.84970 1

2 -12 2 21.8618 2.41716 2

-3 12 -2 43.1133 3.37083 1

3 -12 2 46.2348 2.90000 3

3 -12 2 46.0262 2.93529 2

-4 12 -2 0.56464 1.67416 1

4 -12 2 1.91789 1.20365 3

4 -12 2 1.02279 1.42784 2

-5 12 -2 7.17251 1.97517 1

5 -12 2 8.83287 1.71432 2

5 -12 2 9.56744 1.57053 3

-6 12 -2 3.62844 2.18434 1

6 -12 2 5.33864 1.64380 3

-7 12 -2 -0.7548 2.26924 1

7 -12 2 1.01804 1.64613 3

8 -12 2 13.6015 3.06148 1

8 -12 2 15.0119 2.40304 3

-9 12 -2 9.89586 3.10486 1

9 -12 2 4.34705 2.40596 3

-10 12 -2 2.11260 3.40567 1

10 -12 2 3.29959 2.77623 3

11 11 -2 -0.1899 2.17232 3

-11 -11 2 1.43832 3.33080 5

10 11 -2 1.90064 1.89811 3

-10 -11 2 0.00769 2.71881 5

-8 -11 2 6.48057 2.24228 5

-7 -11 2 8.30216 2.04017 5

-6 -11 2 13.6412 2.00567 5

-6 -11 2 11.3986 1.73601 2

-5 -11 2 139.223 5.36957 5

-5 -11 2 135.887 5.35658 2

4 11 -2 71.8171 3.04757 6

4 11 -2 74.3646 3.06354 1

-4 -11 2 71.4312 3.44751 2

-4 -11 2 76.5906 3.37540 5

3 11 -2 124.978 4.45398 6

3 11 -2 121.679 4.38417 1

3 11 -2 127.300 4.74658 1

-3 -11 2 132.819 4.76953 5

-3 -11 2 130.600 4.93608 2

2 11 -2 -2.5972 1.17359 1

-2 -11 2 -1.3525 1.00781 2

-2 -11 2 -0.5799 1.03212 5

1 11 -2 375.861 12.2009 1

1 11 -2 377.772 12.3957 1

-1 -11 2 383.229 12.5016 2

-1 -11 2 381.006 12.2215 5

0 11 -2 4.88285 1.02223 1

0 11 -2 5.18300 1.34813 1

0 -11 2 5.29748 1.25576 2

0 -11 2 5.84542 1.04266 5

-1 11 -2 33.2154 2.08423 1

-1 11 -2 35.7923 2.67551 1

1 -11 2 39.9402 2.47019 2

-2 11 -2 18.5504 1.82486 1

-2 11 -2 17.8381 2.44953 1

2 -11 2 14.2201 2.10112 2

2 -11 2 14.3073 1.95323 3

-3 11 -2 268.657 9.64179 1

-3 11 -2 292.973 9.78917 4

3 -11 2 277.022 9.38103 3

3 -11 2 267.526 9.43004 2

-4 11 -2 1.70056 1.54396 1

4 -11 2 1.32494 1.33056 2

4 -11 2 -0.2311 1.13318 3

-5 11 -2 13.7418 2.15339 1

5 -11 2 15.0631 2.23168 1

5 -11 2 14.7122 1.72794 3

-6 11 -2 0.74999 1.89260 1

6 -11 2 3.18526 1.50708 3

6 -11 2 0.84031 1.90010 1

-7 11 -2 16.3420 2.64667 1

7 -11 2 15.0025 2.48814 1

7 -11 2 12.1704 2.04686 3

7 -11 2 12.9587 1.88287 6

-8 11 -2 0.73526 2.37234 1

8 -11 2 -1.1239 2.09551 1

8 -11 2 -1.7423 1.61506 6

8 -11 2 -0.6178 1.95792 3

9 -11 2 1.43226 2.39599 3

9 -11 2 -0.3244 1.93670 6

9 -11 2 1.08369 2.58705 1

-10 11 -2 9.90375 3.21094 1

10 -11 2 2.60250 2.99366 1

10 -11 2 2.72518 3.03952 3

-11 11 -2 3.71043 3.62398 1

11 -11 2 -1.2574 3.46240 1

11 -11 2 -3.9149 3.10900 3

12 10 -2 2.82142 2.49098 3

11 10 -2 2.68825 2.11415 3

-11 -10 2 2.77680 3.27290 5

-10 -10 2 0.55504 2.57334 5

-9 -10 2 11.9999 2.59112 5

-8 -10 2 10.8911 2.22205 5

-7 -10 2 81.9110 4.15414 5

-6 -10 2 6.98849 1.76407 5

-5 -10 2 11.8048 1.59951 5

-5 -10 2 10.2162 1.52019 2

4 10 -2 57.4580 2.65516 6

-4 -10 2 59.7247 2.96660 5

-4 -10 2 60.2985 2.96563 2

3 10 -2 86.8184 3.21902 6

3 10 -2 87.5009 3.14711 1

3 10 -2 84.5487 3.47391 1

-3 -10 2 87.1187 3.67385 2

-3 -10 2 90.3252 3.52465 5

2 10 -2 290.757 9.34542 1

2 10 -2 287.716 9.51974 1

-2 -10 2 291.077 9.51545 5

-2 -10 2 291.463 9.68220 2

1 10 -2 3.90810 1.14289 1

1 10 -2 3.78623 0.80228 1

-1 -10 2 3.34959 1.27059 2

-1 -10 2 5.52615 1.17093 5

0 10 -2 371.417 11.5880 1

0 10 -2 360.190 11.7538 1

0 10 -2 343.499 11.6662 3

0 -10 2 356.969 11.4805 5

0 -10 2 362.895 11.7862 2

-1 10 -2 5.60978 1.28692 1

-1 10 -2 4.15085 0.92972 1

-1 10 -2 2.69825 0.87973 3

1 -10 2 4.64088 1.23771 2

-2 10 -2 170.617 6.15826 1

-2 10 -2 182.466 6.43100 1

2 -10 2 167.598 6.43121 4

2 -10 2 187.601 6.31767 2

-3 10 -2 48.5266 3.27436 4

-3 10 -2 46.6293 3.07240 1

3 -10 2 48.9016 2.82250 3

3 -10 2 51.0356 2.76119 2

-4 10 -2 35.6407 3.30507 4

-4 10 -2 36.3515 3.00552 1

4 -10 2 35.0868 2.99620 1

4 -10 2 40.9518 2.69450 3

-5 10 -2 84.1354 4.22314 1

-5 10 -2 84.3419 4.59520 4

5 -10 2 76.1020 4.12517 1

5 -10 2 80.7357 3.93754 3

-6 10 -2 19.9803 3.21005 1

6 -10 2 14.6524 1.68316 6

6 -10 2 17.1954 2.18756 1

6 -10 2 13.8395 1.79732 3

-7 10 -2 4.55751 2.00135 1

7 -10 2 -0.2864 1.77005 1

7 -10 2 2.83245 1.70953 3

7 -10 2 2.63231 1.48213 6

-8 10 -2 8.64204 2.44225 1

8 -10 2 6.50346 2.17898 1

8 -10 2 6.49298 2.05745 3

8 -10 2 8.90992 1.86948 6

-9 10 -2 7.24756 2.67707 1

9 -10 2 6.22262 2.01642 6

9 -10 2 2.09867 2.19712 3

9 -10 2 1.90675 2.30055 1

-10 10 -2 0.06476 2.96108 1

10 -10 2 2.62811 2.79417 1

10 -10 2 0.00062 2.70470 3

10 -10 2 -0.9837 2.05773 6

-11 10 -2 3.44740 3.32063 1

11 -10 2 -5.2324 2.93521 3

11 -10 2 1.11926 2.97293 1

11 -10 2 -0.8208 2.51843 6

12 9 -2 1.71988 2.34916 3

-12 -9 2 0.38259 3.23521 5

11 9 -2 -0.1494 1.75050 3

-11 -9 2 5.03860 3.00552 5

10 9 -2 29.6091 3.59239 3

-9 -9 2 65.5505 4.34995 5

-8 -9 2 5.77672 1.91572 5

-7 -9 2 20.2928 2.58439 5

-6 -9 2 -0.0178 1.44394 5

-5 -9 2 34.7052 2.42157 5

-5 -9 2 34.2726 2.36894 2

4 9 -2 380.587 12.3844 6

-4 -9 2 381.541 12.5490 2

-4 -9 2 389.100 12.5360 5

3 9 -2 86.7781 3.07568 6

3 9 -2 86.7820 2.93831 1

3 9 -2 81.4393 3.30650 1

-3 -9 2 79.8604 3.31883 5

-3 -9 2 77.8849 3.39913 2

2 9 -2 853.127 27.4767 3

2 9 -2 890.817 27.2829 1

-2 -9 2 870.307 27.5458 2

1 9 -2 415.869 13.2756 3

1 9 -2 403.093 13.1198 1

1 9 -2 417.225 13.2267 1

-1 -9 2 406.282 13.1573 5

-1 -9 2 420.602 13.3476 2

0 9 -2 1224.42 38.3868 1

0 9 -2 1246.96 38.3674 3

0 9 -2 1235.73 38.2891 1

0 -9 2 1221.12 38.4337 2

0 -9 2 1191.03 38.2068 5

-1 9 -2 84.0761 3.13753 1

-1 9 -2 78.2283 3.20166 3

-1 9 -2 84.8448 3.38681 1

1 -9 2 81.1082 3.36826 2

-2 9 -2 790.700 24.1022 4

-2 9 -2 747.835 23.8114 3

-2 9 -2 742.983 23.9129 1

-2 9 -2 757.987 24.0831 1

2 -9 2 736.653 24.0584 4

2 -9 2 758.409 23.9532 3

2 -9 2 751.601 23.9785 2

-3 9 -2 12.5879 1.50680 3

-3 9 -2 13.1212 2.04182 1

3 -9 2 10.7352 1.67734 1

-4 9 -2 51.4411 3.28613 4

4 -9 2 47.5621 2.86541 3

4 -9 2 41.9842 2.98332 1

-5 9 -2 2.43282 2.29213 4

5 -9 2 4.76322 1.13098 6

5 -9 2 6.12322 1.28956 3

5 -9 2 3.44692 1.32001 1

-6 9 -2 30.1912 3.64339 4

6 -9 2 27.7868 2.88639 1

6 -9 2 26.4516 2.32412 6

6 -9 2 26.5415 2.97722 3

7 -9 2 35.3729 2.46043 6

7 -9 2 38.7888 3.60793 3

7 -9 2 41.7765 3.34719 1

8 -9 2 11.2423 2.15535 1

8 -9 2 12.6156 2.16712 3

-9 9 -2 2.68837 2.27235 1

9 -9 2 2.46136 1.90479 1

9 -9 2 1.20210 2.05955 3

-10 9 -2 5.45395 2.82912 1

10 -9 2 4.47094 2.44041 1

10 -9 2 2.69925 2.60425 3

10 -9 2 3.17914 2.06329 6

-11 9 -2 5.23973 3.25651 1

11 -9 2 -0.6449 2.69313 1

11 -9 2 2.01271 2.22894 6

11 -9 2 -4.3901 2.94914 3

-12 9 -2 5.35858 3.52040 1

12 -9 2 2.54629 2.85859 6

12 -9 2 -6.6015 3.36242 3

12 -9 2 2.38334 3.12879 1

12 8 -2 2.19728 2.07989 3

12 8 -2 -1.5204 1.80418 1

-12 -8 2 -2.0807 3.18846 5

11 8 -2 4.51110 1.78334 3

11 8 -2 5.70584 1.65134 1

-11 -8 2 6.81609 2.77446 5

10 8 -2 0.76302 1.55954 3

10 8 -2 2.81431 1.31292 1

-10 -8 2 3.69882 2.13392 5

9 8 -2 26.8570 2.28161 1

9 8 -2 25.6022 2.75493 3

-9 -8 2 22.1404 3.22346 5

-8 -8 2 1.53980 1.58224 5

-7 -8 2 113.517 4.73770 5

-6 -8 2 5.14121 1.39900 5

-5 -8 2 27.4187 2.06159 2

-5 -8 2 17.6657 2.10663 5

4 8 -2 136.771 4.73975 6

-4 -8 2 129.075 4.84395 2

-4 -8 2 136.326 4.88449 5

3 8 -2 2.86661 0.73724 6

-3 -8 2 2.78533 0.99524 2

-3 -8 2 2.47320 0.98465 5

2 8 -2 420.057 13.4187 5

2 8 -2 422.316 13.2677 6

2 8 -2 411.455 13.3466 1

2 8 -2 432.864 13.2675 1

2 8 -2 431.846 13.4641 3

-2 -8 2 419.396 13.5222 2

-2 -8 2 404.147 13.4516 5

1 8 -2 0.42063 0.62703 3

1 8 -2 0.86645 0.55272 1

-1 -8 2 -0.8270 0.76966 2

-1 -8 2 -0.0135 0.71426 5

0 8 -2 740.783 23.0902 1

0 8 -2 750.455 23.1647 3

0 -8 2 729.879 23.2311 2

0 -8 2 718.383 23.0247 5

-1 8 -2 2.44621 0.69574 3

-1 8 -2 1.57728 0.88546 1

-1 8 -2 2.06059 0.71001 1

1 -8 2 1.94844 0.67477 3

1 -8 2 2.53261 0.85444 2

-2 8 -2 171.085 6.07571 4

-2 8 -2 170.412 5.69573 3

-2 8 -2 171.797 5.81051 1

2 -8 2 172.697 5.88714 2

2 -8 2 167.073 6.06872 4

2 -8 2 170.620 6.00030 1

2 -8 2 166.580 5.84493 3

-3 8 -2 52.9359 2.98805 4

-3 8 -2 53.2136 2.54836 1

-3 8 -2 56.5809 2.31138 3

3 -8 2 52.0055 2.83085 1

3 -8 2 56.0450 2.73262 3

3 -8 2 52.3571 3.04929 4

-4 8 -2 30.7401 2.66670 4

4 -8 2 29.2792 2.28872 1

4 -8 2 28.7283 1.62628 6

-5 8 -2 172.118 6.71009 4

5 -8 2 170.897 6.25545 3

5 -8 2 169.459 6.39836 1

-6 8 -2 -1.9621 2.19006 4

6 -8 2 1.13385 1.20951 3

6 -8 2 0.88762 1.19603 1

7 -8 2 58.3300 3.61826 1

7 -8 2 54.6552 3.75676 3

8 -8 2 9.79400 1.86164 1

8 -8 2 9.86842 1.81229 3

9 -8 2 33.2859 3.69426 1

9 -8 2 27.7578 4.21526 3

10 -8 2 1.40463 1.99528 1

10 -8 2 0.06131 2.24564 3

-11 8 -2 4.73514 2.99526 1

11 -8 2 2.14766 3.12238 3

11 -8 2 2.15017 2.46161 1

-12 8 -2 3.07211 3.37013 1

12 -8 2 -4.5653 3.14479 3

12 -8 2 0.90041 2.74415 1

13 7 -2 -1.9211 2.17599 3

13 7 -2 1.72173 2.31912 1

-13 -7 2 1.82594 3.71953 5

12 7 -2 2.51158 1.97114 1

12 7 -2 2.27064 2.02712 3

-12 -7 2 1.84373 3.12438 5

11 7 -2 18.7397 2.85363 1

-11 -7 2 15.8558 3.06819 5

10 7 -2 11.2181 1.57069 1

10 7 -2 7.12900 1.60445 3

-10 -7 2 13.9426 2.26813 5

9 7 -2 2.60820 1.17412 1

9 7 -2 4.14084 1.27667 3

-9 -7 2 2.84234 1.82801 5

8 7 -2 20.2458 2.01432 1

-8 -7 2 25.1648 2.67022 5

7 7 -2 238.006 7.74589 1

-7 -7 2 231.839 8.20311 5

6 7 -2 83.8643 3.87982 4

-6 -7 2 91.4462 3.77785 5

-5 -7 2 59.6836 2.87492 5

-5 -7 2 66.3962 2.84827 2

-4 -7 2 1.29875 1.08398 5

-4 -7 2 2.46664 0.85424 2

3 7 -2 184.968 6.20200 5

-3 -7 2 173.464 6.43730 5

-3 -7 2 197.137 6.29303 2

2 7 -2 123.158 4.12057 6

2 7 -2 124.337 4.26666 5

2 7 -2 130.599 4.07696 1

2 7 -2 122.346 4.18561 1

-2 -7 2 118.715 4.29444 5

1 7 -2 749.365 23.5035 5

1 7 -2 766.480 23.5657 3

1 7 -2 795.673 23.4653 1

1 7 -2 727.280 23.4917 1

1 7 -2 747.437 23.5966 2

-1 -7 2 746.111 23.6551 2

-1 -7 2 714.811 23.5044 5

0 7 -2 94.2050 3.55140 1

0 7 -2 108.636 3.54645 3

0 7 -2 98.9201 3.42596 1

0 -7 2 97.6735 3.74783 2

0 -7 2 96.8875 3.37350 5

-1 7 -2 51.4352 2.00856 1

-1 7 -2 43.6604 2.28637 4

-1 7 -2 51.5053 2.02531 3

1 -7 2 45.9598 2.34620 4

1 -7 2 46.6095 2.14393 1

1 -7 2 48.3754 2.04590 3

-2 7 -2 0.80464 0.65015 1

-2 7 -2 1.89149 0.54499 3

2 -7 2 1.14426 0.70323 3

2 -7 2 0.43224 1.26828 4

2 -7 2 2.19161 0.83167 1

-3 7 -2 321.556 10.3355 1

-3 7 -2 316.481 10.6038 4

-3 7 -2 320.662 10.1433 3

3 -7 2 309.705 10.4357 1

3 -7 2 308.527 10.5322 4

3 -7 2 316.047 10.3216 3

-4 7 -2 12.1928 2.13365 4

-4 7 -2 12.9719 1.33107 3

-5 7 -2 319.338 10.8999 4

5 -7 2 316.941 10.5087 3

5 -7 2 306.802 10.5910 1

-6 7 -2 13.8813 2.56311 4

6 -7 2 8.83579 1.44309 1

6 -7 2 10.3124 1.36190 3

-7 7 -2 38.2812 3.78599 4

7 -7 2 38.3681 2.80828 1

7 -7 2 40.1450 2.97311 3

8 -7 2 2.61290 1.40179 1

8 -7 2 1.31402 1.45507 3

9 -7 2 46.0735 3.86291 3

9 -7 2 41.4871 3.52518 1

10 -7 2 1.71530 1.83645 1

10 -7 2 3.83313 1.95673 3

11 -7 2 2.00485 2.13787 1

11 -7 2 1.22887 2.34080 3

-12 7 -2 -2.8143 3.95214 1

12 -7 2 2.25985 2.47338 1

12 -7 2 -0.6234 2.96704 3

13 6 -2 2.02364 2.33198 1

13 6 -2 0.90127 2.17980 3

-13 -6 2 -0.3348 3.38344 5

12 6 -2 -0.4721 1.82181 3

12 6 -2 3.94205 1.98205 1

-12 -6 2 -1.4969 2.72443 5

11 6 -2 0.80424 1.53804 3

11 6 -2 1.04274 1.56423 1

-11 -6 2 2.14646 2.33995 5

10 6 -2 3.70913 1.33493 3

10 6 -2 7.00643 1.49788 1

-10 -6 2 5.61530 2.16472 5

9 6 -2 -0.6028 1.14393 3

9 6 -2 -0.9174 1.13170 1

-9 -6 2 1.03777 1.48536 5

8 6 -2 32.3673 2.10179 1

8 6 -2 29.4248 2.31448 3

-8 -6 2 22.3331 2.62287 5

7 6 -2 21.8973 1.73936 1

-7 -6 2 24.3267 2.12961 5

6 6 -2 113.803 4.63246 4

-6 -6 2 120.602 4.52901 5

5 6 -2 4.87911 1.35776 4

-5 -6 2 5.07812 1.06877 2

-5 -6 2 4.46265 1.10488 5

-4 -6 2 40.4684 2.11233 5

-4 -6 2 42.5008 2.04735 2

3 6 -2 205.742 6.55784 5

3 6 -2 193.846 6.53568 2

-3 -6 2 193.868 6.57221 5

2 6 -2 0.08630 0.57896 5

2 6 -2 1.37575 0.37651 1

2 6 -2 1.69744 0.66195 2

2 6 -2 0.01974 0.50804 1

-2 -6 2 0.73386 0.65863 2

-2 -6 2 0.49871 0.64509 5

1 6 -2 5.38330 0.79780 1

1 6 -2 8.16553 0.62160 1

1 6 -2 5.50543 0.83144 3

1 6 -2 5.87924 0.80736 5

-1 -6 2 7.40743 0.73985 5

-1 -6 2 5.59400 0.77712 2

0 6 -2 146.450 4.60939 3

0 6 -2 130.278 4.61147 1

0 -6 2 135.365 4.48806 5

0 -6 2 138.656 4.67281 2

-1 6 -2 694.800 21.3908 1

-1 6 -2 699.072 21.3401 3

-1 6 -2 668.118 21.4758 4

1 -6 2 680.789 21.4345 2

1 -6 2 682.091 21.4963 4

1 -6 2 681.865 21.4373 1

1 -6 2 654.072 21.3569 3

-2 6 -2 16.6964 1.05216 3

-2 6 -2 15.7747 1.21398 1

2 -6 2 17.7419 1.71137 4

2 -6 2 18.7243 1.36789 3

2 -6 2 14.1939 1.35594 1

-3 6 -2 164.392 5.34328 3

-3 6 -2 156.726 5.75675 4

-3 6 -2 166.312 5.55405 1

3 -6 2 160.952 5.51056 3

3 -6 2 155.450 5.58882 1

3 -6 2 162.231 5.79081 4

-4 6 -2 68.0729 2.99604 1

-4 6 -2 65.0493 2.56749 3

4 -6 2 60.3804 2.88898 1

4 -6 2 62.3555 2.81414 3

-5 6 -2 240.518 8.45368 4

5 -6 2 229.239 8.00054 3

5 -6 2 238.061 8.12277 1

-6 6 -2 115.000 5.18084 4

6 -6 2 114.835 4.51978 3

6 -6 2 113.249 4.58183 1

-7 6 -2 26.3003 3.30204 4

7 -6 2 28.1103 2.45178 3

7 -6 2 30.3234 2.39865 1

8 -6 2 35.9267 3.10599 3

8 -6 2 36.5921 2.92297 1

9 -6 2 19.7803 2.56449 1

9 -6 2 12.3833 1.84621 3

10 -6 2 -1.1323 1.58560 1

10 -6 2 0.87463 1.80946 3

11 -6 2 -3.0338 2.18918 3

-12 6 -2 4.14739 2.96021 1

12 -6 2 2.26761 2.79462 3

12 -6 2 5.82250 2.41226 1

-13 6 -2 4.06427 3.47655 1

13 -6 2 -5.1873 3.13451 3

13 -6 2 2.86501 2.81388 1

13 5 -2 4.33804 2.41992 1

13 5 -2 0.14154 2.70120 3

-13 -5 2 -0.8514 3.20618 5

12 5 -2 1.26026 1.93981 1

12 5 -2 0.55618 1.76401 3

-12 -5 2 0.10925 2.62835 5

11 5 -2 2.78636 1.61270 1

11 5 -2 1.16564 1.49036 3

-11 -5 2 3.75333 2.09866 5

10 5 -2 -0.8980 1.37260 1

10 5 -2 1.68851 1.21144 3

-10 -5 2 0.56017 1.72212 5

9 5 -2 30.0267 2.53840 1

9 5 -2 26.4512 2.47494 3

-9 -5 2 30.6130 2.72399 5

8 5 -2 59.2677 2.70616 1

8 5 -2 54.9075 2.84717 3

-8 -5 2 49.8115 2.25971 1

-8 -5 2 55.0423 2.99998 5

7 5 -2 53.9529 2.42329 1

-7 -5 2 50.1621 2.81503 5

6 5 -2 5.41446 1.38272 4

-6 -5 2 5.32376 1.17407 5

5 5 -2 45.6293 1.89675 1

5 5 -2 48.7419 2.44357 4

-5 -5 2 49.1450 2.45236 5

4 5 -2 117.303 4.15272 5

3 5 -2 386.839 11.9535 5

3 5 -2 383.955 11.9259 2

-3 -5 2 349.239 11.9449 5

-3 -5 2 375.282 11.9465 2

2 5 -2 418.635 12.8110 2

2 5 -2 417.942 12.7856 5

-2 -5 2 395.363 12.8183 2

-2 -5 2 382.961 12.7847 5

1 5 -2 777.249 24.5640 1

1 5 -2 775.763 24.6030 5

1 5 -2 850.867 24.7106 1

1 5 -2 799.862 24.6342 3

1 5 -2 799.675 24.6778 2

-1 -5 2 777.102 24.6891 2

-1 -5 2 734.089 24.6101 5

0 5 -2 118.101 3.72218 3

0 5 -2 107.371 3.79939 4

0 5 -2 114.254 3.68244 1

0 -5 2 114.505 3.86798 4

0 -5 2 103.302 3.62178 5

-1 5 -2 159.504 5.48337 4

-1 5 -2 168.164 5.29108 3

-1 5 -2 165.660 5.36053 1

1 -5 2 163.282 5.51166 4

1 -5 2 157.178 5.38052 1

1 -5 2 162.892 5.32161 3

-2 5 -2 60.7172 2.14610 3

-2 5 -2 59.8830 2.31919 1

-2 5 -2 58.4733 2.54986 4

2 -5 2 57.4564 2.31000 3

2 -5 2 58.6687 2.56588 4

2 -5 2 57.4677 2.34737 1

-3 5 -2 89.7356 2.99761 3

-3 5 -2 87.7293 3.49695 4

-3 5 -2 81.2781 3.24727 1

3 -5 2 83.2820 3.14699 3

3 -5 2 76.0783 3.21964 1

3 -5 2 85.6415 3.48146 4

-4 5 -2 85.5826 3.24186 1

-4 5 -2 73.7731 3.75384 4

-4 5 -2 87.7952 3.01973 3

4 -5 2 79.3846 3.47537 4

4 -5 2 80.4940 3.10325 3

4 -5 2 80.8551 3.47066 1

-5 5 -2 91.4974 3.59018 3

-5 5 -2 87.1334 4.16497 4

5 -5 2 95.4247 3.75330 1

-6 5 -2 134.822 5.73813 4

6 -5 2 138.847 5.08720 3

6 -5 2 145.465 5.24707 1

-7 5 -2 138.685 5.95062 4

7 -5 2 136.870 5.14640 3

7 -5 2 142.338 5.30300 1

7 -5 2 125.095 4.69486 5

8 -5 2 19.3274 2.43126 3

8 -5 2 22.4282 2.28564 1

9 -5 2 2.18512 1.25095 1

9 -5 2 -0.8243 1.20642 3

10 -5 2 5.44296 1.71173 1

10 -5 2 4.44092 1.66557 3

11 -5 2 0.96255 1.96234 3

11 -5 2 2.69939 1.80379 1

12 -5 2 0.09726 2.10891 1

12 -5 2 -1.9387 2.55952 3

13 -5 2 -5.9342 3.02150 3

13 -5 2 1.59186 2.52724 1

13 4 -2 0.53391 2.72420 3

13 4 -2 2.84357 2.37019 1

-13 -4 2 9.18008 3.18832 5

12 4 -2 1.09358 1.65085 3

12 4 -2 0.92287 1.89271 1

-12 -4 2 4.38688 2.61713 5

11 4 -2 4.05201 1.64213 1

11 4 -2 2.54025 1.37216 3

-11 -4 2 3.34973 1.99878 5

10 4 -2 2.32385 1.36372 1

10 4 -2 2.09653 1.15666 3

-10 -4 2 3.68807 1.73544 5

9 4 -2 23.2921 2.34931 3

9 4 -2 27.4037 2.32105 1

-9 -4 2 23.2789 1.88290 1

-9 -4 2 24.8944 2.44303 5

8 4 -2 -0.5824 0.92529 1

8 4 -2 0.03363 0.84063 3

-8 -4 2 0.11420 1.18053 5

7 4 -2 37.9349 2.16612 1

7 4 -2 39.2627 2.27668 3

-7 -4 2 42.1798 2.49366 5

6 4 -2 -1.8725 1.23807 4

-6 -4 2 2.32612 1.14417 5

5 4 -2 29.6094 1.98469 4

-5 -4 2 33.6142 1.88289 5

4 4 -2 422.103 12.8736 2

4 4 -2 416.093 12.9288 5

-4 -4 2 386.338 12.9129 5

-4 -4 2 389.555 12.8886 2

3 4 -2 112.789 3.84409 2

3 4 -2 115.966 3.88241 5

-3 -4 2 111.006 3.85906 2

2 4 -2 637.178 19.4823 5

2 4 -2 635.937 19.4963 2

-2 -4 2 588.894 19.4929 5

-2 -4 2 622.798 19.5086 2

1 4 -2 181.303 5.47629 1

1 4 -2 178.116 5.61205 2

1 4 -2 174.695 5.55371 5

-1 -4 2 173.920 5.62592 2

-1 -4 2 157.793 5.55329 5

0 4 -2 469.914 14.0993 1

0 4 -2 450.273 14.0812 3

0 4 -2 428.025 14.1676 4

0 -4 2 458.694 14.0924 1

0 -4 2 452.555 14.2023 4

0 -4 2 435.481 14.1462 2

-1 4 -2 1047.92 32.6426 4

-1 4 -2 1089.08 32.5806 1

-1 4 -2 1011.21 32.3594 3

1 -4 2 1018.46 32.4354 4

1 -4 2 1023.81 32.6584 1

1 -4 2 1024.68 32.5059 3

-2 4 -2 1367.81 41.2994 3

-2 4 -2 1320.97 41.1080 1

-2 4 -2 1335.44 41.5397 4

2 -4 2 1294.55 41.1933 4

2 -4 2 1310.27 41.3521 3

2 -4 2 1263.11 41.5321 1

-3 4 -2 1092.14 34.3813 4

-3 4 -2 1117.49 34.1006 3

-3 4 -2 1115.99 34.2425 1

3 -4 2 1089.54 34.3961 3

3 -4 2 1037.82 34.2276 1

3 -4 2 1093.81 34.3576 4

-4 4 -2 19.1559 1.50987 1

-4 4 -2 19.2196 1.27983 3

4 -4 2 17.1284 1.90737 4

4 -4 2 20.6375 1.49643 1

-5 4 -2 38.9144 2.69812 4

-5 4 -2 33.9091 1.93910 3

5 -4 2 43.4253 1.69720 5

5 -4 2 39.3197 2.16369 3

5 -4 2 37.3904 2.19826 1

-6 4 -2 51.0359 3.47745 4

6 -4 2 65.7581 2.91162 1

6 -4 2 63.0603 2.79647 3

6 -4 2 66.2739 2.47734 5

-7 4 -2 16.1459 2.71402 4

7 -4 2 15.4535 1.87938 3

7 -4 2 12.6395 1.92017 1

7 -4 2 15.5992 1.59187 5

8 -4 2 21.0974 2.30349 1

8 -4 2 17.2507 2.22752 3

9 -4 2 4.83037 1.50503 1

9 -4 2 3.02977 1.32049 3

10 -4 2 2.56003 1.52620 3

10 -4 2 2.30847 1.37168 1

11 -4 2 4.95188 2.09438 3

11 -4 2 4.14882 1.82445 1

12 -4 2 3.59788 1.97362 1

12 -4 2 3.52750 2.37416 3

-13 4 -2 4.06208 2.93124 1

13 -4 2 3.88305 2.49296 1

13 -4 2 -2.2331 2.66781 3

13 3 -2 -0.7977 2.67269 3

13 3 -2 2.13840 2.25266 1

12 3 -2 2.52497 1.87849 1

12 3 -2 1.58615 1.86659 3

-12 -3 2 5.93296 2.79093 5

11 3 -2 2.57585 1.57430 1

11 3 -2 2.61722 1.33434 3

-11 -3 2 5.43930 1.98111 5

10 3 -2 1.00807 1.17188 3

10 3 -2 1.01128 1.40009 1

-10 -3 2 1.14509 1.18984 1

-10 -3 2 2.38232 1.53060 5

9 3 -2 -0.3130 0.96509 3

9 3 -2 -1.4192 1.06863 1

-9 -3 2 1.23360 1.29284 5

8 3 -2 0.56027 0.97699 1

8 3 -2 1.00766 0.89209 3

-8 -3 2 -0.6118 1.15080 5

7 3 -2 29.5082 1.87736 1

7 3 -2 31.9421 2.00300 3

-7 -3 2 28.1085 2.02208 5

-6 -3 2 404.998 13.0480 5

-6 -3 2 398.722 12.7697 1

5 3 -2 8.76651 1.34136 4

5 3 -2 6.71195 1.00017 1

-5 -3 2 6.89746 0.68955 1

4 3 -2 151.991 5.10087 5

4 3 -2 152.133 4.88681 1

4 3 -2 153.617 5.02132 2

-4 -3 2 145.018 5.09649 5

3 3 -2 199.625 6.37314 5

3 3 -2 202.773 6.33606 2

-3 -3 2 181.709 6.36781 5

-3 -3 2 198.972 6.34347 2

2 3 -2 729.055 22.4783 2

2 3 -2 729.562 22.4922 5

-2 -3 2 766.245 22.7323 2

-2 -3 2 651.152 22.4866 5

1 3 -2 808.451 24.9523 2

1 3 -2 811.904 25.0484 5

-1 -3 2 740.168 25.1902 5

-1 -3 2 837.374 25.2732 2

0 3 -2 62.9246 2.18102 3

0 3 -2 66.2138 2.21708 1

0 -3 2 63.6718 2.26940 2

0 -3 2 68.2367 2.20603 1

0 -3 2 68.0664 2.40450 4

-1 3 -2 1604.01 49.5769 1

-1 3 -2 1484.43 49.3866 3

1 -3 2 1576.91 49.3436 3

1 -3 2 1590.57 49.6581 1

1 -3 2 1614.70 49.5683 4

-2 3 -2 233.120 7.73949 4

-2 3 -2 241.585 7.62932 1

-2 3 -2 249.994 7.50534 3

2 -3 2 237.989 7.74722 4

2 -3 2 227.064 7.61083 1

2 -3 2 223.486 7.49114 3

-3 3 -2 108.703 3.60026 3

-3 3 -2 106.750 3.72936 1

-3 3 -2 100.749 3.91785 4

3 -3 2 103.268 3.91259 4

3 -3 2 100.141 3.57268 3

3 -3 2 116.417 3.75026 1

-4 3 -2 1082.75 34.3492 4

-4 3 -2 1068.41 34.1590 1

-4 3 -2 1105.15 34.0815 5

4 -3 2 1093.16 34.3260 4

4 -3 2 1041.95 34.0244 3

4 -3 2 1069.93 34.1689 1

4 -3 2 1152.22 33.9182 5

-5 3 -2 695.373 21.9916 5

-5 3 -2 715.160 22.5619 4

5 -3 2 734.945 22.0360 5

5 -3 2 661.624 22.3377 1

-6 3 -2 12.8551 1.23351 5

-6 3 -2 18.1373 2.43111 4

6 -3 2 13.7448 1.41016 5

6 -3 2 14.8632 1.64661 1

6 -3 2 13.4665 1.60447 3

-7 3 -2 14.7503 2.70493 4

7 -3 2 20.6671 1.51469 5

7 -3 2 20.9029 1.96341 1

7 -3 2 15.2056 1.72812 3

8 -3 2 31.1791 2.32691 3

8 -3 2 33.8197 2.59605 1

9 -3 2 0.64740 1.24549 1

9 -3 2 -0.6090 1.04942 3

10 -3 2 26.9276 3.09295 3

10 -3 2 28.5357 3.10965 1

11 -3 2 1.75311 1.56753 1

11 -3 2 -0.2125 1.69375 3

12 -3 2 0.60581 2.33086 3

12 -3 2 3.18347 1.97476 1

13 -3 2 0.61416 2.26732 1

13 -3 2 -3.8424 2.96135 3

14 2 -2 -5.5233 2.73831 1

14 2 -2 -1.1806 2.83501 3

13 2 -2 2.54434 2.33919 1

13 2 -2 2.00600 2.33505 3

12 2 -2 6.20899 3.50615 3

12 2 -2 5.72292 2.00883 1

11 2 -2 2.23766 1.42538 3

11 2 -2 2.75569 1.60782 1

-11 -2 2 2.10989 1.78387 5

10 2 -2 -0.5380 1.20074 1

10 2 -2 -0.3491 1.10516 3

-10 -2 2 -0.2662 1.23559 1

-10 -2 2 0.17232 1.47493 5

9 2 -2 129.677 4.93532 1

9 2 -2 119.620 4.92306 3

-9 -2 2 127.811 4.88436 5

8 2 -2 151.805 5.74523 3

8 2 -2 161.240 5.67488 1

-8 -2 2 160.583 5.71687 5

7 2 -2 11.8082 1.49125 1

7 2 -2 11.5460 1.70842 3

-7 -2 2 11.6822 1.70042 5

6 2 -2 83.5239 3.21974 3

-6 -2 2 82.0026 2.98249 1

-6 -2 2 80.7148 3.23227 5

5 2 -2 641.828 20.4531 1

5 2 -2 649.576 20.5572 5

5 2 -2 684.422 20.5054 2

5 2 -2 655.942 20.6693 4

-5 -2 2 643.713 20.5842 5

-5 -2 2 622.080 20.3518 1

4 2 -2 414.275 13.6061 1

4 2 -2 462.382 13.6780 2

4 2 -2 448.435 13.7249 3

-4 -2 2 414.190 13.5334 1

-4 -2 2 419.012 13.7264 5

3 2 -2 3275.49 105.330 1

3 2 -2 3665.25 105.644 2

3 2 -2 3402.93 105.777 5

-3 -2 2 3124.70 105.764 5

-3 -2 2 3399.28 105.146 1

-3 -2 2 3406.03 105.715 2

2 2 -2 782.780 24.1801 5

2 2 -2 796.074 24.1523 2

-2 -2 2 684.826 24.1718 5

-2 -2 2 832.823 24.4739 2

1 2 -2 127.624 4.07259 5

1 2 -2 135.513 4.08256 2

-1 -2 2 129.080 4.08066 2

-1 -2 2 116.048 4.07058 5

0 2 -2 5.13883 0.38048 1

0 2 -2 5.26314 0.64615 4

-2 2 -2 36.7826 1.57205 1

-2 2 -2 39.1013 1.77473 4

-2 2 -2 39.8187 1.44480 3

2 -2 2 38.0141 1.80340 4

2 -2 2 38.3714 1.41109 3

2 -2 2 34.6862 1.58458 1

-3 2 -2 747.145 22.8811 5

-3 2 -2 730.142 23.0106 4

-3 2 -2 701.555 22.8469 1

3 -2 2 707.342 22.6771 5

3 -2 2 720.847 22.9860 4

3 -2 2 761.957 22.9657 3

3 -2 2 719.569 22.8639 1

-4 2 -2 18.1130 1.66469 4

-4 2 -2 17.0061 1.34048 1

-4 2 -2 18.6330 0.95237 5

4 -2 2 17.7139 1.15633 3

4 -2 2 19.1914 1.62053 4

4 -2 2 17.3717 1.31077 1

4 -2 2 20.2949 1.06756 5

-5 2 -2 378.437 11.9318 5

5 -2 2 330.304 12.1953 3

5 -2 2 418.543 12.0070 5

-6 2 -2 27.0439 2.58566 4

-6 2 -2 29.7804 1.57846 5

6 -2 2 29.1711 1.82109 3

6 -2 2 32.2783 1.78642 5

6 -2 2 26.0115 1.96642 1

-7 2 -2 165.502 5.52751 5

-7 2 -2 170.799 6.42077 4

7 -2 2 150.224 5.68232 3

7 -2 2 162.668 5.82792 1

-8 2 -2 10.9316 1.60360 5

8 -2 2 7.58842 1.24269 1

8 -2 2 8.06493 1.05126 3

-9 2 -2 8.71686 1.40738 5

9 -2 2 10.2514 1.57035 1

9 -2 2 5.87978 1.18626 3

10 -2 2 1.49022 1.14938 3

10 -2 2 0.65962 1.44817 1

11 -2 2 12.5027 2.14321 1

11 -2 2 7.02276 2.05924 3

12 -2 2 0.50277 1.73168 1

12 -2 2 -0.3217 2.38046 3

13 -2 2 1.61267 2.96598 3

13 -2 2 -2.0945 2.15282 1

14 1 -2 5.81680 3.00438 1

14 1 -2 1.70718 3.47204 3

13 1 -2 -1.6420 2.23879 3

13 1 -2 0.47786 2.31251 1

12 1 -2 -0.3806 3.09915 3

12 1 -2 1.28997 1.78026 1

11 1 -2 4.28033 1.57380 1

11 1 -2 2.99910 1.58560 3

-11 -1 2 2.19664 1.68022 1

10 1 -2 1.93346 1.29131 1

10 1 -2 1.13702 1.22414 3

-10 -1 2 1.82863 1.34308 1

-10 -1 2 2.28096 1.46428 5

9 1 -2 4.45935 1.28843 1

9 1 -2 3.44806 1.04394 3

-9 -1 2 6.68332 1.41961 5

8 1 -2 35.7774 2.30495 3

8 1 -2 40.4551 2.39441 1

-8 -1 2 40.7300 2.28010 5

7 1 -2 4.36185 1.03147 1

7 1 -2 2.82723 0.96774 3

-7 -1 2 2.71761 1.12529 5

6 1 -2 2.70254 0.76955 3

6 1 -2 3.63225 0.83805 1

-6 -1 2 4.17683 0.91397 5

5 1 -2 356.195 11.9038 1

5 1 -2 402.715 11.9165 2

5 1 -2 343.466 12.1341 3

5 1 -2 391.915 11.9876 5

5 1 -2 380.158 12.1270 4

-5 -1 2 356.907 11.9398 5

-5 -1 2 376.464 11.8431 1

4 1 -2 1064.76 34.8798 1

4 1 -2 1137.54 35.1358 3

4 1 -2 1161.45 34.5523 2

4 1 -2 1101.57 34.5976 5

-4 -1 2 1054.58 34.9466 5

-4 -1 2 1117.52 34.6727 1

3 1 -2 993.014 30.0377 3

3 1 -2 956.801 29.4554 2

3 1 -2 914.276 29.4962 5

3 1 -2 943.336 29.8049 1

-3 -1 2 947.210 29.5963 1

-3 -1 2 911.141 29.8875 5

2 1 -2 0.83872 0.35157 5

2 1 -2 1.14995 0.30091 2

2 1 -2 0.65161 0.25600 3

2 1 -2 0.69150 0.21658 1

-2 -1 2 1.30627 0.34174 5

-2 -1 2 1.55685 0.29689 2

-2 -1 2 1.32997 0.24322 1

1 1 -2 3434.27 96.6918 2

1 1 -2 2764.49 96.8672 5

0 1 -2 74.7772 2.49481 4

0 1 -2 74.8435 2.40003 1

0 1 -2 76.1696 2.42136 6

0 -1 2 76.2452 2.41987 6

0 -1 2 72.5618 2.39445 1

0 -1 2 76.5285 2.50282 4

-1 1 -2 76.6262 2.69910 4

-1 1 -2 79.9278 2.56853 6

1 -1 2 79.0811 2.52421 3

1 -1 2 78.7100 2.57308 6

1 -1 2 80.7763 2.71820 4

1 -1 2 77.9290 2.57669 1

-2 1 -2 1410.92 43.0658 1

-2 1 -2 1354.18 43.2916 4

-2 1 -2 1314.21 42.6986 5

2 -1 2 1384.97 42.9577 3

2 -1 2 1360.23 43.0770 4

2 -1 2 1358.90 43.1763 1

-3 1 -2 345.355 11.1355 5

-3 1 -2 362.845 11.2787 1

-3 1 -2 353.959 11.4356 4

3 -1 2 356.713 11.2054 3

3 -1 2 355.541 11.4188 4

3 -1 2 361.164 11.2638 1

3 -1 2 343.077 11.1553 5

-4 1 -2 79.8442 3.18410 4

-4 1 -2 79.4523 2.89791 1

-4 1 -2 77.6761 2.73074 5

4 -1 2 80.4168 3.17021 4

4 -1 2 78.8844 2.88959 1

4 -1 2 76.8387 2.77733 5

4 -1 2 76.3261 2.81099 3

-5 1 -2 85.8135 2.96857 5

-5 1 -2 78.8803 3.13955 1

-5 1 -2 81.2049 3.85113 4

5 -1 2 77.6624 3.51374 4

5 -1 2 85.7795 3.03411 5

5 -1 2 81.4123 3.25441 3

-6 1 -2 261.626 9.26724 4

6 -1 2 262.736 8.82270 3

6 -1 2 266.486 8.90294 1

6 -1 2 279.404 8.79885 5

-7 1 -2 29.7839 1.78521 5

7 -1 2 28.0450 2.16572 1

7 -1 2 32.4127 2.04823 3

-8 1 -2 0.90374 1.01233 5

8 -1 2 0.32804 0.77606 3

8 -1 2 0.48898 1.03036 1

-9 1 -2 26.6546 2.15140 5

9 -1 2 23.2943 2.17886 3

9 -1 2 27.0370 2.51839 1

-10 1 -2 1.95608 1.46357 5

10 -1 2 0.66648 1.24173 3

10 -1 2 1.71065 1.45482 1

11 -1 2 0.87192 1.61038 3

11 -1 2 -1.3461 1.65697 1

12 -1 2 0.43108 1.76237 1

12 -1 2 1.32809 2.24697 3

13 -1 2 0.13734 2.27115 1

13 -1 2 0.38372 2.67483 3

14 0 -2 2.17817 3.02701 1

14 0 -2 -3.8872 3.43859 3

13 0 -2 -0.4804 2.64182 3

13 0 -2 -1.0164 2.24924 1

12 0 -2 5.25591 1.93033 1

12 0 -2 8.47468 3.67108 3

11 0 -2 13.3648 1.92909 3

11 0 -2 12.0518 1.83374 1

-11 0 2 19.6669 3.12530 1

10 0 -2 23.9198 2.68133 3

10 0 -2 22.2453 2.53141 1

-10 0 2 18.5871 2.72550 1

9 0 -2 72.7217 3.55869 3

9 0 -2 74.3769 3.44692 1

-9 0 2 76.5040 3.42193 5

8 0 -2 67.9981 3.11040 1

8 0 -2 61.3322 3.01297 3

-8 0 2 70.7995 3.00340 5

7 0 -2 9.91603 1.14719 3

7 0 -2 12.1811 1.59028 1

-7 0 2 14.5547 1.33519 5

6 0 -2 1525.52 48.6004 1

6 0 -2 1515.13 48.6184 3

-6 0 2 1618.22 49.0556 5

5 0 -2 3100.52 90.7430 5

5 0 -2 2855.74 91.1936 1

5 0 -2 2745.35 90.2569 4

-5 0 2 2888.41 90.5354 1

-5 0 2 2889.96 90.9610 5

4 0 -2 448.825 13.6199 5

4 0 -2 443.958 13.7749 4

4 0 -2 415.770 13.6136 1

-4 0 2 407.296 13.5964 5

-4 0 2 431.128 13.5919 1

3 0 -2 17.6631 1.01694 3

3 0 -2 16.6260 0.88831 5

3 0 -2 16.6228 0.91917 1

-3 0 2 19.0462 0.94719 3

-3 0 2 19.0949 0.91854 1

-3 0 2 16.8781 0.94298 5

2 0 -2 4490.66 145.088 3

2 0 -2 4830.98 144.667 2

-2 0 2 4690.92 144.982 3

-2 0 2 4572.28 144.987 5

1 0 -2 1411.45 43.2356 1

1 0 -2 1405.76 43.1187 2

1 0 -2 1336.34 43.3569 3

-1 0 2 1398.06 43.3285 3

-1 0 2 1410.99 43.5382 6

-1 0 2 1342.38 43.1012 2

-1 0 2 1374.38 43.2167 1

0 0 -2 981.043 34.5378 4

0 0 -2 956.171 34.4450 6

0 0 -2 1152.95 34.4362 1

0 0 -2 993.787 34.1871 3

0 0 2 1219.38 34.5891 3

0 0 2 1033.72 34.6010 1

0 0 2 1223.27 34.7199 6

0 0 2 1181.55 34.6334 4

-1 0 -2 29.4551 1.02573 5

-1 0 -2 30.4576 1.17471 4

-1 0 -2 30.2005 1.07458 6

1 0 2 28.8334 1.05423 1

-2 0 -2 109.901 3.75052 4

-2 0 -2 109.447 3.60087 1

-2 0 -2 107.813 3.54313 5

2 0 2 103.014 3.57191 1

2 0 2 110.385 3.56443 5

2 0 2 111.731 3.64485 6

2 0 2 115.231 3.78259 4

-3 0 -2 1037.63 32.5011 5

-3 0 -2 1052.59 32.7511 4

-3 0 -2 991.527 32.1682 1

3 0 2 991.549 32.3160 4

3 0 2 1058.82 32.4445 5

3 0 2 1050.03 32.4779 1

-4 0 -2 4519.02 143.423 5

-4 0 -2 4535.28 143.424 1

-4 0 -2 4595.78 144.249 4

4 0 2 4736.16 144.018 4

4 0 2 4593.23 143.480 5

4 0 2 4570.20 143.835 1

-5 0 -2 128.160 4.68285 1

-5 0 -2 138.775 4.58850 5

5 0 2 128.749 4.75989 1

5 0 2 140.054 5.01459 4

5 0 2 142.471 4.68486 5

-6 0 -2 15.4093 2.15868 4

6 0 2 12.0920 1.37894 5

6 0 2 12.6744 1.52132 1

6 0 2 10.4354 1.36857 3

-7 0 -2 55.1480 2.53295 5

7 0 2 53.8062 2.59854 3

7 0 2 56.6415 2.73787 1

-8 0 -2 263.633 8.60038 5

8 0 2 241.205 8.69873 3

8 0 2 267.964 8.85496 1

-9 0 -2 -0.0821 1.19348 5

9 0 2 -1.0373 0.85225 3

9 0 2 -0.0720 1.13850 1

-10 0 -2 2.28081 1.45383 5

10 0 2 1.36075 1.41891 1

10 0 2 0.65870 1.03632 3

-11 0 -2 -0.7552 1.69472 5

11 0 2 1.50870 1.73863 1

11 0 2 0.99084 1.61957 3

-12 0 -2 8.08774 2.41079 5

12 0 2 5.30918 2.37312 3

12 0 2 9.87934 2.38698 1

13 0 2 -1.5120 2.17941 1

13 0 2 -4.6458 2.72639 3

14 -1 -2 -1.1569 3.11337 3

14 -1 -2 0.26525 2.97471 1

13 -1 -2 -0.6975 2.25855 1

13 -1 -2 -2.0426 2.53801 3

12 -1 -2 0.79624 2.02774 1

12 -1 -2 0.66866 2.36953 3

-12 1 2 3.97186 2.18773 1

11 -1 -2 3.87198 1.66385 1

11 -1 -2 5.49029 1.68726 3

-11 1 2 3.61952 1.83754 1

10 -1 -2 2.39233 1.28383 3

10 -1 -2 2.60274 1.26395 1

-10 1 2 3.60564 1.60745 1

9 -1 -2 4.11242 1.31310 1

9 -1 -2 3.53232 1.03655 3

8 -1 -2 29.4115 2.22638 1

8 -1 -2 32.4307 2.26847 3

7 -1 -2 1.77067 0.94245 1

7 -1 -2 1.18951 0.82485 3

6 -1 -2 1.61135 0.72323 3

6 -1 -2 1.72918 0.84758 5

6 -1 -2 2.34086 0.80912 1

5 -1 -2 368.105 12.7491 1

5 -1 -2 384.822 12.7376 4

5 -1 -2 423.840 12.5235 5

-5 1 2 379.233 12.4885 5

-5 1 2 389.040 12.5820 3

-5 1 2 404.904 12.5693 1

4 -1 -2 1023.25 31.2654 3

4 -1 -2 934.354 30.7846 1

4 -1 -2 970.143 30.7102 5

4 -1 -2 998.484 30.8982 4

-4 1 2 986.104 30.7716 1

-4 1 2 981.895 30.7782 3

3 -1 -2 881.865 26.5568 5

3 -1 -2 887.941 26.6421 1

3 -1 -2 792.966 26.4234 3

-3 1 2 834.662 26.4194 3

-3 1 2 824.565 26.3949 1

2 -1 -2 1.09644 0.30944 1

-2 1 2 1.49032 0.27755 3

-2 1 2 1.84558 0.37387 1

-2 1 2 1.24692 0.27728 1

1 -1 -2 3602.44 109.342 1

1 -1 -2 3539.29 109.488 6

1 -1 -2 3367.16 109.414 3

-1 1 2 3508.21 109.373 1

0 -1 -2 71.8431 2.44429 3

0 -1 -2 75.8776 2.45859 4

0 -1 -2 73.6382 2.47275 6

0 -1 -2 75.5369 2.46051 1

0 1 2 78.0269 2.47636 6

0 1 2 76.5205 2.46897 1

0 1 2 76.4524 2.45202 3

-1 -1 -2 119.406 3.91679 6

-1 -1 -2 119.124 3.88364 5

-1 -1 -2 120.199 3.92591 4

-1 -1 -2 120.832 3.90252 1

1 1 2 124.596 3.92852 6

1 1 2 120.372 3.90414 1

1 1 2 123.256 3.93548 4

-2 -1 -2 1544.84 49.1932 5

-2 -1 -2 1548.42 49.3284 4

-2 -1 -2 1573.48 49.1164 1

2 1 2 1611.54 49.4879 4

2 1 2 1448.83 49.1034 1

2 1 2 1635.49 49.6567 6

2 1 2 1588.63 49.2351 5

-3 -1 -2 296.310 9.55597 5

-3 -1 -2 299.816 9.71028 4

-3 -1 -2 300.523 9.57494 1

3 1 2 316.342 9.75959 4

3 1 2 294.259 9.55233 1

3 1 2 299.978 9.59204 5

-4 -1 -2 65.3817 2.42374 1

-4 -1 -2 62.5581 2.37539 5

-4 -1 -2 65.5417 2.69440 4

4 1 2 64.2594 2.37356 1

4 1 2 66.3828 2.71449 4

4 1 2 64.7679 2.47071 5

-5 -1 -2 101.288 4.11470 4

-5 -1 -2 101.442 3.70089 5

-5 -1 -2 106.520 3.74994 1

5 1 2 108.621 3.80875 1

5 1 2 104.969 3.82865 5

5 1 2 104.312 4.11278 4

-6 -1 -2 308.525 9.80017 5

6 1 2 292.683 9.84552 3

6 1 2 302.193 9.83301 1

-7 -1 -2 25.8045 1.87460 5

7 1 2 24.3606 1.98128 1

7 1 2 22.6319 1.81005 3

-8 -1 -2 -0.3926 1.10671 5

8 1 2 -0.0366 1.06861 1

8 1 2 0.26734 0.84867 3

-9 -1 -2 23.6888 2.23821 5

9 1 2 18.9302 2.41755 1

9 1 2 17.6220 2.12828 3

-10 -1 -2 4.87068 1.55617 5

10 1 2 1.18301 1.17726 3

10 1 2 2.00787 1.41443 1

-11 -1 -2 0.50898 1.81624 5

11 1 2 0.31181 1.29912 3

11 1 2 2.23930 1.79956 1

-12 -1 -2 0.66485 2.30324 5

12 1 2 -1.1811 2.00812 3

12 1 2 1.36314 2.37686 1

13 1 2 1.02383 2.56362 3

13 1 2 0.48783 2.17359 1

14 -2 -2 0.12242 3.21251 1

14 -2 -2 -6.5519 2.96484 3

13 -2 -2 5.51497 2.43029 1

13 -2 -2 2.74825 2.90992 3

-12 2 2 10.5756 2.27005 1

11 -2 -2 2.51631 1.62839 1

11 -2 -2 -0.3667 1.65031 3

-11 2 2 1.24987 1.90462 1

10 -2 -2 0.77931 1.38798 3

10 -2 -2 1.67431 1.33107 1

-10 2 2 -0.6408 1.62213 1

9 -2 -2 119.416 4.82428 3

9 -2 -2 110.668 4.70847 1

8 -2 -2 135.320 5.03183 3

8 -2 -2 127.634 5.04040 1

7 -2 -2 18.8765 1.88332 3

7 -2 -2 18.9882 1.94882 1

6 -2 -2 84.1116 3.33622 3

6 -2 -2 87.2881 3.11329 5

6 -2 -2 78.4755 3.36457 1

5 -2 -2 644.739 19.7856 5

5 -2 -2 581.271 19.9745 1

5 -2 -2 665.743 20.0911 3

5 -2 -2 612.656 20.0446 4

-5 2 2 632.001 19.9121 1

-5 2 2 629.303 19.8930 3

4 -2 -2 374.321 12.4837 1

4 -2 -2 395.071 12.5610 4

4 -2 -2 384.901 12.3216 5

4 -2 -2 381.654 12.4534 3

-4 2 2 399.223 12.4323 3

-4 2 2 401.655 12.4427 1

3 -2 -2 3706.32 112.073 5

3 -2 -2 3579.50 112.282 4

3 -2 -2 3394.38 112.600 3

3 -2 -2 3632.69 112.811 1

-3 2 2 3631.50 112.252 1

-3 2 2 3560.64 112.386 3

-3 2 2 3594.08 112.456 1

2 -2 -2 887.477 27.6465 1

2 -2 -2 864.811 27.6062 6

-2 2 2 850.690 27.2925 3

-2 2 2 850.258 27.6250 1

-2 2 2 899.931 27.5580 1

1 -2 -2 155.615 5.08726 6

1 -2 -2 160.297 5.08329 1

-1 2 2 156.606 5.09989 1

-1 2 2 163.114 5.11192 3

-1 2 2 157.534 5.12430 6

0 -2 -2 3.85660 0.48065 6

0 -2 -2 2.96728 0.47058 1

0 -2 -2 3.22681 0.25944 2

0 -2 -2 2.79771 0.42847 3

0 2 2 2.73202 0.33040 3

0 2 2 2.68139 0.25514 2

0 2 2 2.72779 0.37720 1

-2 -2 -2 38.0837 1.53687 4

-2 -2 -2 37.4927 1.54082 1

-2 -2 -2 37.5772 1.49961 5

2 2 2 39.5336 1.63176 4

2 2 2 39.5765 1.52601 5

2 2 2 38.9778 1.53139 6

2 2 2 35.1501 1.53322 1

-3 -2 -2 736.456 22.8200 1

-3 -2 -2 740.573 22.9057 4

-3 -2 -2 707.984 22.8171 5

3 2 2 732.062 22.8601 5

3 2 2 757.887 22.9512 4

3 2 2 726.106 22.9382 6

3 2 2 697.648 22.7858 1

-4 -2 -2 20.4944 1.26347 5

-4 -2 -2 21.4059 1.27089 1

-4 -2 -2 23.4245 1.52060 4

4 2 2 20.1056 1.17038 1

4 2 2 21.3586 1.54946 4

4 2 2 19.0426 1.30692 5

-5 -2 -2 358.818 11.8058 4

-5 -2 -2 372.621 11.6237 5

-5 -2 -2 358.767 11.5754 1

5 2 2 342.949 11.6405 5

5 2 2 381.448 11.8847 4

5 2 2 348.770 11.6222 1

-6 -2 -2 38.4755 2.07742 5

6 2 2 41.0470 1.95141 1

-7 -2 -2 179.613 6.01796 5

7 2 2 170.957 6.06453 3

7 2 2 167.150 5.97833 1

-8 -2 -2 11.3631 1.44011 5

8 2 2 8.51675 1.15043 3

8 2 2 9.94170 1.34704 1

-9 -2 -2 9.01795 1.53608 5

9 2 2 5.44385 1.08041 3

9 2 2 6.97258 1.45267 1

-10 -2 -2 2.68323 1.54094 5

10 2 2 0.09603 1.07510 3

10 2 2 1.88004 1.37707 1

-11 -2 -2 10.6791 2.11646 5

11 2 2 5.62000 1.42455 3

11 2 2 8.76214 1.93155 1

-12 -2 -2 -1.9361 2.25883 5

12 2 2 0.55400 2.30778 1

12 2 2 -0.1219 1.43107 3

-13 -2 -2 0.74722 2.93513 5

13 2 2 -1.2525 2.71097 3

13 2 2 -0.6604 2.30584 1

13 -3 -2 -2.0389 2.37255 3

13 -3 -2 1.42167 2.50285 1

12 -3 -2 0.14787 3.75153 3

12 -3 -2 3.79566 2.08920 1

-12 3 2 3.47638 2.26846 1

11 -3 -2 0.35134 1.62755 1

11 -3 -2 2.26341 1.68836 3

-11 3 2 3.22998 2.07217 1

10 -3 -2 2.03175 1.45049 1

10 -3 -2 1.49744 1.27150 3

-10 3 2 -0.7870 1.71983 1

9 -3 -2 1.49902 1.26632 1

9 -3 -2 0.15885 1.21024 3

-9 3 2 0.18970 1.46325 1

8 -3 -2 0.98518 1.11196 1

8 -3 -2 1.32946 1.08310 3

7 -3 -2 14.4824 1.98329 3

7 -3 -2 18.8798 1.89064 1

6 -3 -2 325.979 10.0025 5

6 -3 -2 278.547 10.2290 1

6 -3 -2 320.135 10.2047 3

5 -3 -2 14.3863 1.05972 5

5 -3 -2 10.5030 1.73553 4

-5 3 2 12.2959 1.09047 3

4 -3 -2 132.866 4.69463 3

4 -3 -2 131.264 4.73919 1

4 -3 -2 141.822 4.49540 5

4 -3 -2 135.709 4.80263 4

-4 3 2 139.173 4.62432 3

-4 3 2 134.687 4.62321 1

3 -3 -2 198.093 6.59527 1

3 -3 -2 200.261 6.61687 4

3 -3 -2 194.005 6.58487 3

-3 3 2 208.389 6.51828 1

-3 3 2 205.437 6.59752 1

-3 3 2 196.911 6.50824 3

2 -3 -2 721.203 23.4031 6

2 -3 -2 811.011 24.0525 1

2 -3 -2 704.991 23.4570 3

2 -3 -2 731.979 23.4423 4

-2 3 2 769.662 23.7843 1

-2 3 2 771.944 23.4144 1

-2 3 2 725.795 23.4095 3

1 -3 -2 897.077 27.7253 1

1 -3 -2 890.637 27.8085 3

1 -3 -2 877.460 27.6998 6

-1 3 2 845.934 27.8090 1

-1 3 2 896.822 27.5219 6

-1 3 2 855.252 27.4107 3

0 -3 -2 52.4133 2.00772 6

0 -3 -2 55.0957 2.04155 3

0 -3 -2 55.4990 1.89276 2

0 -3 -2 59.0138 2.01580 1

0 3 2 59.4216 2.09151 1

0 3 2 56.3014 1.95560 2

0 3 2 59.2934 2.05486 6

-1 -3 -2 1666.89 51.4978 6

-1 -3 -2 1650.87 51.3804 5

-1 -3 -2 1642.02 51.1037 1

-1 -3 -2 1595.71 51.1808 2

1 3 2 1620.69 51.0759 2

1 3 2 1671.82 51.4945 6

1 3 2 1590.83 51.2630 1

-2 -3 -2 256.924 8.36310 5

-2 -3 -2 268.334 8.35353 1

2 3 2 264.580 8.31502 2

2 3 2 250.074 8.35632 1

2 3 2 261.050 8.40050 6

2 3 2 269.403 8.39371 5

-3 -3 -2 101.033 3.51594 5

-3 -3 -2 100.950 3.51547 4

-3 -3 -2 102.448 3.49106 1

3 3 2 101.987 3.58422 6

3 3 2 98.5091 3.45629 1

3 3 2 105.368 3.47230 2

3 3 2 102.280 3.59561 4

3 3 2 104.457 3.55638 5

-4 -3 -2 1248.54 37.1050 1

-4 -3 -2 1165.56 37.1247 5

4 3 2 1200.23 37.2117 1

4 3 2 1262.42 37.1327 2

4 3 2 1092.16 37.2428 6

4 3 2 1123.44 37.1603 5

4 3 2 1214.39 37.2615 4

-5 -3 -2 728.979 23.8857 5

-5 -3 -2 762.330 23.8350 1

5 3 2 778.691 24.0917 4

-6 -3 -2 7.39663 1.16535 5

6 3 2 5.76844 0.89793 1

-7 -3 -2 16.9184 1.78739 5

7 3 2 19.1695 1.63309 1

-8 -3 -2 39.0826 2.57294 5

8 3 2 38.6423 2.43573 1

-9 -3 -2 0.38609 1.39539 5

9 3 2 0.29163 0.93589 3

9 3 2 -0.4992 1.16120 1

-10 -3 -2 24.4907 2.81697 5

10 3 2 18.2154 2.46541 3

10 3 2 28.8934 2.98707 1

-11 -3 -2 0.47546 1.99292 5

11 3 2 1.92533 1.74981 1

11 3 2 -1.8521 1.24690 3

12 3 2 -0.2020 1.50812 3

12 3 2 3.58581 2.30780 1

-13 -3 -2 0.05781 2.96505 5

13 3 2 -1.6565 2.41310 1

13 3 2 -0.4871 1.56989 3

13 -4 -2 1.30796 2.58694 3

13 -4 -2 1.63300 2.57116 1

12 -4 -2 1.31734 2.25749 3

12 -4 -2 5.48823 2.22051 1

-12 4 2 0.88128 2.46570 1

11 -4 -2 1.57407 1.78795 3

11 -4 -2 2.68160 1.76375 1

-11 4 2 7.21084 2.22860 1

10 -4 -2 1.13069 1.43815 3

10 -4 -2 2.22909 1.59300 1

-10 4 2 4.03930 2.06504 1

9 -4 -2 21.3579 2.56214 1

9 -4 -2 20.7187 2.68691 3

-9 4 2 23.9774 3.23445 1

8 -4 -2 1.42235 1.09206 3

8 -4 -2 -0.0129 1.21109 1

-8 4 2 -0.6759 1.37161 1

7 -4 -2 31.2563 2.43739 1

7 -4 -2 38.4272 2.46996 3

6 -4 -2 0.81112 0.77007 5

6 -4 -2 -2.0909 1.01301 1

6 -4 -2 0.98429 0.94464 3

5 -4 -2 51.8145 2.00617 5

5 -4 -2 50.2042 2.44957 3

5 -4 -2 49.9671 2.47903 1

-5 4 2 48.1802 2.72078 1

4 -4 -2 394.282 13.0960 3

4 -4 -2 412.506 13.1759 4

4 -4 -2 416.124 12.8758 5

4 -4 -2 390.897 13.1504 1

-4 4 2 406.021 13.1366 1

-4 4 2 415.270 13.0139 3

-4 4 2 415.417 13.0389 1

3 -4 -2 90.5876 3.32232 6

3 -4 -2 98.0582 3.47447 1

3 -4 -2 93.9507 3.42560 4

3 -4 -2 90.5587 3.43335 3

-3 4 2 95.3352 3.48442 1

-3 4 2 95.4425 3.30441 1

-3 4 2 97.6799 3.31747 3

2 -4 -2 582.732 18.3581 6

2 -4 -2 578.652 18.4548 3

2 -4 -2 598.130 18.4014 4

-2 4 2 578.320 18.4232 1

-2 4 2 582.491 18.3870 3

1 -4 -2 131.859 4.40437 6

1 -4 -2 130.497 4.30107 2

1 -4 -2 136.694 4.39627 1

1 -4 -2 127.235 4.34631 4

1 -4 -2 128.633 4.50028 3

-1 4 2 136.291 4.42551 1

-1 4 2 129.328 4.46285 1

-1 4 2 139.762 4.48835 3

-1 4 2 139.327 4.34617 6

0 -4 -2 481.719 15.5048 2

0 -4 -2 483.583 15.5548 6

0 -4 -2 502.979 15.5747 1

0 4 2 505.011 15.5069 2

0 4 2 490.520 15.6323 3

0 4 2 494.511 15.6205 1

0 4 2 510.737 15.5763 6

-1 -4 -2 1118.83 35.6218 1

-1 -4 -2 1138.99 35.8979 2

-1 -4 -2 1170.16 36.2466 5

-1 -4 -2 1137.62 35.9272 6

1 4 2 1099.53 35.5805 2

1 4 2 1182.17 36.0306 6

1 4 2 1111.03 35.6653 1

1 4 2 1184.37 35.9304 3

-2 -4 -2 1403.61 43.5487 5

-2 -4 -2 1324.67 42.9553 1

-2 -4 -2 1344.34 43.2267 2

2 4 2 1351.44 43.1703 1

2 4 2 1450.27 43.2182 5

2 4 2 1456.93 43.5523 6

2 4 2 1323.55 42.9481 2

-3 -4 -2 1036.96 32.4509 1

-3 -4 -2 1023.42 32.5061 5

3 4 2 1033.45 32.5446 5

3 4 2 1032.62 32.5755 6

3 4 2 1033.80 32.4612 1

3 4 2 1069.81 32.4664 2

-4 -4 -2 15.4541 1.30328 1

-4 -4 -2 14.4696 1.26468 5

4 4 2 15.5580 1.43157 5

4 4 2 14.0819 1.14427 1

4 4 2 14.5626 1.28262 6

4 4 2 16.0871 1.51179 4

4 4 2 17.8483 1.27587 2

-5 -4 -2 44.0556 2.22336 1

-5 -4 -2 47.4887 2.27354 5

5 4 2 48.9589 2.54521 4

-6 -4 -2 68.3123 3.04818 5

6 4 2 69.1496 3.38543 4

6 4 2 74.8048 2.83422 1

-7 -4 -2 10.4269 1.46610 5

7 4 2 12.1794 1.58978 1

-8 -4 -2 22.1846 2.26474 5

8 4 2 19.0256 1.96619 1

-9 -4 -2 4.21282 1.58324 5

9 4 2 2.86980 0.98562 3

9 4 2 4.44812 1.38654 1

-10 -4 -2 4.10143 1.92632 5

10 4 2 2.21200 1.18753 3

10 4 2 3.97501 1.60067 1

-11 -4 -2 8.40573 2.24657 5

11 4 2 7.88064 2.01110 1

11 4 2 5.25912 1.52228 3

-12 -4 -2 6.02117 2.50435 5

12 4 2 2.57473 1.53693 3

12 4 2 6.90308 2.38619 1

-13 -4 -2 -0.3381 3.09530 5

13 4 2 -0.1235 1.67381 3

13 4 2 -1.0179 2.46315 1

13 -5 -2 2.44223 2.71354 1

13 -5 -2 -2.1782 2.83098 3

-13 5 2 6.74823 3.29764 1

12 -5 -2 1.54446 2.26215 3

12 -5 -2 -0.8702 2.14673 1

-12 5 2 2.92246 2.58061 1

11 -5 -2 0.74416 2.10213 3

11 -5 -2 3.00692 1.90210 1

-11 5 2 3.59797 2.39317 1

10 -5 -2 0.07010 1.56497 1

10 -5 -2 0.11764 1.42473 3

-10 5 2 1.75507 2.04328 1

9 -5 -2 25.2103 2.98907 3

9 -5 -2 23.6943 2.78761 1

-9 5 2 19.5629 2.46709 1

8 -5 -2 45.3803 3.03528 1

8 -5 -2 49.6011 3.13081 3

-8 5 2 44.2003 3.31216 1

7 -5 -2 54.4702 3.12708 1

7 -5 -2 58.9842 3.15002 3

-7 5 2 53.2572 3.36744 1

6 -5 -2 2.16400 1.12672 1

6 -5 -2 2.51676 0.95397 3

6 -5 -2 3.95254 0.85886 5

-6 5 2 1.64316 1.26536 1

5 -5 -2 50.0801 2.67424 3

5 -5 -2 37.1448 2.55201 1

5 -5 -2 53.0327 1.95856 5

-5 5 2 41.2514 2.76772 1

4 -5 -2 118.198 4.39576 4

4 -5 -2 118.754 4.23316 6

4 -5 -2 124.807 4.57418 3

-4 5 2 114.252 4.17857 1

-4 5 2 116.694 4.13439 3

3 -5 -2 390.268 12.7873 1

3 -5 -2 381.138 12.6039 6

3 -5 -2 394.905 12.7346 4

3 -5 -2 397.806 12.7646 3

-3 5 2 397.925 12.7437 1

-3 5 2 409.901 12.6281 3

2 -5 -2 395.862 12.7757 3

2 -5 -2 404.786 12.6882 4

2 -5 -2 395.729 12.6362 6

-2 5 2 385.640 12.7360 1

-2 5 2 400.197 12.6989 3

-2 5 2 408.441 12.6685 1

1 -5 -2 762.542 24.4302 3

1 -5 -2 755.913 24.2655 2

1 -5 -2 767.200 24.3140 6

-1 5 2 782.828 24.4126 3

-1 5 2 824.955 24.3859 1

-1 5 2 796.081 24.2742 6

-1 5 2 749.095 24.3983 1

0 -5 -2 116.110 3.88239 2

0 -5 -2 116.408 3.90825 6

0 -5 -2 117.301 4.03357 3

0 -5 -2 118.001 3.93530 1

0 5 2 121.254 3.95428 6

0 5 2 121.454 3.88437 2

0 5 2 119.222 4.04714 3

0 5 2 114.687 4.04663 1

-1 -5 -2 158.049 5.06950 1

-1 -5 -2 154.463 5.06795 6

-1 -5 -2 152.011 5.04653 2

-1 -5 -2 150.020 5.12231 5

1 5 2 154.161 5.03490 2

1 5 2 155.292 5.18706 1

1 5 2 156.084 5.10152 6

1 5 2 157.748 5.16730 3

-2 -5 -2 65.2858 2.40432 1

-2 -5 -2 62.7714 2.45431 5

-2 -5 -2 68.2597 2.40832 2

2 5 2 66.4975 2.37606 2

2 5 2 68.6138 2.43856 6

2 5 2 60.1177 2.50919 1

-3 -5 -2 75.9381 2.88218 5

-3 -5 -2 70.9397 2.73085 1

3 5 2 75.0692 2.73421 2

3 5 2 74.3338 2.89745 5

3 5 2 71.6642 2.84581 1

3 5 2 77.1993 2.82205 6

-4 -5 -2 83.9489 3.05417 1

-4 -5 -2 84.1128 3.13454 5

4 5 2 76.7920 3.11555 1

4 5 2 82.3839 3.19202 6

-5 -5 -2 75.8292 3.14892 5

5 5 2 71.2405 3.32944 4

5 5 2 77.9541 2.91719 1

-6 -5 -2 145.997 5.34682 5

6 5 2 145.047 5.73178 4

6 5 2 157.592 5.17057 1

-7 -5 -2 141.749 5.40725 5

7 5 2 149.986 5.20732 1

-8 -5 -2 27.0893 2.44985 5

8 5 2 24.9272 2.16433 1

-9 -5 -2 0.41368 1.50997 5

9 5 2 -0.3734 1.31098 1

-10 -5 -2 9.79748 2.08680 5

10 5 2 4.28813 1.62639 1

10 5 2 4.48874 1.36750 3

-11 -5 -2 8.20392 2.39313 5

11 5 2 2.38733 1.53769 3

11 5 2 2.17218 1.97797 1

-12 -5 -2 1.40371 2.61265 5

12 5 2 -0.1162 2.24178 1

12 5 2 0.19525 1.47868 3

-13 -5 -2 1.87546 3.21243 5

13 5 2 0.66214 1.81724 3

13 5 2 -0.0182 2.48739 1

13 -6 -2 2.80868 3.16791 6

13 -6 -2 -0.1407 2.84566 1

-13 6 2 1.56165 3.38504 1

12 -6 -2 0.07254 2.48897 3

12 -6 -2 0.12713 2.37536 1

-12 6 2 3.00638 2.89119 1

11 -6 -2 0.34093 2.09105 3

11 -6 -2 1.77106 2.07554 1

-11 6 2 -0.0386 2.46687 1

10 -6 -2 5.40222 1.90377 3

10 -6 -2 4.27636 1.93872 1

-10 6 2 8.48386 2.32719 1

9 -6 -2 -0.2674 1.57749 3

9 -6 -2 0.37834 1.47518 1

-9 6 2 -0.1664 1.93817 1

8 -6 -2 30.8986 3.04845 3

8 -6 -2 29.7017 2.82364 1

-8 6 2 26.6899 3.18500 1

7 -6 -2 19.2556 2.52987 3

7 -6 -2 18.3068 2.38254 1

-7 6 2 14.9326 1.94851 1

6 -6 -2 95.1213 3.45574 5

6 -6 -2 88.8952 4.19064 1

6 -6 -2 103.010 4.26499 3

6 -6 -2 96.7662 3.94248 6

-6 6 2 92.7717 4.33303 1

5 -6 -2 2.67719 1.20112 6

5 -6 -2 3.42701 1.13952 3

5 -6 -2 2.73354 0.72190 5

5 -6 -2 -0.6051 1.10172 1

-5 6 2 -0.9422 1.29645 1

4 -6 -2 41.4522 2.21985 6

4 -6 -2 43.7192 2.53603 4

-4 6 2 51.0163 2.55911 1

3 -6 -2 184.747 6.48233 3

3 -6 -2 183.097 6.33443 4

3 -6 -2 183.694 6.20138 6

-3 6 2 191.935 6.24529 3

-3 6 2 189.171 6.23332 1

2 -6 -2 1.15626 0.98287 4

2 -6 -2 2.28721 0.75565 3

2 -6 -2 3.77532 0.81716 6

-2 6 2 3.29102 0.83200 1

-2 6 2 3.37998 0.64612 3

1 -6 -2 2.90422 0.68121 6

1 -6 -2 1.42582 0.65960 3

1 -6 -2 2.66424 0.54443 2

-1 6 2 2.95711 0.56639 6

-1 6 2 1.83967 0.72365 1

-1 6 2 1.91903 0.59897 3

-1 6 2 2.39237 0.57335 1

0 -6 -2 107.839 3.65327 6

0 -6 -2 110.138 3.84517 3

0 -6 -2 105.429 3.75275 5

0 -6 -2 108.965 3.72119 1

0 -6 -2 108.289 3.68030 2

0 6 2 113.859 3.71958 6

0 6 2 104.379 3.88939 1

0 6 2 107.203 3.66465 2

0 6 2 114.400 3.87752 3

-1 -6 -2 676.176 21.9732 2

-1 -6 -2 695.491 21.9769 6

-1 -6 -2 704.607 21.9764 1

-1 -6 -2 678.863 22.0284 5

1 6 2 710.682 22.1045 3

1 6 2 724.902 22.0160 6

1 6 2 707.649 22.0817 1

1 6 2 706.498 21.9720 2

-2 -6 -2 12.0905 1.21449 5

-2 -6 -2 13.2828 1.10531 2

-2 -6 -2 15.8719 1.09832 1

2 6 2 16.0257 1.46122 1

2 6 2 14.4339 1.07182 2

2 6 2 12.6776 1.10588 6

-3 -6 -2 163.618 5.69551 2

-3 -6 -2 168.537 5.54208 1

3 6 2 175.965 5.86064 5

3 6 2 163.320 5.58410 2

3 6 2 167.274 5.65437 1

3 6 2 165.167 5.65254 6

-4 -6 -2 87.9260 3.35111 5

-4 -6 -2 85.0915 3.17167 1

4 6 2 80.9817 3.27588 6

-5 -6 -2 263.594 8.83813 5

5 6 2 266.586 8.65511 1

6 6 2 123.743 4.36964 1

6 6 2 117.105 4.91397 4

-7 -6 -2 32.8467 2.39353 5

7 6 2 31.9644 2.02719 1

-8 -6 -2 43.3905 3.05816 5

8 6 2 46.3159 2.69066 1

-9 -6 -2 20.6265 2.78474 5

9 6 2 18.1580 2.44361 1

-10 -6 -2 1.88950 2.09117 5

10 6 2 0.09311 1.67319 1

10 6 2 0.22188 1.28120 3

11 6 2 -0.2179 1.93945 3

11 6 2 -0.3397 2.00601 1

-12 -6 -2 3.63594 2.87797 5

12 6 2 5.73079 2.37967 1

12 6 2 0.36814 1.79814 3

-13 -6 -2 -0.6843 3.39065 5

13 6 2 0.00012 2.02047 3

13 6 2 -3.2428 2.51452 1

13 -7 -2 -1.1296 3.11445 6

13 -7 -2 -4.4355 3.13510 1

13 -7 -2 -7.7278 3.46923 3

-13 7 2 0.84281 3.60413 1

12 -7 -2 0.46347 2.88737 3

12 -7 -2 -0.9002 2.71396 1

12 -7 -2 1.82601 3.47823 6

-12 7 2 4.35075 3.23138 1

11 -7 -2 8.93259 2.57531 6

11 -7 -2 7.53848 3.21250 1

11 -7 -2 14.6365 2.71119 3

-11 7 2 8.15712 2.77878 1

10 -7 -2 8.83371 2.38644 3

10 -7 -2 8.86240 2.30826 6

10 -7 -2 6.51020 2.19831 1

-10 7 2 10.1983 2.66800 1

9 -7 -2 3.02753 1.86687 6

9 -7 -2 3.79077 1.82015 1

9 -7 -2 2.49580 1.83752 3

-9 7 2 2.69612 2.02178 1

8 -7 -2 18.4032 2.67032 6

8 -7 -2 21.9235 3.37448 3

8 -7 -2 14.9612 2.14835 1

-8 7 2 16.3842 2.28452 1

7 -7 -2 170.099 7.19516 1

7 -7 -2 193.800 6.94923 6

7 -7 -2 207.255 7.19112 3

-7 7 2 188.617 7.29055 1

6 -7 -2 80.4317 3.82741 3

6 -7 -2 73.6268 3.42062 6

6 -7 -2 75.5512 2.88408 5

6 -7 -2 70.3242 3.76426 1

-6 7 2 72.9880 3.84724 1

5 -7 -2 55.7678 3.18432 1

5 -7 -2 65.8658 3.25973 3

5 -7 -2 55.5442 2.84711 6

5 -7 -2 62.6890 2.33667 5

-5 7 2 53.7692 3.16934 1

4 -7 -2 8.75817 1.34439 3

4 -7 -2 8.51512 1.25482 6

4 -7 -2 4.13912 1.67912 4

-4 7 2 5.14403 1.31642 1

3 -7 -2 215.798 7.43252 6

3 -7 -2 214.065 7.62091 4

-3 7 2 223.801 7.71178 1

-3 7 2 246.549 7.93412 3

2 -7 -2 127.637 4.33915 2

2 -7 -2 120.772 4.91068 3

2 -7 -2 125.253 4.37086 6

-2 7 2 123.775 4.64975 1

-2 7 2 127.589 4.48916 3

-2 7 2 133.305 4.42542 1

1 -7 -2 755.032 24.1028 3

1 -7 -2 740.328 23.8732 6

1 -7 -2 748.357 23.9142 2

-1 7 2 794.255 23.8470 6

-1 7 2 777.402 24.0799 3

-1 7 2 793.621 24.0063 1

-1 7 2 725.846 24.3634 1

0 -7 -2 113.311 3.86290 6

0 -7 -2 110.323 3.95019 2

0 -7 -2 112.572 4.00398 5

0 7 2 120.517 4.18072 3

0 7 2 119.361 3.95223 6

-1 -7 -2 43.7301 1.83784 2

-1 -7 -2 41.9678 1.78413 1

-1 -7 -2 35.8481 1.92554 5

1 7 2 37.0566 2.76867 1

1 7 2 44.4333 1.80550 2

1 7 2 42.5097 1.91785 6

1 7 2 43.6766 2.01920 3

-2 -7 -2 3.75100 0.85795 2

-2 -7 -2 3.96744 0.67636 1

-2 -7 -2 2.28967 0.91574 5

2 7 2 2.60983 0.64456 3

2 7 2 3.13888 0.72007 2

-3 -7 -2 322.340 10.3284 5

-3 -7 -2 317.971 10.2639 2

-3 -7 -2 314.243 10.1435 1

3 7 2 311.640 10.2822 6

-4 -7 -2 18.9337 1.41394 1

-4 -7 -2 12.3035 1.70485 5

4 7 2 17.7067 1.67451 1

-5 -7 -2 344.592 11.2594 5

5 7 2 339.553 11.0856 1

-6 -7 -2 9.97420 1.40642 5

6 7 2 8.30925 1.14408 1

-7 -7 -2 35.0239 2.63418 5

7 7 2 39.0747 2.33153 1

-8 -7 -2 1.49159 1.67363 5

8 7 2 -0.1255 1.20661 1

-9 -7 -2 48.9600 3.54197 5

9 7 2 49.8959 3.14530 1

-10 -7 -2 2.17501 2.13506 5

10 7 2 1.99610 1.78090 1

10 7 2 2.37733 1.41364 3

-11 -7 -2 1.93965 2.61236 5

11 7 2 4.88530 2.10414 1

11 7 2 1.30489 1.67097 3

-12 -7 -2 0.15502 2.99728 5

12 7 2 0.42110 1.92772 3

12 7 2 -1.6859 2.35117 1

12 -8 -2 -2.3724 2.99122 1

12 -8 -2 -2.8982 3.21357 3

12 -8 -2 -4.5100 2.96264 6

-12 8 2 3.07779 4.84013 1

11 -8 -2 8.38348 5.37183 3

11 -8 -2 5.14656 2.70898 1

11 -8 -2 5.18126 2.67032 6

-11 8 2 7.21999 3.12419 1

10 -8 -2 2.93571 2.30020 6

10 -8 -2 2.45195 2.23766 1

10 -8 -2 3.49421 2.30875 3

-10 8 2 4.48690 2.58253 1

9 -8 -2 13.2965 2.41768 1

9 -8 -2 16.1119 2.32487 3

9 -8 -2 14.9255 2.46441 6

-9 8 2 16.1064 2.65977 1

8 -8 -2 0.24753 1.73922 6

8 -8 -2 1.08787 1.52952 3

8 -8 -2 0.96747 1.69676 1

-8 8 2 0.78468 1.93251 1

7 -8 -2 92.4676 4.24875 6

7 -8 -2 80.8594 4.64133 1

7 -8 -2 100.624 4.70422 3

-7 8 2 89.9799 4.77035 1

6 -8 -2 5.58471 0.89969 5

6 -8 -2 2.42208 1.46378 1

6 -8 -2 5.35571 1.50339 6

6 -8 -2 3.43990 1.35446 3

-6 8 2 2.62133 1.52557 1

5 -8 -2 25.2639 2.74400 3

5 -8 -2 19.9446 2.12517 6

-5 8 2 23.6801 2.63346 1

4 -8 -2 112.609 4.58967 3

4 -8 -2 111.880 4.23503 6

-4 8 2 109.913 4.65528 1

3 -8 -2 1.63190 1.04744 6

3 -8 -2 1.45412 1.34021 4

3 -8 -2 0.91860 1.14526 3

-3 8 2 1.02758 1.23362 1

2 -8 -2 381.768 12.0740 2

2 -8 -2 376.810 12.0248 6

2 -8 -2 351.139 12.4961 3

-2 8 2 380.765 12.3528 1

-2 8 2 391.326 12.1534 1

1 -8 -2 0.43200 0.77390 2

1 -8 -2 0.04610 0.65946 6

-1 8 2 1.69432 0.63543 6

-1 8 2 1.24195 0.84127 3

-1 8 2 1.52344 0.98385 1

-1 8 2 1.58525 0.72889 1

0 -8 -2 750.443 24.2145 2

0 -8 -2 736.825 24.2308 5

0 -8 -2 739.472 24.1150 6

0 8 2 818.721 24.3023 1

0 8 2 755.755 24.3617 1

0 8 2 796.457 24.4645 3

0 8 2 793.318 24.1601 6

-1 -8 -2 2.86811 0.85175 2

-1 -8 -2 3.58410 0.92461 5

1 8 2 4.11605 1.01234 6

1 8 2 3.04039 1.06058 1

1 8 2 2.82002 0.78458 2

-2 -8 -2 201.428 6.57947 1

-2 -8 -2 192.513 6.53810 5

-2 -8 -2 186.135 6.46828 2

2 8 2 192.672 6.64334 1

2 8 2 192.791 6.46623 6

-3 -8 -2 56.1146 2.52459 2

-3 -8 -2 56.5639 2.69923 5

3 8 2 56.4460 2.53033 6

3 8 2 61.8053 2.77020 1

-4 -8 -2 31.0765 2.08061 5

4 8 2 28.7385 2.13454 1

-5 -8 -2 179.448 6.36457 5

5 8 2 178.101 6.16860 1

-6 -8 -2 -0.0748 1.10856 5

6 8 2 -1.5794 0.91690 1

-7 -8 -2 68.9462 3.69491 5

7 8 2 75.8471 3.29691 1

-8 -8 -2 18.0339 2.69003 5

8 8 2 16.5711 2.25170 1

-9 -8 -2 41.6582 3.57185 5

9 8 2 34.6469 3.02714 1

-10 -8 -2 4.10053 2.40821 5

10 8 2 -1.4810 1.77295 1

-11 -8 -2 3.66730 2.86859 5

11 8 2 5.09646 1.85906 3

11 8 2 6.40732 2.22143 1

-12 -8 -2 -0.0041 3.31818 5

12 8 2 -1.0491 2.07321 3

12 8 2 1.19947 2.41292 1

12 -9 -2 -5.2446 3.29647 3

12 -9 -2 1.18413 3.29300 6

-12 9 2 -1.5834 3.54405 1

11 -9 -2 3.00991 2.87345 6

11 -9 -2 2.01128 2.93083 3

-11 9 2 0.86161 3.46292 1

10 -9 -2 19.1147 2.94379 6

10 -9 -2 15.5834 3.07910 3

10 -9 -2 17.4048 3.02611 1

-10 9 2 18.9024 3.23667 1

9 -9 -2 46.1607 3.79974 6

9 -9 -2 42.1611 4.70722 1

9 -9 -2 50.0641 5.00057 3

-9 9 2 50.5463 5.05399 1

8 -9 -2 1.87606 2.06418 1

8 -9 -2 4.01590 1.99249 6

8 -9 -2 3.00429 1.86253 3

-8 9 2 6.37093 2.27515 1

7 -9 -2 21.2473 3.39294 3

7 -9 -2 19.0193 2.62255 6

-7 9 2 13.3313 2.26315 1

6 -9 -2 2.34849 0.78815 5

6 -9 -2 2.82186 1.58450 3

6 -9 -2 0.97526 1.62383 6

-6 9 2 -0.2778 1.75683 1

5 -9 -2 37.5911 2.53576 6

5 -9 -2 39.6281 2.92524 3

-5 9 2 29.0033 3.08588 1

4 -9 -2 365.444 12.1969 3

4 -9 -2 350.797 11.8178 6

-4 9 2 374.055 12.3402 1

3 -9 -2 72.5237 3.23176 4

3 -9 -2 71.0343 3.47589 3

3 -9 -2 68.8102 3.17910 2

3 -9 -2 72.2028 2.95766 6

-3 9 2 66.8802 3.55466 1

2 -9 -2 859.988 27.2880 3

-2 9 2 859.347 27.1209 1

-2 9 2 856.869 27.3312 1

1 -9 -2 368.574 13.2273 2

1 -9 -2 394.822 13.0543 5

1 -9 -2 419.408 13.3144 3

-1 9 2 422.378 12.8843 6

-1 9 2 408.174 13.3249 1

-1 9 2 432.928 13.1557 1

0 -9 -2 1216.55 38.6084 6

0 -9 -2 1175.86 38.7789 5

0 9 2 1317.67 38.8286 1

0 9 2 1221.34 38.9074 1

0 9 2 1256.44 38.6408 6

-1 -9 -2 72.5993 2.95947 2

-1 -9 -2 75.7013 2.99272 5

1 9 2 74.3195 2.93961 6

1 9 2 69.5936 3.26523 1

-2 -9 -2 844.459 26.6159 5

-2 -9 -2 821.551 26.5655 2

2 9 2 863.892 26.6970 1

2 9 2 840.180 26.5547 6

-3 -9 -2 13.6632 1.46240 5

-3 -9 -2 13.1070 1.31052 2

3 9 2 12.3140 1.98938 1

3 9 2 15.1339 1.71390 6

-4 -9 -2 56.0642 2.86830 5

-4 -9 -2 58.0999 2.78219 2

4 9 2 58.7230 2.89800 1

-5 -9 -2 4.25628 1.42427 5

5 9 2 5.77731 1.13224 1

6 9 2 23.0679 2.16490 1

-7 -9 -2 44.1851 3.15017 5

7 9 2 48.6831 2.65903 1

-8 -9 -2 20.1965 2.47398 5

8 9 2 19.4501 2.42473 1

-9 -9 -2 1.99761 2.22012 5

9 9 2 1.76132 1.52286 1

-10 -9 -2 6.63488 2.72027 5

10 9 2 3.01947 1.94004 1

-11 -9 -2 1.84053 2.94591 5

11 9 2 1.55622 2.37191 1

11 9 2 -1.0585 1.84849 3

-12 -9 -2 3.34131 3.52339 5

12 9 2 3.34541 2.38128 3

12 9 2 0.35198 2.44097 1

12 -10 -2 -4.3392 3.46914 6

12 -10 -2 -4.3667 3.33274 3

-12 10 2 1.60960 3.95991 1

11 -10 -2 -3.2456 2.76430 6

11 -10 -2 -0.4132 2.90667 3

-11 10 2 1.34398 3.43414 1

10 -10 -2 0.04019 2.73883 3

10 -10 -2 -1.5911 2.49981 6

-10 10 2 3.21266 3.09164 1

9 -10 -2 6.20043 2.29785 6

9 -10 -2 6.22413 2.34752 3

-9 10 2 5.98761 2.88130 1

8 -10 -2 7.24006 2.24048 3

8 -10 -2 7.82922 2.23034 6

-8 10 2 11.5943 2.71031 1

7 -10 -2 69.2201 4.41980 3

7 -10 -2 59.5809 3.64086 6

-7 10 2 60.4327 4.54362 1

6 -10 -2 8.64261 1.87634 6

6 -10 -2 5.84468 1.71665 3

-6 10 2 3.91565 1.98209 1

5 -10 -2 8.66646 1.57383 3

5 -10 -2 10.7161 1.62217 6

-5 10 2 6.97463 1.94869 1

4 -10 -2 56.7321 3.32622 3

4 -10 -2 50.8785 2.72895 6

4 -10 -2 56.8972 2.78031 2

-4 10 2 54.4299 3.68627 1

3 -10 -2 75.1083 3.71382 3

3 -10 -2 79.0957 3.26693 2

3 -10 -2 79.8818 3.51065 4

-3 10 2 73.9896 3.92532 1

2 -10 -2 292.688 9.58207 2

2 -10 -2 292.813 9.84389 3

-2 10 2 286.531 9.93873 1

1 -10 -2 1.00329 0.97118 2

-1 10 2 4.41034 1.42629 1

-1 10 2 2.01567 0.98794 1

0 -10 -2 366.942 11.9734 5

0 -10 -2 366.387 11.9944 2

0 10 2 375.631 11.7985 6

0 10 2 377.732 12.0126 1

0 10 2 364.677 12.2347 1

-1 -10 -2 5.82918 1.17247 5

-1 -10 -2 3.79476 1.00700 2

1 10 2 7.30099 1.24073 6

1 10 2 4.82514 1.04800 1

1 10 2 4.26318 1.42256 1

-2 -10 -2 195.230 6.83921 2

-2 -10 -2 200.113 6.87844 5

2 10 2 201.127 6.83401 6

2 10 2 204.014 7.06862 1

-3 -10 -2 52.6807 2.54538 2

-3 -10 -2 49.0277 2.66748 5

3 10 2 45.6602 2.87655 1

3 10 2 50.4148 2.54531 6

-4 -10 -2 38.1009 2.39024 2

-4 -10 -2 41.3341 2.57972 5

4 10 2 44.9711 2.58463 1

-5 -10 -2 90.6474 3.97624 5

5 10 2 88.5568 3.86361 1

-6 -10 -2 17.1016 2.46186 5

6 10 2 20.3044 2.21556 1

-7 -10 -2 3.03527 1.75705 5

7 10 2 3.91497 1.33390 1

-8 -10 -2 10.8833 2.28438 5

8 10 2 9.08178 1.52240 1

-9 -10 -2 5.82451 2.55156 5

9 10 2 5.63447 1.69511 1

-10 -10 -2 3.93315 2.82820 5

10 10 2 1.00856 1.96659 1

-11 -10 -2 5.74356 3.34666 5

11 10 2 5.27062 3.38812 1

11 10 2 0.60524 1.97148 3

11 -11 -2 -4.4790 3.52575 6

11 -11 -2 -3.2720 3.19401 3

-11 11 2 2.46600 3.72243 1

10 -11 -2 -0.1991 2.76354 3

10 -11 -2 -1.6096 2.63134 6

-10 11 2 2.71662 3.35915 1

9 -11 -2 -1.7411 2.35562 3

9 -11 -2 -1.9607 2.21786 6

-9 11 2 6.97155 3.09152 1

8 -11 -2 6.06161 2.13893 6

8 -11 -2 4.39534 2.18034 3

-8 11 2 5.88841 2.85549 1

7 -11 -2 4.29184 1.88284 3

7 -11 -2 5.33314 2.04143 6

-7 11 2 6.59928 2.54214 1

6 -11 -2 7.71188 1.84347 3

6 -11 -2 6.78669 1.88084 6

-6 11 2 6.51158 2.31899 1

5 -11 -2 110.378 4.61434 6

5 -11 -2 124.997 5.35696 3

5 -11 -2 116.905 4.65047 2

-5 11 2 122.879 5.74533 1

4 -11 -2 57.7799 2.76361 6

4 -11 -2 61.3796 3.52180 3

4 -11 -2 62.3466 2.97553 2

-4 11 2 53.1394 4.03338 1

3 -11 -2 116.771 4.74484 3

3 -11 -2 113.707 4.39072 2

-3 11 2 107.289 5.08590 1

2 -11 -2 -1.9142 1.02496 2

2 -11 -2 1.39259 1.14352 5

2 -11 -2 -1.1215 0.93392 3

-2 11 2 0.43725 1.50657 1

1 -11 -2 374.181 12.2095 2

1 -11 -2 375.375 12.1248 5

-1 11 2 379.671 12.5393 1

-1 11 2 368.807 12.2241 1

0 -11 -2 1.99865 1.15322 2

0 -11 -2 2.71463 1.10449 5

0 11 2 0.58228 1.37000 1

0 11 2 2.10358 1.02129 1

-1 -11 -2 44.0749 2.43163 5

-1 -11 -2 43.4948 2.36587 2

1 11 2 36.5772 2.22809 6

1 11 2 46.7762 2.39770 1

1 11 2 46.7830 2.87869 1

-2 -11 -2 25.6263 2.06782 5

-2 -11 -2 26.4062 1.98307 2

2 11 2 22.7803 2.57647 1

2 11 2 22.0579 2.07658 6

2 11 2 26.5103 2.02955 1

-3 -11 -2 320.813 10.2349 5

-3 -11 -2 296.210 10.1627 2

3 11 2 298.797 10.2945 6

3 11 2 304.315 10.2957 1

-4 -11 -2 0.49239 1.14080 2

-4 -11 -2 -0.3157 1.35972 5

4 11 2 -0.8277 1.31465 1

-5 -11 -2 18.3450 2.54005 5

5 11 2 20.5072 2.41796 1

-6 -11 -2 2.18820 1.61991 5

6 11 2 0.91494 1.24071 1

-7 -11 -2 18.9768 2.43217 5

7 11 2 17.1515 2.49974 1

-8 -11 -2 -1.4245 2.15285 5

8 11 2 0.86044 1.35223 1

-9 -11 -2 2.33856 2.87065 5

9 11 2 1.26445 1.74694 1

-10 -11 -2 6.15495 3.33877 5

10 11 2 -0.5265 2.05955 1

-11 -11 -2 7.38212 3.85410 5

11 11 2 0.67897 2.42021 3

11 11 2 4.15975 2.34564 1

10 -12 -2 1.84975 2.99342 3

-10 12 2 8.26134 3.87375 1

9 -12 -2 -1.8643 2.71319 6

9 -12 -2 -0.0588 2.37936 3

-9 12 2 7.21211 3.31338 1

8 -12 -2 -1.1219 2.00322 6

8 -12 -2 3.49672 2.20404 3

-8 12 2 -0.8006 2.93769 1

7 -12 -2 0.19231 1.87066 3

7 -12 -2 -1.3661 1.63985 6

-7 12 2 -0.0031 2.69326 1

6 -12 -2 0.91544 1.42883 2

6 -12 -2 1.63668 1.78775 3

-6 12 2 1.87295 2.43421 1

5 -12 -2 1.93657 1.50572 3

5 -12 -2 2.63470 1.37973 2

-5 12 2 -2.6767 2.10097 1

4 -12 -2 0.31649 1.36441 3

4 -12 -2 1.03893 1.26958 2

-4 12 2 -0.7303 1.91571 1

3 -12 -2 137.733 5.23102 2

3 -12 -2 140.288 5.59074 3

3 -12 -2 139.689 4.97544 5

-3 12 2 134.048 5.92727 1

2 -12 -2 117.128 4.54129 2

2 -12 -2 115.682 4.31826 5

2 -12 -2 111.430 4.74001 3

-2 12 2 114.856 5.16572 1

1 -12 -2 16.6328 1.92841 5

1 -12 -2 15.0243 1.63109 2

-1 12 2 16.2590 2.01487 1

0 -12 -2 37.9931 2.45773 2

0 -12 -2 36.1745 2.38310 5

0 12 2 36.7530 2.45508 1

0 12 2 36.1824 3.04681 1

-1 -12 -2 203.776 6.99323 5

-1 -12 -2 201.869 7.04526 2

1 12 2 205.073 7.33080 1

1 12 2 196.834 6.99487 1

-2 -12 -2 21.0111 2.17779 5

-2 -12 -2 19.5363 2.11761 2

2 12 2 23.1639 2.78653 1

2 12 2 20.7025 2.18282 1

-3 -12 -2 53.3968 2.94642 5

-3 -12 -2 48.0413 2.84436 2

3 12 2 51.0175 3.25965 1

-4 -12 -2 2.60491 1.43737 2

-4 -12 -2 1.55411 1.57810 5

4 12 2 1.91895 1.52745 1

-5 -12 -2 14.3594 2.15195 5

5 12 2 13.7780 1.70257 1

-6 -12 -2 6.12498 1.96293 5

6 12 2 4.09678 1.48121 1

-7 -12 -2 -0.5320 1.98179 5

7 12 2 0.13824 1.38419 1

8 12 2 21.8895 2.86987 1

-9 -12 -2 9.65527 3.18080 5

9 12 2 5.61006 1.90764 1

-10 -12 -2 5.47908 3.37816 5

10 12 2 2.11173 2.11316 1

10 -13 -2 5.21746 2.93113 3

-10 13 2 2.77827 4.10087 1

9 -13 -2 1.76920 2.52693 3

-9 13 2 1.22665 3.66197 1

8 -13 -2 3.16419 2.41942 3

-8 13 2 8.76729 3.56128 1

7 -13 -2 10.5704 2.34743 3

7 -13 -2 10.4686 1.92427 2

-7 13 2 8.29316 3.16567 1

6 -13 -2 1.94292 1.92307 3

6 -13 -2 -0.0320 1.57799 2

-6 13 2 -0.3048 2.73748 1

5 -13 -2 3.77646 1.54057 2

5 -13 -2 2.14607 1.70980 3

-5 13 2 1.82389 2.39659 1

4 -13 -2 2.92246 1.48490 2

4 -13 -2 3.84345 1.62847 3

-4 13 2 1.03417 2.07757 1

3 -13 -2 42.2183 2.46029 5

3 -13 -2 38.7934 2.78740 2

3 -13 -2 43.5252 3.30416 3

-3 13 2 38.3697 3.87793 1

2 -13 -2 12.8616 1.95851 5

2 -13 -2 12.5546 1.67763 2

-2 13 2 11.8296 2.16302 1

1 -13 -2 44.1702 2.61267 5

1 -13 -2 46.5867 2.79631 2

-1 13 2 44.4193 3.57551 1

0 -13 -2 6.46262 1.53456 2

0 -13 -2 8.56740 1.46941 5

0 13 2 5.87992 1.80412 1

-1 -13 -2 2.44837 1.32298 2

-1 -13 -2 0.95057 1.33327 5

1 13 2 0.00502 1.67691 1

-2 -13 -2 5.76742 1.53210 2

-2 -13 -2 4.92542 1.55456 5

2 13 2 4.69197 1.82100 1

-3 -13 -2 3.06805 1.44161 2

-3 -13 -2 3.37257 1.60222 5

3 13 2 1.15870 1.76044 1

-4 -13 -2 11.4221 1.82280 2

-4 -13 -2 12.2692 2.03814 5

4 13 2 10.6083 1.91590 1

-5 -13 -2 5.49908 1.96391 5

5 13 2 2.09234 1.49787 1

-6 -13 -2 15.8221 2.50454 5

6 13 2 15.3352 1.84665 1

-7 -13 -2 24.7310 3.58924 5

7 13 2 14.9674 1.89458 1

-8 -13 -2 2.26243 2.91819 5

8 13 2 2.39174 1.68750 1

-9 -13 -2 1.98770 3.14775 5

-10 -13 -2 -0.6739 3.86411 5

9 -14 -2 -1.5162 2.70888 3

9 -14 -2 -2.9411 3.53453 2

-9 14 2 10.5259 4.02983 1

8 -14 -2 -0.3837 2.01565 2

8 -14 -2 0.94138 4.52987 3

-8 14 2 2.92207 3.53356 1

7 -14 -2 2.08001 1.89608 2

7 -14 -2 2.62641 2.27266 3

-7 14 2 4.80183 3.28699 1

6 -14 -2 1.80959 1.98070 3

6 -14 -2 1.94015 1.70386 2

5 -14 -2 34.4789 4.02127 3

5 -14 -2 30.7474 3.06044 2

-5 14 2 34.5890 5.07684 1

4 -14 -2 8.87935 1.82457 2

4 -14 -2 3.99378 1.76300 3

-4 14 2 5.62957 2.60763 1

3 -14 -2 -1.4796 1.46864 2

3 -14 -2 0.70600 1.30251 5

-3 14 2 1.14699 2.18362 1

2 -14 -2 5.51493 1.67588 2

2 -14 -2 6.19175 1.41107 5

-2 14 2 4.89903 2.23435 1

1 -14 -2 3.62215 1.39855 5

1 -14 -2 -0.1684 1.40790 2

-1 14 2 -2.3016 1.92280 1

0 -14 -2 -0.5920 1.41792 2

0 -14 -2 0.87463 1.44798 5

0 14 2 0.03120 1.84711 1

-1 -14 -2 0.24485 1.49539 5

-1 -14 -2 1.12824 1.42132 2

1 14 2 3.77894 1.86631 1

-2 -14 -2 16.7065 1.89150 2

-2 -14 -2 16.7591 1.97152 5

2 14 2 12.1019 2.22615 1

-3 -14 -2 43.5924 3.22673 5

-3 -14 -2 43.5463 3.08105 2

3 14 2 47.2902 3.62086 1

-4 -14 -2 9.29685 2.03857 5

-4 -14 -2 8.17364 1.82502 2

4 14 2 6.84318 1.94123 1

-5 -14 -2 0.33761 1.96304 5

5 14 2 1.60830 1.69123 1

-6 -14 -2 -0.2607 2.28095 5

6 14 2 -2.7875 1.56250 1

-7 -14 -2 3.45959 2.60451 5

-8 -14 -2 1.96001 2.95419 5

-9 -14 -2 9.13009 3.62513 5

8 -15 -2 0.42190 2.21151 2

8 -15 -2 5.24086 2.87497 3

-8 15 2 2.76037 3.91141 1

7 -15 -2 1.07493 2.04068 2

-7 15 2 -1.2593 3.52088 1

6 -15 -2 1.73387 1.92938 2

6 -15 -2 0.67959 2.10444 3

-6 15 2 -2.8235 3.24321 1

5 -15 -2 6.12361 2.00289 2

5 -15 -2 4.71349 1.96962 3

-5 15 2 6.68606 3.24072 1

4 -15 -2 7.52107 1.84171 3

4 -15 -2 4.32708 1.89758 2

-4 15 2 6.51093 2.94565 1

3 -15 -2 17.1594 2.17630 2

-3 15 2 11.5741 3.02423 1

2 -15 -2 2.16870 1.39828 5

2 -15 -2 0.06721 1.65590 2

-2 15 2 2.58566 2.42565 1

1 -15 -2 12.2861 1.90219 2

1 -15 -2 13.1905 1.77153 5

-1 15 2 12.0366 2.43692 1

0 -15 -2 0.06649 1.55683 2

0 -15 -2 -0.5181 1.49645 5

0 15 2 -0.2017 2.09064 1

-1 -15 -2 12.2566 1.89260 2

-1 -15 -2 13.2524 1.87956 5

1 15 2 9.33586 2.28795 1

-2 -15 -2 10.2487 1.90798 5

-2 -15 -2 8.93151 1.76855 2

2 15 2 7.49174 2.30296 1

-3 -15 -2 8.03845 1.91113 2

-3 -15 -2 12.6467 2.06858 5

3 15 2 8.82235 2.23816 1

-4 -15 -2 3.23032 2.00193 5

-4 -15 -2 1.59956 1.87100 2

4 15 2 2.22574 1.94433 1

-5 -15 -2 3.30788 2.35757 5

5 15 2 2.53975 1.81571 1

-6 -15 -2 2.21853 2.50627 5

6 15 2 0.08031 1.78858 1

-7 -15 -2 -4.7938 2.75536 5

-8 -15 -2 5.20674 3.53282 5

7 -16 -2 3.87416 2.61030 3

7 -16 -2 3.14068 2.55663 2

-7 16 2 2.95143 3.98789 1

6 -16 -2 -2.9151 2.19292 2

-6 16 2 1.63531 3.79963 1

5 -16 -2 -0.3753 1.93232 3

5 -16 -2 0.96035 2.16810 2

-5 16 2 -1.2754 3.30759 1

4 -16 -2 5.05531 2.15660 2

-4 16 2 -0.3702 3.13436 1

3 -16 -2 17.5609 2.51401 2

-3 16 2 18.0577 3.79248 1

2 -16 -2 0.92798 1.39974 5

2 -16 -2 -0.1807 1.87044 2

-2 16 2 1.18325 2.68905 1

1 -16 -2 2.06464 1.87389 2

1 -16 -2 0.81952 1.54020 5

-1 16 2 -0.9503 2.50291 1

0 -16 -2 1.04228 1.84960 2

0 -16 -2 1.68614 1.60672 5

0 16 2 1.26333 2.35971 1

-1 -16 -2 17.2323 2.31503 2

-1 -16 -2 18.9042 2.76234 5

1 16 2 18.6170 2.86181 1

-2 -16 -2 0.67813 1.86377 5

-2 -16 -2 3.61663 1.93692 2

2 16 2 -1.8906 2.27830 1

-3 -16 -2 1.15360 1.98637 5

-3 -16 -2 1.25282 1.78268 2

3 16 2 -1.1034 2.26406 1

-4 -16 -2 -0.7956 2.17104 5

4 16 2 0.56388 2.06729 1

-5 -16 -2 16.7697 2.89234 5

5 16 2 10.7940 2.29362 1

-6 -16 -2 2.42765 2.87414 5

-7 -16 -2 1.33371 3.10762 5

6 -17 -2 -0.3361 3.19673 2

6 -17 -2 4.92838 2.51631 3

-6 17 2 -7.0568 3.89312 1

5 -17 -2 2.87621 2.49307 2

-5 17 2 9.78830 3.99398 1

4 -17 -2 7.11272 2.62573 2

-4 17 2 2.81841 3.58045 1

3 -17 -2 5.93930 2.33040 2

-3 17 2 4.81705 3.33036 1

2 -17 -2 1.02265 2.07271 2

-2 17 2 -0.6765 3.00006 1

1 -17 -2 0.73539 1.61788 5

1 -17 -2 3.31570 2.12056 2

-1 17 2 2.49642 2.88785 1

0 -17 -2 -0.0318 1.63766 5

0 -17 -2 -0.5341 1.95106 2

0 17 2 2.40750 2.98832 1

-1 -17 -2 4.47798 2.10567 2

-1 -17 -2 2.34399 1.89287 5

-2 -17 -2 8.99943 2.15853 5

-2 -17 -2 7.60112 2.24538 2

-3 -17 -2 8.29511 2.41967 5

-3 -17 -2 4.47641 2.59913 2

3 17 2 5.09511 4.42224 1

-4 -17 -2 10.8025 2.71949 5

-5 -17 -2 2.88781 2.77926 5

-6 -17 -2 4.34763 3.23557 5

4 -18 -2 2.96754 2.63950 2

-4 18 2 2.19582 4.00487 1

-3 18 2 -0.3936 3.65287 1

2 -18 -2 10.7525 3.34496 2

-2 18 2 10.7938 3.61565 1

1 -18 -2 -1.5810 3.57160 2

-1 18 2 3.53225 3.21365 1

0 -18 -2 9.60680 4.69024 2

0 18 2 7.02874 3.18902 1

-1 -18 -2 2.95049 2.32450 2

-1 -18 -2 2.33470 2.14014 5

1 18 2 -0.2552 2.96206 1

-2 -18 -2 3.27322 2.30381 5

-2 -18 -2 -0.1221 2.45921 2

2 18 2 -1.3826 2.86596 1

-3 -18 -2 1.22408 2.41833 5

-3 -18 -2 4.52902 2.53551 2

3 18 2 -2.1283 2.78675 1

-4 -18 -2 16.7110 2.98914 5

4 18 2 7.27429 2.77696 1

0 -19 -2 1.63998 2.81426 2

0 19 2 -1.8625 3.25591 1

-4 -18 3 -1.6898 2.61518 2

-3 -18 3 0.79937 2.50841 2

-2 -18 3 5.89161 2.81956 2

1 18 -3 3.07475 2.70428 1

-1 -18 3 2.42451 2.53371 2

0 18 -3 0.94245 2.74056 1

0 -18 3 4.54241 2.81211 2

-1 18 -3 0.00192 2.88808 1

1 -18 3 1.79361 2.71279 2

-2 18 -3 3.15180 3.14268 1

2 -18 3 0.40924 2.67466 2

-3 18 -3 2.28229 3.33356 1

3 -18 3 -1.5619 2.68574 2

-4 18 -3 0.77189 3.62790 1

4 -18 3 4.14782 2.90233 2

-6 -17 3 -2.5673 2.66085 5

-5 -17 3 1.16304 2.68023 2

-5 -17 3 1.13787 2.79059 5

-4 -17 3 1.34835 2.34560 2

-4 -17 3 6.69849 2.05531 5

-3 -17 3 -0.6625 2.21647 2

-3 -17 3 1.09565 1.78787 5

2 17 -3 6.65959 2.32042 1

-2 -17 3 6.13091 1.74620 5

-2 -17 3 8.58460 2.66566 2

1 17 -3 3.06271 2.38485 1

-1 -17 3 0.50929 2.37571 2

0 17 -3 7.32075 2.70969 1

0 -17 3 5.40571 2.89591 2

-1 17 -3 1.98248 2.83392 1

1 -17 3 1.41854 3.01395 2

-2 17 -3 0.48060 2.75964 1

2 -17 3 2.18428 2.48816 2

-3 17 -3 4.00156 3.08867 1

3 -17 3 5.56943 2.50056 2

-4 17 -3 15.0829 3.48566 1

4 -17 3 14.4989 2.99646 2

-5 17 -3 0.34943 3.32294 1

5 -17 3 0.79769 2.88757 2

-7 -16 3 -1.7070 2.72642 5

-6 -16 3 8.25204 2.68027 2

-6 -16 3 1.91735 2.46135 5

-5 -16 3 -1.4713 2.22738 2

-5 -16 3 2.35080 2.13558 5

-4 -16 3 19.9433 2.66263 2

-4 -16 3 25.3049 3.29448 5

3 16 -3 21.6977 3.24224 1

-3 -16 3 29.7316 3.00476 5

-3 -16 3 33.1440 4.55668 2

2 16 -3 4.19180 2.04027 1

-2 -16 3 2.99092 1.51408 5

-2 -16 3 1.97724 1.96314 2

1 16 -3 -1.1127 2.08798 1

-1 -16 3 -1.8153 1.77108 2

0 16 -3 30.2603 3.92791 1

0 -16 3 22.4597 2.69330 2

-1 16 -3 -1.2522 2.39959 1

1 -16 3 0.82460 2.13574 2

-2 16 -3 11.3973 2.74203 1

2 -16 3 16.0298 2.50344 2

-3 16 -3 4.87665 2.84760 1

3 -16 3 5.91728 2.31537 2

-4 16 -3 2.90272 2.88375 1

4 -16 3 1.15067 2.42158 2

-5 16 -3 13.2472 3.36478 1

5 -16 3 10.1361 2.65106 2

-6 16 -3 11.3689 3.41411 1

6 -16 3 14.3362 2.72414 2

-7 16 -3 4.99730 3.52476 1

7 -16 3 1.54256 2.49106 2

-8 -15 3 0.47305 3.02616 5

-7 -15 3 -2.2941 2.48338 5

-6 -15 3 12.0223 2.51520 5

-6 -15 3 9.32774 2.54445 2

-5 -15 3 7.62937 2.19595 5

-4 -15 3 -1.5352 1.73658 2

-4 -15 3 1.72548 1.84746 5

3 15 -3 11.9836 1.88300 1

-3 -15 3 15.0375 2.28841 2

-3 -15 3 18.5949 2.07581 5

2 15 -3 44.7219 3.40066 1

-2 -15 3 46.3711 2.85951 5

-2 -15 3 44.9560 3.63211 2

1 15 -3 11.5589 2.14845 1

-1 -15 3 11.5062 1.51379 5

-1 -15 3 11.3691 2.09316 2

0 15 -3 -0.2450 1.93189 1

0 -15 3 0.67151 1.81058 2

-1 15 -3 28.2032 3.87383 1

1 -15 3 25.7999 3.21143 2

-2 15 -3 1.90391 2.16322 1

2 -15 3 1.13693 2.02221 2

-3 15 -3 -0.9372 2.33512 1

3 -15 3 -1.4664 1.96811 2

-4 15 -3 -0.1597 2.75477 1

4 -15 3 1.39967 1.95849 2

-5 15 -3 15.7736 3.10596 1

5 -15 3 21.8493 3.06838 3

5 -15 3 16.5790 2.50523 2

-6 15 -3 7.50237 3.07548 1

6 -15 3 9.66592 1.85410 3

-7 15 -3 0.77036 3.07078 1

7 -15 3 -1.9887 2.30306 2

7 -15 3 5.81519 1.88185 3

-8 15 -3 5.01810 3.51744 1

8 -15 3 1.74251 2.52331 2

-9 -14 3 6.11863 3.11347 5

-8 -14 3 15.2786 3.00862 5

-7 -14 3 4.83377 2.40032 5

-6 -14 3 6.61209 2.15780 5

-6 -14 3 5.43035 2.03331 2

-5 -14 3 5.88206 1.99035 5

-5 -14 3 6.31861 1.97470 2

-4 -14 3 3.72452 1.73487 5

-4 -14 3 -1.2205 1.67729 2

3 14 -3 14.7297 1.82981 1

-3 -14 3 13.7712 2.00238 2

-3 -14 3 17.5516 2.39045 5

2 14 -3 -0.5826 1.58384 1

-2 -14 3 -1.5363 1.60671 2

-2 -14 3 -2.5993 1.26533 5

1 14 -3 133.633 5.60538 1

-1 -14 3 140.042 5.72595 2

-1 -14 3 137.717 5.02420 5

0 14 -3 0.52643 1.78530 1

0 -14 3 -0.1205 1.68848 2

-1 14 -3 4.68274 1.93917 1

1 -14 3 7.19134 1.80372 2

-2 14 -3 11.8693 2.24821 1

2 -14 3 14.3787 2.07922 2

-3 14 -3 77.9465 4.79041 1

3 -14 3 73.1320 4.10609 2

-4 14 -3 0.87513 2.14179 1

4 -14 3 4.43581 1.32938 3

4 -14 3 1.13699 1.89173 2

-5 14 -3 9.11302 2.58753 1

5 -14 3 9.27616 1.58340 3

5 -14 3 7.23833 2.08516 2

-6 14 -3 0.72476 2.55100 1

6 -14 3 -0.3105 1.95411 2

6 -14 3 1.54109 1.59399 3

-7 14 -3 12.7642 3.13799 1

7 -14 3 10.1026 2.98745 2

7 -14 3 14.9384 2.24290 3

-8 14 -3 6.31428 3.15442 1

8 -14 3 3.42714 2.21052 3

-9 14 -3 0.03367 3.40688 1

9 -14 3 5.14185 2.81242 3

10 13 -3 10.9235 2.79188 3

-10 -13 3 14.1195 3.49865 5

-9 -13 3 -0.7866 2.73945 5

-8 -13 3 3.84479 3.19385 5

-7 -13 3 1.58779 2.14820 5

-6 -13 3 16.7318 2.26248 2

-6 -13 3 22.1059 2.52691 5

-5 -13 3 12.9635 2.03358 5

-5 -13 3 9.49328 1.85770 2

-4 -13 3 7.55880 1.74479 2

-4 -13 3 12.3293 1.84271 5

3 13 -3 110.667 4.53480 1

-3 -13 3 113.561 4.90031 2

-3 -13 3 119.281 4.60158 5

2 13 -3 14.8186 2.34938 1

-2 -13 3 16.8979 2.01568 5

-2 -13 3 14.5563 1.98862 2

1 13 -3 18.6757 2.67117 1

-1 -13 3 25.2082 1.95475 5

-1 -13 3 23.2156 2.59903 2

0 13 -3 2.27871 1.53604 1

0 -13 3 3.74570 1.58691 2

-1 13 -3 113.623 4.96613 1

1 -13 3 109.944 4.82164 2

-2 13 -3 26.6003 3.13175 1

2 -13 3 20.7462 2.74560 2

-3 13 -3 50.7628 3.86780 4

-3 13 -3 51.5319 3.78776 1

3 -13 3 46.1030 3.26809 2

-4 13 -3 5.22883 1.99378 1

4 -13 3 8.68325 1.41005 3

4 -13 3 8.68561 1.81821 2

-5 13 -3 33.6322 3.86206 1

5 -13 3 27.9456 3.02580 3

5 -13 3 26.0388 3.13458 2

-6 13 -3 3.64904 2.33184 1

6 -13 3 3.55091 1.56147 3

6 -13 3 -0.5101 1.78189 2

-7 13 -3 8.40506 3.08754 1

7 -13 3 3.12329 1.86442 3

-8 13 -3 4.63874 2.90190 1

8 -13 3 1.37940 2.08048 3

-9 13 -3 2.94204 3.14303 1

9 -13 3 4.75948 2.36342 3

10 12 -3 2.45304 2.13939 3

-10 -12 3 6.31639 3.18089 5

-9 -12 3 -2.1348 2.57295 5

-8 -12 3 18.5061 2.80519 5

-7 -12 3 26.4546 3.28003 5

-6 -12 3 22.6898 2.96139 5

-6 -12 3 22.9922 2.91200 2

-5 -12 3 34.6776 2.91359 5

-5 -12 3 31.9881 2.85797 2

-4 -12 3 4.26588 1.40974 5

-4 -12 3 2.52657 1.48208 2

3 12 -3 7.73846 1.04060 1

3 12 -3 8.27356 1.40624 1

-3 -12 3 10.0039 1.56240 5

-3 -12 3 10.2860 1.68789 2

2 12 -3 11.6354 1.52842 1

2 12 -3 10.2030 1.59236 1

-2 -12 3 14.6906 1.91027 5

-2 -12 3 14.9517 1.64221 2

1 12 -3 32.8701 2.06352 1

1 12 -3 35.2857 2.60432 1

-1 -12 3 32.1042 2.66683 2

-1 -12 3 36.9042 2.13121 5

0 12 -3 93.3155 3.78873 1

0 12 -3 94.6471 4.18584 1

0 -12 3 96.6865 4.13689 2

-1 12 -3 9.74806 1.41140 1

-1 12 -3 9.81387 1.69677 1

1 -12 3 12.8824 1.68601 2

-2 12 -3 36.8067 3.07376 1

2 -12 3 37.3536 2.80493 2

-3 12 -3 -0.5002 2.26198 4

-3 12 -3 0.61038 1.52851 1

3 -12 3 2.01175 1.07672 3

3 -12 3 2.34817 1.46694 2

-4 12 -3 123.526 5.59014 4

-4 12 -3 121.759 5.44228 1

4 -12 3 119.649 4.89643 3

4 -12 3 116.286 5.04773 2

-5 12 -3 43.4085 3.86709 1

-5 12 -3 40.3422 4.05447 4

5 -12 3 45.3593 3.24692 3

-6 12 -3 0.97971 2.14739 1

6 -12 3 1.74136 1.52038 3

6 -12 3 0.63256 2.10101 1

-7 12 -3 0.06796 2.27905 1

7 -12 3 1.19886 1.71280 3

7 -12 3 -0.1804 2.31240 1

-8 12 -3 4.71213 3.25330 1

8 -12 3 -1.3905 2.58357 1

8 -12 3 -1.8512 1.98043 3

-9 12 -3 1.19731 2.93699 1

9 -12 3 -1.9835 2.14910 3

-10 12 -3 2.28367 3.33273 1

10 -12 3 4.06198 3.53506 1

10 -12 3 -1.3564 2.61562 3

11 11 -3 1.18622 2.27499 3

-11 -11 3 -2.7734 3.03268 5

10 11 -3 2.52567 2.15554 3

-10 -11 3 4.93343 2.81828 5

-9 -11 3 1.84760 3.60070 5

-8 -11 3 46.6242 3.97639 5

-7 -11 3 39.1205 3.30971 5

-6 -11 3 0.65532 1.40310 2

-6 -11 3 -1.1788 1.53975 5

-5 -11 3 2.05093 1.38604 2

-5 -11 3 2.29738 1.54439 5

4 11 -3 2.47746 1.00215 6

-4 -11 3 1.29251 1.28665 5

-4 -11 3 -0.4154 1.34487 2

3 11 -3 20.0353 2.00154 1

-3 -11 3 20.4727 1.99044 5

-3 -11 3 21.3415 2.23176 2

2 11 -3 25.9592 1.72332 1

2 11 -3 26.8508 2.18681 1

-2 -11 3 30.2577 1.97983 5

-2 -11 3 26.8384 2.37157 2

1 11 -3 6.72747 1.38045 1

1 11 -3 4.98523 0.98274 1

-1 -11 3 6.76821 1.50717 2

0 11 -3 227.913 7.85626 1

0 11 -3 245.500 8.09022 1

0 -11 3 234.397 8.14109 2

-1 11 -3 83.3963 3.56695 1

-1 11 -3 92.7514 3.89831 1

1 -11 3 91.2410 3.85010 2

-2 11 -3 98.5081 4.24160 1

-2 11 -3 118.476 4.60232 1

2 -11 3 114.044 4.47781 2

-3 11 -3 -0.4233 2.11860 4

-3 11 -3 -1.9041 1.42866 1

3 -11 3 1.05025 1.30531 2

3 -11 3 1.29938 0.99904 3

-4 11 -3 75.7342 4.16538 4

-4 11 -3 75.5737 3.97499 1

4 -11 3 73.2793 3.55839 3

4 -11 3 64.5938 4.01333 1

-5 11 -3 6.97002 1.87909 1

-5 11 -3 2.59160 2.86130 4

5 -11 3 2.73793 1.75577 1

5 -11 3 2.84596 1.31998 3

-6 11 -3 8.68710 2.04140 1

6 -11 3 8.22388 1.63444 3

6 -11 3 7.04217 1.99784 1

-7 11 -3 3.21123 2.14381 1

7 -11 3 1.23845 1.69188 3

7 -11 3 1.64779 2.06958 1

-8 11 -3 3.48918 2.48090 1

8 -11 3 1.35899 2.24943 1

8 -11 3 0.95310 1.95404 3

8 -11 3 4.21735 1.67726 6

-9 11 -3 11.8024 3.05115 1

9 -11 3 11.0922 2.64689 3

9 -11 3 9.86704 2.84248 1

9 -11 3 7.59403 2.47413 6

-10 11 -3 3.60968 3.06885 1

10 -11 3 0.97337 2.87743 1

10 -11 3 -0.4128 2.82135 3

-11 11 -3 4.94847 3.53731 1

11 -11 3 -2.0482 3.28022 1

11 -11 3 -6.1024 3.09259 3

12 10 -3 0.90374 2.41508 3

-12 -10 3 7.93595 3.97011 5

11 10 -3 -0.2655 1.91664 3

-11 -10 3 0.12027 3.12433 5

10 10 -3 2.39196 2.23991 3

-10 -10 3 1.59714 2.52839 5

9 10 -3 24.2111 3.27670 3

-9 -10 3 26.6429 3.64531 5

-8 -10 3 24.3123 3.33085 5

-7 -10 3 20.0894 2.76973 5

-6 -10 3 15.0663 1.93849 5

-6 -10 3 17.6928 2.25014 2

-5 -10 3 61.4319 3.16465 2

-5 -10 3 63.6724 3.13473 5

4 10 -3 3.42214 0.95629 6

-4 -10 3 1.83176 1.25138 5

-4 -10 3 3.77797 1.18546 2

3 10 -3 35.2287 1.72001 6

-3 -10 3 35.1873 2.33217 2

-3 -10 3 36.6173 2.16635 5

2 10 -3 32.7717 1.71104 1

2 10 -3 33.1415 2.13198 1

-2 -10 3 35.4163 2.03649 5

-2 -10 3 35.1824 2.39178 2

1 10 -3 120.451 4.34783 1

1 10 -3 125.358 4.56925 1

1 10 -3 125.055 4.58934 3

-1 -10 3 130.520 4.43926 5

-1 -10 3 125.731 4.72555 2

0 10 -3 36.9957 1.96718 1

0 10 -3 37.2444 2.34619 1

0 10 -3 36.2393 2.25188 3

0 -10 3 38.7234 1.83162 5

0 -10 3 37.5862 2.43668 2

-1 10 -3 270.639 9.01588 1

-1 10 -3 257.426 8.79088 3

-1 10 -3 264.522 8.84040 1

1 -10 3 276.748 9.05417 2

-2 10 -3 268.308 9.14126 3

-2 10 -3 293.578 9.56551 1

-2 10 -3 266.895 9.31302 1

-2 10 -3 298.761 9.96349 4

2 -10 3 298.537 9.49386 3

2 -10 3 286.491 9.46302 2

2 -10 3 249.271 9.55395 4

-3 10 -3 36.3601 2.92704 4

3 -10 3 30.3451 2.58912 1

3 -10 3 28.4202 2.52296 3

3 -10 3 29.0476 2.40599 2

-4 10 -3 -0.6282 2.21641 4

4 -10 3 1.17187 1.39698 1

4 -10 3 1.88826 1.08160 3

-5 10 -3 23.0027 3.35782 4

5 -10 3 24.2513 2.85493 1

5 -10 3 20.2721 2.65960 3

-6 10 -3 -1.5159 2.81794 4

6 -10 3 1.03958 1.27024 6

6 -10 3 5.55133 1.69310 1

6 -10 3 1.79549 1.38916 3

7 -10 3 13.1442 2.10035 1

7 -10 3 8.61576 1.87468 3

7 -10 3 11.4293 1.66552 6

8 -10 3 -0.1704 1.85217 3

8 -10 3 0.36668 1.83245 1

8 -10 3 1.39988 1.51537 6

-9 10 -3 9.85156 2.64426 1

9 -10 3 4.18929 1.77129 6

9 -10 3 3.01609 2.23223 3

9 -10 3 3.99233 2.32469 1

-10 10 -3 9.28042 3.02864 1

10 -10 3 7.23024 2.13156 6

10 -10 3 6.19467 2.80709 1

-11 10 -3 6.17962 3.42716 1

11 -10 3 -0.3353 3.01014 1

11 -10 3 5.51561 3.08287 6

11 -10 3 -2.8760 3.13490 3

12 9 -3 -0.4471 2.30314 3

-12 -9 3 0.54114 3.50998 5

11 9 -3 1.68290 2.04522 3

-11 -9 3 3.25808 2.86901 5

10 9 -3 3.39321 1.68887 3

-10 -9 3 6.44128 3.58875 5

9 9 -3 5.66474 1.57022 3

-9 -9 3 6.30920 2.14519 5

-8 -9 3 11.1534 2.03193 5

-7 -9 3 16.7934 2.04382 5

-6 -9 3 25.3945 2.27958 2

-6 -9 3 29.0758 2.55653 5

-5 -9 3 30.6875 2.38632 5

-5 -9 3 34.5204 2.32563 2

4 9 -3 60.7234 2.61889 6

-4 -9 3 64.7745 2.91878 2

-4 -9 3 59.3162 2.90058 5

3 9 -3 67.4717 2.59344 6

-3 -9 3 68.4709 2.93112 5

-3 -9 3 68.0285 3.02546 2

2 9 -3 63.5463 2.63397 3

2 9 -3 58.4733 2.52446 5

2 9 -3 53.3553 2.28611 1

-2 -9 3 65.1450 2.90015 2

-2 -9 3 60.5739 2.86288 5

1 9 -3 1.25201 0.91271 1

1 9 -3 3.01794 0.82551 3

-1 -9 3 4.96755 0.94155 5

-1 -9 3 2.11106 0.95219 2

0 9 -3 2.54901 0.81569 3

0 9 -3 0.47051 0.75277 1

0 9 -3 -0.4923 0.92620 1

0 -9 3 2.50358 1.05503 2

-1 9 -3 83.7323 3.59526 4

-1 9 -3 84.8677 3.49300 1

-1 9 -3 83.1106 3.22386 3

-1 9 -3 86.3546 3.26919 1

1 -9 3 86.6410 3.49374 2

-2 9 -3 4.92893 0.77178 3

-2 9 -3 7.15681 1.92442 4

2 -9 3 5.61190 1.83942 4

2 -9 3 2.66147 1.20669 1

2 -9 3 5.50559 0.92933 3

2 -9 3 6.17826 1.27384 2

-3 9 -3 56.3899 3.07293 1

-3 9 -3 48.1737 2.21023 3

-3 9 -3 47.0687 3.07140 4

3 -9 3 50.4429 3.23649 1

-4 9 -3 41.5610 3.21332 4

4 -9 3 46.0233 2.73617 3

4 -9 3 40.6848 2.83480 1

-5 9 -3 19.6900 3.02004 4

5 -9 3 27.6891 2.54831 1

5 -9 3 27.6140 1.74661 6

5 -9 3 27.5275 2.60506 3

5 -9 3 27.3568 1.57207 6

-6 9 -3 14.9550 2.99983 4

6 -9 3 11.6944 1.76754 6

6 -9 3 7.99719 1.59167 3

6 -9 3 7.10379 1.59615 1

-7 9 -3 -1.3184 2.69536 4

7 -9 3 0.20992 1.54607 3

7 -9 3 -0.5673 1.44200 1

8 -9 3 2.39630 1.75458 1

8 -9 3 0.73285 1.79623 3

9 -9 3 3.12368 1.96272 1

9 -9 3 -1.8683 1.97641 3

10 -9 3 -0.3678 2.51590 3

10 -9 3 0.89424 3.66278 1

-11 9 -3 6.33891 3.18733 1

11 -9 3 -0.2727 2.51518 1

11 -9 3 -4.6864 3.19007 3

-12 9 -3 3.46644 3.66715 1

12 -9 3 -7.0069 3.53006 3

12 -9 3 0.53074 2.81851 6

12 8 -3 -0.1171 1.96603 3

-12 -8 3 0.69861 3.07894 5

11 8 -3 -0.7087 1.84066 3

-11 -8 3 2.75775 2.63593 5

10 8 -3 5.17183 1.61743 3

-10 -8 3 13.0618 2.58326 5

9 8 -3 -0.1774 1.25461 3

-9 -8 3 -1.3630 1.88278 5

-8 -8 3 2.77578 1.75415 5

-7 -8 3 19.6499 2.52401 5

6 8 -3 7.45282 1.77935 4

-6 -8 3 4.29571 1.35366 5

-5 -8 3 127.392 4.71628 2

-5 -8 3 123.709 4.76573 5

-4 -8 3 -1.9598 1.01580 5

-4 -8 3 -0.9775 0.96297 2

3 8 -3 38.6278 1.68420 6

-3 -8 3 33.8285 2.06007 2

-3 -8 3 34.7256 1.99204 5

2 8 -3 251.359 8.27608 1

2 8 -3 257.203 8.17936 1

2 8 -3 254.736 8.35083 5

2 8 -3 264.816 8.38795 3

-2 -8 3 260.614 8.52753 2

-2 -8 3 252.021 8.38307 5

1 8 -3 15.5967 1.12201 1

1 8 -3 14.5286 1.46833 1

1 8 -3 13.6628 1.33899 3

-1 -8 3 15.6786 1.23222 5

-1 -8 3 14.7314 1.57181 2

0 8 -3 70.1235 2.67897 3

0 8 -3 63.6019 2.54093 1

0 -8 3 65.5039 2.78378 2

0 -8 3 67.4909 2.45045 5

-1 8 -3 84.6908 3.70560 4

-1 8 -3 98.9705 3.45881 1

-1 8 -3 95.5429 3.37500 3

1 -8 3 87.5918 3.83784 4

1 -8 3 96.8719 3.57742 1

1 -8 3 96.4970 3.64631 2

1 -8 3 95.6113 3.40915 3

-2 8 -3 371.793 11.8815 3

-2 8 -3 376.261 12.0704 1

-2 8 -3 385.003 12.2721 4

2 -8 3 363.936 12.2879 4

2 -8 3 372.204 12.0064 3

2 -8 3 365.634 12.1587 1

-3 8 -3 -0.9633 0.94441 1

-3 8 -3 1.59378 0.60170 3

3 -8 3 0.14376 1.05326 1

3 -8 3 0.61568 0.88157 3

-4 8 -3 2.58391 0.69029 3

-4 8 -3 5.27464 1.99101 4

4 -8 3 2.05086 1.14966 1

4 -8 3 2.87661 1.29292 3

-5 8 -3 -2.6491 2.03925 4

5 -8 3 0.39193 1.15277 1

5 -8 3 0.34764 0.84081 6

5 -8 3 1.08856 1.11161 3

-6 8 -3 -2.9751 2.17603 4

6 -8 3 2.04976 1.16055 1

6 -8 3 0.60344 1.19157 3

6 -8 3 3.30139 1.03392 6

-7 8 -3 119.666 6.08853 4

7 -8 3 125.835 5.39182 3

7 -8 3 127.610 5.29127 1

8 -8 3 40.1570 3.33893 1

8 -8 3 38.6979 3.74681 3

9 -8 3 2.33349 1.79394 1

9 -8 3 -0.8055 1.83766 3

10 -8 3 0.42325 2.00504 1

10 -8 3 -0.1201 2.16396 3

11 -8 3 8.28525 2.52686 1

11 -8 3 1.54303 3.08945 3

-12 8 -3 7.55559 3.41176 1

12 -8 3 -6.6705 3.22098 3

12 -8 3 0.74842 2.85523 1

13 7 -3 -3.1359 2.26181 3

13 7 -3 -0.7141 2.36651 1

-13 -7 3 -0.4545 3.86670 5

12 7 -3 0.50190 2.00281 3

12 7 -3 1.95094 1.91916 1

-12 -7 3 3.97536 3.01321 5

11 7 -3 4.02650 1.77981 3

11 7 -3 5.15554 1.54000 1

-11 -7 3 5.82846 2.72689 5

10 7 -3 2.05164 1.32548 3

10 7 -3 -0.4587 1.23837 1

-10 -7 3 2.23022 2.06420 5

9 7 -3 10.9334 1.47913 3

9 7 -3 12.2171 1.82017 1

-9 -7 3 18.5980 2.25386 5

8 7 -3 120.251 4.91474 3

-8 -7 3 126.091 5.08367 5

-7 -7 3 253.778 8.78960 5

6 7 -3 15.7604 2.10527 4

-6 -7 3 19.3707 2.03307 5

-5 -7 3 72.9998 3.13229 2

-5 -7 3 74.2410 3.17886 5

-4 -7 3 69.9841 2.98624 5

3 7 -3 162.489 5.54668 5

-3 -7 3 168.459 5.65762 2

-3 -7 3 161.802 5.62699 5

2 7 -3 507.460 15.7450 5

2 7 -3 511.477 15.5960 1

2 7 -3 490.159 15.6369 1

-2 -7 3 473.914 15.7700 5

-2 -7 3 505.609 15.8755 2

1 7 -3 27.6303 1.35625 5

1 7 -3 25.1464 1.41101 1

1 7 -3 27.0335 1.29379 1

1 7 -3 30.5339 1.44222 3

-1 -7 3 25.5440 1.37493 5

0 7 -3 1158.03 35.8979 1

0 7 -3 1182.72 35.8935 3

0 -7 3 1105.64 36.0360 2

-1 7 -3 565.297 17.7269 3

-1 7 -3 583.223 17.8388 1

-1 7 -3 555.860 17.9404 4

1 -7 3 558.675 17.9863 4

1 -7 3 550.623 17.7545 3

1 -7 3 555.671 18.4619 2

1 -7 3 571.805 17.8771 1

-2 7 -3 829.999 25.7465 3

-2 7 -3 834.151 26.0905 4

-2 7 -3 858.001 25.9490 1

2 -7 3 809.108 26.0816 4

2 -7 3 797.181 25.8234 3

2 -7 3 802.305 25.9641 1

-3 7 -3 2.12334 1.58697 4

-3 7 -3 0.89476 0.93114 1

-3 7 -3 1.93082 0.56445 3

3 -7 3 1.99721 0.88892 3

3 -7 3 0.18000 0.91543 1

3 -7 3 3.54401 1.68875 4

-4 7 -3 457.090 14.3601 3

-4 7 -3 452.855 14.8676 4

4 -7 3 446.272 14.2454 6

4 -7 3 441.411 14.5041 3

-5 7 -3 2.38180 2.00437 4

5 -7 3 4.01600 1.08972 3

5 -7 3 2.62922 1.15085 1

-6 7 -3 5.24853 2.33539 4

6 -7 3 6.20340 1.30897 1

6 -7 3 4.33262 1.13695 3

-7 7 -3 18.3270 3.08273 4

7 -7 3 14.7036 2.39811 1

7 -7 3 19.0449 2.60723 3

8 -7 3 3.53156 1.41765 1

8 -7 3 -0.4413 1.34770 3

9 -7 3 14.1541 2.05024 3

9 -7 3 18.5295 2.93529 1

10 -7 3 -0.4224 1.72421 1

10 -7 3 -2.0004 1.89213 3

11 -7 3 1.30688 2.14013 1

11 -7 3 -1.0031 2.45289 3

-12 7 -3 3.14809 3.06495 1

12 -7 3 -5.4484 2.75699 3

12 -7 3 1.01974 2.48184 1

13 6 -3 0.94121 2.83261 3

13 6 -3 -1.0961 2.37284 1

-13 -6 3 1.10272 3.35967 5

12 6 -3 -0.1447 1.76982 3

12 6 -3 1.27179 1.87602 1

-12 -6 3 -1.7104 2.75924 5

11 6 -3 0.51636 1.43571 3

11 6 -3 1.13507 1.55249 1

-11 -6 3 1.05901 2.28626 5

10 6 -3 10.5274 1.76856 3

10 6 -3 13.8729 1.61066 1

-10 -6 3 15.0517 2.33158 5

9 6 -3 44.3956 2.94207 3

9 6 -3 43.4597 2.53344 1

-9 -6 3 52.6371 3.14486 5

8 6 -3 1.80995 0.92479 1

8 6 -3 1.10802 1.05720 3

-8 -6 3 -0.5423 1.44114 5

-7 -6 3 8.45951 1.46028 5

6 6 -3 67.2802 3.21943 4

-6 -6 3 74.2855 3.20656 5

-5 -6 3 487.006 15.7401 2

-5 -6 3 494.352 15.8082 5

-4 -6 3 25.6969 1.69038 2

-4 -6 3 25.6519 1.75360 5

3 6 -3 19.2607 1.30640 5

-3 -6 3 17.1033 1.39850 5

2 6 -3 470.446 14.0228 5

2 6 -3 458.228 14.0878 2

-2 -6 3 433.150 14.3184 2

-2 -6 3 409.024 14.0229 5

1 6 -3 33.0092 1.28203 1

1 6 -3 31.6462 1.33240 5

1 6 -3 31.2804 1.38112 3

-1 -6 3 28.4727 1.56434 2

-1 -6 3 30.0234 1.39902 5

0 6 -3 739.368 24.2491 4

0 6 -3 781.302 24.1317 3

0 -6 3 778.354 24.1678 1

0 -6 3 803.454 24.3256 4

0 -6 3 753.192 24.2486 2

-1 6 -3 39.7645 1.55187 3

-1 6 -3 39.6896 1.70127 1

-1 6 -3 33.0953 1.98983 4

1 -6 3 38.3992 1.73687 1

1 -6 3 36.1366 2.08970 4

1 -6 3 38.0875 1.59595 3

-2 6 -3 961.936 28.2499 1

-2 6 -3 894.702 28.0254 3

-2 6 -3 889.202 28.3410 4

2 -6 3 875.375 28.0998 3

2 -6 3 862.885 28.2211 1

2 -6 3 896.581 28.3741 4

-3 6 -3 32.3062 1.45098 3

-3 6 -3 25.4043 2.13353 4

-3 6 -3 31.5955 1.78633 1

3 -6 3 28.2613 1.75885 3

3 -6 3 30.3886 1.76777 1

3 -6 3 28.7431 2.24191 4

3 -6 3 27.5923 1.31686 6

-4 6 -3 483.396 16.0433 4

-4 6 -3 495.571 15.5929 3

4 -6 3 498.673 16.0987 1

4 -6 3 482.962 15.6825 3

-5 6 -3 79.0680 4.06146 4

5 -6 3 87.3957 3.59265 1

-6 6 -3 8.82506 2.42566 4

6 -6 3 6.15163 1.08307 3

6 -6 3 7.10280 1.31709 1

-7 6 -3 -0.6207 2.35019 4

7 -6 3 0.25682 1.13756 1

7 -6 3 -0.6395 1.06007 3

-8 6 -3 44.4823 4.29437 4

8 -6 3 47.5706 3.09397 1

8 -6 3 49.6042 3.36379 3

9 -6 3 4.13745 1.54131 1

9 -6 3 3.31216 1.51654 3

10 -6 3 1.31166 1.81450 3

10 -6 3 2.36557 1.66409 1

11 -6 3 -1.4287 1.84233 1

12 -6 3 5.05416 2.37601 1

12 -6 3 -2.9297 2.72799 3

13 5 -3 -0.9462 2.68697 3

13 5 -3 -0.8061 2.32689 1

-13 -5 3 -1.8895 3.25540 5

12 5 -3 -0.2451 1.78841 3

12 5 -3 0.81714 1.87453 1

-12 -5 3 1.73181 2.66192 5

11 5 -3 1.07632 1.46273 3

11 5 -3 5.07268 1.64583 1

-11 -5 3 6.90825 2.25120 5

10 5 -3 8.90927 1.53319 1

10 5 -3 7.26269 1.49948 3

-10 -5 3 11.1230 2.01012 5

9 5 -3 59.7239 2.96130 1

9 5 -3 56.9503 3.26538 3

-9 -5 3 58.8787 3.35264 5

8 5 -3 76.3599 3.49204 3

8 5 -3 76.5618 3.20647 1

-8 -5 3 79.2824 3.59778 5

7 5 -3 1.22948 0.77870 1

7 5 -3 0.21490 0.83265 3

-7 -5 3 -0.4719 1.15257 5

-6 -5 3 46.7272 2.51158 5

-5 -5 3 1326.06 40.8860 2

-5 -5 3 1282.90 40.9498 5

4 5 -3 908.908 26.4303 5

-4 -5 3 768.040 26.6001 5

3 5 -3 880.305 26.5526 5

3 5 -3 878.118 26.5553 2

-3 -5 3 835.980 26.5590 2

-3 -5 3 789.053 26.5569 5

2 5 -3 666.088 20.0544 2

2 5 -3 646.557 20.0001 5

2 5 -3 635.308 20.0864 4

-2 -5 3 637.158 20.0646 2

-2 -5 3 600.981 20.0217 5

1 5 -3 1408.68 46.0809 2

1 5 -3 1554.15 46.2730 1

1 5 -3 1541.99 46.3737 3

1 5 -3 1497.24 46.2082 5

1 5 -3 1434.41 46.4479 4

-1 -5 3 1392.89 46.2448 5

-1 -5 3 1520.22 46.6925 2

0 5 -3 606.348 18.9649 1

0 5 -3 609.317 18.9113 3

0 -5 3 622.919 19.1153 4

0 -5 3 604.884 18.9497 1

0 -5 3 578.600 19.0246 2

-1 5 -3 3620.82 108.079 1

-1 5 -3 3330.60 108.280 4

-1 5 -3 3439.59 107.627 3

1 -5 3 3480.80 108.337 4

1 -5 3 3375.89 108.292 1

1 -5 3 3461.83 107.912 3

-2 5 -3 46.6900 2.41432 4

-2 5 -3 57.0323 1.92545 3

-2 5 -3 55.5548 2.18920 1

2 -5 3 51.1107 2.05623 3

2 -5 3 55.7766 2.51302 4

2 -5 3 47.6323 2.12814 1

2 -5 3 52.6036 1.88201 6

-3 5 -3 19.2518 1.11477 3

-3 5 -3 18.0152 1.40009 1

-3 5 -3 12.0828 1.75398 4

3 -5 3 18.1914 1.37348 1

3 -5 3 16.6187 1.01351 6

3 -5 3 17.0828 1.30694 3

3 -5 3 15.3226 1.82055 4

-4 5 -3 2.15865 0.59222 3

-4 5 -3 0.03931 0.80113 1

4 -5 3 0.89985 0.65548 3

-5 5 -3 360.965 12.2204 4

5 -5 3 375.123 11.9452 1

5 -5 3 351.156 11.8027 3

-6 5 -3 117.526 5.12606 4

6 -5 3 115.390 4.50681 1

6 -5 3 114.345 4.37646 3

-7 5 -3 90.9010 4.73940 4

7 -5 3 85.6265 3.76820 3

7 -5 3 95.1350 4.01626 1

-8 5 -3 0.81421 2.59081 4

8 -5 3 0.54819 1.25823 1

8 -5 3 1.68558 1.04201 3

9 -5 3 10.2871 1.63346 3

9 -5 3 15.2088 2.39911 1

10 -5 3 3.02984 1.58646 1

10 -5 3 2.36036 1.78905 3

11 -5 3 6.45578 3.15699 1

11 -5 3 4.62403 2.10764 3

12 -5 3 -1.9014 2.05052 1

12 -5 3 -2.8262 2.53281 3

13 -5 3 -4.7534 3.15491 3

13 -5 3 1.98520 2.60429 1

13 4 -3 0.03429 2.37269 1

13 4 -3 0.59704 2.48038 3

12 4 -3 3.48674 1.88391 1

12 4 -3 0.87924 2.24006 3

-12 -4 3 -0.5131 2.47218 5

11 4 -3 2.07704 1.58414 1

11 4 -3 -0.4608 1.22157 3

-11 -4 3 0.85273 1.93053 5

10 4 -3 15.8892 2.32232 1

10 4 -3 10.6833 1.63527 3

-10 -4 3 15.6978 2.04598 5

9 4 -3 0.48164 0.99007 3

9 4 -3 1.34280 1.12346 1

-9 -4 3 -0.6742 1.37656 5

8 4 -3 17.3440 2.05787 3

8 4 -3 13.1854 1.81087 1

-8 -4 3 17.9600 2.09523 5

7 4 -3 398.623 12.4582 1

7 4 -3 380.824 12.6680 3

-7 -4 3 395.190 12.6076 1

-7 -4 3 373.808 12.6764 5

6 4 -3 619.006 19.6422 1

6 4 -3 620.853 19.7360 3

-6 -4 3 617.637 19.7857 5

5 4 -3 14.1905 1.06148 1

4 4 -3 1.43898 0.72415 5

4 4 -3 1.37253 0.61353 2

-4 -4 3 0.53515 0.67819 2

-4 -4 3 1.79544 0.75970 5

3 4 -3 1527.94 47.2181 2

3 4 -3 1516.05 47.2361 5

-3 -4 3 1451.54 47.8892 5

-3 -4 3 1561.91 47.5737 2

2 4 -3 254.359 7.91259 5

2 4 -3 256.299 7.94120 2

-2 -4 3 252.544 7.96581 2

-2 -4 3 226.670 7.92921 5

1 4 -3 4298.50 131.009 5

1 4 -3 4280.14 130.841 1

1 4 -3 4376.96 131.251 2

1 4 -3 4440.14 130.932 6

-1 -4 3 4093.09 131.414 2

-1 -4 3 3722.46 131.048 5

0 4 -3 156.110 5.01540 1

0 -4 3 156.018 5.20851 4

0 -4 3 153.151 4.97742 1

0 -4 3 152.265 5.06820 2

-1 4 -3 52.0884 2.22646 4

-1 4 -3 55.5428 1.87849 3

-1 4 -3 52.6816 2.04439 1

1 -4 3 56.1244 2.30285 4

1 -4 3 51.8401 2.01560 1

1 -4 3 53.5989 1.91106 3

-2 4 -3 9.81856 1.26011 4

-2 4 -3 9.18226 0.98701 1

-2 4 -3 11.1059 0.74445 3

2 -4 3 8.42969 0.92045 1

2 -4 3 7.80305 1.37681 4

2 -4 3 8.18027 0.81968 3

2 -4 3 7.97557 0.66182 6

-3 4 -3 3378.92 105.916 4

-3 4 -3 3542.36 105.510 3

3 -4 3 3390.52 105.455 4

3 -4 3 3406.52 105.485 6

3 -4 3 3186.19 105.489 3

3 -4 3 3315.33 106.175 1

-4 4 -3 5.85273 1.60795 4

-4 4 -3 6.60421 0.99341 3

-4 4 -3 7.07511 1.00590 1

4 -4 3 7.44653 0.95062 1

4 -4 3 6.85342 1.07732 3

4 -4 3 9.91459 1.63453 4

-5 4 -3 125.920 5.04579 4

5 -4 3 126.694 4.64207 1

-6 4 -3 46.0039 3.39521 4

6 -4 3 52.4151 2.59963 1

6 -4 3 57.9153 2.54734 3

6 -4 3 51.3602 2.20808 5

-7 4 -3 31.3991 3.19335 4

7 -4 3 30.7652 2.43203 1

7 -4 3 34.2025 1.96403 5

7 -4 3 35.4504 2.21861 3

-8 4 -3 15.9035 2.94357 4

8 -4 3 11.3357 1.34577 3

8 -4 3 14.1195 2.17493 1

9 -4 3 12.8258 1.75672 1

9 -4 3 11.8194 1.56578 3

10 -4 3 1.37977 1.61996 1

10 -4 3 0.73104 1.56301 3

11 -4 3 0.79588 2.02575 3

11 -4 3 0.50388 1.68120 1

12 -4 3 -0.4701 1.96108 1

12 -4 3 -1.3178 2.37787 3

13 -4 3 -3.2737 2.92004 3

13 -4 3 1.28576 2.34790 1

13 3 -3 0.48955 2.35255 3

13 3 -3 2.59416 2.36509 1

12 3 -3 3.58623 2.22736 3

12 3 -3 6.55644 2.00479 1

11 3 -3 26.0933 2.95668 1

11 3 -3 25.1816 3.49090 3

-11 -3 3 30.2269 3.27063 5

10 3 -3 0.85256 1.34785 1

10 3 -3 -0.3469 1.06488 3

-10 -3 3 2.32700 1.21309 1

-10 -3 3 -1.5064 1.60698 5

9 3 -3 3.71866 1.20886 1

9 3 -3 3.85650 1.12748 3

-9 -3 3 6.47024 1.45641 5

-9 -3 3 4.26714 1.10209 1

8 3 -3 0.48181 0.97022 1

8 3 -3 3.40538 0.99742 3

-8 -3 3 0.57155 0.86018 1

-8 -3 3 2.39038 1.25486 5

7 3 -3 34.1858 1.97979 1

7 3 -3 35.3020 2.21461 3

-7 -3 3 39.4813 2.24547 5

-7 -3 3 33.3987 1.79249 1

6 3 -3 607.415 18.8009 3

6 3 -3 580.625 18.7257 1

-6 -3 3 551.471 18.5184 1

-6 -3 3 587.720 18.8081 5

-6 -3 3 617.413 18.5172 1

5 3 -3 193.043 6.27983 2

5 3 -3 184.968 6.14587 1

4 3 -3 9.35989 0.72195 1

4 3 -3 9.39544 1.05835 5

4 3 -3 9.58577 0.96197 2

-4 -3 3 10.5127 1.15058 5

-4 -3 3 9.28404 0.95155 2

3 3 -3 103.553 3.44537 5

3 3 -3 104.714 3.41414 2

-3 -3 3 99.0311 3.41416 2

-3 -3 3 93.6094 3.45932 5

2 3 -3 4023.09 119.315 5

2 3 -3 4096.39 119.344 2

-2 -3 3 3875.96 119.408 2

-2 -3 3 3276.92 119.277 5

1 3 -3 3144.05 93.9853 6

1 3 -3 3071.15 93.9907 5

1 3 -3 3002.57 94.3793 4

1 3 -3 3119.43 94.1615 2

-1 -3 3 2772.71 94.0869 5

-1 -3 3 2951.88 94.1769 2

0 3 -3 149.508 5.06356 1

0 3 -3 157.257 5.26337 4

0 -3 3 167.027 5.28419 4

0 -3 3 147.696 5.05902 1

0 -3 3 169.270 5.00090 6

-1 3 -3 812.300 26.0237 4

-1 3 -3 803.075 26.0680 1

-1 3 -3 893.019 25.9754 3

1 -3 3 828.486 25.9466 6

1 -3 3 826.930 26.2654 1

1 -3 3 805.487 25.9219 3

1 -3 3 831.711 26.0435 4

-2 3 -3 126.913 4.06227 3

-2 3 -3 123.635 4.38355 4

-2 3 -3 120.463 4.20302 1

2 -3 3 118.453 4.04295 3

2 -3 3 128.235 4.42659 4

2 -3 3 123.790 4.07564 6

2 -3 3 129.747 4.21541 1

-3 3 -3 159.999 5.67478 4

-3 3 -3 158.830 5.43863 1

3 -3 3 163.289 5.43600 1

3 -3 3 165.461 5.70143 4

3 -3 3 160.133 5.27221 3

-4 3 -3 192.728 7.20817 4

-4 3 -3 191.753 6.64525 1

4 -3 3 207.439 6.32588 5

4 -3 3 195.343 6.92085 4

4 -3 3 197.949 6.46240 3

4 -3 3 193.842 6.61577 1

-5 3 -3 -1.2473 1.42753 4

-5 3 -3 0.33303 0.53499 5

5 -3 3 -0.5777 0.78255 1

5 -3 3 -1.5290 0.99846 3

-6 3 -3 58.4927 2.30277 5

-6 3 -3 53.8007 3.42234 4

6 -3 3 59.4336 2.75930 1

6 -3 3 57.5185 2.58497 3

6 -3 3 62.0332 2.44970 5

-7 3 -3 -0.4050 1.96367 4

-7 3 -3 2.00833 0.94434 5

7 -3 3 2.43892 1.03622 1

7 -3 3 1.19919 0.87796 3

7 -3 3 4.05447 1.05449 5

-8 3 -3 147.440 4.95399 5

-8 3 -3 138.371 6.20388 4

8 -3 3 133.604 5.34234 1

8 -3 3 137.209 5.20428 3

9 -3 3 8.46944 1.31648 3

9 -3 3 12.2752 1.65918 1

10 -3 3 6.17341 1.73938 1

10 -3 3 7.17039 1.64560 3

11 -3 3 -1.9687 1.85072 1

11 -3 3 -1.1451 1.92440 3

12 -3 3 7.94550 2.43924 3

12 -3 3 3.42958 1.92488 1

13 -3 3 -0.8655 2.24493 1

14 2 -3 -2.5898 2.84926 3

14 2 -3 1.16502 2.93920 1

13 2 -3 1.45636 2.62401 3

13 2 -3 2.96219 2.37225 1

12 2 -3 0.93308 1.80158 1

11 2 -3 1.11819 1.62266 3

11 2 -3 2.71603 1.59877 1

10 2 -3 0.57027 1.28628 1

10 2 -3 0.88602 1.11498 3

-10 -2 3 2.12723 1.56423 5

-10 -2 3 -0.5211 1.21862 1

9 2 -3 23.4228 2.26778 3

9 2 -3 24.7046 2.21335 1

-9 -2 3 22.9283 2.19008 1

-9 -2 3 25.9301 2.32184 5

8 2 -3 28.1830 1.98820 1

8 2 -3 29.8355 2.28023 3

-8 -2 3 28.4092 2.16137 5

-8 -2 3 27.3678 2.01421 1

7 2 -3 10.2982 1.48135 1

7 2 -3 11.8111 1.72676 3

-7 -2 3 9.76696 1.43239 1

-7 -2 3 10.3084 1.32809 5

6 2 -3 0.71909 0.73902 3

-6 -2 3 1.68465 0.96341 5

-6 -2 3 2.72843 0.68102 1

-6 -2 3 2.66821 0.66859 1

5 2 -3 9.02162 1.12498 2

5 2 -3 8.73191 0.79306 1

5 2 -3 9.02555 1.02571 5

-5 -2 3 8.63896 0.71073 1

-5 -2 3 8.09663 0.95578 5

-5 -2 3 5.94753 0.85915 1

4 2 -3 2136.34 62.7446 2

4 2 -3 2057.00 63.3812 3

4 2 -3 2062.94 62.8471 1

4 2 -3 1929.60 62.4349 5

-4 -2 3 1802.08 62.8561 5

-4 -2 3 2036.64 62.5451 1

3 2 -3 3058.24 97.5378 3

3 2 -3 2977.85 97.4826 5

3 2 -3 3292.15 97.2924 2

3 2 -3 3236.60 97.0734 1

-3 -2 3 2991.97 97.3442 2

2 2 -3 363.290 11.3082 5

2 2 -3 377.902 11.2919 3

2 2 -3 368.850 11.2815 2

-2 -2 3 328.374 11.3126 5

-2 -2 3 353.397 11.2821 2

1 2 -3 7.58425 0.56436 2

1 2 -3 8.28867 0.53578 6

1 2 -3 8.15683 0.49381 5

-1 -2 3 7.04706 0.58452 2

0 2 -3 1402.27 44.8246 6

0 2 -3 1455.26 44.8369 1

0 -2 3 1466.26 44.8442 6

0 -2 3 1423.00 44.9404 4

0 -2 3 1417.19 44.8858 1

-1 2 -3 1716.36 53.2478 1

-1 2 -3 1637.58 53.3786 4

-1 2 -3 1706.26 53.0157 6

1 -2 3 1647.26 52.7871 3

1 -2 3 1724.49 53.2816 1

1 -2 3 1718.64 53.5114 4

-2 2 -3 258.739 8.64820 4

-2 2 -3 266.411 8.50554 1

2 -2 3 260.111 8.37708 3

2 -2 3 262.487 8.47909 1

2 -2 3 281.192 8.70735 4

2 -2 3 262.303 8.44170 6

-3 2 -3 242.753 7.90232 4

-3 2 -3 239.106 7.70180 1

3 -2 3 233.192 7.63625 6

3 -2 3 232.092 7.55784 3

3 -2 3 232.079 7.65770 1

3 -2 3 237.324 7.89444 4

-4 2 -3 113.680 4.31820 1

-4 2 -3 126.727 4.03955 5

-4 2 -3 127.801 4.63514 4

4 -2 3 125.907 4.09461 5

4 -2 3 120.812 4.61229 4

4 -2 3 125.452 4.18092 3

4 -2 3 118.143 4.31399 1

-5 2 -3 191.906 7.05972 4

-5 2 -3 201.959 6.44871 5

5 -2 3 197.588 6.76236 1

5 -2 3 187.774 6.77345 3

5 -2 3 209.987 6.51853 5

-6 2 -3 330.838 11.2535 4

-6 2 -3 331.661 10.5652 5

6 -2 3 351.829 10.6763 5

6 -2 3 314.111 10.8496 1

6 -2 3 320.662 10.7369 3

-7 2 -3 8.76457 1.09269 5

-7 2 -3 10.9160 2.28309 4

7 -2 3 5.52918 0.91313 3

7 -2 3 7.61927 1.19439 1

-8 2 -3 11.7427 1.58119 5

8 -2 3 12.4085 1.70137 3

8 -2 3 8.68038 1.35808 1

-9 2 -3 3.07823 1.21407 5

9 -2 3 4.65461 1.34963 1

9 -2 3 2.84556 1.16954 3

-10 2 -3 3.89523 1.47844 5

10 -2 3 1.71475 1.57051 1

10 -2 3 0.36969 1.21444 3

11 -2 3 -0.0515 1.56916 3

11 -2 3 0.21055 1.83107 1

12 -2 3 -0.5965 2.07220 3

12 -2 3 1.42809 1.77302 1

13 -2 3 0.80247 2.28461 1

13 -2 3 -0.7059 2.95957 3

14 1 -3 1.04149 3.37223 3

14 1 -3 1.39252 2.95529 1

13 1 -3 1.14104 2.49497 3

13 1 -3 3.82223 2.43106 1

12 1 -3 14.0069 4.00387 3

12 1 -3 9.72163 2.07263 1

11 1 -3 2.84685 1.63137 3

11 1 -3 1.95778 1.66750 1

-11 -1 3 3.34894 1.61361 1

10 1 -3 1.86012 1.20279 3

10 1 -3 3.47679 1.30063 1

-10 -1 3 3.44822 1.42402 1

9 1 -3 76.7718 3.60169 3

9 1 -3 80.1570 3.61483 1

-9 -1 3 76.9449 3.60602 1

-9 -1 3 82.2261 3.61153 5

8 1 -3 66.8036 2.99429 1

8 1 -3 66.2910 3.12738 3

-8 -1 3 68.1507 3.07178 5

-8 -1 3 67.3901 3.04504 1

7 1 -3 4.25821 0.98833 3

7 1 -3 3.49217 0.96001 1

-7 -1 3 3.33202 0.93940 1

-7 -1 3 4.56995 1.12417 5

6 1 -3 9.58430 1.40269 1

6 1 -3 13.0359 1.45259 3

-6 -1 3 12.4708 1.07375 1

-6 -1 3 11.0783 1.30436 5

-6 -1 3 12.1226 1.29364 1

5 1 -3 7.10065 0.80735 2

5 1 -3 6.64267 0.96005 5

5 1 -3 5.35486 0.83795 3

5 1 -3 5.90629 0.71614 1

-5 -1 3 6.32552 0.69252 1

-5 -1 3 6.56489 0.86404 1

4 1 -3 27.2160 1.26831 2

4 1 -3 27.4635 1.32029 5

4 1 -3 27.5853 1.43036 3

4 1 -3 26.2380 1.23462 1

-4 -1 3 24.1227 1.18214 1

-4 -1 3 25.5538 1.36328 5

-4 -1 3 24.5586 1.19191 1

3 1 -3 1.41749 0.34675 1

3 1 -3 0.58604 0.42958 3

3 1 -3 1.10839 0.48301 5

3 1 -3 1.22354 0.42276 2

-3 -1 3 1.32311 0.36471 1

-3 -1 3 1.13395 0.49597 5

2 1 -3 1034.84 31.2877 2

2 1 -3 958.046 31.0866 1

2 1 -3 981.620 31.3821 3

-2 -1 3 959.607 31.5443 5

-2 -1 3 1047.75 31.1942 1

-2 -1 3 1015.14 31.3478 3

-2 -1 3 964.751 31.2857 2

1 1 -3 169.181 5.43416 1

1 1 -3 174.710 5.45355 5

1 1 -3 175.251 5.49495 6

1 1 -3 168.392 5.42950 3

-1 -1 3 169.787 5.42277 3

-1 -1 3 168.357 5.44228 2

-1 -1 3 180.122 5.49630 6

0 1 -3 607.776 19.5630 1

0 1 -3 637.264 19.6508 6

0 1 -3 618.987 19.8041 4

0 -1 3 617.580 19.6731 1

0 -1 3 650.566 19.7374 6

0 -1 3 616.799 19.5874 4

-1 1 -3 1235.42 39.9987 4

-1 1 -3 1299.35 40.0595 1

-1 1 -3 1274.76 39.8500 6

1 -1 3 1310.37 40.0098 6

1 -1 3 1244.48 39.8206 4

1 -1 3 1243.61 39.9296 1

-2 1 -3 481.921 15.1875 4

-2 1 -3 497.205 15.0478 1

-2 1 -3 456.872 14.9073 5

2 -1 3 468.575 15.0110 1

2 -1 3 494.901 15.0562 6

2 -1 3 498.989 15.2259 4

2 -1 3 445.922 14.9791 5

-3 1 -3 910.681 29.0153 1

-3 1 -3 917.219 29.1950 4

-3 1 -3 928.432 29.1492 5

3 -1 3 902.333 29.1938 4

3 -1 3 985.701 29.3937 3

3 -1 3 890.782 28.9803 1

3 -1 3 951.251 29.0556 5

-4 1 -3 240.286 7.70321 5

-4 1 -3 243.049 7.89643 1

-4 1 -3 245.493 8.13952 4

4 -1 3 234.715 7.83956 1

4 -1 3 240.211 7.75222 5

4 -1 3 245.310 8.15707 4

4 -1 3 237.348 7.78639 3

-5 1 -3 827.244 26.3500 5

5 -1 3 855.020 26.4200 5

-6 1 -3 425.913 14.1345 4

6 -1 3 419.211 13.7971 1

6 -1 3 421.674 13.7077 3

6 -1 3 436.655 13.6733 5

-7 1 -3 52.9433 2.38773 5

-7 1 -3 48.8488 3.46244 4

7 -1 3 52.9547 2.56757 3

7 -1 3 53.9498 2.72924 1

-8 1 -3 9.72456 1.23739 5

8 -1 3 6.69228 1.02581 3

8 -1 3 7.78331 1.31695 1

-9 1 -3 56.0875 2.74002 5

9 -1 3 48.1437 3.05478 1

9 -1 3 44.6295 2.72965 3

-10 1 -3 11.6411 1.70534 5

10 -1 3 6.70764 1.34713 3

10 -1 3 10.6969 1.73709 1

-11 1 -3 9.98432 1.98834 5

11 -1 3 8.97706 2.00390 1

11 -1 3 5.77828 1.67544 3

12 -1 3 4.42035 2.30683 1

12 -1 3 2.74776 2.12292 3

13 -1 3 0.61286 2.11382 1

13 -1 3 -1.8006 2.53581 3

14 0 -3 -5.3629 3.41876 3

14 0 -3 -1.1386 3.09348 1

13 0 -3 2.68178 2.32632 1

13 0 -3 -1.1538 2.66877 3

12 0 -3 -2.4676 1.84439 1

12 0 -3 4.25601 3.48047 3

11 0 -3 0.83190 1.49476 3

11 0 -3 -0.7675 1.60680 1

10 0 -3 2.22481 1.30774 1

10 0 -3 1.43485 1.09383 3

-10 0 3 1.20574 1.50484 1

9 0 -3 2.04663 1.12845 3

9 0 -3 2.31053 1.17309 1

-9 0 3 0.94528 1.28021 1

8 0 -3 -0.5229 0.90487 3

8 0 -3 -1.6904 1.01024 1

-8 0 3 -1.3492 1.05825 1

7 0 -3 -1.3068 0.81872 3

7 0 -3 -1.2234 0.89717 1

-7 0 3 0.27443 0.93610 5

6 0 -3 2.48517 0.72858 3

6 0 -3 4.18821 0.83494 1

-6 0 3 4.86913 0.99502 5

-6 0 3 4.39218 0.84254 1

5 0 -3 2.14461 0.80406 5

5 0 -3 -0.2234 1.24490 4

5 0 -3 3.39223 0.70353 1

-5 0 3 2.62644 0.65100 1

-5 0 3 1.04171 0.80677 5

-5 0 3 4.39770 0.72601 1

4 0 -3 0.43223 0.44786 1

4 0 -3 0.98528 0.49357 3

4 0 -3 0.74551 0.47153 5

-4 0 3 0.72170 0.46969 1

-4 0 3 1.20570 0.45240 3

-4 0 3 0.64164 0.54900 5

-4 0 3 0.29682 0.48971 1

3 0 -3 1.11878 0.45080 3

3 0 -3 0.81732 0.35994 5

3 0 -3 1.04368 0.40670 1

3 0 -3 0.83104 0.37915 2

-3 0 3 0.54346 0.34507 1

-3 0 3 1.71184 0.35322 3

-3 0 3 1.33379 0.41570 1

2 0 -3 2.28314 0.30126 2

2 0 -3 2.43189 0.34041 1

2 0 -3 2.17394 0.35438 3

-2 0 3 3.54492 0.42867 1

-2 0 3 2.02105 0.29220 3

-2 0 3 2.66220 0.31316 1

1 0 -3 2.31404 0.36729 6

1 0 -3 2.65960 0.30779 1

1 0 -3 2.56670 0.35663 3

1 0 -3 2.90743 0.23908 2

-1 0 3 3.36463 0.32151 3

-1 0 3 2.92954 0.43483 1

0 0 -3 3.19938 0.48130 4

0 0 -3 2.35996 0.26882 3

0 0 -3 2.94669 0.37718 1

0 0 3 2.73436 0.27955 3

0 0 3 3.54021 0.38522 1

-1 0 -3 2.88511 0.35176 1

-1 0 -3 2.05247 0.36798 6

-1 0 -3 1.87123 0.63120 4

1 0 3 2.44903 0.61560 4

1 0 3 2.28397 0.31503 1

1 0 3 2.14122 0.37444 6

-2 0 -3 0.11357 0.83710 4

-2 0 -3 1.74472 0.42744 5

2 0 3 2.30098 0.90971 4

2 0 3 2.48060 0.42526 1

2 0 3 1.57349 0.46780 5

-3 0 -3 1.01758 0.42973 5

-3 0 -3 1.64751 0.47625 1

-3 0 -3 0.83533 0.91228 4

3 0 3 0.91566 0.48047 5

3 0 3 1.18440 0.41937 1

3 0 3 0.92690 0.39571 6

3 0 3 1.97996 0.93285 4

-4 0 -3 1.62256 0.65376 1

-4 0 -3 4.43348 1.20623 4

4 0 3 1.39131 0.58388 1

4 0 3 2.13519 0.64928 5

4 0 3 1.37180 1.16601 4

-5 0 -3 0.24579 0.64270 5

-5 0 -3 5.25076 1.29878 4

5 0 3 4.46526 1.34053 4

5 0 3 1.95648 0.79107 5

-6 0 -3 4.20605 1.59893 4

6 0 3 0.56218 0.76005 1

6 0 3 1.25209 0.74880 3

6 0 3 1.54964 0.94037 5

-7 0 -3 -1.7359 0.90235 5

-7 0 -3 -3.5110 1.81989 4

7 0 3 -0.6528 0.76168 3

7 0 3 -1.1443 0.93235 1

-8 0 -3 0.79020 1.09513 5

8 0 3 1.39855 0.94461 3

8 0 3 -0.0889 1.08404 1

-9 0 -3 -0.3332 1.17215 5

9 0 3 -1.2313 1.01304 3

9 0 3 -1.2675 1.23699 1

-10 0 -3 1.07626 1.51795 5

10 0 3 1.84789 1.44703 1

10 0 3 0.35052 0.99715 3

-11 0 -3 1.55289 1.77457 5

11 0 3 -0.9157 1.81866 1

11 0 3 1.09579 1.62955 3

-12 0 -3 0.33275 2.30527 5

12 0 3 1.41313 2.02745 3

12 0 3 3.45759 2.27210 1

13 0 3 -1.8424 2.14863 1

13 0 3 -0.6664 2.80577 3

14 -1 -3 -5.8373 3.07878 3

14 -1 -3 3.06195 3.19823 1

13 -1 -3 3.52481 2.59400 1

13 -1 -3 4.64380 2.89518 3

12 -1 -3 12.8483 4.14335 3

12 -1 -3 9.10933 2.23222 1

-12 1 3 9.55300 4.14703 1

11 -1 -3 2.91103 1.75891 3

11 -1 -3 1.34943 1.60137 1

-11 1 3 1.67039 1.76202 1

10 -1 -3 0.37663 1.31114 1

10 -1 -3 2.79338 1.40330 3

-10 1 3 2.89909 1.59878 1

9 -1 -3 72.7550 3.62989 3

9 -1 -3 76.1023 3.59552 1

8 -1 -3 68.0055 3.18511 3

8 -1 -3 63.2850 3.08129 1

-8 1 3 64.0765 3.30023 1

7 -1 -3 3.49653 0.92287 3

7 -1 -3 4.02676 1.00987 1

-7 1 3 4.78207 1.15022 1

6 -1 -3 7.59625 0.98290 1

6 -1 -3 5.77677 0.91689 3

-6 1 3 8.76803 1.06306 1

5 -1 -3 3.06968 1.25884 4

5 -1 -3 2.41625 0.65009 5

-5 1 3 4.46983 0.76200 1

-5 1 3 4.48536 0.78091 1

-5 1 3 3.62740 0.67884 3

4 -1 -3 42.2114 1.73559 3

4 -1 -3 33.0399 1.64741 1

-4 1 3 34.9248 1.57745 1

-4 1 3 33.0470 1.66707 1

-4 1 3 38.1984 1.61064 3

3 -1 -3 2.92170 0.51495 1

3 -1 -3 2.64786 0.44158 5

3 -1 -3 3.15641 0.54452 3

-3 1 3 2.40041 0.39920 1

-3 1 3 4.36480 0.66201 3

-3 1 3 2.89528 0.50422 1

2 -1 -3 949.215 32.1369 3

2 -1 -3 1029.88 32.2344 1

2 -1 -3 1048.48 32.7191 6

-2 1 3 988.422 32.2729 1

-2 1 3 1027.88 32.2360 3

-2 1 3 1093.12 32.3062 1

1 -1 -3 192.508 6.25334 6

1 -1 -3 190.577 6.22718 3

-1 1 3 195.461 6.21613 3

-1 1 3 201.017 6.23908 6

-1 1 3 197.165 6.23817 1

0 -1 -3 702.993 21.5740 1

0 -1 -3 671.661 21.5104 6

0 -1 -3 688.990 21.5824 4

0 -1 -3 668.439 21.5238 3

0 1 3 667.987 21.2545 6

0 1 3 686.340 21.4574 1

0 1 3 673.707 21.6702 3

-1 -1 -3 1377.06 43.6047 4

-1 -1 -3 1353.62 43.7087 6

-1 -1 -3 1380.10 43.3530 1

1 1 3 1389.08 43.6162 4

1 1 3 1373.85 43.5390 1

1 1 3 1412.48 43.7915 6

-2 -1 -3 462.611 14.4525 6

-2 -1 -3 458.075 14.4158 1

-2 -1 -3 457.492 14.4912 4

-2 -1 -3 444.115 14.3693 5

2 1 3 477.254 14.5363 4

2 1 3 464.659 14.4006 5

2 1 3 466.875 14.4711 6

2 1 3 431.466 14.3903 1

-3 -1 -3 833.117 26.9975 1

-3 -1 -3 916.052 27.2802 5

-3 -1 -3 855.070 27.1330 4

3 1 3 865.513 27.1128 6

3 1 3 887.339 27.1857 4

3 1 3 857.111 26.9979 5

3 1 3 825.237 26.9732 1

-4 -1 -3 246.060 7.93029 5

-4 -1 -3 246.696 8.18825 4

-4 -1 -3 251.492 8.01398 1

4 1 3 244.531 7.99220 5

4 1 3 248.743 8.25054 4

4 1 3 243.870 8.12717 6

4 1 3 241.461 7.95677 1

-5 -1 -3 872.370 27.8235 5

-5 -1 -3 894.987 29.0903 4

5 1 3 868.612 27.8975 5

5 1 3 910.914 28.1962 4

-6 -1 -3 465.130 14.9429 4

-6 -1 -3 459.788 14.5059 5

6 1 3 436.525 14.5397 1

-7 -1 -3 46.9779 2.38158 5

7 1 3 48.6666 2.42959 1

7 1 3 45.6300 2.36092 3

-8 -1 -3 10.2382 1.36554 5

8 1 3 6.51814 1.30653 1

8 1 3 5.86860 0.98342 3

-9 -1 -3 59.0088 2.99753 5

9 1 3 49.1619 2.91042 3

9 1 3 55.3268 3.22094 1

10 1 3 11.3111 1.82873 1

10 1 3 7.89637 1.40878 3

-11 -1 -3 10.9884 2.08667 5

11 1 3 6.41415 1.45234 3

11 1 3 9.55911 1.99157 1

-12 -1 -3 12.4464 2.53900 5

12 1 3 4.12668 2.17280 3

12 1 3 7.84902 2.30548 1

-13 -1 -3 -1.8414 2.83239 5

13 1 3 -2.7216 2.67368 3

13 1 3 -0.9695 2.70137 1

14 -2 -3 0.78453 3.26982 1

13 -2 -3 2.90710 3.00551 3

13 -2 -3 6.19165 2.63930 1

-12 2 3 2.00410 2.11806 1

11 -2 -3 -0.3625 1.58245 1

11 -2 -3 1.16769 1.51516 3

-11 2 3 0.16535 1.79211 1

10 -2 -3 1.28049 1.42235 1

10 -2 -3 3.17573 1.57558 3

-10 2 3 2.10280 1.68589 1

9 -2 -3 33.4406 2.77114 3

9 -2 -3 28.3336 2.63065 1

-9 2 3 24.1270 3.04503 1

8 -2 -3 19.6186 2.17234 1

8 -2 -3 25.1957 2.22443 3

-8 2 3 19.3270 2.51139 1

7 -2 -3 16.8503 1.93068 1

7 -2 -3 18.6352 1.92936 3

-7 2 3 14.0248 2.17507 1

6 -2 -3 4.34786 1.00274 1

6 -2 -3 4.83917 0.83381 3

-6 2 3 5.82312 1.12241 1

5 -2 -3 7.61358 0.85286 3

5 -2 -3 6.94732 1.44661 4

5 -2 -3 5.29858 0.96441 1

5 -2 -3 7.45435 0.92322 5

-5 2 3 7.16256 1.12384 3

-5 2 3 4.42315 0.78995 1

-5 2 3 4.98770 0.93169 1

4 -2 -3 2028.29 61.3949 5

4 -2 -3 1969.88 61.6210 1

4 -2 -3 1928.10 60.9282 4

4 -2 -3 1895.90 61.8156 3

-4 2 3 2024.18 61.2683 1

-4 2 3 1946.90 61.1652 1

-4 2 3 1864.63 60.8037 3

3 -2 -3 3271.19 101.088 1

3 -2 -3 3062.07 100.954 3

-3 2 3 3287.51 100.517 1

-3 2 3 3174.65 100.705 1

-3 2 3 3243.93 100.712 3

2 -2 -3 376.981 12.2001 3

2 -2 -3 394.842 12.1450 1

2 -2 -3 387.755 12.1837 6

-2 2 3 374.808 12.1622 1

-2 2 3 389.058 12.1553 3

1 -2 -3 6.66722 0.68169 1

1 -2 -3 8.33742 0.82008 6

1 -2 -3 8.02185 0.82943 3

-1 2 3 9.39617 0.68590 6

-1 2 3 6.99167 0.82317 1

0 -2 -3 1651.93 50.7633 1

0 -2 -3 1630.19 50.6839 4

0 -2 -3 1548.14 50.9073 3

0 -2 -3 1596.12 50.9675 6

0 2 3 1674.72 50.9684 1

0 2 3 1571.87 50.3569 2

0 2 3 1644.23 50.8568 6

-1 -2 -3 1905.40 59.1735 4

-1 -2 -3 1842.25 59.3862 6

-1 -2 -3 1892.09 59.1677 1

1 2 3 1947.12 59.4590 6

1 2 3 1847.23 59.2989 1

-2 -2 -3 274.201 8.91281 5

-2 -2 -3 279.369 8.97344 6

-2 -2 -3 271.271 8.95460 4

-2 -2 -3 287.957 8.95164 1

2 2 3 272.675 8.93766 1

2 2 3 288.230 9.00099 6

2 2 3 283.962 8.94404 5

2 2 3 283.351 8.99864 4

-3 -2 -3 305.300 9.85990 5

-3 -2 -3 304.560 9.96601 4

-3 -2 -3 316.735 9.89332 1

3 2 3 307.167 9.89932 5

3 2 3 311.944 9.98628 6

3 2 3 321.209 10.0437 4

3 2 3 297.834 9.85075 1

-4 -2 -3 150.983 5.07332 5

-4 -2 -3 150.968 5.27111 4

-4 -2 -3 152.957 5.11948 1

4 2 3 150.734 5.14267 5

4 2 3 153.897 5.36192 4

4 2 3 150.514 5.05505 1

-5 -2 -3 170.262 6.09570 5

-5 -2 -3 181.127 6.18277 4

5 2 3 187.389 6.30384 4

5 2 3 165.350 6.10354 1

5 2 3 165.822 5.92839 5

-6 -2 -3 330.418 10.6434 5

-6 -2 -3 326.913 10.9827 4

6 2 3 324.659 10.6249 1

-7 -2 -3 15.4231 1.72163 5

7 2 3 15.5352 1.68383 1

-8 -2 -3 14.0699 1.94243 5

8 2 3 14.1076 1.98646 1

8 2 3 12.5148 1.71548 3

-9 -2 -3 3.77759 1.47980 5

9 2 3 2.07405 1.05115 3

9 2 3 1.88617 1.34109 1

-10 -2 -3 0.57308 1.60663 5

10 2 3 0.57253 1.15325 3

10 2 3 1.61513 1.53290 1

-11 -2 -3 2.18581 1.96221 5

11 2 3 0.30763 1.37039 3

11 2 3 2.39333 1.83910 1

-12 -2 -3 -0.6981 2.33779 5

12 2 3 0.64519 2.16263 1

12 2 3 1.30042 1.37700 3

-13 -2 -3 -1.8541 2.83171 5

13 2 3 -4.3320 2.99862 3

13 2 3 -0.9591 2.65244 1

13 -3 -3 -1.5666 2.31323 3

13 -3 -3 2.65333 2.64756 1

12 -3 -3 5.40193 2.53596 1

12 -3 -3 2.56944 2.15395 3

-12 3 3 3.18414 2.35447 1

11 -3 -3 22.5766 3.10237 1

11 -3 -3 19.8858 2.52235 3

-11 3 3 16.2919 2.56258 1

10 -3 -3 0.91520 1.44423 1

10 -3 -3 1.01903 1.29652 3

-10 3 3 0.59931 1.64639 1

9 -3 -3 3.07110 1.32491 1

9 -3 -3 4.27341 1.29613 3

-9 3 3 5.30495 1.71213 1

8 -3 -3 2.90058 1.11734 3

8 -3 -3 -0.0919 1.17243 1

-8 3 3 -0.7293 1.40255 1

7 -3 -3 25.5996 2.24010 1

7 -3 -3 31.7987 2.26051 3

6 -3 -3 516.409 17.7296 1

6 -3 -3 555.527 17.7110 3

6 -3 -3 573.952 17.6319 5

-6 3 3 556.667 17.7283 1

5 -3 -3 193.710 6.33084 5

-5 3 3 191.875 6.60587 1

4 -3 -3 10.0964 1.24377 3

4 -3 -3 8.70034 1.25350 4

4 -3 -3 10.6975 1.19341 1

4 -3 -3 10.3726 0.84389 5

-4 3 3 8.59659 0.95182 3

-4 3 3 8.47726 1.23355 1

-4 3 3 9.66731 0.95534 1

3 -3 -3 86.1204 3.24089 3

3 -3 -3 90.1161 3.20471 1

3 -3 -3 89.2968 3.16312 6

-3 3 3 88.8074 3.09871 3

-3 3 3 89.2662 3.07440 1

-3 3 3 87.4254 3.18003 1

2 -3 -3 4153.82 127.250 1

2 -3 -3 3996.97 127.386 6

-2 3 3 4188.69 127.358 1

-2 3 3 3981.76 127.156 3

-2 3 3 4010.08 127.481 1

1 -3 -3 3064.60 97.9136 3

1 -3 -3 3080.80 97.7406 6

1 -3 -3 3179.16 97.7518 1

1 -3 -3 3026.02 97.0398 2

-1 3 3 3200.07 97.5941 6

-1 3 3 3185.89 97.7712 3

-1 3 3 3094.04 97.8290 1

0 -3 -3 141.740 4.74371 6

0 -3 -3 141.387 4.73526 3

0 -3 -3 148.499 4.73699 1

0 -3 -3 139.447 4.56297 2

0 3 3 155.198 4.77142 6

0 3 3 145.837 4.74064 3

0 3 3 133.758 4.59304 2

0 3 3 146.405 4.79333 1

-1 -3 -3 923.135 28.9857 5

-1 -3 -3 946.411 29.0003 6

-1 -3 -3 893.047 28.5117 1

1 3 3 905.531 28.5042 2

1 3 3 873.139 28.5337 1

1 3 3 935.178 28.7811 6

-2 -3 -3 132.251 4.49746 6

-2 -3 -3 135.898 4.49130 1

-2 -3 -3 132.148 4.46444 5

2 3 3 132.807 4.39014 2

2 3 3 132.462 4.50376 1

2 3 3 141.947 4.53889 6

-3 -3 -3 175.798 5.97109 4

-3 -3 -3 184.968 5.97255 1

-3 -3 -3 177.605 5.95550 5

3 3 3 177.848 6.04506 6

3 3 3 176.494 5.93757 1

3 3 3 183.230 6.07359 4

3 3 3 184.453 6.00755 5

3 3 3 188.164 5.92071 2

-4 -3 -3 221.495 7.57166 5

-4 -3 -3 232.898 7.55969 1

-4 -3 -3 225.187 7.63505 4

4 3 3 231.049 7.71238 6

4 3 3 232.671 7.77422 4

4 3 3 224.674 7.50444 1

4 3 3 232.501 7.53054 2

4 3 3 244.250 7.65779 5

-5 -3 -3 1.07645 1.23869 4

-5 -3 -3 0.68241 0.80526 5

5 3 3 -0.6409 0.63562 1

5 3 3 1.61550 1.35655 4

-6 -3 -3 60.2911 2.68406 5

6 3 3 61.8235 2.57372 1

-7 -3 -3 3.06557 1.18128 5

7 3 3 2.51070 0.90610 1

-8 -3 -3 163.402 5.86658 5

8 3 3 156.211 5.77305 1

-9 -3 -3 16.4735 2.31739 5

9 3 3 16.8774 2.51691 1

9 3 3 14.6937 2.03709 3

-10 -3 -3 9.80335 2.01204 5

10 3 3 5.60265 1.40214 3

10 3 3 7.93602 1.75969 1

-11 -3 -3 0.46524 1.93228 5

11 3 3 0.06926 1.77683 1

11 3 3 -1.6534 1.12031 3

-12 -3 -3 8.23967 4.16509 5

12 3 3 9.26788 2.49297 1

12 3 3 4.35970 1.53023 3

-13 -3 -3 1.55198 3.11604 5

13 3 3 -0.6262 2.65205 1

13 3 3 -1.0158 1.63612 3

13 -4 -3 -0.1503 2.80232 3

13 -4 -3 -1.6456 2.61351 1

12 -4 -3 0.95358 2.18865 1

12 -4 -3 1.21488 2.24173 3

-12 4 3 4.83161 2.52365 1

11 -4 -3 1.47223 2.01062 3

11 -4 -3 1.77847 1.87580 1

-11 4 3 1.23311 2.20731 1

10 -4 -3 15.8720 2.10085 3

10 -4 -3 12.0757 1.94566 1

-10 4 3 13.0409 2.33461 1

9 -4 -3 1.51137 1.36770 1

9 -4 -3 3.06815 1.37200 3

-9 4 3 0.47378 1.66524 1

8 -4 -3 18.0499 2.38211 3

8 -4 -3 10.7598 1.64196 1

-8 4 3 12.8521 1.93742 1

7 -4 -3 314.849 11.3083 1

7 -4 -3 355.179 11.3081 3

-7 4 3 337.812 11.3685 1

6 -4 -3 592.860 18.0911 5

6 -4 -3 583.000 18.3706 3

6 -4 -3 534.404 18.4091 1

5 -4 -3 15.2214 1.74659 1

5 -4 -3 20.3079 1.20658 5

5 -4 -3 20.2528 1.79030 3

-5 4 3 19.2367 1.82843 1

4 -4 -3 2.21628 1.01557 4

4 -4 -3 2.00967 0.79878 6

4 -4 -3 2.50636 0.75418 3

4 -4 -3 2.30365 0.84799 1

-4 4 3 2.22938 0.81719 1

-4 4 3 0.93647 0.59475 1

-4 4 3 2.75779 0.57255 3

3 -4 -3 1492.15 47.9193 4

3 -4 -3 1565.81 48.5255 6

3 -4 -3 1564.50 48.7349 3

-3 4 3 1488.03 47.8571 3

-3 4 3 1515.51 47.8509 1

-3 4 3 1577.27 48.4594 1

2 -4 -3 245.967 8.01175 1

2 -4 -3 254.448 8.04592 4

2 -4 -3 248.460 8.05318 6

2 -4 -3 243.687 8.14340 3

-2 4 3 259.970 8.04458 1

-2 4 3 248.996 8.06289 3

-2 4 3 237.229 8.08813 1

-2 4 3 255.828 7.97483 6

1 -4 -3 4393.22 141.920 3

1 -4 -3 4452.54 140.937 2

1 -4 -3 4513.86 141.556 1

1 -4 -3 4400.10 141.528 6

-1 4 3 4618.53 141.610 3

-1 4 3 4576.33 141.845 1

-1 4 3 4732.80 141.301 6

0 -4 -3 170.074 5.80557 6

0 -4 -3 177.309 5.67578 2

0 -4 -3 180.477 5.81219 1

0 -4 -3 173.296 5.85651 3

0 4 3 187.819 5.84412 6

0 4 3 180.175 5.67578 2

0 4 3 182.835 5.87733 3

0 4 3 175.059 5.87246 1

-1 -4 -3 68.6176 2.48002 5

-1 -4 -3 66.9333 2.33784 2

-1 -4 -3 65.9416 2.42803 6

-1 -4 -3 70.4737 2.48125 1

1 4 3 68.5431 2.48583 3

1 4 3 66.8687 2.32287 2

1 4 3 68.6636 2.50373 6

1 4 3 68.1761 2.56200 1

-2 -4 -3 4.73266 0.73609 5

-2 -4 -3 6.22357 0.93325 1

-2 -4 -3 5.78714 0.74358 6

2 4 3 5.56166 0.71081 2

2 4 3 6.36396 0.85837 6

2 4 3 5.17545 0.75037 1

-3 -4 -3 3644.08 112.274 1

-3 -4 -3 3481.87 112.902 5

3 4 3 3716.62 112.443 2

3 4 3 3641.37 113.046 6

3 4 3 3573.88 112.516 5

3 4 3 3533.68 112.644 1

-4 -4 -3 11.3293 1.29991 4

-4 -4 -3 13.1539 1.33068 1

4 4 3 8.41210 0.86740 2

4 4 3 11.2710 1.39861 4

4 4 3 7.47746 1.06102 6

4 4 3 9.05345 1.10872 5

-5 -4 -3 137.197 4.76634 5

5 4 3 131.896 5.06851 4

5 4 3 132.726 4.65816 1

-6 -4 -3 66.0198 2.98531 5

6 4 3 76.7068 2.88078 1

6 4 3 68.1675 3.58712 4

-7 -4 -3 26.5239 2.19294 5

7 4 3 29.4038 2.00923 1

-8 -4 -3 19.7955 2.19075 5

8 4 3 18.3479 2.02977 1

-9 -4 -3 16.7461 2.51686 5

9 4 3 16.3150 2.38691 1

9 4 3 16.4230 2.09068 3

-10 -4 -3 2.06049 1.76337 5

10 4 3 1.00634 1.14520 3

10 4 3 1.99370 1.68411 1

-11 -4 -3 3.76816 2.13261 5

11 4 3 0.75286 1.27884 3

11 4 3 3.56700 1.97863 1

-12 -4 -3 1.63177 2.56694 5

12 4 3 -1.0319 1.62749 3

12 4 3 0.07551 2.25283 1

-13 -4 -3 -0.4157 3.14786 5

13 4 3 3.92991 2.70403 1

13 4 3 -2.1465 1.82852 3

13 -5 -3 0.16927 2.83639 1

13 -5 -3 1.49426 2.82896 3

13 -5 -3 2.34956 3.27436 6

-13 5 3 -0.3750 3.65529 1

12 -5 -3 -1.2340 2.33012 1

12 -5 -3 0.97908 2.36236 3

-12 5 3 -0.2799 2.69972 1

11 -5 -3 2.37133 2.02997 1

11 -5 -3 3.35173 2.10661 3

-11 5 3 2.45186 2.44323 1

10 -5 -3 7.85332 1.91030 3

10 -5 -3 7.07242 1.97972 1

-10 5 3 9.31407 2.35001 1

9 -5 -3 47.6967 3.48981 1

9 -5 -3 52.4609 3.54761 3

-9 5 3 50.4750 3.88273 1

8 -5 -3 65.1232 3.46507 3

8 -5 -3 57.2444 3.43734 1

-8 5 3 55.6855 3.60947 1

7 -5 -3 1.20833 1.11844 3

7 -5 -3 0.60793 1.24490 1

-7 5 3 0.81975 1.34553 1

6 -5 -3 36.7069 2.53041 1

6 -5 -3 32.7566 2.38391 6

6 -5 -3 40.2888 2.60910 3

6 -5 -3 41.3496 1.92798 5

-6 5 3 35.3222 2.69028 1

5 -5 -3 1208.48 37.1208 3

5 -5 -3 1141.88 37.1753 1

5 -5 -3 1231.88 36.7882 5

-5 5 3 1121.48 37.3530 1

4 -5 -3 755.072 24.2075 6

4 -5 -3 780.689 24.2757 4

4 -5 -3 776.468 24.3961 1

-4 5 3 749.467 24.1316 3

3 -5 -3 896.530 29.2197 3

3 -5 -3 932.729 29.1680 4

3 -5 -3 907.590 29.1152 6

-3 5 3 952.346 29.2158 1

-3 5 3 946.280 29.1217 3

2 -5 -3 693.335 21.9784 4

2 -5 -3 672.705 22.0943 3

2 -5 -3 683.278 21.9956 6

-2 5 3 719.449 22.0029 1

-2 5 3 695.698 22.0708 1

-2 5 3 737.951 21.9245 6

-2 5 3 690.958 22.0275 3

1 -5 -3 1512.74 47.1846 2

1 -5 -3 1483.39 46.9825 1

1 -5 -3 1512.57 47.2666 6

1 -5 -3 1556.28 47.8983 3

-1 5 3 1478.05 47.0450 3

-1 5 3 1571.65 47.1235 6

-1 5 3 1432.60 47.0356 1

0 -5 -3 566.796 17.5674 1

0 -5 -3 552.427 17.4539 2

0 -5 -3 543.177 17.6318 3

0 -5 -3 543.854 17.5422 6

0 5 3 571.389 17.5669 6

0 5 3 564.832 17.4518 2

0 5 3 563.437 17.6354 3

0 5 3 553.282 17.6330 1

-1 -5 -3 3760.29 117.671 2

-1 -5 -3 3678.59 118.058 5

-1 -5 -3 3695.42 118.021 6

-1 -5 -3 3794.84 117.643 1

1 5 3 3763.63 117.899 1

1 5 3 3845.92 117.934 6

1 5 3 3825.76 117.543 2

-2 -5 -3 68.3191 2.52021 2

-2 -5 -3 67.3192 2.61401 1

2 5 3 67.6354 2.75367 1

2 5 3 76.4157 2.67368 6

2 5 3 70.6121 2.50634 2

-3 -5 -3 11.6685 1.41261 5

-3 -5 -3 11.4231 1.26405 1

3 5 3 12.6773 1.38531 5

3 5 3 14.0508 1.08003 2

3 5 3 12.7502 1.39480 1

3 5 3 11.3518 1.19443 6

-4 -5 -3 0.58079 0.84496 5

-4 -5 -3 0.87760 0.81102 1

4 5 3 -1.5010 1.06774 4

4 5 3 0.73135 0.69913 6

-5 -5 -3 392.649 12.5554 5

5 5 3 376.461 12.6875 4

5 5 3 391.666 12.4298 1

-6 -5 -3 124.950 4.65238 5

6 5 3 116.461 5.04408 4

6 5 3 130.625 4.47764 1

-7 -5 -3 95.4579 3.90626 5

7 5 3 88.8763 3.61622 1

-8 -5 -3 1.70780 1.37706 5

8 5 3 3.79214 1.10476 1

-9 -5 -3 14.5390 1.99763 5

9 5 3 12.5385 1.61334 1

-10 -5 -3 4.22977 1.91034 5

10 5 3 1.85096 1.27155 3

10 5 3 3.74535 1.76111 1

-11 -5 -3 6.64735 2.39764 5

11 5 3 8.04486 2.17178 1

-12 -5 -3 0.65196 2.63618 5

12 5 3 -0.1128 1.72783 3

12 5 3 -0.0825 2.32335 1

-13 -5 -3 -4.6765 3.16522 5

13 5 3 1.14732 1.85236 3

13 5 3 3.45312 2.75821 1

13 -6 -3 -2.2439 3.18917 6

13 -6 -3 -0.2833 3.04913 3

13 -6 -3 2.33856 3.09048 1

-13 6 3 3.93777 3.30033 1

12 -6 -3 -0.1367 2.74898 3

12 -6 -3 0.16459 2.61693 1

-12 6 3 -0.3972 2.90937 1

11 -6 -3 0.97598 2.33665 6

11 -6 -3 -1.0939 2.03273 3

11 -6 -3 0.08082 2.18657 1

-11 6 3 -0.5292 2.42368 1

10 -6 -3 12.9775 2.28637 3

10 -6 -3 9.19883 2.20471 1

10 -6 -3 15.7213 2.38216 6

-10 6 3 14.6773 2.57963 1

9 -6 -3 35.4221 3.34104 1

9 -6 -3 37.8798 3.25433 6

9 -6 -3 41.3157 3.69510 3

-9 6 3 34.6176 3.97482 1

8 -6 -3 1.12147 1.38195 3

8 -6 -3 0.71626 1.53220 6

8 -6 -3 1.64757 1.42219 1

-8 6 3 0.89396 1.58338 1

7 -6 -3 8.77824 1.68380 6

7 -6 -3 7.32674 1.57381 1

7 -6 -3 7.21837 1.52486 3

-7 6 3 6.34404 1.64948 1

6 -6 -3 87.1068 3.79389 3

6 -6 -3 72.9238 3.73534 1

6 -6 -3 79.2130 3.54760 6

-6 6 3 75.2127 3.81994 1

5 -6 -3 449.088 15.3557 1

5 -6 -3 490.488 15.4755 6

5 -6 -3 474.599 15.3093 3

-5 6 3 459.257 15.3156 1

4 -6 -3 31.3633 2.37369 3

4 -6 -3 32.6058 1.91050 6

4 -6 -3 25.3096 2.13541 4

-4 6 3 27.7490 2.21423 1

3 -6 -3 19.8158 1.57669 6

3 -6 -3 18.2715 1.79053 3

3 -6 -3 20.2790 1.57994 4

-3 6 3 18.3587 1.41126 3

-3 6 3 19.2292 1.30000 1

2 -6 -3 416.369 13.1927 4

2 -6 -3 407.806 13.3586 3

2 -6 -3 398.350 13.1966 6

2 -6 -3 400.705 13.0726 2

-2 6 3 428.693 13.0981 6

-2 6 3 430.757 13.2344 1

-2 6 3 415.307 13.2716 3

1 -6 -3 46.1597 1.97128 6

1 -6 -3 44.1138 1.73619 2

1 -6 -3 44.2278 1.89085 1

1 -6 -3 41.8651 2.09098 3

-1 6 3 47.6967 1.84532 6

-1 6 3 42.6687 2.10349 1

-1 6 3 48.2977 2.12219 3

-1 6 3 48.9427 1.96780 1

0 -6 -3 744.214 23.3051 1

0 -6 -3 740.526 23.4146 3

0 -6 -3 728.010 23.2143 2

0 -6 -3 732.330 23.2798 6

0 6 3 746.167 23.2101 2

0 6 3 731.195 23.4016 1

0 6 3 768.388 23.4154 3

0 6 3 743.669 23.2927 6

-1 -6 -3 54.7168 2.26689 5

-1 -6 -3 54.0688 2.11946 2

-1 -6 -3 55.9961 2.20116 1

-1 -6 -3 56.2404 2.15773 6

1 6 3 56.3028 2.29143 3

1 6 3 55.5789 2.42677 1

1 6 3 58.7920 2.29460 6

1 6 3 55.1684 2.08060 2

-2 -6 -3 932.263 30.0606 2

-2 -6 -3 903.461 30.3182 5

-2 -6 -3 976.501 30.0856 1

2 6 3 971.772 30.1672 6

2 6 3 993.274 30.1965 1

2 6 3 972.678 30.0569 2

2 6 3 973.093 30.2140 3

-3 -6 -3 31.4165 1.75513 5

-3 -6 -3 31.9990 1.74576 1

3 6 3 34.7969 1.88757 1

3 6 3 30.5312 1.59184 2

3 6 3 31.0107 1.74090 6

-4 -6 -3 506.445 16.1358 5

4 6 3 500.981 16.0716 1

-5 -6 -3 71.3107 3.13152 5

5 6 3 72.7454 2.97733 1

-6 -6 -3 7.37041 1.47922 5

6 6 3 9.71208 2.08996 4

6 6 3 4.60961 1.07892 1

-7 -6 -3 0.15318 1.27730 5

7 6 3 -0.8881 0.94352 1

-8 -6 -3 53.2848 3.25528 5

8 6 3 57.5622 3.02187 1

-9 -6 -3 7.29020 1.88925 5

9 6 3 6.01596 1.52466 1

-10 -6 -3 1.69590 2.05363 5

10 6 3 1.62181 1.35640 3

10 6 3 3.18094 1.78020 1

11 6 3 -1.9358 2.02730 1

11 6 3 -0.4542 1.32723 3

-12 -6 -3 -1.7453 2.83063 5

12 6 3 2.43029 2.32761 1

12 6 3 3.17056 1.73948 3

13 -7 -3 -1.0021 3.34924 6

13 -7 -3 -5.6914 3.53327 3

-13 7 3 4.71293 3.77225 1

12 -7 -3 -1.0275 3.09928 6

12 -7 -3 1.09934 3.07626 3

12 -7 -3 -0.9137 2.82590 1

-12 7 3 2.31969 3.15844 1

11 -7 -3 3.71787 2.77448 3

11 -7 -3 6.47855 2.65205 6

11 -7 -3 -6.6544 3.88176 1

-11 7 3 3.59769 2.78867 1

10 -7 -3 -0.1336 2.20256 3

10 -7 -3 -0.9594 2.09260 1

10 -7 -3 -2.1122 2.15418 6

-10 7 3 1.42684 2.45241 1

9 -7 -3 10.4517 1.95232 3

9 -7 -3 11.1257 2.15950 6

9 -7 -3 6.93775 1.96394 1

-9 7 3 9.02024 2.35947 1

8 -7 -3 95.0524 4.45055 6

8 -7 -3 101.119 4.81798 3

8 -7 -3 87.0152 4.76317 1

-8 7 3 88.3409 4.97677 1

7 -7 -3 170.874 7.20799 1

7 -7 -3 186.804 6.90130 6

7 -7 -3 205.171 7.14388 3

-7 7 3 180.173 7.17224 1

6 -7 -3 22.7587 2.22676 6

6 -7 -3 20.7160 2.74578 3

6 -7 -3 17.1787 2.52084 1

-6 7 3 16.7755 2.68023 1

5 -7 -3 66.5689 3.43896 3

5 -7 -3 63.7938 3.21701 6

-5 7 3 56.9095 3.40577 1

4 -7 -3 68.2057 3.00809 6

4 -7 -3 70.6967 3.47487 3

4 -7 -3 61.5071 3.09588 4

-4 7 3 67.1561 3.35700 1

3 -7 -3 163.838 5.64831 3

3 -7 -3 144.216 5.39004 4

3 -7 -3 158.727 5.38053 6

-3 7 3 151.056 5.61349 1

-3 7 3 149.787 5.28278 1

2 -7 -3 492.322 15.8093 2

2 -7 -3 486.494 16.3503 3

2 -7 -3 499.121 15.9150 6

-2 7 3 530.526 15.9718 1

-2 7 3 506.506 16.0913 1

-2 7 3 488.661 15.9941 3

1 -7 -3 36.6159 1.67236 2

1 -7 -3 36.0431 1.80492 6

-1 7 3 37.0325 2.09833 3

-1 7 3 40.1531 1.73942 6

-1 7 3 39.3378 1.85504 1

0 -7 -3 1191.29 37.4836 1

0 -7 -3 1194.55 37.4220 2

0 -7 -3 1223.28 37.7520 6

0 -7 -3 1161.25 37.4979 5

0 7 3 1197.17 37.4111 2

0 7 3 1244.91 37.4781 6

0 7 3 1235.83 37.6325 3

0 7 3 1139.49 37.7736 1

-1 -7 -3 604.573 19.3524 2

-1 -7 -3 608.906 19.3845 1

-1 -7 -3 611.470 19.3805 6

-1 -7 -3 579.693 19.6132 5

1 7 3 601.896 19.6939 1

1 7 3 635.655 19.3508 2

1 7 3 622.927 19.5441 3

1 7 3 651.179 19.4611 6

-2 -7 -3 844.683 26.9407 2

-2 -7 -3 849.337 26.8873 1

2 7 3 861.307 26.8901 2

2 7 3 872.053 26.9820 6

-3 -7 -3 1.33646 0.96712 5

3 7 3 0.54390 1.02335 1

3 7 3 1.39092 0.91174 6

-4 -7 -3 528.893 17.1578 5

4 7 3 542.202 17.1128 1

-5 -7 -3 7.17139 1.41680 5

5 7 3 5.96323 1.13466 1

-6 -7 -3 5.41910 1.38290 5

6 7 3 7.16593 1.11728 1

-7 -7 -3 12.3144 1.65661 5

7 7 3 11.3070 1.37362 1

-8 -7 -3 -0.4199 1.47755 5

8 7 3 -0.3336 1.19111 1

-9 -7 -3 20.6686 3.08606 5

9 7 3 24.8984 2.79106 1

-10 -7 -3 2.36587 2.21189 5

10 7 3 0.90798 1.82837 1

-11 -7 -3 2.58480 2.63301 5

11 7 3 -0.9341 2.14275 1

11 7 3 0.56111 1.36315 3

-12 -7 -3 3.38943 3.19035 5

12 7 3 1.35905 1.95620 3

12 7 3 3.69706 2.49461 1

12 -8 -3 -5.4487 3.03202 6

12 -8 -3 -3.2280 2.94396 3

11 -8 -3 -2.8129 2.71088 6

11 -8 -3 -0.6646 3.00547 3

-11 8 3 5.91458 3.12007 1

10 -8 -3 4.44946 2.34797 3

10 -8 -3 7.63203 2.45825 1

10 -8 -3 5.15497 2.48985 6

-10 8 3 5.46312 2.75719 1

9 -8 -3 -0.2014 1.90901 3

9 -8 -3 -0.2269 2.04603 1

9 -8 -3 -3.3815 1.99340 6

-9 8 3 0.00375 2.24024 1

8 -8 -3 2.16711 1.86983 6

8 -8 -3 1.97360 1.72253 3

8 -8 -3 1.36326 1.80781 1

-8 8 3 2.02857 2.05148 1

7 -8 -3 12.8556 1.93065 3

7 -8 -3 15.4148 2.13139 6

-7 8 3 8.80199 2.10896 1

6 -8 -3 4.13379 1.51996 6

6 -8 -3 4.03458 1.39976 3

-6 8 3 2.79565 1.57312 1

5 -8 -3 109.858 4.48426 6

5 -8 -3 119.910 4.99029 3

-5 8 3 111.518 4.89232 1

4 -8 -3 1.87402 1.15032 3

4 -8 -3 0.79375 1.24611 6

4 -8 -3 0.00210 1.49894 4

-4 8 3 -1.7805 1.30149 1

3 -8 -3 38.3998 2.62826 3

3 -8 -3 43.4775 2.41553 4

3 -8 -3 39.6007 2.27754 6

3 -8 -3 39.8863 2.00713 2

-3 8 3 36.0395 2.66727 1

2 -8 -3 252.343 8.49626 3

2 -8 -3 247.144 8.19844 6

2 -8 -3 250.961 8.12530 2

-2 8 3 240.499 8.47321 1

-2 8 3 256.441 8.27271 1

1 -8 -3 8.64895 1.23796 6

1 -8 -3 10.0994 1.24437 2

1 -8 -3 9.85072 1.13319 3

-1 8 3 12.0896 1.41767 1

-1 8 3 12.0093 1.20356 6

-1 8 3 12.4036 1.88803 1

0 -8 -3 84.4207 3.05673 2

0 -8 -3 80.9016 2.97315 6

0 8 3 86.4387 3.12851 6

0 8 3 75.5374 3.42195 1

-1 -8 -3 100.422 3.47749 6

-1 -8 -3 90.3205 3.72178 2

1 8 3 99.6724 3.50660 2

1 8 3 98.4435 3.66036 6

1 8 3 98.0728 3.89163 1

-2 -8 -3 383.517 12.7002 5

2 8 3 403.126 12.7139 6

2 8 3 394.221 12.8408 1

-3 -8 -3 -0.4452 1.06338 5

-3 -8 -3 0.43471 0.85181 2

3 8 3 2.18766 1.14900 6

3 8 3 1.81266 1.13900 1

-4 -8 -3 1.48084 1.11321 5

4 8 3 1.77173 1.11364 1

-5 -8 -3 -0.0428 1.17545 5

5 8 3 -0.3119 1.01219 1

-6 -8 -3 0.71363 1.28825 5

6 8 3 2.08451 1.03101 1

-7 -8 -3 127.303 5.24286 5

7 8 3 133.120 4.95019 1

-8 -8 -3 39.1780 3.09622 5

8 8 3 45.6768 2.78549 1

-9 -8 -3 1.41098 2.06505 5

9 8 3 -0.2450 1.52972 1

-10 -8 -3 1.55694 3.85658 5

10 8 3 0.80352 1.79269 1

-11 -8 -3 10.9848 3.08952 5

11 8 3 7.48124 3.67569 1

-12 -8 -3 -3.1949 3.32377 5

12 8 3 0.35619 1.92425 3

12 8 3 0.85947 2.49297 1

12 -9 -3 -4.9025 3.32372 3

12 -9 -3 -2.2737 3.29370 6

-12 9 3 3.54711 3.82317 1

11 -9 -3 0.72077 2.93159 3

11 -9 -3 -1.0602 2.94587 6

-11 9 3 -1.1668 3.28374 1

10 -9 -3 2.78537 2.57530 6

10 -9 -3 3.72449 2.50674 3

-10 9 3 4.41104 2.90798 1

9 -9 -3 3.36227 2.28943 6

9 -9 -3 2.38334 2.24118 3

-9 9 3 6.74481 2.77170 1

8 -9 -3 8.67169 2.10512 3

8 -9 -3 6.66616 2.08101 6

-8 9 3 9.49422 2.36888 1

7 -9 -3 8.06847 1.88707 3

7 -9 -3 10.9164 2.01362 6

-7 9 3 6.80655 2.19894 1

6 -9 -3 20.8299 2.68915 6

6 -9 -3 27.3576 3.30813 3

-6 9 3 18.1534 2.48251 1

5 -9 -3 32.7418 2.97515 3

5 -9 -3 30.8257 2.43301 6

-5 9 3 23.2833 3.17349 1

4 -9 -3 53.1774 3.23026 3

4 -9 -3 52.9461 2.84063 6

-4 9 3 55.8938 3.40321 1

3 -9 -3 64.0814 2.90755 6

3 -9 -3 64.8752 3.30283 3

3 -9 -3 64.4369 2.70017 2

3 -9 -3 62.8755 2.95117 4

-3 9 3 58.0273 3.39409 1

2 -9 -3 52.5482 2.82315 3

2 -9 -3 48.0415 2.29987 6

-2 9 3 46.4465 2.35320 1

-2 9 3 48.0649 2.91499 1

1 -9 -3 4.26391 0.87707 6

1 -9 -3 2.79863 0.98571 3

1 -9 -3 1.52524 1.20847 5

-1 9 3 4.32722 0.95321 1

-1 9 3 4.64466 1.32695 1

-1 9 3 5.91503 0.92095 6

0 -9 -3 1.68129 0.71037 6

0 -9 -3 0.94247 0.82997 2

0 -9 -3 0.69955 1.00529 5

0 9 3 1.46315 1.21126 1

0 9 3 0.03149 0.91330 1

0 9 3 1.84068 1.04448 6

-1 -9 -3 82.9495 3.28890 2

-1 -9 -3 88.5733 3.43890 5

1 9 3 86.2346 3.73609 1

1 9 3 87.2752 3.41470 6

-2 -9 -3 7.39838 1.26833 5

-2 -9 -3 7.40873 1.06509 2

2 9 3 10.7167 1.71594 6

2 9 3 7.64900 1.42565 1

-3 -9 -3 65.7043 2.95861 2

-3 -9 -3 69.0439 3.12194 5

3 9 3 71.5020 3.33754 1

3 9 3 73.1449 3.13039 6

-4 -9 -3 66.7001 3.19213 5

4 9 3 69.3914 3.21903 1

-5 -9 -3 24.9617 2.27767 5

5 9 3 31.2552 2.23580 1

-6 -9 -3 8.13492 1.72767 5

6 9 3 4.72634 1.32308 1

-7 -9 -3 -0.1630 1.47139 5

7 9 3 0.56544 1.20136 1

-8 -9 -3 6.39568 2.06931 5

8 9 3 3.62640 1.43986 1

-9 -9 -3 1.90602 2.10824 5

9 9 3 0.35353 1.56999 1

-10 -9 -3 1.97866 2.79235 5

10 9 3 -1.0401 1.94508 1

-11 -9 -3 2.01180 3.00271 5

11 9 3 0.35423 1.78644 3

11 9 3 2.55648 2.72144 1

12 9 3 3.45377 2.72285 1

12 9 3 -0.1001 2.37067 3

12 -10 -3 -1.3356 3.59759 3

12 -10 -3 -3.4034 3.47875 6

-12 10 3 -1.3574 4.23188 1

11 -10 -3 -2.0025 2.92006 6

11 -10 -3 -5.3402 2.75272 3

-11 10 3 3.35200 3.53045 1

10 -10 -3 -3.6442 2.54459 6

10 -10 -3 -5.8930 3.06834 3

-10 10 3 3.59895 3.20574 1

9 -10 -3 14.4872 2.73824 6

9 -10 -3 13.7708 2.64755 3

-9 10 3 14.5370 3.05902 1

8 -10 -3 18.0576 2.56411 6

8 -10 -3 10.5119 2.50363 3

-8 10 3 15.7018 2.85072 1

7 -10 -3 14.2325 2.23006 6

7 -10 -3 13.0488 2.06573 3

-7 10 3 13.7828 2.63236 1

6 -10 -3 11.0703 1.83481 3

6 -10 -3 12.1848 1.99365 6

-6 10 3 8.65243 2.30876 1

5 -10 -3 61.3052 3.69428 3

5 -10 -3 58.4333 3.32141 6

-5 10 3 55.9702 3.94505 1

4 -10 -3 3.51093 1.42537 6

4 -10 -3 3.50829 1.27182 3

4 -10 -3 2.12597 1.12916 2

-4 10 3 2.90749 1.71041 1

3 -10 -3 29.1948 1.98223 2

3 -10 -3 29.3634 2.08548 6

3 -10 -3 30.6673 2.66377 3

-3 10 3 27.9282 2.81806 1

2 -10 -3 29.6052 1.75971 6

2 -10 -3 31.9969 2.50704 3

2 -10 -3 32.8163 1.98255 2

-2 10 3 31.8282 2.77169 1

1 -10 -3 110.607 4.15067 5

1 -10 -3 115.161 4.44279 3

1 -10 -3 114.291 4.11334 2

1 -10 -3 107.951 3.87781 6

-1 10 3 109.432 4.63259 1

-1 10 3 112.934 4.24623 1

-1 10 3 112.611 3.88381 6

0 -10 -3 33.4834 1.64594 6

0 -10 -3 29.4101 1.91684 2

0 -10 -3 34.2077 2.06922 5

0 10 3 29.5980 2.09045 1

0 10 3 31.0136 2.68535 1

0 10 3 30.8521 1.90193 6

-1 -10 -3 292.913 9.54157 2

-1 -10 -3 292.780 9.60075 5

1 10 3 289.918 9.82955 1

1 10 3 285.740 9.53507 6

-2 -10 -3 325.481 10.7215 5

-2 -10 -3 316.583 10.6332 2

2 10 3 319.731 10.7114 6

2 10 3 339.002 10.9302 1

-3 -10 -3 39.1364 2.41790 5

-3 -10 -3 38.3827 2.23412 2

3 10 3 31.7739 2.67167 1

-4 -10 -3 2.35525 1.32489 5

4 10 3 4.15636 1.26886 1

-5 -10 -3 30.5812 2.56403 5

5 10 3 22.8098 2.50521 1

-6 -10 -3 3.01544 1.77080 5

6 10 3 4.11173 1.34367 1

-7 -10 -3 12.4659 2.03219 5

7 10 3 9.75723 1.59579 1

-8 -10 -3 1.40769 1.99455 5

8 10 3 2.05666 1.46324 1

9 10 3 7.02673 1.84231 1

-10 -10 -3 7.81206 3.13421 5

10 10 3 5.62189 2.13215 1

-11 -10 -3 4.18461 3.28993 5

11 10 3 6.04684 2.55970 1

11 -11 -3 -0.5790 3.09233 3

11 -11 -3 -7.1956 3.25421 6

-11 11 3 1.92949 3.99153 1

10 -11 -3 -3.3814 2.65033 6

10 -11 -3 -3.7095 2.92656 3

-10 11 3 0.02871 3.48578 1

9 -11 -3 2.41294 2.48582 6

9 -11 -3 -0.0741 2.39409 3

-9 11 3 4.16423 3.21289 1

8 -11 -3 34.1935 4.48062 3

8 -11 -3 29.9143 3.61370 6

-8 11 3 25.2439 3.57139 1

7 -11 -3 27.5086 3.21003 6

7 -11 -3 29.1294 4.00581 3

-7 11 3 32.9138 4.59206 1

6 -11 -3 2.03634 1.50611 3

6 -11 -3 -0.7021 1.65966 6

5 -11 -3 2.00193 1.22762 2

5 -11 -3 -0.3685 1.48259 3

-5 11 3 -0.2364 1.92042 1

4 -11 -3 2.87069 1.17365 2

4 -11 -3 3.18547 1.33208 3

4 -11 -3 3.94107 1.42869 6

-4 11 3 0.69419 1.85305 1

3 -11 -3 25.5669 1.79773 6

3 -11 -3 27.6344 2.70937 3

3 -11 -3 27.4878 2.12643 2

-3 11 3 23.4722 2.94489 1

2 -11 -3 25.9131 2.06394 5

2 -11 -3 26.7539 2.42186 3

2 -11 -3 26.2024 2.06950 2

-2 11 3 26.4635 2.94517 1

1 -11 -3 6.80056 1.25960 5

1 -11 -3 9.07348 1.29276 2

-1 11 3 7.99614 1.68419 1

0 -11 -3 235.253 7.92978 5

0 -11 -3 238.308 7.93452 2

0 11 3 239.164 8.29665 1

0 11 3 229.934 7.73139 6

0 11 3 234.763 7.98961 1

-1 -11 -3 86.4817 3.54139 5

-1 -11 -3 85.9489 3.50795 2

1 11 3 88.7340 3.63244 1

1 11 3 87.9839 3.96792 1

1 11 3 80.4659 3.46715 6

-2 -11 -3 118.414 4.42066 5

-2 -11 -3 110.973 4.31126 2

2 11 3 105.075 4.39572 6

2 11 3 119.993 4.80049 1

-3 -11 -3 0.16283 1.26414 5

-3 -11 -3 0.02099 1.06205 2

3 11 3 -0.2662 1.41404 1

-4 -11 -3 85.1451 3.92899 5

4 11 3 90.8277 3.99875 1

-5 -11 -3 5.21752 1.72642 5

5 11 3 6.80375 1.55166 1

-6 -11 -3 10.9236 1.96415 5

6 11 3 11.2263 1.64593 1

-7 -11 -3 4.89431 1.95165 5

7 11 3 5.05882 1.52876 1

-8 -11 -3 1.07774 2.23037 5

8 11 3 0.63852 1.54239 1

-9 -11 -3 10.5852 2.96260 5

9 11 3 10.3498 2.07269 1

-10 -11 -3 5.24852 3.32624 5

10 11 3 -4.4046 2.08790 1

-11 -11 -3 0.46382 3.54456 5

11 11 3 -0.0476 2.49603 1

10 -12 -3 3.73291 2.76269 3

-10 12 3 7.48330 3.95296 1

9 -12 -3 -2.6757 2.39935 6

9 -12 -3 -1.5550 3.16364 3

-9 12 3 3.81388 3.46170 1

8 -12 -3 10.1964 2.37281 6

8 -12 -3 11.6084 2.49634 3

7 -12 -3 17.5145 2.32426 6

7 -12 -3 15.6419 2.49563 3

-7 12 3 14.5275 3.20454 1

6 -12 -3 20.1378 2.40775 2

6 -12 -3 17.5339 2.21692 6

6 -12 -3 13.4979 2.13883 3

-6 12 3 15.1609 2.88073 1

5 -12 -3 31.4686 2.50629 2

5 -12 -3 33.4168 3.47652 3

5 -12 -3 30.2973 2.55228 6

-5 12 3 24.7533 2.98353 1

4 -12 -3 3.58607 1.32885 2

4 -12 -3 2.77534 1.47016 3

-4 12 3 1.10348 1.95038 1

3 -12 -3 7.74943 1.40897 5

3 -12 -3 7.52760 1.51907 3

3 -12 -3 9.25302 1.39218 2

-3 12 3 7.70073 2.04875 1

2 -12 -3 13.1870 1.51393 2

2 -12 -3 10.7804 1.42479 3

2 -12 -3 15.0810 1.96181 5

-2 12 3 13.0907 2.09978 1

1 -12 -3 34.8129 2.32064 5

1 -12 -3 32.4662 2.30560 2

-1 12 3 34.2715 3.18369 1

0 -12 -3 98.6303 4.00039 2

0 -12 -3 99.4020 3.95049 5

0 12 3 102.250 4.61799 1

0 12 3 95.5258 3.99703 1

-1 -12 -3 12.5230 1.48546 2

-1 -12 -3 11.9543 1.52216 5

1 12 3 10.9061 1.57318 6

1 12 3 9.60256 1.45654 1

1 12 3 9.41061 1.86529 1

-2 -12 -3 39.6770 2.46469 2

-2 -12 -3 42.7944 2.70155 5

2 12 3 37.0940 2.62360 1

2 12 3 40.9732 3.19604 1

-3 -12 -3 1.05450 1.18288 2

-3 -12 -3 1.73755 1.44478 5

3 12 3 0.40361 1.63719 1

-4 -12 -3 142.551 5.48632 5

4 12 3 134.211 5.54683 1

-5 -12 -3 51.1347 3.38812 5

5 12 3 52.5230 3.25017 1

-6 -12 -3 5.25724 1.94090 5

6 12 3 0.58253 1.47636 1

-7 -12 -3 -0.1037 2.10068 5

7 12 3 -0.1914 1.50705 1

-8 -12 -3 5.41930 2.78463 5

8 12 3 0.61087 1.71145 1

-9 -12 -3 -1.6889 2.81311 5

9 12 3 -2.4559 1.89108 1

-10 -12 -3 -2.0386 3.37849 5

10 12 3 -3.9217 2.88689 1

10 -13 -3 7.97099 3.18714 3

-10 13 3 10.8498 4.24830 1

9 -13 -3 -2.5892 2.48450 3

-9 13 3 1.50030 3.64728 1

8 -13 -3 1.86697 2.36575 3

8 -13 -3 0.75577 1.82589 2

-8 13 3 3.20973 3.35268 1

7 -13 -3 0.18133 2.03006 3

7 -13 -3 1.09057 1.63092 2

-7 13 3 -0.7873 3.09421 1

6 -13 -3 16.8650 2.42961 3

6 -13 -3 12.7674 1.89005 2

-6 13 3 15.0297 3.11858 1

5 -13 -3 10.0290 1.73630 2

5 -13 -3 7.75764 1.86500 3

-5 13 3 7.68244 2.63362 1

4 -13 -3 9.76228 1.87309 3

4 -13 -3 12.0073 1.70966 2

4 -13 -3 11.1137 1.56727 5

-4 13 3 9.48468 2.48080 1

3 -13 -3 110.324 4.93274 3

3 -13 -3 106.350 4.15026 5

3 -13 -3 99.1810 4.33453 2

-3 13 3 98.4755 5.28669 1

2 -13 -3 12.5788 1.66767 2

2 -13 -3 13.5131 1.62056 5

-2 13 3 11.2298 2.15149 1

1 -13 -3 23.4913 2.30983 2

1 -13 -3 26.0212 2.23389 5

-1 13 3 20.1287 2.38871 1

0 -13 -3 2.82324 1.46822 5

0 -13 -3 4.84633 1.30495 2

0 13 3 1.08718 1.78125 1

-1 -13 -3 106.149 4.32018 2

-1 -13 -3 105.016 4.38314 5

1 13 3 107.164 4.90174 1

-2 -13 -3 29.8780 2.41214 2

-2 -13 -3 26.3723 2.55535 5

2 13 3 29.3090 3.25306 1

-3 -13 -3 60.5303 3.31649 5

-3 -13 -3 58.8246 3.18271 2

3 13 3 50.7660 3.75147 1

-4 -13 -3 8.97162 1.92814 5

4 13 3 8.89099 1.88735 1

-5 -13 -3 27.5091 3.08231 5

5 13 3 24.4927 2.93015 1

-6 -13 -3 1.55166 2.05179 5

6 13 3 0.75859 1.61500 1

-7 -13 -3 9.87572 3.82474 5

7 13 3 6.88744 1.84047 1

-8 -13 -3 1.52841 2.79532 5

8 13 3 -0.0196 1.79334 1

-9 -13 -3 4.08977 3.17336 5

9 -14 -3 6.95591 2.93258 3

9 -14 -3 1.52093 2.22333 2

-9 14 3 4.44214 4.18171 1

8 -14 -3 10.4476 2.79839 3

8 -14 -3 9.06327 2.15193 2

-8 14 3 8.35433 3.92497 1

7 -14 -3 6.14583 2.43937 3

7 -14 -3 5.28110 1.91508 2

-7 14 3 1.31299 3.41737 1

6 -14 -3 7.50313 2.22774 3

6 -14 -3 6.75886 1.76410 2

5 -14 -3 7.45501 1.84053 2

5 -14 -3 5.69584 1.96054 3

-5 14 3 6.21335 3.01226 1

4 -14 -3 2.40410 1.60294 3

4 -14 -3 4.62627 1.35669 5

4 -14 -3 2.81770 1.61298 2

-4 14 3 0.11647 2.52108 1

3 -14 -3 17.6389 2.26700 5

3 -14 -3 18.4433 2.60973 2

3 -14 -3 15.6264 2.00630 3

-3 14 3 14.5708 2.74336 1

2 -14 -3 0.05863 1.39210 5

2 -14 -3 -1.8753 1.36724 2

-2 14 3 -1.5388 2.17593 1

1 -14 -3 128.521 5.24044 2

1 -14 -3 138.618 5.12302 5

-1 14 3 129.250 6.00011 1

0 -14 -3 -1.5224 1.48131 5

0 -14 -3 -1.1079 1.30893 2

0 14 3 1.93425 1.96972 1

-1 -14 -3 7.65613 1.70821 5

-1 -14 -3 7.46035 1.57258 2

1 14 3 6.42617 2.10553 1

-2 -14 -3 16.3439 1.86897 2

-2 -14 -3 17.8865 2.56418 5

2 14 3 15.7758 2.31007 1

-3 -14 -3 82.1646 4.06137 5

-3 -14 -3 76.9639 3.96180 2

3 14 3 83.3090 4.49977 1

-4 -14 -3 1.92386 1.85580 5

4 14 3 1.72861 1.86299 1

-5 -14 -3 9.67083 2.25922 5

5 14 3 8.83409 1.97496 1

-6 -14 -3 5.10202 2.36084 5

6 14 3 -0.2846 1.72139 1

-7 -14 -3 20.0374 3.10905 5

7 14 3 13.4449 2.12324 1

-8 -14 -3 1.81765 3.14509 5

-9 -14 -3 -3.9105 3.42420 5

9 14 3 -3.9790 2.12822 1

8 -15 -3 2.75271 2.83305 3

8 -15 -3 7.27824 2.41982 2

-8 15 3 1.62569 4.06798 1

7 -15 -3 0.38114 1.96376 2

7 -15 -3 5.47113 2.51317 3

-7 15 3 0.63814 3.79235 1

6 -15 -3 7.43251 2.31993 3

6 -15 -3 8.23437 2.18640 2

-6 15 3 3.81789 3.51599 1

5 -15 -3 5.98026 1.97576 2

5 -15 -3 4.84104 2.02881 3

-5 15 3 -1.0647 3.22924 1

4 -15 -3 2.12725 1.75441 2

4 -15 -3 0.33810 1.71880 3

-4 15 3 -2.2818 2.83064 1

3 -15 -3 13.9968 1.71066 5

3 -15 -3 13.4381 2.09702 2

-3 15 3 7.17572 2.85620 1

2 -15 -3 42.0776 3.26553 2

2 -15 -3 45.2466 2.98806 5

-2 15 3 45.1394 4.67259 1

1 -15 -3 11.6627 1.91163 2

1 -15 -3 11.4323 1.82615 5

-1 15 3 11.4466 2.54095 1

0 -15 -3 0.39775 1.46260 2

0 -15 -3 1.18118 1.54410 5

0 15 3 -1.0145 2.15767 1

-1 -15 -3 23.1023 2.80531 2

-1 -15 -3 22.1650 2.73163 5

1 15 3 29.5014 3.80911 1

-2 -15 -3 2.46804 1.77188 5

-2 -15 -3 0.77457 1.58266 2

2 15 3 4.25280 2.14908 1

-3 -15 -3 -1.1934 1.85486 5

-3 -15 -3 0.47418 1.64481 2

3 15 3 0.97587 2.16291 1

-4 -15 -3 2.42743 2.05497 5

4 15 3 0.76079 2.04128 1

-5 -15 -3 19.2737 2.76443 5

5 15 3 16.0200 2.29454 1

-6 -15 -3 8.90429 2.76742 5

6 15 3 5.90332 2.05313 1

-7 -15 -3 3.58038 2.97059 5

7 15 3 0.10196 1.99180 1

-8 -15 -3 4.84928 3.35643 5

7 -16 -3 4.96384 2.69774 3

7 -16 -3 2.21261 2.32624 2

-7 16 3 -0.6144 3.96164 1

6 -16 -3 4.46378 2.27544 2

6 -16 -3 3.53436 2.33105 3

-6 16 3 -0.5346 3.79668 1

5 -16 -3 0.19768 2.06505 2

5 -16 -3 0.40536 2.09549 3

-5 16 3 -0.6210 3.40019 1

4 -16 -3 19.8377 2.53305 2

-4 16 3 19.0985 3.72058 1

3 -16 -3 21.0129 2.41285 2

-3 16 3 22.4250 3.52270 1

2 -16 -3 -0.1550 1.72971 2

2 -16 -3 -0.8579 1.43282 5

-2 16 3 2.16560 3.23919 1

1 -16 -3 2.17496 1.64839 5

1 -16 -3 -1.1512 1.73168 2

-1 16 3 -2.4510 2.50981 1

0 -16 -3 23.3981 3.05851 2

0 -16 -3 21.4946 2.80899 5

0 16 3 20.7854 2.93318 1

-1 -16 -3 0.61493 1.72943 2

-1 -16 -3 3.59905 1.87120 5

1 16 3 -3.7233 2.34164 1

-2 -16 -3 13.1615 2.11643 2

-2 -16 -3 12.9696 2.18193 5

2 16 3 8.75454 2.61663 1

-3 -16 -3 6.93680 2.20473 5

-3 -16 -3 5.46184 2.13770 2

3 16 3 4.76504 2.43210 1

-4 -16 -3 2.86215 2.29041 5

4 16 3 2.46929 2.22202 1

-5 -16 -3 15.1807 2.88238 5

5 16 3 11.5011 2.48934 1

-6 -16 -3 13.0968 3.07559 5

6 16 3 11.5773 3.77319 1

-7 -16 -3 4.36184 3.21046 5

6 -17 -3 4.89117 2.52525 3

6 -17 -3 5.32547 2.65945 2

-6 17 3 2.31783 4.09110 1

5 -17 -3 5.43906 2.48629 2

-5 17 3 -0.6617 3.73307 1

4 -17 -3 3.60436 2.18747 2

-4 17 3 5.76177 3.59922 1

3 -17 -3 0.39421 2.09033 2

-3 17 3 -5.6959 3.10274 1

2 -17 -3 6.73625 2.21393 5

2 -17 -3 6.49784 2.27400 2

-2 17 3 4.54070 3.17588 1

1 -17 -3 1.48020 2.05502 2

1 -17 -3 2.45431 1.79022 5

-1 17 3 2.78388 2.84732 1

0 -17 -3 5.75427 1.91015 5

0 -17 -3 4.68436 2.02525 2

0 17 3 2.69119 2.83631 1

-1 -17 -3 2.47429 1.95859 2

-1 -17 -3 0.97389 1.94213 5

1 17 3 1.40815 2.91826 1

-2 -17 -3 1.32819 2.09883 5

-2 -17 -3 -1.1728 2.00013 2

2 17 3 -2.2158 3.06476 1

-3 -17 -3 11.3111 4.66270 2

-3 -17 -3 5.62471 2.49840 5

3 17 3 5.06133 3.08268 1

-4 -17 -3 13.6460 2.79343 5

4 17 3 7.99564 2.67253 1

-5 -17 -3 0.76027 2.83540 5

5 17 3 -1.5282 2.37229 1

4 -18 -3 4.06155 2.87069 2

-4 18 3 -4.1705 3.87117 1

3 -18 -3 7.44669 2.61226 2

-3 18 3 2.00577 3.62341 1

2 -18 -3 5.68788 2.55134 2

-2 18 3 1.00880 3.44095 1

1 -18 -3 1.32695 2.37967 2

-1 18 3 7.72738 3.31180 1

0 -18 -3 2.67825 2.02336 5

0 -18 -3 1.17911 2.69813 2

0 18 3 1.55535 3.11431 1

-1 -18 -3 1.02750 2.15855 5

-1 -18 -3 0.08022 2.75613 2

1 18 3 -0.5227 2.98665 1

-2 -18 -3 5.50315 2.54682 2

2 18 3 -3.2346 2.84404 1

-3 -18 -3 6.52857 2.61727 5

3 18 3 -1.2687 2.91709 1

-4 -18 -3 4.81437 2.90029 5

4 18 3 3.52092 2.78115 1

-4 -18 4 1.41205 2.73192 2

-3 -18 4 -1.3091 2.76486 2

-2 -18 4 18.3176 3.12308 2

1 18 -4 -0.8508 2.61466 1

-1 -18 4 5.08243 2.72057 2

0 18 -4 2.27739 2.82934 1

0 -18 4 2.95511 2.65495 2

-1 18 -4 4.78610 2.94210 1

1 -18 4 0.19990 2.62084 2

-2 18 -4 9.00260 3.22925 1

2 -18 4 9.57362 3.04560 2

-3 18 -4 0.02774 3.32195 1

3 -18 4 3.68371 2.75519 2

-6 -17 4 4.20971 2.65759 2

-6 -17 4 -2.3408 2.48565 5

-5 -17 4 -0.8382 2.50540 2

-4 -17 4 8.66520 2.23562 5

-4 -17 4 9.01768 2.76071 2

-3 -17 4 -1.5655 1.82612 5

-3 -17 4 -0.9787 2.26365 2

2 17 -4 -2.3301 2.17491 1

-2 -17 4 -0.2282 2.28760 2

1 17 -4 4.52868 2.38100 1

-1 -17 4 -1.4171 2.30933 2

0 17 -4 19.7728 2.91844 1

0 -17 4 19.7076 2.85214 2

-1 17 -4 0.83218 2.60041 1

1 -17 4 -0.8303 2.46617 2

-2 17 -4 5.37609 2.87510 1

2 -17 4 4.12088 2.58098 2

-3 17 -4 10.6416 3.10031 1

3 -17 4 6.65822 2.59489 2

-4 17 -4 -2.2139 3.08428 1

4 -17 4 2.03919 2.67363 2

-5 17 -4 2.32466 3.32157 1

5 -17 4 -0.6004 2.73994 2

-7 -16 4 -1.4319 2.63508 5

-6 -16 4 5.20969 2.93124 5

-6 -16 4 6.63557 2.76667 2

-5 -16 4 8.73945 2.42430 2

-5 -16 4 9.70133 2.38023 5

-4 -16 4 -1.6387 1.76817 5

-4 -16 4 0.05553 1.99804 2

-3 -16 4 1.78608 1.58037 5

-3 -16 4 1.29983 3.75110 2

2 16 -4 3.36806 1.93863 1

-2 -16 4 0.53960 1.98424 2

-2 -16 4 1.48888 1.36819 5

1 16 -4 -0.8506 2.02151 1

-1 -16 4 -1.4972 2.01913 2

0 16 -4 1.54030 2.18833 1

0 -16 4 0.45086 2.01912 2

-1 16 -4 6.48207 2.58954 1

1 -16 4 5.07696 2.36176 2

-2 16 -4 2.50944 2.47261 1

2 -16 4 0.40534 2.09064 2

-3 16 -4 8.21260 2.80774 1

3 -16 4 6.26228 2.38703 2

-4 16 -4 -0.0001 2.81036 1

4 -16 4 -1.2653 2.89703 2

-5 16 -4 7.75707 3.11323 1

5 -16 4 8.30290 2.52415 2

-6 16 -4 4.88611 3.29069 1

6 -16 4 3.53747 2.47148 2

-7 16 -4 6.20755 3.47466 1

7 -16 4 1.19298 2.54377 2

-8 -15 4 7.27574 2.91434 5

-7 -15 4 0.84683 2.57205 5

-6 -15 4 -2.4283 2.17978 2

-6 -15 4 -1.2342 2.16943 5

-5 -15 4 2.85717 1.90008 5

-5 -15 4 -0.8274 2.23938 2

-4 -15 4 1.76865 1.85924 2

-4 -15 4 3.91290 1.74023 5

3 15 -4 6.92513 1.69719 1

-3 -15 4 8.24462 1.67892 5

-3 -15 4 7.60194 2.16806 2

2 15 -4 0.58693 1.76322 1

-2 -15 4 -0.4624 1.84964 2

-2 -15 4 2.01328 1.41529 5

1 15 -4 22.6615 3.20346 1

-1 -15 4 20.0125 2.40090 2

0 15 -4 3.02372 2.06713 1

0 -15 4 3.93350 2.05858 2

-1 15 -4 0.29814 1.99798 1

1 -15 4 0.87438 1.88086 2

-2 15 -4 10.6234 2.47284 1

2 -15 4 11.6466 2.31375 2

-3 15 -4 0.43439 2.24266 1

3 -15 4 -0.1148 1.92643 2

4 -15 4 25.4013 3.67694 2

-5 15 -4 14.0206 2.99870 1

5 -15 4 10.0860 2.34222 2

-6 15 -4 8.62120 2.97693 1

6 -15 4 9.51070 1.80522 3

6 -15 4 6.39764 2.53710 2

-7 15 -4 0.09514 2.97252 1

7 -15 4 2.40426 2.48541 2

-8 15 -4 9.32015 3.50258 1

8 -15 4 3.13797 2.66376 2

9 14 -4 2.42608 2.33430 3

-9 -14 4 4.67683 3.06402 5

-8 -14 4 0.57916 2.55018 5

-7 -14 4 13.3725 2.65591 5

-6 -14 4 4.67159 2.77477 2

-6 -14 4 4.84537 2.14381 5

-5 -14 4 0.33912 1.88071 2

-5 -14 4 2.11617 1.82231 5

-4 -14 4 6.10207 1.76711 5

-4 -14 4 3.83754 1.89046 2

-3 -14 4 1.82021 1.48184 5

-3 -14 4 2.20078 1.62955 2

2 14 -4 20.5743 2.69514 1

-2 -14 4 22.1747 2.96182 2

-2 -14 4 23.6339 2.26340 5

1 14 -4 47.9169 3.45645 1

-1 -14 4 52.4323 2.66754 5

-1 -14 4 53.4818 3.53928 2

0 14 -4 10.6803 1.93864 1

0 -14 4 11.3745 2.00981 2

-1 14 -4 8.60178 2.00865 1

1 -14 4 7.36193 1.77257 2

-2 14 -4 -1.0662 1.79618 1

2 -14 4 0.35924 1.70720 2

-3 14 -4 -2.5207 1.94333 1

3 -14 4 0.48596 1.82686 2

-4 14 -4 2.24038 2.19172 1

4 -14 4 2.29391 1.92449 2

-5 14 -4 14.0094 2.67792 1

5 -14 4 15.2956 2.38465 2

5 -14 4 21.4119 2.85490 3

-6 14 -4 22.2880 3.09971 1

6 -14 4 19.1364 2.66808 2

6 -14 4 21.9828 3.15562 3

-7 14 -4 10.3289 3.03833 1

7 -14 4 12.0269 2.10518 3

7 -14 4 6.92039 2.38947 2

-8 14 -4 4.53477 3.11465 1

8 -14 4 5.48765 2.12489 3

10 13 -4 -2.6256 2.65231 3

-10 -13 4 3.03230 3.33612 5

9 13 -4 5.18862 2.18267 3

-9 -13 4 3.50449 2.73907 5

-8 -13 4 0.13469 3.96038 5

-7 -13 4 8.92471 2.33153 5

-6 -13 4 21.4570 3.17146 2

-6 -13 4 21.0961 3.15419 5

-5 -13 4 -2.0355 1.63100 2

-5 -13 4 -0.9043 1.54250 5

-4 -13 4 12.1730 1.85302 2

-4 -13 4 13.0051 1.86944 5

-3 -13 4 5.53030 1.66151 2

-3 -13 4 6.80100 1.53124 5

2 13 -4 6.26163 1.46191 1

-2 -13 4 8.22197 1.34790 5

-2 -13 4 4.86077 1.65491 2

1 13 -4 3.65060 1.51723 1

-1 -13 4 4.33662 1.13956 5

-1 -13 4 1.73696 1.46738 2

0 13 -4 38.2876 3.01128 1

0 -13 4 34.2563 2.98009 2

-1 13 -4 28.5327 2.97972 1

1 -13 4 28.0578 2.86410 2

-2 13 -4 10.5708 1.92029 1

2 -13 4 12.4026 1.84910 2

-3 13 -4 50.8657 3.70707 1

-3 13 -4 52.8615 3.81628 4

3 -13 4 45.2369 3.32854 2

-4 13 -4 5.33711 2.80281 4

-4 13 -4 2.12549 1.81440 1

4 -13 4 4.46237 1.18970 3

4 -13 4 3.07728 1.61617 2

-5 13 -4 3.71333 2.10374 1

-5 13 -4 8.24548 3.21008 4

5 -13 4 5.41731 1.97937 2

5 -13 4 4.29181 1.37402 3

-6 13 -4 7.17116 2.48924 1

6 -13 4 8.35434 1.67187 3

-7 13 -4 13.2859 2.85936 1

7 -13 4 7.77652 1.94150 3

-8 13 -4 0.40996 2.81531 1

8 -13 4 1.17526 1.98899 3

8 -13 4 -1.9706 2.89422 1

-9 13 -4 3.56666 3.18217 1

9 -13 4 2.81627 2.28899 3

9 -13 4 -0.2454 3.31126 1

10 12 -4 -1.3137 2.23330 3

-10 -12 4 1.73254 2.90634 5

9 12 -4 4.20061 2.13323 3

-9 -12 4 4.70781 2.64842 5

-8 -12 4 32.0465 3.87411 5

-7 -12 4 36.4278 3.57462 5

-6 -12 4 24.6050 2.89911 5

-6 -12 4 26.4166 2.92878 2

-5 -12 4 29.4551 2.83809 5

-5 -12 4 30.9885 2.84550 2

-4 -12 4 76.8082 3.78818 2

-4 -12 4 78.5385 3.64994 5

-3 -12 4 2.55032 1.32063 5

-3 -12 4 2.20902 1.30302 2

2 12 -4 7.26854 1.34214 1

-2 -12 4 7.97680 1.68445 2

-2 -12 4 8.89123 1.30549 5

1 12 -4 23.6516 2.36022 1

1 12 -4 20.7792 1.84081 1

-1 -12 4 19.0096 1.74037 5

-1 -12 4 24.6029 2.49808 2

0 12 -4 -2.9949 1.29530 1

0 12 -4 -1.4389 1.09655 1

0 -12 4 -0.4217 1.40284 2

-1 12 -4 165.296 6.15641 1

-1 12 -4 181.483 6.45306 1

1 -12 4 169.573 6.43222 2

-2 12 -4 21.5474 2.66678 1

2 -12 4 20.1462 2.54277 2

-3 12 -4 60.2076 3.88626 4

-3 12 -4 63.4044 3.76621 1

3 -12 4 54.2014 3.36193 2

3 -12 4 56.2121 2.99002 3

-4 12 -4 47.7530 3.86906 4

-4 12 -4 46.5366 3.64019 1

4 -12 4 43.8995 2.86515 3

4 -12 4 41.5930 3.18252 2

-5 12 -4 45.2086 3.70999 1

-5 12 -4 42.6916 4.03738 4

5 -12 4 45.8573 3.12281 3

5 -12 4 35.2732 3.71680 1

-6 12 -4 5.75953 3.15144 4

-6 12 -4 3.09466 2.11044 1

6 -12 4 3.01660 1.47639 3

6 -12 4 1.99462 2.06362 1

-7 12 -4 10.7937 2.56618 1

7 -12 4 7.57081 2.38991 1

7 -12 4 7.03341 1.80261 3

-8 12 -4 6.95378 2.82457 1

8 -12 4 1.42231 2.54398 1

8 -12 4 2.71997 2.04505 3

-9 12 -4 1.68167 2.98580 1

9 -12 4 1.20537 2.19792 3

9 -12 4 -3.1441 3.81062 1

-10 12 -4 4.14392 3.37332 1

10 -12 4 4.48528 3.42379 1

10 -12 4 -2.0922 2.74565 3

11 11 -4 -3.6242 2.33673 3

-11 -11 4 0.47194 3.24194 5

10 11 -4 8.29549 2.31672 3

-10 -11 4 11.8472 3.17015 5

9 11 -4 12.2348 2.04347 3

-9 -11 4 23.9552 4.11661 5

-8 -11 4 54.4681 4.02226 5

-7 -11 4 5.46775 2.00629 5

-6 -11 4 83.7988 4.20135 2

-6 -11 4 96.3083 4.24432 5

-5 -11 4 10.8599 1.59353 2

-5 -11 4 12.4497 1.77977 5

4 11 -4 53.3663 2.41137 6

-4 -11 4 48.6761 3.06015 2

-4 -11 4 55.4927 2.88577 5

-3 -11 4 83.8268 3.79020 2

-3 -11 4 85.6042 3.52803 5

2 11 -4 155.050 5.52982 1

2 11 -4 153.612 5.33456 1

-2 -11 4 161.390 5.49690 5

-2 -11 4 159.109 5.88011 2

1 11 -4 169.716 6.03007 1

1 11 -4 177.776 6.21508 1

-1 -11 4 181.260 6.42094 2

-1 -11 4 181.967 6.20524 5

0 11 -4 11.6497 1.45298 1

0 11 -4 12.7472 1.81225 3

0 11 -4 11.7908 1.62025 1

0 -11 4 16.5558 2.19738 2

-1 11 -4 18.3114 2.13705 1

-1 11 -4 18.8617 1.86282 1

-1 11 -4 12.8247 1.62693 3

1 -11 4 16.9178 2.09990 2

-2 11 -4 5.64917 1.29314 1

-2 11 -4 7.96405 0.98455 3

-2 11 -4 13.2879 2.34160 4

-2 11 -4 7.16899 1.51746 1

2 -11 4 9.52483 1.50728 2

-3 11 -4 79.4809 3.87125 1

-3 11 -4 73.3139 4.09452 4

3 -11 4 76.2214 3.35155 3

3 -11 4 77.0404 3.70384 2

-4 11 -4 33.8012 3.42576 4

4 -11 4 30.8225 3.08327 1

4 -11 4 32.8772 2.64542 3

-5 11 -4 28.5590 3.62948 4

5 -11 4 34.7443 3.28022 1

5 -11 4 30.3711 2.79528 3

-6 11 -4 5.13435 3.03881 4

6 -11 4 4.17179 1.88296 1

6 -11 4 2.60723 1.46034 3

7 -11 4 12.1063 2.33089 1

7 -11 4 10.7981 1.87867 3

8 -11 4 0.94947 2.25050 1

8 -11 4 -1.2075 1.91235 3

8 -11 4 -1.4895 1.60940 6

-9 11 -4 3.39879 2.74180 1

9 -11 4 -0.8976 2.32175 3

9 -11 4 3.78118 2.71668 1

-10 11 -4 1.18209 3.10160 1

10 -11 4 -0.9499 2.65235 3

10 -11 4 0.99416 3.01400 1

12 10 -4 -3.4798 2.47733 3

-12 -10 4 1.10860 3.57805 5

11 10 -4 -0.5431 2.28051 3

-11 -10 4 -0.9240 2.99300 5

10 10 -4 0.35809 1.92155 3

-10 -10 4 1.90447 2.56257 5

9 10 -4 1.41130 1.50303 3

-9 -10 4 4.53169 2.17233 5

-8 -10 4 0.02148 1.87790 5

-7 -10 4 3.86755 1.77341 5

-6 -10 4 99.3650 4.33079 2

-6 -10 4 95.0431 4.33747 5

-5 -10 4 33.1118 2.48001 2

-5 -10 4 36.6327 2.47521 5

4 10 -4 52.1178 2.36066 6

-4 -10 4 54.5517 2.89100 2

-4 -10 4 52.4443 2.73222 5

3 10 -4 72.8390 2.70831 6

-3 -10 4 75.8362 3.10436 5

-3 -10 4 70.0444 3.29742 2

2 10 -4 28.4474 1.92302 1

2 10 -4 26.4202 1.85004 3

2 10 -4 25.3438 1.54200 1

-2 -10 4 25.3973 2.23405 2

-2 -10 4 25.1085 1.77216 5

1 10 -4 12.4000 1.75031 3

1 10 -4 11.2156 1.23958 1

-1 -10 4 11.3978 1.49112 2

-1 -10 4 11.0575 1.39994 5

0 10 -4 90.6225 3.57434 1

0 10 -4 84.4874 3.45911 3

0 10 -4 89.5278 3.38352 1

0 -10 4 88.4965 3.74230 2

-1 10 -4 22.8115 2.43340 4

-1 10 -4 17.0074 1.74681 1

-1 10 -4 17.9078 1.59254 3

-1 10 -4 17.5922 2.18703 1

1 -10 4 18.0741 2.08546 2

-2 10 -4 121.645 4.32841 3

-2 10 -4 117.796 4.59770 1

-2 10 -4 121.649 5.33864 4

2 -10 4 125.123 4.77527 2

2 -10 4 124.892 4.45087 3

2 -10 4 122.296 4.77904 1

-3 10 -4 -1.0871 2.00013 4

-3 10 -4 -0.1026 0.63652 3

3 -10 4 -0.5224 0.84050 3

3 -10 4 -0.6453 1.25463 1

-4 10 -4 125.992 5.44060 4

4 -10 4 124.864 5.16452 1

4 -10 4 125.604 4.83134 3

-5 10 -4 -0.3272 2.56499 4

5 -10 4 4.27199 1.30529 3

5 -10 4 1.08470 1.43906 1

-6 10 -4 60.2934 4.52105 4

6 -10 4 67.1673 3.89189 1

6 -10 4 62.5204 3.63104 3

-7 10 -4 6.95088 3.18081 4

7 -10 4 3.37822 1.38149 6

7 -10 4 2.92223 1.82546 1

7 -10 4 1.61292 1.63197 3

8 -10 4 0.31841 1.88101 3

8 -10 4 1.12089 1.88993 1

8 -10 4 1.54004 1.42494 6

9 -10 4 3.15147 2.27885 3

9 -10 4 7.32900 2.42899 1

9 -10 4 4.74340 1.75888 6

10 -10 4 0.77569 1.98334 6

10 -10 4 2.71650 2.55474 1

-11 10 -4 1.11741 3.27935 1

11 -10 4 1.94632 3.08721 1

-12 -9 4 -3.7574 3.23937 5

11 9 -4 4.32747 1.99364 3

-11 -9 4 0.04983 2.87518 5

10 9 -4 0.46539 1.58861 3

-10 -9 4 2.69775 4.54401 5

9 9 -4 10.0891 1.75498 3

-9 -9 4 12.9620 2.31387 5

-8 -9 4 5.55158 1.85775 5

-7 -9 4 31.5915 2.94119 5

-6 -9 4 421.798 13.7788 2

-6 -9 4 413.960 13.7795 5

-5 -9 4 315.360 10.3951 5

-5 -9 4 305.002 10.3730 2

-4 -9 4 120.554 4.47269 5

-4 -9 4 116.476 4.53344 2

3 9 -4 115.250 3.94801 6

3 9 -4 114.844 4.14571 3

-3 -9 4 116.152 4.39807 2

-3 -9 4 112.620 4.23427 5

2 9 -4 183.459 6.76012 1

2 9 -4 204.128 6.56866 1

2 9 -4 208.769 6.77308 3

2 9 -4 199.815 6.73293 5

-2 -9 4 207.476 6.94083 5

-2 -9 4 208.031 6.98884 2

1 9 -4 94.9692 3.30718 1

1 9 -4 89.3636 3.41660 1

1 9 -4 90.9623 3.45107 3

-1 -9 4 91.3627 3.32637 5

-1 -9 4 90.4890 3.68842 2

0 9 -4 354.804 11.6101 3

0 9 -4 364.711 11.7302 1

0 9 -4 367.424 11.6488 1

0 -9 4 360.761 11.8519 2

-1 9 -4 10.6068 1.32370 3

-1 9 -4 15.9989 1.89919 4

-1 9 -4 14.2114 1.51424 1

1 -9 4 15.1503 1.60486 2

1 -9 4 19.6736 2.34413 4

-2 9 -4 505.302 16.1023 3

-2 9 -4 526.965 16.3577 1

-2 9 -4 516.337 16.8729 4

2 -9 4 523.247 16.2259 3

2 -9 4 517.674 16.4502 2

2 -9 4 506.118 16.4334 1

2 -9 4 465.172 16.5422 4

-3 9 -4 62.9845 2.58729 3

-3 9 -4 67.9704 3.50798 4

3 -9 4 64.6464 3.55357 1

3 -9 4 64.4399 2.95759 3

-4 9 -4 234.397 8.38647 4

4 -9 4 226.897 8.14180 1

-5 9 -4 10.1255 2.48686 4

5 -9 4 9.11032 1.51640 1

5 -9 4 10.9853 1.28164 6

5 -9 4 7.63254 1.37935 3

-6 9 -4 105.375 5.28546 4

6 -9 4 97.0937 4.66914 3

6 -9 4 107.753 4.75185 1

-7 9 -4 -3.0926 2.69857 4

7 -9 4 -1.0961 1.44368 3

7 -9 4 0.49505 1.51771 1

8 -9 4 12.2336 2.10071 3

8 -9 4 15.5038 2.23017 1

9 -9 4 2.09581 1.92673 1

9 -9 4 -3.0763 2.06790 3

10 -9 4 -4.7782 2.43538 3

11 -9 4 -2.6856 3.21701 3

11 -9 4 2.00314 2.67083 1

12 8 -4 1.13869 2.14476 3

-12 -8 4 -3.0935 3.03414 5

11 8 -4 1.29954 2.63222 3

-11 -8 4 2.24065 2.65159 5

10 8 -4 6.80334 1.71654 3

-10 -8 4 7.31301 2.35332 5

9 8 -4 -0.1435 1.32597 3

-9 -8 4 -1.2716 1.81062 5

8 8 -4 5.13619 1.28393 3

-8 -8 4 8.51851 1.79712 5

-7 -8 4 74.9976 3.69572 5

-6 -8 4 8.92610 1.40532 2

-6 -8 4 12.6236 1.64354 5

-5 -8 4 -0.7432 1.14874 5

-5 -8 4 -0.3748 0.98349 2

-4 -8 4 40.1014 2.25100 5

-4 -8 4 38.8459 2.30432 2

-3 -8 4 416.556 13.9717 5

-3 -8 4 448.662 14.0011 2

2 8 -4 94.3431 3.31989 3

2 8 -4 87.5337 3.28239 5

-2 -8 4 91.4471 3.55900 2

-2 -8 4 88.7972 3.33933 5

1 8 -4 264.479 8.39408 1

1 8 -4 264.874 8.45243 3

1 8 -4 255.634 8.37833 5

-1 -8 4 260.980 8.64508 2

-1 -8 4 256.532 8.39873 5

0 8 -4 384.564 12.0332 1

0 8 -4 365.512 12.1755 4

0 8 -4 378.947 11.9786 3

0 -8 4 373.474 12.1923 2

-1 8 -4 83.8575 2.97957 3

-1 8 -4 88.6993 3.20401 1

-1 8 -4 76.9852 3.51441 4

1 -8 4 83.9439 3.23943 1

1 -8 4 79.3577 3.71475 4

1 -8 4 83.9095 3.37773 2

-2 8 -4 12.7007 1.14833 3

-2 8 -4 11.5292 1.52761 1

2 -8 4 11.7281 1.49324 3

2 -8 4 17.0796 1.74081 1

2 -8 4 13.6388 2.31535 4

-3 8 -4 453.730 14.0189 1

-3 8 -4 439.308 14.2621 4

-3 8 -4 422.010 13.6987 3

3 -8 4 415.349 14.0293 1

3 -8 4 425.307 13.8550 3

-4 8 -4 328.378 10.8897 3

-4 8 -4 351.471 11.5352 4

4 -8 4 337.270 11.2493 1

4 -8 4 330.890 10.7555 6

-5 8 -4 5.94835 2.27459 4

5 -8 4 7.03066 1.27536 3

5 -8 4 8.90503 1.06646 6

5 -8 4 6.72140 1.27897 1

-6 8 -4 -2.8703 2.35348 4

6 -8 4 4.17887 1.21320 6

6 -8 4 0.37834 1.17070 1

6 -8 4 0.74239 1.20973 3

-7 8 -4 1.27725 2.82978 4

7 -8 4 5.09739 1.52374 1

7 -8 4 1.66471 1.42181 3

-8 8 -4 0.72410 3.01420 4

8 -8 4 -0.2093 1.55405 1

8 -8 4 0.13289 1.62585 3

9 -8 4 1.56777 1.72098 1

9 -8 4 2.52174 1.97781 3

10 -8 4 1.80967 1.93113 1

10 -8 4 -2.7322 2.10289 3

11 -8 4 -0.7393 2.30045 1

11 -8 4 0.12633 3.31438 3

-12 8 -4 5.03511 3.38108 1

12 -8 4 2.14306 2.79816 1

13 7 -4 -1.0951 3.11877 3

-13 -7 4 8.77662 3.77260 5

12 7 -4 7.58376 2.30542 3

12 7 -4 5.71464 2.03817 1

-12 -7 4 17.4845 3.34566 5

11 7 -4 -2.7769 1.65952 3

-11 -7 4 -0.8519 2.44456 5

10 7 -4 2.40077 1.45436 3

-10 -7 4 2.51619 2.21391 5

9 7 -4 4.60000 1.38838 3

-9 -7 4 6.80519 1.84461 5

8 7 -4 17.0204 2.35155 3

-8 -7 4 16.8174 2.46965 5

-7 -7 4 88.5289 3.94110 5

-6 -7 4 90.8660 3.74359 2

-6 -7 4 91.4288 3.85180 5

-5 -7 4 60.7385 2.83570 2

-5 -7 4 63.8987 2.90056 5

-4 -7 4 12.8897 1.71269 5

-4 -7 4 13.8755 1.72068 2

3 7 -4 912.837 28.0324 5

3 7 -4 887.432 28.1258 4

-3 -7 4 881.878 28.1178 5

-3 -7 4 885.794 28.1300 2

2 7 -4 270.601 8.22577 1

2 7 -4 256.033 8.34969 5

-2 -7 4 251.508 8.40862 5

-2 -7 4 257.322 8.53311 2

1 7 -4 452.978 14.1000 3

1 7 -4 473.733 14.0877 1

1 7 -4 434.759 14.0505 5

-1 -7 4 436.731 14.2939 2

-1 -7 4 430.600 14.0832 5

0 7 -4 67.4625 2.49857 3

0 7 -4 60.3108 2.82224 4

0 7 -4 68.8477 2.55588 1

0 -7 4 68.1075 2.54270 1

0 -7 4 68.6094 3.05282 4

-1 7 -4 634.258 18.8577 1

-1 7 -4 599.746 18.6591 3

-1 7 -4 600.615 18.9871 4

1 -7 4 574.183 18.8247 1

1 -7 4 571.011 18.9136 2

1 -7 4 581.951 18.6923 3

1 -7 4 599.340 19.0478 4

-2 7 -4 53.0339 2.80664 4

-2 7 -4 57.6577 2.10434 3

-2 7 -4 58.1875 2.46942 1

2 -7 4 52.2720 2.47383 1

2 -7 4 55.2808 2.33853 3

2 -7 4 58.4172 2.98317 4

-3 7 -4 351.895 11.2074 1

-3 7 -4 344.197 10.9355 3

3 -7 4 335.136 11.0754 3

3 -7 4 336.673 11.2399 1

-4 7 -4 97.7336 4.48751 4

-4 7 -4 106.557 3.71913 3

4 -7 4 99.4129 3.58578 6

4 -7 4 104.991 3.96520 3

-5 7 -4 55.2084 3.52211 4

5 -7 4 57.4516 2.87808 1

5 -7 4 52.3981 2.38317 6

5 -7 4 53.3211 3.07341 3

-6 7 -4 16.5856 2.95381 4

6 -7 4 28.2052 2.20299 1

6 -7 4 21.1942 2.29820 3

-7 7 -4 24.3833 3.58923 4

7 -7 4 27.0014 2.74282 3

7 -7 4 25.1555 2.59132 1

-8 7 -4 -3.7578 2.86206 4

8 -7 4 2.32610 1.36960 1

8 -7 4 -2.4163 1.32421 3

9 -7 4 4.27234 1.78602 1

9 -7 4 2.24879 1.71908 3

10 -7 4 -0.4973 2.08733 3

10 -7 4 2.14844 1.85795 1

11 -7 4 0.57623 2.39689 3

11 -7 4 0.42218 2.18313 1

12 -7 4 3.64835 2.64592 1

13 6 -4 -2.6700 2.62066 3

12 6 -4 0.95396 1.95416 1

12 6 -4 1.24774 1.95626 3

-12 -6 4 2.21210 2.98127 5

11 6 -4 4.30738 1.60111 1

11 6 -4 4.40691 1.75984 3

-11 -6 4 5.55090 2.40474 5

10 6 -4 -0.7336 1.20023 1

10 6 -4 -0.1610 1.37061 3

-10 -6 4 0.10146 1.79066 5

9 6 -4 0.92573 1.19892 3

9 6 -4 0.74093 1.01798 1

-9 -6 4 0.58823 1.47910 5

8 6 -4 0.91697 0.95914 3

-8 -6 4 1.27563 1.39333 5

7 6 -4 4.16738 0.98984 3

-7 -6 4 5.73145 1.33954 5

-6 -6 4 2.08495 1.25062 5

-5 -6 4 342.061 11.2317 2

-5 -6 4 346.370 11.3097 5

-4 -6 4 144.001 4.95736 2

-4 -6 4 138.789 4.98692 5

3 6 -4 41.8721 1.84131 5

-3 -6 4 41.8023 1.95296 5

-3 -6 4 37.0559 1.94713 2

2 6 -4 1.11556 0.72743 2

2 6 -4 1.05635 1.12861 4

2 6 -4 1.52108 0.59473 5

-2 -6 4 0.54884 0.65902 5

-2 -6 4 -0.5836 0.74528 2

1 6 -4 47.2853 2.28282 4

1 6 -4 48.5387 1.81291 5

1 6 -4 47.1707 1.83839 3

1 6 -4 51.9655 1.84394 1

-1 -6 4 44.3801 2.10949 2

-1 -6 4 50.9453 1.89953 5

0 6 -4 473.587 15.7115 4

0 6 -4 498.751 15.4780 3

0 -6 4 514.313 15.8116 4

0 -6 4 484.730 15.5402 1

-1 6 -4 797.147 24.9056 3

-1 6 -4 841.410 25.1094 1

-1 6 -4 796.608 25.2276 4

1 -6 4 781.080 25.0801 1

1 -6 4 792.927 25.2657 4

1 -6 4 777.968 24.9380 3

-2 6 -4 20.3351 1.85450 4

-2 6 -4 21.2483 1.09855 3

-2 6 -4 21.0226 1.47181 1

2 -6 4 17.3673 1.33890 3

2 -6 4 23.7938 2.02653 4

2 -6 4 20.0595 1.45475 1

-3 6 -4 214.061 6.81740 1

-3 6 -4 206.987 6.53972 3

-3 6 -4 198.253 7.07788 4

3 -6 4 189.988 7.08970 4

3 -6 4 196.160 6.47591 6

3 -6 4 196.135 6.64067 3

3 -6 4 197.133 6.77965 1

-4 6 -4 150.636 5.78206 4

-4 6 -4 155.785 5.13317 3

4 -6 4 144.963 5.22757 3

4 -6 4 146.643 5.05478 6

-5 6 -4 108.216 4.86484 4

5 -6 4 109.415 4.07410 6

5 -6 4 116.163 4.38583 1

-6 6 -4 46.9764 3.53637 4

6 -6 4 56.6087 2.89746 1

6 -6 4 55.9826 2.81277 3

-7 6 -4 23.2840 3.30070 4

7 -6 4 22.7021 2.39309 3

7 -6 4 23.7333 2.38168 1

-8 6 -4 27.1489 4.04136 4

8 -6 4 37.6185 2.96146 1

8 -6 4 37.5014 3.15276 3

9 -6 4 7.55936 1.71181 3

9 -6 4 5.19717 1.59855 1

10 -6 4 11.7599 2.08400 1

10 -6 4 12.6626 2.22766 3

11 -6 4 -2.5123 2.14591 3

11 -6 4 -0.1433 1.88428 1

12 -6 4 -0.0141 2.95025 3

12 -6 4 1.85729 2.47066 1

13 5 -4 -0.8017 2.49330 1

13 5 -4 0.07175 2.55639 3

12 5 -4 -0.1722 1.86991 1

12 5 -4 -1.5369 2.15182 3

-12 -5 4 -2.1407 2.50911 5

11 5 -4 -0.1581 1.44049 3

11 5 -4 -1.5476 1.52633 1

-11 -5 4 -0.9547 2.07967 5

10 5 -4 18.0362 2.41778 1

10 5 -4 21.1124 2.78628 3

-10 -5 4 23.1848 2.84405 5

9 5 -4 13.3690 1.91848 1

-9 -5 4 12.0824 1.89661 5

8 5 -4 4.77221 1.17178 3

8 5 -4 5.61267 1.02215 1

-8 -5 4 7.72856 1.56082 5

7 5 -4 15.1643 1.36904 1

7 5 -4 15.4785 1.85746 3

-7 -5 4 14.3937 1.87964 5

-6 -5 4 43.3953 2.39773 5

-5 -5 4 137.286 4.68105 2

-5 -5 4 124.277 4.75954 5

4 5 -4 36.3345 1.77479 5

-4 -5 4 36.0670 1.82544 2

3 5 -4 62.4520 2.39021 5

3 5 -4 66.6614 2.40711 2

-3 -5 4 64.5456 2.65051 2

-3 -5 4 52.8362 2.42973 5

2 5 -4 6.28140 0.91719 2

2 5 -4 5.50730 0.80434 5

2 5 -4 8.50860 1.24610 4

-2 -5 4 7.77529 0.80315 5

-2 -5 4 8.19771 0.82497 2

1 5 -4 12.6681 0.76194 3

1 5 -4 11.6490 0.79165 1

1 5 -4 13.9519 1.36599 4

1 5 -4 10.8899 0.78589 5

-1 -5 4 10.3183 1.04903 2

-1 -5 4 14.0232 0.88146 5

0 5 -4 386.279 12.1533 1

0 -5 4 368.805 12.2190 2

0 -5 4 404.800 12.3952 4

0 -5 4 374.715 12.1194 1

-1 5 -4 1041.00 33.0510 4

-1 5 -4 1071.76 32.9215 1

1 -5 4 1044.62 33.0753 4

1 -5 4 1046.56 33.1316 1

1 -5 4 1053.58 32.8914 3

-2 5 -4 1079.28 33.5630 4

-2 5 -4 1061.03 33.4039 1

-2 5 -4 1112.95 33.2139 3

2 -5 4 1076.94 33.6082 4

2 -5 4 1056.50 33.1922 6

2 -5 4 1054.21 33.4031 1

2 -5 4 1013.43 33.2298 3

-3 5 -4 415.986 13.9653 4

-3 5 -4 433.864 13.3431 3

-3 5 -4 442.297 13.5764 1

3 -5 4 425.394 13.8199 4

3 -5 4 399.494 13.3475 3

3 -5 4 413.267 13.5359 1

3 -5 4 415.389 13.3220 6

-4 5 -4 27.5322 2.34854 4

-4 5 -4 30.6166 1.89598 1

4 -5 4 33.9729 1.76029 3

4 -5 4 32.0242 2.08228 1

4 -5 4 32.1917 1.61964 6

-5 5 -4 169.429 6.56819 4

5 -5 4 163.311 5.87394 6

5 -5 4 172.460 6.09137 1

5 -5 4 181.554 6.10878 3

-6 5 -4 16.6772 2.83013 4

6 -5 4 22.8965 1.87761 3

6 -5 4 20.2957 2.07869 1

-7 5 -4 3.62096 2.40319 4

7 -5 4 3.32746 1.17495 1

7 -5 4 2.53277 0.97215 3

-8 5 -4 18.5858 3.16753 4

8 -5 4 18.3309 2.40180 3

8 -5 4 17.6774 2.51641 1

9 -5 4 -1.0423 1.26986 3

9 -5 4 1.52196 1.43615 1

10 -5 4 5.32936 1.74988 1

10 -5 4 6.03134 1.92762 3

11 -5 4 1.30155 2.14293 3

11 -5 4 5.01252 2.43178 1

12 -5 4 0.11474 2.20634 1

12 -5 4 0.10194 2.73374 3

13 -5 4 -6.1710 2.97321 3

13 -5 4 1.64190 2.64276 1

13 4 -4 -1.1827 2.45667 1

13 4 -4 0.41446 2.77715 3

12 4 -4 0.10276 1.95916 1

12 4 -4 -0.3138 2.18519 3

11 4 -4 1.49158 1.51278 3

11 4 -4 5.04883 1.68455 1

-11 -4 4 3.69925 2.18362 5

10 4 -4 0.20509 1.28864 1

10 4 -4 -0.5560 1.20475 3

-10 -4 4 1.48607 1.70504 5

9 4 -4 24.4466 2.12387 1

9 4 -4 23.0219 2.42215 3

-9 -4 4 20.0282 2.38162 5

8 4 -4 52.8711 2.88930 3

8 4 -4 46.8775 2.54372 1

-8 -4 4 57.6310 2.93046 5

7 4 -4 111.205 3.98173 1

7 4 -4 113.443 4.35922 3

-7 -4 4 112.406 4.43494 5

6 4 -4 1.27771 0.85860 3

-6 -4 4 0.94322 0.99271 5

-5 -4 4 148.138 5.21139 5

4 4 -4 611.903 18.2353 5

4 4 -4 616.145 18.2029 2

-4 -4 4 497.250 18.3910 5

3 4 -4 1182.77 34.6671 2

3 4 -4 1140.89 34.6624 5

-3 -4 4 994.241 34.6780 5

-3 -4 4 1116.35 34.6683 2

2 4 -4 307.605 9.39671 2

2 4 -4 302.524 9.35004 5

-2 -4 4 271.541 9.37629 5

-2 -4 4 293.769 9.40241 2

1 4 -4 177.662 5.61965 1

1 4 -4 178.279 5.65841 5

1 4 -4 171.872 5.90207 4

1 4 -4 178.901 5.76194 2

1 4 -4 174.845 5.65215 6

-1 -4 4 174.797 5.77121 2

-1 -4 4 181.921 5.69166 5

0 4 -4 19.9269 0.97405 1

0 -4 4 19.7575 1.48944 4

0 -4 4 17.8010 0.90855 1

0 -4 4 19.3041 1.09953 2

-1 4 -4 332.320 11.1237 1

-1 4 -4 355.762 11.3000 4

1 -4 4 365.408 11.3435 4

1 -4 4 345.624 11.1321 1

-2 4 -4 1.42742 0.34795 3

-2 4 -4 0.63489 1.14911 4

-2 4 -4 0.88887 0.59867 1

2 -4 4 1.19318 0.52844 1

2 -4 4 1.27608 1.22260 4

2 -4 4 1.13929 0.37615 3

-3 4 -4 0.41333 1.27242 4

-3 4 -4 -0.9783 0.78616 1

3 -4 4 2.82935 1.43631 4

3 -4 4 0.53095 0.69457 1

-4 4 -4 49.6802 2.40547 1

4 -4 4 52.1302 2.09964 3

4 -4 4 50.2222 2.12627 6

-5 4 -4 72.3917 3.54673 4

5 -4 4 69.7069 3.06400 1

5 -4 4 65.8079 2.82301 6

-6 4 -4 253.656 9.19834 4

6 -4 4 255.821 8.72318 1

6 -4 4 261.854 8.56948 3

-7 4 -4 68.2158 4.00232 4

7 -4 4 60.1443 3.10675 1

7 -4 4 66.7320 2.97975 3

-8 4 -4 0.41689 2.37706 4

8 -4 4 -0.5917 0.90246 3

8 -4 4 -0.6236 1.13333 1

9 -4 4 20.8113 2.74247 3

9 -4 4 19.1774 2.66881 1

10 -4 4 10.2351 1.98869 1

10 -4 4 10.1885 1.78205 3

11 -4 4 -1.1971 1.84929 1

11 -4 4 -2.4498 2.05090 3

12 -4 4 0.05875 2.08246 1

12 -4 4 2.67556 2.65447 3

13 -4 4 1.28348 3.13400 3

13 -4 4 2.41714 2.44884 1

13 3 -4 -1.3996 2.49339 1

13 3 -4 1.87672 2.80757 3

12 3 -4 -2.0897 1.90792 1

12 3 -4 0.45301 2.14454 3

11 3 -4 1.50566 1.72104 3

11 3 -4 2.70469 1.64477 1

-11 -3 4 4.32445 2.01204 5

10 3 -4 1.92065 1.15225 3

10 3 -4 2.08180 1.36822 1

-10 -3 4 3.39753 1.75633 5

-10 -3 4 3.09497 1.47387 1

9 3 -4 1.30472 1.00446 3

9 3 -4 -1.2673 1.03600 1

-9 -3 4 0.63068 1.00191 1

-9 -3 4 0.03938 1.30588 5

8 3 -4 53.5031 2.61527 1

8 3 -4 57.4428 2.92930 3

-8 -3 4 48.5188 2.41031 1

-8 -3 4 55.9719 2.84048 5

7 3 -4 0.73385 0.94235 3

7 3 -4 -1.4807 0.73655 1

-7 -3 4 0.55513 1.08166 5

-7 -3 4 -0.3098 0.75561 1

6 3 -4 0.26212 0.79827 3

-6 -3 4 -1.2212 1.01725 5

-6 -3 4 2.05806 0.61350 1

5 3 -4 140.737 4.92610 2

5 3 -4 155.099 5.22366 3

5 3 -4 145.373 4.78556 1

-5 -3 4 139.618 5.08357 5

4 3 -4 89.8309 3.14450 2

4 3 -4 91.8394 3.19181 3

4 3 -4 88.2530 3.20526 5

4 3 -4 90.8169 2.93358 1

-4 -3 4 82.9131 3.14484 2

3 3 -4 626.498 18.6615 3

3 3 -4 598.026 18.6689 5

3 3 -4 623.545 18.6471 2

-3 -3 4 528.184 18.6786 5

-3 -3 4 589.011 18.6424 2

2 3 -4 73.6264 2.49857 2

2 3 -4 71.7459 2.48680 5

-2 -3 4 66.1742 2.51351 5

-2 -3 4 74.1776 2.52765 2

1 3 -4 2683.48 84.9698 5

1 3 -4 2627.43 84.7397 1

1 3 -4 2592.91 85.3779 4

1 3 -4 2854.48 85.2091 2

1 3 -4 2762.19 85.0716 6

-1 -3 4 2802.85 85.1936 5

0 3 -4 989.264 30.5218 1

0 3 -4 957.032 30.6598 4

0 3 -4 951.829 30.4547 6

0 -3 4 987.358 30.4959 4

0 -3 4 961.191 30.5356 1

0 -3 4 984.878 30.4686 6

-1 3 -4 37.2743 1.42396 6

-1 3 -4 41.0567 1.59930 1

1 -3 4 41.4624 1.56646 1

1 -3 4 39.4465 1.97399 4

1 -3 4 38.1984 1.52895 6

-2 3 -4 33.6436 1.49968 1

-2 3 -4 29.1248 1.80973 4

2 -3 4 30.2418 1.31510 6

2 -3 4 30.8612 1.20900 3

2 -3 4 30.5288 1.43262 1

2 -3 4 30.5021 1.86492 4

-3 3 -4 655.925 20.5910 4

-3 3 -4 611.038 20.3854 1

3 -3 4 629.788 20.2048 3

3 -3 4 662.010 20.3105 6

3 -3 4 671.615 20.6386 4

3 -3 4 636.931 20.3736 1

-4 3 -4 63.4628 2.67008 1

-4 3 -4 57.2055 3.26048 4

4 -3 4 58.7245 3.13124 4

4 -3 4 66.0169 2.63200 1

4 -3 4 61.5377 2.38436 3

4 -3 4 64.1214 2.49390 6

-5 3 -4 185.102 6.72550 4

-5 3 -4 187.465 5.93741 5

5 -3 4 184.940 6.33350 1

5 -3 4 199.233 6.02467 5

5 -3 4 179.406 6.25709 6

5 -3 4 163.533 6.35308 3

-6 3 -4 3.34210 0.77954 5

-6 3 -4 2.95200 1.99265 4

6 -3 4 2.85906 0.82292 3

6 -3 4 2.92698 0.91608 5

6 -3 4 4.70250 1.03181 1

-7 3 -4 5.49816 2.39053 4

-7 3 -4 5.44074 0.93520 5

7 -3 4 5.52063 1.13364 5

7 -3 4 3.46934 0.93106 3

7 -3 4 3.34469 1.11559 1

-8 3 -4 83.1704 3.27173 5

-8 3 -4 84.0472 4.79908 4

8 -3 4 81.9417 3.77654 1

8 -3 4 79.8206 3.55171 3

9 -3 4 12.4924 1.76540 1

9 -3 4 14.7486 2.21199 3

10 -3 4 2.93037 1.68766 1

10 -3 4 3.57286 1.42852 3

11 -3 4 -1.1984 1.76877 3

11 -3 4 -1.8834 1.90698 1

12 -3 4 2.43844 2.33649 3

12 -3 4 3.14924 1.94536 1

13 -3 4 -6.1521 2.85379 3

13 -3 4 -0.0951 2.39847 1

14 2 -4 4.43655 3.14166 1

14 2 -4 4.34986 3.43760 3

13 2 -4 0.71767 2.50515 1

13 2 -4 2.55073 2.38986 3

12 2 -4 1.77639 1.96020 1

11 2 -4 13.2743 1.87365 1

11 2 -4 11.6094 1.83527 3

10 2 -4 46.6539 3.24501 3

10 2 -4 46.4242 2.98547 1

-10 -2 4 47.8244 3.06880 5

-10 -2 4 39.6573 2.77698 1

9 2 -4 9.14157 1.40281 1

9 2 -4 15.6487 2.33547 3

-9 -2 4 14.2586 2.13720 5

-9 -2 4 9.63980 1.32513 1

8 2 -4 149.032 5.28113 1

8 2 -4 148.339 5.48764 3

-8 -2 4 144.636 5.40739 5

-8 -2 4 147.104 5.19704 1

7 2 -4 74.3430 3.00298 1

-7 -2 4 71.6593 2.93204 1

-7 -2 4 77.5825 3.22014 5

6 2 -4 59.5410 2.69412 3

-6 -2 4 58.7591 2.33415 1

-6 -2 4 57.7590 2.62055 5

-6 -2 4 57.3509 2.27159 1

5 2 -4 923.319 28.8967 1

5 2 -4 931.627 29.0894 5

5 2 -4 944.337 28.9645 2

-5 -2 4 905.954 28.8175 1

-5 -2 4 912.515 28.8270 1

4 2 -4 2451.09 72.7344 1

4 2 -4 2363.20 72.3394 2

4 2 -4 2325.42 72.6679 5

4 2 -4 2400.14 73.0041 3

-4 -2 4 2090.49 73.2483 5

-4 -2 4 2296.95 72.4131 1

3 2 -4 37.9191 1.45265 2

3 2 -4 36.4827 1.54579 3

3 2 -4 33.5783 1.47439 5

-3 -2 4 34.6976 1.43278 2

2 2 -4 59.5395 2.12635 6

2 2 -4 59.8220 2.07778 5

2 2 -4 59.0123 2.06789 2

2 2 -4 60.3981 2.08121 3

-2 -2 4 57.4864 2.11348 5

-2 -2 4 58.0152 2.08143 2

1 2 -4 2267.05 71.7125 6

1 2 -4 2289.65 71.9065 4

1 2 -4 2324.00 71.6027 2

1 2 -4 2290.22 71.4464 3

1 2 -4 2248.23 71.6309 5

-1 -2 4 2205.59 71.7062 2

-1 -2 4 2402.02 71.7929 6

0 2 -4 1321.70 41.4899 6

0 2 -4 1314.25 41.3955 1

0 2 -4 1321.62 41.8568 4

0 -2 4 1379.36 41.7844 4

0 -2 4 1270.44 41.6281 6

0 -2 4 1325.04 41.4516 1

-1 2 -4 2835.57 89.8946 6

-1 2 -4 2893.24 90.0235 1

-1 2 -4 2865.34 90.4096 4

1 -2 4 2949.26 90.3347 4

1 -2 4 2898.47 89.9516 6

1 -2 4 2806.29 90.0359 1

-2 2 -4 161.931 5.65797 4

-2 2 -4 171.530 5.46972 1

2 -2 4 167.757 5.72006 4

2 -2 4 173.249 5.43685 6

2 -2 4 162.004 5.41032 1

2 -2 4 159.176 5.29572 3

-3 2 -4 295.830 9.70063 5

-3 2 -4 315.382 9.96717 1

-3 2 -4 314.897 10.1743 4

3 -2 4 302.776 9.79828 3

3 -2 4 296.782 9.89933 1

3 -2 4 309.851 9.92550 6

3 -2 4 326.412 10.2416 4

-4 2 -4 54.9404 2.44735 1

-4 2 -4 55.4662 3.91145 4

-4 2 -4 54.3234 2.01308 5

4 -2 4 57.6018 2.18559 3

4 -2 4 55.1172 2.90849 4

4 -2 4 54.8605 2.09313 5

4 -2 4 52.9201 2.28377 1

4 -2 4 55.8968 2.31004 6

-5 2 -4 782.932 24.6191 4

-5 2 -4 772.880 24.0440 5

5 -2 4 797.241 24.1016 5

5 -2 4 759.804 24.3098 1

5 -2 4 724.356 24.3041 6

-6 2 -4 27.2651 2.64371 4

-6 2 -4 30.2738 1.60175 5

6 -2 4 22.7910 1.94188 1

6 -2 4 25.8741 1.78103 3

6 -2 4 27.2099 1.70575 5

-7 2 -4 1.09069 2.07224 4

-7 2 -4 3.17367 0.97275 5

7 -2 4 0.88048 1.06478 1

7 -2 4 0.48331 0.78745 3

-8 2 -4 5.28587 1.10712 5

-8 2 -4 7.25370 2.63684 4

8 -2 4 3.41658 0.99593 3

8 -2 4 3.05421 1.23232 1

-9 2 -4 38.5447 2.36828 5

9 -2 4 34.4547 2.63951 3

9 -2 4 38.3791 2.88700 1

-10 2 -4 1.19127 1.44571 5

10 -2 4 2.35412 1.35748 3

10 -2 4 0.89306 1.63910 1

11 -2 4 0.61018 1.83680 1

11 -2 4 0.47374 1.79672 3

12 -2 4 0.61477 2.30870 1

12 -2 4 -0.9559 2.04349 3

13 -2 4 -0.0993 2.23353 1

13 -2 4 -2.1244 2.85041 3

14 1 -4 -6.3091 2.79565 3

14 1 -4 -1.1434 3.05999 1

13 1 -4 0.43257 2.27549 3

13 1 -4 -0.8216 2.40236 1

12 1 -4 1.02797 3.35504 3

12 1 -4 0.64480 1.97556 1

11 1 -4 2.32502 1.60252 3

11 1 -4 2.15168 1.67875 1

-11 -1 4 2.38675 1.82497 1

10 1 -4 1.65889 1.38758 1

10 1 -4 1.09883 1.30972 3

-10 -1 4 2.62044 1.37605 1

9 1 -4 28.8058 2.36370 1

9 1 -4 30.6111 2.55484 3

-9 -1 4 28.9208 2.40129 1

8 1 -4 -0.4777 0.93057 3

8 1 -4 0.15783 0.92969 1

-8 -1 4 -1.0663 1.11715 5

-8 -1 4 0.59190 0.97662 1

7 1 -4 57.1815 2.76699 3

7 1 -4 54.4838 2.52433 1

-7 -1 4 55.0304 2.54626 1

-7 -1 4 55.5643 2.62658 5

6 1 -4 36.5338 2.09438 1

6 1 -4 42.7727 2.18354 3

-6 -1 4 46.4311 1.85133 1

-6 -1 4 41.8435 2.08845 5

-6 -1 4 41.0005 1.96420 1

5 1 -4 23.4177 1.29302 1

5 1 -4 21.5864 1.39833 5

5 1 -4 23.7640 1.30637 2

5 1 -4 19.6889 2.10867 3

-5 -1 4 21.6870 1.21840 1

-5 -1 4 23.1118 1.27179 1

-5 -1 4 20.9787 1.39083 5

4 1 -4 1582.82 47.8598 3

4 1 -4 1471.58 47.0398 5

4 1 -4 1576.62 47.4290 1

4 1 -4 1506.96 46.9659 2

-4 -1 4 1476.46 47.0221 1

-4 -1 4 1523.78 47.1153 1

-4 -1 4 1415.74 47.6881 5

3 1 -4 30.1742 1.33377 5

3 1 -4 38.1757 1.45182 3

3 1 -4 33.2607 1.30402 2

-3 -1 4 33.4014 1.23420 1

-3 -1 4 32.0219 1.32484 1

-3 -1 4 32.9781 1.35409 3

-3 -1 4 29.8678 1.29338 2

2 1 -4 868.505 26.5101 2

2 1 -4 898.072 27.0943 6

2 1 -4 849.904 26.3846 1

2 1 -4 784.457 26.2773 5

2 1 -4 820.822 26.6322 3

-2 -1 4 883.827 26.3950 1

-2 -1 4 829.524 26.4326 3

-2 -1 4 805.409 26.3738 2

1 1 -4 64.0469 2.15678 6

1 1 -4 60.4847 2.01622 3

1 1 -4 60.3274 2.03145 2

1 1 -4 60.1738 2.07957 1

-1 -1 4 60.2413 1.99846 3

-1 -1 4 62.1484 2.11733 6

-1 -1 4 57.6651 2.03171 2

0 1 -4 6061.14 189.932 6

0 1 -4 6045.85 190.418 4

0 1 -4 5992.32 189.643 1

0 -1 4 6175.33 189.878 6

0 -1 4 6174.08 190.336 4

-1 1 -4 56.2187 2.34482 4

-1 1 -4 60.7444 2.11350 1

-1 1 -4 57.8545 2.09404 6

1 -1 4 61.6616 2.15131 6

1 -1 4 61.8137 2.41674 4

1 -1 4 58.2735 2.07094 1

-2 1 -4 126.729 4.03846 1

-2 1 -4 121.160 4.01033 6

-2 1 -4 114.729 3.87443 5

-2 1 -4 114.191 4.23997 4

2 -1 4 125.596 4.05699 6

2 -1 4 122.039 4.31388 4

2 -1 4 118.323 3.98838 1

-3 1 -4 708.020 22.0128 1

-3 1 -4 703.564 22.1946 4

-3 1 -4 672.285 21.8254 5

3 -1 4 702.510 22.0365 6

3 -1 4 708.796 21.8759 5

3 -1 4 719.113 22.2439 4

3 -1 4 679.624 21.9536 1

-4 1 -4 20.6327 1.39604 1

-4 1 -4 17.5848 1.79320 4

-4 1 -4 19.5754 1.05818 5

4 -1 4 18.7643 1.15100 3

4 -1 4 20.7778 1.19594 5

4 -1 4 18.0580 1.28826 6

4 -1 4 20.4605 1.90959 4

4 -1 4 19.5067 1.24708 1

-5 1 -4 68.6192 3.41304 4

-5 1 -4 72.9887 2.66430 5

5 -1 4 74.2678 2.94929 3

5 -1 4 67.4572 2.85872 1

5 -1 4 75.0405 2.75977 5

-6 1 -4 58.5151 3.28144 4

6 -1 4 61.1911 2.63324 5

6 -1 4 60.1786 2.59290 3

6 -1 4 60.8492 2.72656 1

-7 1 -4 149.411 6.08812 4

-7 1 -4 153.284 5.19671 5

7 -1 4 149.116 5.50475 1

7 -1 4 144.663 5.32464 3

-8 1 -4 6.74053 1.20314 5

-8 1 -4 5.44726 2.50155 4

8 -1 4 3.92299 1.01165 3

8 -1 4 4.83186 1.21884 1

-9 1 -4 3.25054 1.28487 5

9 -1 4 5.46169 1.44937 1

9 -1 4 2.89318 1.09321 3

-10 1 -4 3.25214 1.52027 5

10 -1 4 3.20670 1.53261 1

10 -1 4 3.42841 1.48728 3

-11 1 -4 8.44540 1.92725 5

11 -1 4 6.11402 2.05973 1

11 -1 4 5.16438 1.85479 3

12 -1 4 -0.6823 2.29139 1

12 -1 4 -1.5141 2.09552 3

13 -1 4 -0.6495 2.21743 1

13 -1 4 -0.6443 2.50489 3

14 0 -4 2.79245 3.17768 1

14 0 -4 0.77572 3.28119 3

13 0 -4 1.76283 2.62570 1

13 0 -4 -0.7439 2.46721 3

12 0 -4 5.51589 2.18862 1

12 0 -4 5.48113 3.36091 3

11 0 -4 57.5033 3.76295 1

11 0 -4 62.6459 4.10130 3

10 0 -4 36.9538 2.86795 1

10 0 -4 37.7550 3.07219 3

-10 0 4 37.1133 3.06211 1

9 0 -4 8.92781 1.44051 1

9 0 -4 7.42723 1.28204 3

-9 0 4 8.94807 1.51099 1

8 0 -4 188.972 6.67223 1

8 0 -4 191.429 6.77720 3

-8 0 4 187.987 6.64315 1

7 0 -4 580.494 18.1677 3

7 0 -4 551.209 18.0651 1

-7 0 4 559.995 17.9927 1

6 0 -4 96.5355 3.68927 1

6 0 -4 103.547 3.81348 3

6 0 -4 97.6183 3.57930 2

-6 0 4 98.6500 3.67558 1

5 0 -4 77.1576 2.68209 2

5 0 -4 74.5185 2.76846 1

5 0 -4 71.8642 2.76780 5

-5 0 4 71.4236 2.72527 1

-5 0 4 71.1531 2.80501 3

-5 0 4 72.5321 2.75148 1

4 0 -4 475.340 14.5401 2

4 0 -4 466.320 14.6059 1

4 0 -4 454.899 14.6069 5

4 0 -4 449.883 14.6838 3

-4 0 4 463.214 14.6459 3

-4 0 4 464.705 14.5716 1

-4 0 4 457.370 14.5647 1

3 0 -4 38.9849 1.64508 3

3 0 -4 40.7008 1.46324 2

3 0 -4 40.9076 1.52583 5

-3 0 4 41.8003 1.58019 3

-3 0 4 38.6904 1.46247 1

-3 0 4 35.5268 1.56037 1

1 0 -4 4366.75 136.341 3

1 0 -4 4404.96 136.727 6

1 0 -4 4432.56 136.305 1

-1 0 4 4302.95 135.849 2

-1 0 4 4314.99 136.299 1

-1 0 4 4175.68 136.495 3

-1 0 4 4585.05 136.611 6

0 0 -4 748.442 24.6441 1

0 0 -4 805.992 24.5186 3

0 0 -4 810.060 24.7896 6

0 0 -4 746.074 24.4542 4

0 0 4 781.476 24.5277 1

0 0 4 776.803 24.4135 6

0 0 4 803.001 24.4910 3

0 0 4 774.749 24.4632 4

-1 0 -4 236.330 7.39265 1

-1 0 -4 222.057 7.49450 4

-1 0 -4 219.229 7.39121 6

1 0 4 229.195 7.41908 6

1 0 4 245.779 7.53508 4

1 0 4 234.124 7.37665 1

-2 0 -4 7101.22 225.036 4

-2 0 -4 7164.28 224.754 6

-2 0 -4 7124.76 224.436 1

2 0 4 7223.77 224.801 6

2 0 4 7365.51 225.152 4

-3 0 -4 548.284 17.2350 1

-3 0 -4 554.731 17.4152 4

-3 0 -4 528.992 17.1113 5

3 0 4 541.595 17.3009 6

3 0 4 526.197 17.1842 1

3 0 4 558.906 17.1688 5

3 0 4 561.600 17.4649 4

-4 0 -4 188.248 6.47348 4

-4 0 -4 187.310 6.07040 5

-4 0 -4 186.744 6.23943 1

4 0 4 182.909 6.15294 1

4 0 4 190.269 6.15042 5

4 0 4 182.139 6.30118 6

4 0 4 189.090 6.54024 4

-5 0 -4 69.5074 2.56172 5

5 0 4 66.8137 2.66730 1

5 0 4 65.4315 2.66651 5

-6 0 -4 4.84406 1.79000 4

6 0 4 3.88430 0.88724 1

6 0 4 2.96211 0.81927 3

-7 0 -4 7.87171 2.12002 4

-7 0 -4 5.61949 1.08055 5

7 0 4 4.27919 1.05119 1

7 0 4 3.39362 0.85661 3

-8 0 -4 64.4189 4.05611 4

-8 0 -4 64.5939 2.92640 5

8 0 4 61.0680 3.23276 1

8 0 4 60.6174 2.95159 3

-9 0 -4 1.93794 1.30809 5

9 0 4 0.38722 1.35213 1

9 0 4 -0.0425 1.00218 3

-10 0 -4 2.72155 1.56842 5

10 0 4 3.40115 1.55190 1

10 0 4 2.82171 1.14697 3

-11 0 -4 -0.5616 1.73594 5

11 0 4 -0.6092 1.21388 3

11 0 4 -0.6899 1.76736 1

-12 0 -4 1.60472 2.30794 5

12 0 4 2.34031 2.35303 1

12 0 4 3.45354 2.12667 3

13 0 4 1.00992 2.75564 1

13 0 4 0.84683 2.96631 3

14 -1 -4 -3.9867 2.56955 3

14 -1 -4 -0.0338 3.23150 1

13 -1 -4 -1.3524 2.56621 3

13 -1 -4 -0.3909 2.49644 1

12 -1 -4 2.66913 4.29375 3

12 -1 -4 -1.0293 2.00646 1

11 -1 -4 4.46255 1.70443 1

11 -1 -4 3.26861 1.65441 3

-11 1 4 4.04972 1.80836 1

10 -1 -4 0.59633 1.29230 3

10 -1 -4 1.06701 1.39489 1

9 -1 -4 20.4024 2.45746 3

9 -1 -4 19.3648 2.32597 1

-9 1 4 21.6937 2.68838 1

8 -1 -4 0.36127 0.89291 3

8 -1 -4 -0.5506 1.05291 1

-8 1 4 0.50299 1.18793 1

7 -1 -4 54.3918 2.76612 3

7 -1 -4 54.8643 2.70817 1

-7 1 4 56.1829 2.74860 1

6 -1 -4 51.5563 2.45781 3

6 -1 -4 46.7549 2.34946 1

-6 1 4 50.9489 2.44099 1

5 -1 -4 16.2990 1.36341 3

5 -1 -4 13.7408 1.08819 5

-5 1 4 14.0845 1.32471 1

-5 1 4 13.5737 1.16314 1

-5 1 4 15.9274 1.29644 3

4 -1 -4 1457.78 48.3539 5

4 -1 -4 1623.66 49.3015 1

4 -1 -4 1561.08 49.3083 3

-4 1 4 1543.22 48.9293 1

-4 1 4 1514.73 48.4252 3

-4 1 4 1610.61 48.7423 1

3 -1 -4 30.7972 1.46214 6

3 -1 -4 30.1868 1.30534 1

3 -1 -4 31.7920 1.46367 3

-3 1 4 28.9485 1.40789 1

-3 1 4 32.2250 1.26944 1

-3 1 4 31.7769 1.35242 3

2 -1 -4 805.958 25.5985 1

2 -1 -4 808.295 25.9027 3

2 -1 -4 856.960 26.3603 6

2 -1 -4 821.571 25.5621 2

-2 1 4 767.222 25.6055 1

-2 1 4 790.530 25.5942 3

-2 1 4 885.852 25.9746 6

1 -1 -4 63.7412 2.26603 6

1 -1 -4 58.6727 2.17541 1

1 -1 -4 59.4231 2.17933 3

-1 1 4 62.6261 2.18161 6

-1 1 4 61.5681 2.14258 3

-1 1 4 60.0880 2.19358 1

0 -1 -4 6357.00 199.808 4

0 -1 -4 6494.22 199.569 1

0 -1 -4 6345.93 199.859 6

0 1 4 6265.97 199.528 1

0 1 4 6526.94 199.769 6

-1 -1 -4 70.5336 2.55888 6

-1 -1 -4 73.4282 2.57105 1

-1 -1 -4 70.8188 2.69307 4

1 1 4 74.3957 2.57668 1

1 1 4 74.1719 2.66965 4

1 1 4 72.5831 2.60047 6

-2 -1 -4 115.090 3.87550 1

-2 -1 -4 115.038 3.87905 6

-2 -1 -4 112.880 3.99847 4

-2 -1 -4 112.089 3.79916 5

2 1 4 118.734 4.06758 4

2 1 4 110.251 3.85102 1

2 1 4 117.907 3.91696 6

-3 -1 -4 683.198 22.0535 5

-3 -1 -4 734.200 22.2928 4

-3 -1 -4 711.181 22.1404 1

3 1 4 713.609 22.1089 5

3 1 4 672.353 22.0921 1

3 1 4 726.805 22.3314 4

3 1 4 686.384 22.2098 6

-4 -1 -4 24.6993 1.35326 5

-4 -1 -4 26.5625 1.57573 1

-4 -1 -4 25.6580 1.83063 4

4 1 4 26.8984 1.46464 5

4 1 4 26.5836 1.95639 4

4 1 4 24.4874 1.39119 1

4 1 4 23.9014 1.55948 6

-5 -1 -4 61.5730 3.28207 5

-5 -1 -4 68.1543 3.15354 4

5 1 4 67.2735 2.60998 1

5 1 4 64.9751 2.72493 5

5 1 4 64.7277 3.23221 4

-6 -1 -4 66.0984 3.43037 4

-6 -1 -4 69.5933 2.75419 5

6 1 4 64.4620 2.79712 1

-7 -1 -4 129.717 5.40131 4

-7 -1 -4 132.151 4.67363 5

7 1 4 121.951 4.68080 3

7 1 4 128.900 4.75544 1

-8 -1 -4 2.26977 1.15119 5

8 1 4 3.16328 1.01221 3

8 1 4 2.75483 1.19372 1

-9 -1 -4 5.42506 1.52994 5

9 1 4 4.46517 1.38099 1

9 1 4 2.66736 0.99929 3

-10 -1 -4 6.87988 1.69963 5

10 1 4 1.89679 1.12167 3

10 1 4 2.53943 1.60950 1

-11 -1 -4 3.59443 1.96691 5

11 1 4 8.63053 2.01102 1

11 1 4 2.84439 1.28223 3

-12 -1 -4 -0.7222 2.25247 5

12 1 4 2.32064 2.36813 1

12 1 4 -0.8608 1.45404 3

-13 -1 -4 -1.4169 2.96056 5

13 1 4 1.40080 2.69537 3

13 1 4 3.38146 2.84417 1

14 -2 -4 -0.2813 3.33010 1

14 -2 -4 3.19283 3.36708 3

13 -2 -4 -0.2642 2.60359 1

13 -2 -4 -1.8260 2.35588 3

-12 2 4 2.44124 2.16115 1

11 -2 -4 11.0100 2.11615 1

11 -2 -4 11.5039 1.93046 3

-11 2 4 14.5192 2.23555 1

10 -2 -4 38.4155 3.05138 1

10 -2 -4 42.5964 3.34032 3

-10 2 4 39.6873 3.43124 1

9 -2 -4 7.98408 1.54903 1

9 -2 -4 10.7338 1.45685 3

-9 2 4 10.9184 1.84307 1

8 -2 -4 145.259 5.32219 3

8 -2 -4 126.877 5.25845 1

7 -2 -4 62.2686 3.15785 1

7 -2 -4 73.1911 3.26581 3

-7 2 4 70.9525 3.32060 1

6 -2 -4 68.7228 2.88158 3

6 -2 -4 59.2456 2.82890 1

-6 2 4 60.9920 2.89561 1

5 -2 -4 880.810 27.4764 4

5 -2 -4 853.615 27.1858 5

-5 2 4 903.902 27.4340 3

-5 2 4 853.608 27.2775 1

-5 2 4 838.793 27.2372 1

4 -2 -4 2200.50 68.9956 5

4 -2 -4 2178.64 70.0843 3

4 -2 -4 2298.48 70.2482 1

-4 2 4 2211.25 69.3403 1

-4 2 4 2153.66 69.1764 1

-4 2 4 2203.93 69.2879 3

3 -2 -4 49.6853 2.11284 6

3 -2 -4 54.1384 1.97941 1

3 -2 -4 51.5273 2.15129 3

-3 2 4 54.3465 2.09835 1

-3 2 4 57.0562 1.97566 1

-3 2 4 50.6071 1.99267 3

2 -2 -4 58.5189 2.14052 1

2 -2 -4 56.9917 2.22348 6

2 -2 -4 56.5744 2.25776 3

-2 2 4 58.6098 2.14424 3

-2 2 4 57.8017 2.23769 1

-2 2 4 60.9660 2.13471 6

1 -2 -4 2369.49 73.4436 1

1 -2 -4 2327.92 73.6439 6

1 -2 -4 2266.28 73.5973 3

-1 2 4 2315.64 73.4486 3

-1 2 4 2437.46 73.5527 6

-1 2 4 2323.83 73.5616 1

0 -2 -4 1300.27 39.9814 6

0 -2 -4 1256.48 39.8400 4

0 -2 -4 1224.11 40.0171 3

0 -2 -4 1239.54 39.7493 1

0 2 4 1311.53 40.0036 6

0 2 4 1264.63 40.0789 1

-1 -2 -4 3223.16 102.865 6

-1 -2 -4 3312.85 102.563 1

-1 -2 -4 3253.22 102.628 4

1 2 4 3212.78 102.674 1

1 2 4 3328.35 102.559 4

1 2 4 3320.78 102.856 6

-2 -2 -4 219.704 7.11815 4

-2 -2 -4 211.749 7.07871 6

-2 -2 -4 220.706 7.08083 1

-2 -2 -4 215.021 7.02540 5

2 2 4 229.652 7.16435 4

2 2 4 214.435 7.11304 6

2 2 4 215.093 7.07361 1

-3 -2 -4 352.047 11.2648 4

-3 -2 -4 341.916 11.1209 5

-3 -2 -4 357.381 11.1841 1

3 2 4 355.297 11.1751 5

3 2 4 358.380 11.3279 4

3 2 4 347.455 11.2622 6

3 2 4 332.685 11.1353 1

-4 -2 -4 44.0647 2.07980 1

-4 -2 -4 43.2007 2.29042 4

-4 -2 -4 44.5081 1.94296 5

4 2 4 42.0556 2.04852 1

4 2 4 43.2632 2.15148 6

4 2 4 40.7850 2.01045 5

4 2 4 44.5908 2.44276 4

-5 -2 -4 801.457 25.7592 4

5 2 4 785.487 25.2251 1

5 2 4 816.487 25.5975 4

-6 -2 -4 25.0368 1.71290 5

-6 -2 -4 30.3700 2.44893 4

6 2 4 24.8982 1.75484 1

-7 -2 -4 2.70852 1.09647 5

-7 -2 -4 4.40688 2.02109 4

7 2 4 4.20881 1.02991 1

-8 -2 -4 5.79842 1.39419 5

8 2 4 4.60145 1.14392 1

8 2 4 3.85564 0.93405 3

-9 -2 -4 38.6189 2.68121 5

9 2 4 34.4263 2.88823 1

9 2 4 33.1178 2.41674 3

-10 -2 -4 -0.1875 1.62965 5

10 2 4 1.23527 1.07995 3

10 2 4 1.02229 1.56749 1

-11 -2 -4 0.30523 1.95943 5

11 2 4 0.75326 1.28594 3

11 2 4 1.27555 1.93726 1

-12 -2 -4 0.71466 2.46739 5

12 2 4 0.69205 1.29086 3

12 2 4 -0.8074 2.23805 1

-13 -2 -4 5.03433 2.98708 5

13 2 4 6.21663 2.92605 1

13 2 4 4.34467 3.30595 3

13 -3 -4 0.16686 2.40959 3

13 -3 -4 2.58135 2.77356 1

12 -3 -4 0.26779 2.41515 1

12 -3 -4 -0.0657 2.35125 3

-12 3 4 0.00092 2.27307 1

11 -3 -4 0.17161 1.78874 3

11 -3 -4 2.18708 1.87328 1

-11 3 4 0.19969 2.07311 1

10 -3 -4 2.82462 1.61745 1

10 -3 -4 2.75684 1.43703 3

-10 3 4 4.01182 1.86922 1

9 -3 -4 0.39421 1.36290 1

9 -3 -4 -0.3249 1.07403 3

-9 3 4 0.00213 1.60492 1

8 -3 -4 52.7589 3.00751 3

8 -3 -4 44.6415 2.91453 1

-8 3 4 49.1888 3.29739 1

7 -3 -4 2.78274 1.04932 3

7 -3 -4 0.55880 1.16032 1

-7 3 4 -0.9312 1.23090 1

6 -3 -4 2.18478 0.86765 3

6 -3 -4 -1.0633 1.02919 1

-6 3 4 -0.6077 1.05338 1

5 -3 -4 147.853 5.29087 1

5 -3 -4 148.355 4.95926 5

5 -3 -4 145.134 5.27196 3

-5 3 4 149.328 5.24685 1

4 -3 -4 69.5191 2.84614 6

4 -3 -4 75.0335 2.86726 1

-4 3 4 72.7188 2.66965 1

-4 3 4 73.1950 2.72133 3

-4 3 4 69.8512 2.85849 1

3 -3 -4 556.018 17.6712 6

-3 3 4 569.148 17.5894 1

-3 3 4 555.301 17.6093 3

-3 3 4 550.077 17.6453 1

2 -3 -4 41.8573 1.89504 3

2 -3 -4 42.6166 1.69569 1

2 -3 -4 42.6376 1.81785 6

-2 3 4 43.8111 1.67707 6

-2 3 4 42.0508 1.72313 3

-2 3 4 39.0547 1.81870 1

-2 3 4 40.6896 1.70015 1

1 -3 -4 3123.36 97.6881 1

1 -3 -4 3021.01 97.8241 6

1 -3 -4 3076.23 98.0145 3

-1 3 4 3162.34 97.9087 1

-1 3 4 3120.48 97.7389 3

-1 3 4 3201.56 97.6071 6

0 -3 -4 1079.87 34.3528 6

0 -3 -4 1114.78 34.5658 3

0 -3 -4 1110.13 34.2609 4

0 -3 -4 1081.89 34.1564 1

0 3 4 1152.46 34.4252 6

0 3 4 1075.26 34.1869 1

0 3 4 1044.01 34.1565 3

0 3 4 1090.55 34.1229 2

-1 -3 -4 55.4988 2.04192 1

-1 -3 -4 48.5125 1.97599 6

1 3 4 50.9063 2.04584 6

1 3 4 50.5240 1.77498 2

1 3 4 49.6563 1.94724 3

1 3 4 51.5403 2.11001 1

-2 -3 -4 13.5393 1.15726 4

-2 -3 -4 17.6950 1.15381 1

-2 -3 -4 16.2445 1.05746 6

-2 -3 -4 14.6679 1.09237 5

2 3 4 17.6401 0.92245 2

2 3 4 15.2954 1.22501 4

2 3 4 18.2426 1.20661 1

2 3 4 16.7624 1.10095 6

-3 -3 -4 730.817 23.5172 4

-3 -3 -4 737.048 23.4861 5

-3 -3 -4 772.216 23.5266 1

3 3 4 775.699 23.6044 4

3 3 4 732.780 23.6029 6

3 3 4 735.026 23.4973 1

-4 -3 -4 42.1823 2.25832 4

-4 -3 -4 48.3307 2.25029 5

-4 -3 -4 49.2774 2.19249 1

4 3 4 42.9158 2.43857 4

4 3 4 44.5581 2.17020 6

4 3 4 46.7452 2.20374 1

4 3 4 44.3116 2.15846 5

-5 -3 -4 166.776 5.64881 5

-5 -3 -4 167.161 5.87728 4

5 3 4 165.551 6.08201 4

5 3 4 161.847 5.60848 1

-6 -3 -4 1.68846 1.76000 4

-6 -3 -4 0.32949 1.00678 5

6 3 4 1.92747 0.85632 1

-7 -3 -4 4.08165 1.17820 5

7 3 4 5.73915 1.02468 1

-8 -3 -4 84.4800 3.63719 5

8 3 4 77.8917 3.56284 1

-9 -3 -4 17.8080 2.41945 5

9 3 4 15.4509 2.42364 1

9 3 4 15.0198 1.97930 3

-10 -3 -4 1.61342 1.80348 5

10 3 4 0.63941 1.07003 3

10 3 4 3.41730 1.69396 1

-11 -3 -4 -0.1031 2.04869 5

11 3 4 1.22654 2.00002 1

11 3 4 0.34446 1.39542 3

-12 -3 -4 1.20325 2.70972 5

12 3 4 2.25888 1.60120 3

12 3 4 4.40884 2.35516 1

-13 -3 -4 -4.1230 3.01240 5

13 3 4 0.29536 2.85775 1

13 3 4 -0.7509 1.66211 3

13 -4 -4 -5.5873 2.65888 3

13 -4 -4 1.77258 2.81064 6

13 -4 -4 -0.9677 2.83824 1

12 -4 -4 1.39907 2.23000 1

12 -4 -4 0.14068 2.46215 3

-12 4 4 -2.0266 2.40847 1

11 -4 -4 1.51745 1.91187 1

11 -4 -4 3.68903 1.91853 3

-11 4 4 0.13705 2.20635 1

10 -4 -4 0.64370 1.62856 1

10 -4 -4 1.16101 1.64012 3

-10 4 4 -0.0731 1.90010 1

9 -4 -4 21.5585 2.77584 3

9 -4 -4 18.0276 2.62813 1

-9 4 4 18.0670 2.18282 1

8 -4 -4 34.6406 2.83057 1

-8 4 4 36.0248 3.11612 1

7 -4 -4 82.6273 3.56448 3

7 -4 -4 68.2378 3.53008 1

-7 4 4 71.4326 3.69261 1

6 -4 -4 1.95946 1.18988 1

6 -4 -4 3.59908 1.25927 6

6 -4 -4 3.78355 1.16131 3

5 -4 -4 147.989 4.93390 5

5 -4 -4 145.724 5.32356 3

5 -4 -4 152.718 5.35440 1

5 -4 -4 139.862 5.31122 6

-5 4 4 143.404 5.30288 1

4 -4 -4 564.362 18.0736 6

4 -4 -4 588.947 18.1068 4

-4 4 4 569.961 18.0662 1

-4 4 4 566.697 17.9491 1

-4 4 4 542.134 17.9468 3

3 -4 -4 1030.22 33.3121 3

3 -4 -4 1043.34 33.2538 6

-3 4 4 1105.86 33.2127 1

-3 4 4 1071.22 33.2203 3

-3 4 4 1049.90 33.2736 1

2 -4 -4 296.246 9.41645 1

2 -4 -4 289.921 9.47282 6

-2 4 4 278.668 9.47708 1

-2 4 4 305.599 9.38788 6

-2 4 4 295.339 9.45710 3

1 -4 -4 180.854 6.03427 6

1 -4 -4 175.693 6.04712 3

1 -4 -4 187.185 5.98230 1

-1 4 4 191.475 5.98515 6

-1 4 4 174.262 6.04330 1

-1 4 4 183.580 6.03533 3

0 -4 -4 13.6077 1.09036 6

0 -4 -4 14.0600 1.01422 3

0 -4 -4 14.1880 0.68498 2

0 -4 -4 14.7952 1.01896 1

0 4 4 13.7037 0.69427 2

0 4 4 12.3805 1.21382 1

0 4 4 15.6092 1.15046 6

-1 -4 -4 371.784 12.0389 1

-1 -4 -4 377.906 12.0650 6

1 4 4 371.591 12.1038 1

1 4 4 392.468 12.1030 6

1 4 4 375.484 12.0701 3

1 4 4 382.704 11.9097 2

-2 -4 -4 2.40962 0.68293 6

-2 -4 -4 2.33093 0.70441 5

-2 -4 -4 4.17900 0.71629 1

2 4 4 4.34734 0.53364 2

2 4 4 4.45987 0.79443 1

2 4 4 4.29189 0.78564 6

-3 -4 -4 6.19430 0.99113 4

-3 -4 -4 3.65112 0.84329 5

3 4 4 6.06067 1.00516 6

3 4 4 7.22160 1.19255 4

3 4 4 4.23944 0.86936 1

3 4 4 5.08019 0.76290 2

-4 -4 -4 54.8372 2.39619 1

-4 -4 -4 53.8595 2.39126 4

-4 -4 -4 55.8105 2.41309 5

4 4 4 55.0645 2.72229 6

4 4 4 54.7635 2.23861 2

4 4 4 55.8216 2.62356 4

-5 -4 -4 88.3338 3.40343 5

-5 -4 -4 88.5767 3.58971 4

5 4 4 85.8686 3.83665 4

5 4 4 86.9891 3.34560 1

-6 -4 -4 275.923 9.22849 5

6 4 4 279.229 9.14714 1

-7 -4 -4 61.1255 3.00931 5

7 4 4 63.3264 2.81680 1

-8 -4 -4 0.37624 1.25981 5

8 4 4 1.44353 1.11141 1

-9 -4 -4 24.3566 2.69989 5

9 4 4 19.6471 2.47143 1

-10 -4 -4 19.5917 2.41574 5

10 4 4 11.7601 2.00172 1

10 4 4 17.1301 2.43833 3

-11 -4 -4 -0.4507 2.15717 5

11 4 4 -0.1570 1.46540 3

11 4 4 0.20762 2.06602 1

-12 -4 -4 3.55109 2.65387 5

12 4 4 -1.1802 1.59869 3

12 4 4 0.06413 2.34040 1

-13 -4 -4 -1.1981 3.11997 5

13 4 4 1.56308 2.01423 3

13 4 4 1.71981 2.83727 1

13 -5 -4 3.10733 3.43006 6

13 -5 -4 -0.3270 3.06184 3

13 -5 -4 0.58895 2.99258 1

12 -5 -4 -0.1447 2.43231 1

12 -5 -4 0.38572 2.32291 3

-12 5 4 0.82688 2.66863 1

11 -5 -4 -1.3080 1.93796 1

11 -5 -4 -1.3083 1.82131 3

11 -5 -4 0.10865 2.31762 6

-11 5 4 -1.9133 2.28338 1

10 -5 -4 14.2428 2.32250 1

10 -5 -4 15.8810 2.07497 3

10 -5 -4 20.3586 3.18062 6

-10 5 4 15.3246 2.57574 1

9 -5 -4 9.48631 1.97089 1

9 -5 -4 10.8903 1.83292 3

-9 5 4 13.5078 2.26737 1

8 -5 -4 6.82632 1.46413 3

8 -5 -4 8.60600 1.88199 6

8 -5 -4 6.70132 1.66775 1

-8 5 4 6.60380 1.71722 1

7 -5 -4 10.7919 1.47005 3

7 -5 -4 12.9166 1.91612 6

7 -5 -4 7.66812 1.50612 1

-7 5 4 9.14027 1.58964 1

6 -5 -4 26.7553 2.31007 6

6 -5 -4 36.5317 2.65297 3

6 -5 -4 28.1164 2.41430 1

-6 5 4 26.0410 2.66652 1

5 -5 -4 97.8179 4.00949 3

5 -5 -4 93.1029 4.02448 1

-5 5 4 96.2090 4.41235 1

4 -5 -4 27.7324 1.87831 4

4 -5 -4 30.7433 1.89103 6

4 -5 -4 30.9267 2.31605 3

-4 5 4 30.5502 2.43009 1

3 -5 -4 44.4099 2.28148 4

3 -5 -4 53.4771 2.54129 3

3 -5 -4 49.6923 2.30650 6

-3 5 4 53.3013 2.19775 3

-3 5 4 53.0015 2.40499 1

-3 5 4 53.0561 2.11856 1

2 -5 -4 6.69230 0.98432 6

2 -5 -4 4.86518 0.63949 2

2 -5 -4 5.20285 0.68686 1

2 -5 -4 3.80573 0.78231 3

-2 5 4 4.95408 0.87487 1

-2 5 4 5.54858 0.81937 6

-2 5 4 4.58746 0.67802 3

-2 5 4 4.75407 0.66031 1

1 -5 -4 14.4937 1.30561 3

1 -5 -4 16.8019 1.11309 1

1 -5 -4 15.1295 1.29318 6

1 -5 -4 14.5073 0.77502 2

-1 5 4 14.9035 1.31852 3

-1 5 4 13.0987 1.39347 1

-1 5 4 15.3839 1.14506 6

0 -5 -4 384.643 12.7093 3

0 -5 -4 387.573 12.4840 2

0 -5 -4 381.199 12.6474 6

0 -5 -4 405.984 12.6608 1

0 5 4 405.318 12.4904 2

0 5 4 404.682 12.7497 1

0 5 4 410.537 12.6849 6

0 5 4 399.857 12.7184 3

-1 -5 -4 1078.46 34.7946 5

-1 -5 -4 1166.75 35.1808 6

-1 -5 -4 1097.62 34.6726 2

-1 -5 -4 1116.37 34.7873 1

1 5 4 1111.50 34.6620 2

1 5 4 1107.20 34.8485 3

1 5 4 1130.82 34.8409 6

1 5 4 1087.57 34.8552 1

-2 -5 -4 1127.34 35.7909 1

-2 -5 -4 1113.22 35.8156 5

-2 -5 -4 1157.88 35.8494 6

2 5 4 1156.51 35.8719 1

2 5 4 1167.03 35.8814 6

2 5 4 1141.96 35.7039 2

-3 -5 -4 508.653 16.2006 1

3 5 4 512.819 16.1233 2

3 5 4 507.292 16.3034 6

-4 -5 -4 32.7897 1.89087 5

4 5 4 32.2886 1.87839 1

-5 -5 -4 190.373 6.55798 5

5 5 4 185.201 6.77941 4

5 5 4 195.305 6.48605 1

-6 -5 -4 19.6627 2.03811 5

6 5 4 23.5838 2.48041 4

6 5 4 22.9726 1.85685 1

-7 -5 -4 1.49342 1.15943 5

7 5 4 1.52014 1.01799 1

-8 -5 -4 18.6116 2.38040 5

8 5 4 19.2337 2.21984 1

-9 -5 -4 -2.1507 1.52761 5

9 5 4 -0.1218 1.33527 1

-10 -5 -4 9.14459 2.11897 5

10 5 4 8.10299 1.93222 1

10 5 4 4.69200 1.30124 3

-11 -5 -4 6.38931 2.50265 5

11 5 4 1.53224 1.64427 3

11 5 4 0.80923 2.09718 1

-12 -5 -4 4.50834 2.86075 5

12 5 4 -0.7841 2.30874 1

12 5 4 0.83645 1.77416 3

-13 -5 -4 -1.6402 3.31114 5

13 5 4 0.49036 2.90115 1

13 5 4 -1.1344 1.76209 3

13 -6 -4 0.57356 3.51226 6

13 -6 -4 -4.0318 3.20252 3

-13 6 4 3.35785 3.82572 1

12 -6 -4 5.22504 3.08799 3

12 -6 -4 2.60444 2.66293 1

12 -6 -4 2.45880 3.72252 6

-12 6 4 4.32999 2.92592 1

11 -6 -4 1.35525 2.25895 3

11 -6 -4 5.23193 2.61114 6

11 -6 -4 1.76564 2.36776 1

-11 6 4 4.00287 2.69308 1

10 -6 -4 2.24849 2.23132 6

10 -6 -4 0.10227 1.99181 1

10 -6 -4 0.88657 1.75841 3

-10 6 4 0.65770 2.20318 1

9 -6 -4 0.19371 1.72582 1

9 -6 -4 0.86156 1.81284 6

9 -6 -4 -0.0711 1.43939 3

-9 6 4 2.35698 2.03827 1

8 -6 -4 2.47786 1.53802 3

8 -6 -4 0.69906 1.50598 1

8 -6 -4 2.58701 1.61982 6

-8 6 4 0.83808 1.71338 1

7 -6 -4 0.97806 1.37547 1

7 -6 -4 4.21547 1.53193 6

7 -6 -4 4.89624 1.40116 3

-7 6 4 2.95020 1.53451 1

6 -6 -4 2.13369 1.28599 3

6 -6 -4 0.65926 1.23414 1

6 -6 -4 2.32910 1.36497 6

-6 6 4 0.30045 1.34506 1

5 -6 -4 294.250 10.1279 3

-5 6 4 302.176 10.1567 1

4 -6 -4 135.246 5.11692 4

4 -6 -4 142.843 5.12294 6

4 -6 -4 143.479 5.36640 3

-4 6 4 144.436 5.28122 1

3 -6 -4 39.6251 2.02676 4

3 -6 -4 37.8042 2.13833 6

2 -6 -4 3.93240 0.93304 4

2 -6 -4 0.76434 0.48805 2

2 -6 -4 0.01704 0.60577 1

2 -6 -4 2.35910 0.94937 6

-2 6 4 1.57234 0.67460 1

-2 6 4 -0.0095 0.69242 3

-2 6 4 1.59423 0.61890 6

-2 6 4 0.32923 0.95707 1

1 -6 -4 40.0448 1.88715 1

1 -6 -4 41.7380 1.61028 2

1 -6 -4 42.5913 2.09045 3

1 -6 -4 42.4007 2.00191 6

-1 6 4 45.4476 2.11287 3

-1 6 4 36.4186 2.25011 1

-1 6 4 46.6265 1.87909 6

0 -6 -4 505.403 16.3004 3

0 -6 -4 499.443 16.1948 6

0 -6 -4 492.376 16.0467 2

0 -6 -4 516.905 16.2123 1

0 6 4 523.912 16.2201 6

0 6 4 523.060 16.3090 3

0 6 4 521.337 16.0546 2

0 6 4 506.192 16.3087 1

-1 -6 -4 885.448 27.5274 1

-1 -6 -4 863.367 27.5449 5

-1 -6 -4 858.892 27.5219 6

-1 -6 -4 850.176 27.4096 2

1 6 4 876.983 27.6582 1

1 6 4 883.374 27.4046 2

1 6 4 884.017 27.6210 3

1 6 4 909.762 27.5842 6

-2 -6 -4 19.5537 1.56679 1

-2 -6 -4 23.0128 1.39648 6

-2 -6 -4 22.1357 1.78151 5

2 6 4 22.9683 1.29630 2

2 6 4 23.7018 1.52924 6

2 6 4 23.4600 2.03220 1

2 6 4 23.3838 1.48276 3

-3 -6 -4 230.578 7.45925 5

-3 -6 -4 235.548 7.53028 1

3 6 4 202.985 7.61342 1

3 6 4 225.071 7.50645 6

3 6 4 220.849 7.31444 2

-4 -6 -4 152.570 5.39392 5

4 6 4 152.119 5.40189 1

-5 -6 -4 127.077 4.73904 5

5 6 4 127.823 4.63830 1

-6 -6 -4 52.3136 2.78808 5

6 6 4 52.0848 3.31483 4

6 6 4 53.9351 2.63325 1

-7 -6 -4 24.6611 2.19454 5

7 6 4 28.1117 2.12467 1

-8 -6 -4 38.4889 2.96555 5

8 6 4 43.2782 2.80232 1

-9 -6 -4 8.00491 1.85187 5

9 6 4 8.04015 1.62188 1

-10 -6 -4 17.2122 2.50504 5

10 6 4 14.2287 2.10941 1

-11 -6 -4 0.77019 2.52259 5

11 6 4 -1.5641 1.49623 3

11 6 4 -1.9580 2.19192 1

-12 -6 -4 8.34239 3.18871 5

12 6 4 3.83596 1.76924 3

12 6 4 1.66065 2.61330 1

13 -7 -4 -1.2245 3.35852 3

13 -7 -4 1.39096 3.71380 6

-13 7 4 -2.4641 4.09221 1

12 -7 -4 2.45655 3.03201 3

12 -7 -4 9.04240 3.48770 6

-12 7 4 12.9288 3.52104 1

11 -7 -4 0.15386 2.81288 6

11 -7 -4 -1.7510 2.90077 3

-11 7 4 -1.7811 2.62113 1

10 -7 -4 2.14297 2.01438 3

10 -7 -4 0.30666 2.20068 1

10 -7 -4 -1.1910 2.20074 6

-10 7 4 4.45363 2.59612 1

9 -7 -4 3.57128 1.94701 1

9 -7 -4 4.47967 1.88269 3

9 -7 -4 3.86972 2.09174 6

-9 7 4 2.24060 2.24158 1

8 -7 -4 19.0135 2.92689 6

8 -7 -4 10.1955 2.11471 1

-8 7 4 14.1983 2.32814 1

7 -7 -4 82.9710 4.16754 3

7 -7 -4 72.4422 3.81817 6

-7 7 4 69.1220 4.12447 1

6 -7 -4 87.3673 3.92007 6

6 -7 -4 88.5520 4.24679 3

-6 7 4 86.3210 4.34566 1

5 -7 -4 57.1480 3.27482 3

-5 7 4 48.1541 3.25838 1

4 -7 -4 10.1414 1.45348 3

4 -7 -4 15.7911 2.03127 6

4 -7 -4 13.1769 1.94591 4

-4 7 4 8.97497 1.55584 1

3 -7 -4 840.327 26.7423 3

3 -7 -4 821.826 26.5583 6

3 -7 -4 849.718 26.5667 4

-3 7 4 852.366 26.7353 1

2 -7 -4 234.656 7.78734 6

2 -7 -4 232.659 8.06431 3

2 -7 -4 231.033 7.52262 2

-2 7 4 243.282 7.60900 6

-2 7 4 233.372 7.75136 1

-2 7 4 225.804 7.92040 1

1 -7 -4 464.922 15.5594 6

1 -7 -4 469.623 15.4072 2

-1 7 4 520.399 15.8879 3

-1 7 4 499.900 15.5261 6

-1 7 4 492.622 15.7564 1

0 -7 -4 65.4802 2.45951 2

0 -7 -4 62.5866 2.57983 6

0 7 4 69.1658 2.72955 6

0 7 4 67.1713 2.46547 2

0 7 4 69.8550 2.98969 3

-1 -7 -4 584.531 18.9828 2

-1 -7 -4 605.453 19.7319 5

-1 -7 -4 597.049 19.0771 6

1 7 4 610.187 18.9823 2

1 7 4 633.401 19.1664 6

1 7 4 587.083 19.4349 1

-2 -7 -4 52.5019 2.26143 2

-2 -7 -4 58.3964 2.32604 6

-2 -7 -4 53.3291 2.46352 5

2 7 4 53.5412 2.24494 2

2 7 4 55.7237 2.50715 6

2 7 4 57.7659 2.73693 1

-3 -7 -4 397.093 12.8673 5

3 7 4 406.341 12.9311 1

3 7 4 388.947 12.8985 6

-4 -7 -4 123.142 4.65434 5

4 7 4 127.415 4.68134 1

-5 -7 -4 64.6396 3.03923 5

5 7 4 64.4505 2.95415 1

-6 -7 -4 29.5367 2.35448 5

6 7 4 29.7743 2.07986 1

-7 -7 -4 23.7110 2.41619 5

7 7 4 25.5271 2.33740 1

-8 -7 -4 -0.8602 1.53395 5

8 7 4 1.74610 1.18910 1

-9 -7 -4 6.97438 2.00047 5

9 7 4 5.68656 1.57713 1

-10 -7 -4 0.23013 2.20298 5

10 7 4 -0.2803 1.75383 1

-11 -7 -4 -0.8800 2.64054 5

11 7 4 2.27207 1.68845 3

-12 -7 -4 2.43503 3.16092 5

12 7 4 0.13138 1.76710 3

12 7 4 -2.2455 2.64398 1

12 -8 -4 3.07408 3.45417 6

12 -8 -4 -4.9501 3.03730 3

-12 8 4 5.23929 5.05318 1

11 -8 -4 0.48936 2.93040 6

11 -8 -4 4.38606 2.61874 3

-11 8 4 7.54605 3.10816 1

10 -8 -4 6.00123 2.65997 6

10 -8 -4 3.64262 2.45258 3

-10 8 4 3.29497 2.74174 1

9 -8 -4 -0.2810 2.13031 6

9 -8 -4 -0.0414 1.94920 3

-9 8 4 -0.5999 2.28682 1

8 -8 -4 6.05431 2.05306 6

8 -8 -4 2.88661 1.89004 3

-8 8 4 3.36144 2.20922 1

7 -8 -4 67.2697 4.13659 3

7 -8 -4 61.3699 3.68016 6

-7 8 4 62.7063 4.33634 1

6 -8 -4 8.59640 1.70317 3

6 -8 -4 14.5276 1.94960 6

-6 8 4 8.49849 1.94486 1

5 -8 -4 0.45781 1.19409 3

-5 8 4 0.77320 1.51966 1

4 -8 -4 44.7379 2.88430 4

4 -8 -4 48.2956 2.75180 6

4 -8 -4 44.3201 3.05980 3

-4 8 4 37.8945 2.96501 1

3 -8 -4 454.517 14.8196 3

3 -8 -4 450.853 14.3835 2

3 -8 -4 447.121 14.5657 4

3 -8 -4 449.491 14.5865 6

-3 8 4 459.606 14.8347 1

2 -8 -4 84.7760 3.11909 2

2 -8 -4 84.7975 3.44267 6

2 -8 -4 82.7026 3.68876 3

-2 8 4 89.7972 3.37332 1

-2 8 4 84.0586 3.67975 1

-2 8 4 88.7603 3.12136 6

1 -8 -4 236.290 7.81248 2

1 -8 -4 227.080 7.91507 6

1 -8 -4 246.032 8.19775 3

-1 8 4 248.978 7.89964 6

-1 8 4 240.347 8.19032 1

-1 8 4 247.581 8.04583 1

0 -8 -4 344.631 11.0034 6

0 -8 -4 339.088 10.9310 2

0 8 4 351.563 11.0569 6

0 8 4 331.606 11.2413 1

-1 -8 -4 103.462 3.84176 6

-1 -8 -4 106.786 3.82347 2

-1 -8 -4 105.067 4.02253 5

1 8 4 115.331 4.28214 1

1 8 4 109.338 4.05443 6

1 8 4 107.091 3.80867 2

-2 -8 -4 9.92671 1.24628 5

2 8 4 11.8376 1.73524 6

2 8 4 7.69952 1.33758 1

-3 -8 -4 489.781 16.2324 5

3 8 4 520.196 16.3175 1

-4 -8 -4 393.713 12.8095 5

4 8 4 390.338 12.8146 1

-5 -8 -4 10.3957 1.58236 5

5 8 4 10.2967 1.33438 1

-6 -8 -4 -0.5026 1.33907 5

6 8 4 0.88762 1.10615 1

-7 -8 -4 2.00927 1.49229 5

7 8 4 5.09334 1.37494 1

-8 -8 -4 2.47274 1.74089 5

8 8 4 1.02764 1.32792 1

-9 -8 -4 2.89899 2.02555 5

9 8 4 0.90989 1.64164 1

-10 -8 -4 -0.1291 3.18706 5

10 8 4 -3.3818 1.79225 1

-11 -8 -4 2.34737 3.05022 5

11 8 4 -1.4448 2.40248 1

11 8 4 0.29396 1.69382 3

-12 -8 -4 1.74715 3.65569 5

12 8 4 -1.6137 1.88387 3

12 8 4 4.85277 2.79097 1

12 -9 -4 -3.7156 3.38868 3

12 -9 -4 -4.8714 3.51204 6

-12 9 4 -1.8573 3.79457 1

11 -9 -4 -2.8767 2.89448 3

11 -9 -4 -2.0610 2.99405 6

-11 9 4 5.87449 3.55691 1

10 -9 -4 1.12733 2.57025 6

10 -9 -4 1.53694 2.62014 3

-10 9 4 3.38228 3.05676 1

9 -9 -4 8.78436 2.36698 3

9 -9 -4 7.66541 2.49105 6

-9 9 4 9.53852 2.89963 1

8 -9 -4 5.01317 2.02564 3

8 -9 -4 3.83993 2.11621 6

-8 9 4 1.18295 2.30726 1

7 -9 -4 28.4610 3.03475 6

7 -9 -4 25.0746 3.68376 3

-7 9 4 18.7217 2.69757 1

6 -9 -4 376.780 12.7405 3

6 -9 -4 361.020 12.4464 6

-6 9 4 372.004 12.9510 1

5 -9 -4 262.993 9.58957 6

5 -9 -4 274.968 9.62276 3

-5 9 4 276.628 9.73734 1

4 -9 -4 118.578 4.30571 2

4 -9 -4 116.309 4.62665 6

4 -9 -4 117.268 4.97837 3

-4 9 4 120.289 5.04358 1

3 -9 -4 124.334 4.37290 2

3 -9 -4 122.802 4.67918 6

3 -9 -4 125.520 4.98237 3

-3 9 4 121.441 5.03437 1

2 -9 -4 212.656 7.03734 2

2 -9 -4 209.635 7.17625 6

2 -9 -4 212.599 7.49587 3

-2 9 4 210.199 7.57037 1

-2 9 4 218.263 6.98312 6

1 -9 -4 88.1258 3.76953 3

1 -9 -4 90.0515 3.40854 6

1 -9 -4 91.8275 3.36302 2

-1 9 4 92.2835 3.61804 1

-1 9 4 88.4841 3.94272 1

-1 9 4 94.4433 3.39119 6

0 -9 -4 363.629 11.9007 5

0 -9 -4 361.600 11.7747 6

0 9 4 381.828 11.8700 6

0 9 4 359.604 12.1058 1

-1 -9 -4 16.5930 1.39684 6

-1 -9 -4 15.1829 1.75267 5

1 9 4 16.7547 1.89390 6

1 9 4 15.7358 1.65700 1

-2 -9 -4 567.799 18.2122 2

-2 -9 -4 563.039 18.3135 5

2 9 4 570.413 18.3485 6

2 9 4 589.874 18.4904 1

-3 -9 -4 68.2214 3.07912 5

3 9 4 69.5758 3.42901 1

-4 -9 -4 232.417 8.10148 5

4 9 4 241.661 8.20411 1

-5 -9 -4 11.8631 1.61164 5

5 9 4 13.3595 1.53877 1

-6 -9 -4 123.023 4.91063 5

6 9 4 115.324 4.73004 1

-7 -9 -4 -0.1818 1.47722 5

7 9 4 0.09778 1.27561 1

-8 -9 -4 23.4028 3.17587 5

8 9 4 13.8699 1.85552 1

-9 -9 -4 -1.1374 2.18993 5

9 9 4 1.60979 1.71195 1

-10 -9 -4 0.35865 2.81201 5

10 9 4 0.76841 2.08564 1

-11 -9 -4 6.30360 3.36573 5

11 9 4 -2.0661 2.50474 1

12 -10 -4 -6.4776 3.61834 3

-12 10 4 3.92179 4.23647 1

11 -10 -4 -1.8902 2.87214 3

-11 10 4 3.97671 3.78894 1

10 -10 -4 -3.8656 2.77448 6

-10 10 4 6.49148 3.43495 1

9 -10 -4 0.80350 2.51563 3

9 -10 -4 0.91772 2.44283 6

-9 10 4 0.47175 2.92451 1

8 -10 -4 -1.1394 2.18715 6

8 -10 -4 1.71837 2.03195 3

-8 10 4 1.27158 2.49900 1

7 -10 -4 1.80339 1.63440 3

7 -10 -4 2.30610 1.95237 6

-7 10 4 -0.6193 2.25978 1

6 -10 -4 83.9675 4.23969 6

6 -10 -4 85.4008 4.67605 3

-6 10 4 87.9903 5.08311 1

5 -10 -4 26.4092 2.87219 6

5 -10 -4 32.7875 2.13600 2

5 -10 -4 25.8904 3.17900 3

-5 10 4 29.9796 3.47278 1

4 -10 -4 47.8875 3.29982 3

4 -10 -4 46.1054 2.43465 2

4 -10 -4 48.2284 2.82038 6

-4 10 4 47.7972 3.43861 1

3 -10 -4 60.1392 3.31035 3

3 -10 -4 57.9592 2.91592 6

-3 10 4 56.1327 3.51679 1

2 -10 -4 26.8998 1.89313 6

2 -10 -4 29.2911 2.49218 3

-2 10 4 30.8502 2.79149 1

1 -10 -4 13.5626 1.97704 3

1 -10 -4 13.0867 1.41417 6

1 -10 -4 14.0425 1.73497 5

-1 10 4 13.2790 1.44466 6

-1 10 4 11.1712 1.61717 1

-1 10 4 12.5663 1.68344 1

0 -10 -4 98.8264 3.80305 5

0 -10 -4 99.1376 3.55325 6

0 -10 -4 97.3544 3.60485 2

0 10 4 96.7356 4.22430 1

0 10 4 92.7749 3.73020 6

0 10 4 93.5729 3.87889 1

-1 -10 -4 12.7824 1.58981 2

-1 -10 -4 19.5280 1.90543 5

1 10 4 13.8049 2.08751 6

1 10 4 18.0864 2.47848 1

-2 -10 -4 137.861 5.04802 5

-2 -10 -4 133.202 4.89295 2

2 10 4 133.650 5.07560 6

2 10 4 142.354 5.36149 1

-3 -10 -4 -0.7471 1.20654 5

-3 -10 -4 -0.7389 0.94397 2

3 10 4 -0.6128 1.33618 1

-4 -10 -4 150.406 5.67297 5

4 10 4 154.340 5.75523 1

-5 -10 -4 3.48584 1.53185 5

5 10 4 1.39301 1.47443 1

-6 -10 -4 80.2848 4.04093 5

6 10 4 86.9622 3.87377 1

-7 -10 -4 6.49397 1.79554 5

7 10 4 3.20761 1.52068 1

-8 -10 -4 -1.1292 2.15960 5

8 10 4 0.84683 1.53590 1

-9 -10 -4 9.05061 3.04541 5

-10 -10 -4 0.62334 2.93439 5

10 10 4 0.02319 2.17466 1

-11 -10 -4 -2.7087 3.38188 5

11 10 4 -2.4541 2.58354 1

11 -11 -4 -5.0493 3.36709 6

11 -11 -4 0.68625 3.40987 3

-11 11 4 0.71407 3.90724 1

10 -11 -4 5.01156 3.04075 6

10 -11 -4 6.99918 3.18959 3

-10 11 4 5.58236 3.74540 1

9 -11 -4 9.13791 2.66901 3

9 -11 -4 9.85347 2.87349 6

-9 11 4 8.14214 3.41371 1

8 -11 -4 30.0656 3.80110 6

8 -11 -4 42.1922 4.73782 3

-8 11 4 40.8941 5.34311 1

7 -11 -4 3.40608 2.12260 6

7 -11 -4 7.37645 1.96930 3

-7 11 4 9.37899 2.78026 1

6 -11 -4 68.1256 3.84940 6

6 -11 -4 82.4149 4.49625 3

6 -11 -4 71.9178 3.41418 2

-6 11 4 68.7825 5.03437 1

5 -11 -4 9.21434 1.71268 3

5 -11 -4 10.8603 2.29383 6

5 -11 -4 10.0342 1.49336 2

-5 11 4 8.57182 2.15796 1

4 -11 -4 45.0509 2.50032 2

4 -11 -4 49.5939 2.86675 6

4 -11 -4 47.6518 3.42543 3

-4 11 4 37.6043 3.74640 1

3 -11 -4 88.9886 3.70510 6

3 -11 -4 96.4432 3.75558 2

3 -11 -4 95.2468 4.35855 3

-3 11 4 97.8067 4.77909 1

2 -11 -4 159.767 5.96945 3

2 -11 -4 151.667 5.39656 6

2 -11 -4 160.406 5.52807 2

-2 11 4 156.006 6.17923 1

1 -11 -4 181.098 6.12859 2

1 -11 -4 176.121 6.19685 5

1 -11 -4 173.868 5.94272 6

-1 11 4 170.544 5.92892 6

-1 11 4 181.715 6.68417 1

0 -11 -4 16.9269 1.85411 5

0 -11 -4 14.7787 1.67889 2

0 -11 -4 14.9124 1.48271 6

0 11 4 14.9235 1.87396 1

0 11 4 11.6999 1.84341 6

0 11 4 14.9335 2.02511 1

-1 -11 -4 20.5997 1.92490 5

-1 -11 -4 15.9774 1.79576 2

1 11 4 14.7474 2.11835 6

1 11 4 19.3010 2.60290 1

-2 -11 -4 10.0055 1.55074 5

-2 -11 -4 10.1029 1.33018 2

2 11 4 9.61877 1.71986 1

-3 -11 -4 73.9842 3.35256 2

-3 -11 -4 75.0007 3.60098 5

3 11 4 81.3874 3.94487 1

-4 -11 -4 37.5897 2.83108 5

4 11 4 41.3756 3.00983 1

-5 -11 -4 42.7730 3.02533 5

5 11 4 39.9786 3.08166 1

-6 -11 -4 5.13486 1.74356 5

6 11 4 3.83072 1.52502 1

-7 -11 -4 14.8857 2.33836 5

7 11 4 14.9604 1.90640 1

-8 -11 -4 1.74649 2.36974 5

8 11 4 -1.4770 1.63881 1

-9 -11 -4 1.71976 2.74672 5

-10 -11 -4 -0.9319 3.12781 5

10 11 4 -2.7609 2.29992 1

10 -12 -4 -5.2116 4.04580 6

10 -12 -4 -2.0081 2.81241 3

-10 12 4 8.90203 3.98064 1

9 -12 -4 0.22583 3.99998 3

9 -12 -4 1.36232 2.70174 6

-9 12 4 2.22669 3.51830 1

8 -12 -4 18.7124 2.80089 6

8 -12 -4 18.9897 2.70935 3

-8 12 4 19.4674 3.60030 1

7 -12 -4 18.2201 2.61142 3

7 -12 -4 19.1093 2.55651 6

7 -12 -4 17.7286 2.59905 2

-7 12 4 18.8327 3.43002 1

6 -12 -4 17.1544 2.30397 2

6 -12 -4 13.9383 2.24125 3

6 -12 -4 17.2327 2.25371 6

-6 12 4 12.4049 2.83704 1

5 -12 -4 37.8603 3.67083 3

5 -12 -4 27.6391 2.43562 2

-5 12 4 30.6920 4.12649 1

4 -12 -4 70.1716 3.25329 2

4 -12 -4 68.4361 4.07153 3

4 -12 -4 66.7100 3.15709 6

-4 12 4 66.9603 4.55195 1

3 -12 -4 2.39198 1.37341 3

3 -12 -4 2.77555 1.23813 2

-3 12 4 2.33061 1.98678 1

2 -12 -4 6.40205 1.27656 2

2 -12 -4 7.01881 1.42794 5

2 -12 -4 3.69017 1.25071 3

-2 12 4 4.69336 1.89548 1

1 -12 -4 30.3936 2.31163 5

1 -12 -4 31.5046 1.74910 6

1 -12 -4 32.0253 2.19826 2

-1 12 4 28.6530 1.77476 6

-1 12 4 24.6283 3.15946 1

0 -12 -4 -0.3989 1.04275 2

0 -12 -4 -1.1718 1.20301 5

0 12 4 -0.6552 1.59798 1

-1 -12 -4 170.350 5.89360 2

-1 -12 -4 170.732 5.99423 5

1 12 4 153.827 5.91827 6

1 12 4 166.237 6.46966 1

1 12 4 163.213 6.04845 1

-2 -12 -4 23.4501 2.17310 2

-2 -12 -4 25.3190 2.39558 5

2 12 4 24.1644 3.04620 1

-3 -12 -4 59.7176 3.12114 2

-3 -12 -4 65.2499 3.33812 5

3 12 4 61.3242 3.78466 1

-4 -12 -4 54.9642 3.33949 5

4 12 4 56.9548 3.51769 1

-5 -12 -4 44.0909 3.32457 5

5 12 4 47.8656 3.27184 1

-6 -12 -4 2.52208 2.04625 5

6 12 4 2.79752 1.62987 1

-7 -12 -4 11.3222 2.45937 5

7 12 4 9.32276 1.90397 1

-8 -12 -4 6.35701 2.69365 5

8 12 4 2.81460 1.88804 1

-9 -12 -4 4.38953 3.15187 5

9 12 4 -0.8230 2.01377 1

-10 -12 -4 3.76323 3.49392 5

10 12 4 -4.1095 2.35167 1

10 -13 -4 1.01091 3.10101 3

-10 13 4 1.68000 4.29020 1

9 -13 -4 1.87081 2.61371 3

9 -13 -4 1.30211 1.93466 2

-9 13 4 -5.9100 3.50958 1

8 -13 -4 1.69273 2.30929 3

8 -13 -4 1.59641 1.76631 2

-8 13 4 0.95281 3.46686 1

7 -13 -4 6.15012 2.20916 6

7 -13 -4 6.19069 1.76641 2

7 -13 -4 5.71511 2.42384 3

-7 13 4 4.92841 3.37839 1

6 -13 -4 16.0312 1.99006 2

6 -13 -4 15.1819 2.46946 3

6 -13 -4 21.4777 2.87430 6

-6 13 4 14.3508 3.21529 1

5 -13 -4 -1.6982 1.55285 3

5 -13 -4 0.91996 1.39777 2

-5 13 4 -0.6343 2.56373 1

4 -13 -4 11.3976 1.67088 2

4 -13 -4 12.2564 1.89348 3

-4 13 4 6.66991 2.56888 1

3 -13 -4 2.50442 1.40054 5

3 -13 -4 0.82505 1.37350 3

3 -13 -4 3.88302 1.38585 2

-3 13 4 2.95071 2.15920 1

2 -13 -4 8.43819 1.49879 5

2 -13 -4 7.83494 1.39797 2

-2 13 4 4.73219 2.02772 1

1 -13 -4 2.13903 1.42702 5

1 -13 -4 0.87075 1.17345 2

-1 13 4 0.55045 1.80892 1

0 -13 -4 42.1918 2.59912 2

0 -13 -4 40.0918 2.76812 5

0 13 4 44.8499 3.63471 1

-1 -13 -4 28.8448 2.57612 5

-1 -13 -4 28.0395 2.31992 2

1 13 4 30.3074 3.31044 1

-2 -13 -4 10.8533 1.57678 2

-2 -13 -4 14.3511 1.90357 5

2 13 4 11.0624 2.12049 1

-3 -13 -4 49.1103 3.04550 2

-3 -13 -4 55.3299 3.25927 5

3 13 4 47.3827 3.68283 1

-4 -13 -4 3.79900 1.75864 5

4 13 4 2.65615 1.81018 1

-5 -13 -4 3.05054 1.96281 5

5 13 4 4.26542 1.81076 1

-6 -13 -4 8.14158 2.38932 5

6 13 4 7.59120 1.88932 1

-7 -13 -4 14.6365 3.07682 5

7 13 4 9.36701 2.06067 1

-8 -13 -4 -2.8234 2.72143 5

8 13 4 -3.9713 1.93088 1

-9 -13 -4 3.32161 3.20253 5

9 13 4 -1.6971 2.26488 1

9 -14 -4 2.60325 2.16019 2

9 -14 -4 3.77473 2.96554 3

-9 14 4 3.44036 4.09942 1

8 -14 -4 1.04262 1.96715 2

8 -14 -4 3.69891 2.65681 3

-8 14 4 -0.4477 3.80391 1

7 -14 -4 9.27599 1.99101 2

7 -14 -4 11.3905 2.78397 3

-7 14 4 7.65458 3.75689 1

6 -14 -4 5.24007 1.82319 2

6 -14 -4 6.96462 2.30161 3

5 -14 -4 2.74519 1.56644 5

5 -14 -4 0.84065 1.68564 2

5 -14 -4 1.06518 1.94333 3

-5 14 4 5.55758 2.98590 1

4 -14 -4 5.28978 1.67128 2

4 -14 -4 5.44407 1.55991 5

4 -14 -4 4.98614 1.86205 3

-4 14 4 3.26221 2.68388 1

3 -14 -4 2.42788 1.66833 3

3 -14 -4 0.54576 1.50427 5

3 -14 -4 3.27996 1.55969 2

-3 14 4 2.48496 2.84026 1

2 -14 -4 22.5323 2.51566 2

2 -14 -4 26.0386 2.46145 5

-2 14 4 18.8615 2.67493 1

1 -14 -4 52.4378 3.06522 5

1 -14 -4 46.2668 2.96254 2

-1 14 4 52.2524 4.19688 1

0 -14 -4 14.4809 1.89747 5

0 -14 -4 7.78299 1.59845 2

0 14 4 10.1066 2.24453 1

-1 -14 -4 10.2773 1.65337 2

-1 -14 -4 9.62292 1.78344 5

1 14 4 11.7071 2.24191 1

-2 -14 -4 0.64964 1.63390 5

-2 -14 -4 0.11648 1.34087 2

2 14 4 2.66921 2.02555 1

-3 -14 -4 0.97697 1.52697 2

-3 -14 -4 -1.5586 1.67102 5

3 14 4 1.24829 1.98906 1

-4 -14 -4 1.45030 1.89001 5

4 14 4 1.23564 1.94729 1

-5 -14 -4 20.9280 2.65726 5

5 14 4 15.7204 2.26443 1

6 14 4 19.3567 2.31163 1

-7 -14 -4 12.8588 3.13449 5

7 14 4 12.9960 2.26676 1

-8 -14 -4 4.75687 3.18312 5

8 -15 -4 9.73896 3.07250 3

-8 15 4 7.02333 4.30615 1

7 -15 -4 1.39303 2.09022 2

7 -15 -4 4.79874 2.62684 3

-7 15 4 -2.1775 3.75156 1

6 -15 -4 1.82715 2.27237 3

6 -15 -4 -1.5644 1.86173 2

-6 15 4 -5.6360 3.39530 1

5 -15 -4 2.91708 2.02083 3

5 -15 -4 2.97174 1.86320 2

-5 15 4 -0.4009 3.19300 1

4 -15 -4 2.95192 1.81177 3

4 -15 -4 2.19529 1.52825 5

4 -15 -4 2.28152 1.75610 2

-4 15 4 0.40201 3.10094 1

3 -15 -4 8.40486 1.68402 5

3 -15 -4 9.46375 1.78591 2

-3 15 4 5.05811 2.81577 1

2 -15 -4 1.24696 1.60966 5

2 -15 -4 -0.1285 1.46186 2

-2 15 4 3.21342 2.54878 1

1 -15 -4 16.1364 1.98668 2

1 -15 -4 17.3783 2.66549 5

-1 15 4 15.3002 2.67028 1

0 -15 -4 6.39274 1.65585 2

0 -15 -4 5.66516 1.79414 5

0 15 4 7.11879 2.39313 1

-1 -15 -4 1.79366 1.51618 2

-1 -15 -4 -1.1755 1.65912 5

1 15 4 -2.6092 2.19469 1

-2 -15 -4 9.15509 1.80163 2

-2 -15 -4 8.07386 1.98449 5

2 15 4 6.46084 2.33703 1

-3 -15 -4 1.49273 1.98028 5

3 15 4 0.10469 2.22176 1

-4 -15 -4 33.0812 3.56784 5

4 15 4 32.6023 3.70614 1

-5 -15 -4 20.3246 4.86522 5

5 15 4 9.88206 2.33014 1

-6 -15 -4 7.30255 2.83421 5

6 15 4 3.81864 2.14722 1

-7 -15 -4 0.37626 2.87408 5

-8 -15 -4 16.4898 3.82305 5

7 -16 -4 2.74513 2.69798 3

-7 16 4 -0.7654 4.18448 1

6 -16 -4 8.92262 2.49152 3

6 -16 -4 4.95226 2.29523 2

-6 16 4 10.1756 4.03651 1

5 -16 -4 7.63632 2.24734 2

-5 16 4 3.78622 3.68774 1

4 -16 -4 1.14755 1.90908 2

-4 16 4 4.05784 3.24958 1

3 -16 -4 2.00461 1.82502 2

3 -16 -4 1.69326 1.61235 5

-3 16 4 -2.2115 2.90311 1

2 -16 -4 0.14950 1.76403 2

2 -16 -4 2.02024 1.76560 5

1 -16 -4 4.45033 1.76151 2

1 -16 -4 -0.2545 1.67923 5

-1 16 4 -1.0057 2.60873 1

0 -16 -4 0.70906 1.80904 5

0 -16 -4 3.43071 1.71697 2

0 16 4 0.43371 2.52589 1

-1 -16 -4 9.08801 1.91806 2

-1 -16 -4 4.65208 1.93315 5

1 16 4 6.14953 2.59009 1

-2 -16 -4 2.22799 1.85526 2

-2 -16 -4 2.77126 2.03818 5

2 16 4 3.25787 2.53833 1

-3 -16 -4 3.60300 2.19454 5

3 16 4 0.74206 2.48208 1

4 16 4 -1.4608 2.27008 1

-5 -16 -4 13.1236 2.91269 5

-6 -16 -4 1.22187 3.00571 5

6 16 4 4.00272 2.31892 1

7 16 4 -4.0154 2.34119 1

6 -17 -4 6.88433 2.67742 3

-6 17 4 -6.5807 4.14115 1

5 -17 -4 3.47697 2.05676 2

-5 17 4 2.62571 3.93074 1

4 -17 -4 11.9814 2.43442 2

-4 17 4 7.75133 3.91785 1

3 -17 -4 1.58345 2.12824 2

-3 17 4 -2.9617 3.26597 1

2 -17 -4 3.16647 2.03766 2

2 -17 -4 3.42347 1.82988 5

-2 17 4 -0.0155 3.06349 1

1 -17 -4 -0.2467 1.92075 2

1 -17 -4 2.04080 1.90103 5

-1 17 4 0.59066 2.90002 1

0 -17 -4 23.7094 3.42682 2

0 -17 -4 25.9771 3.21831 5

0 17 4 20.3885 3.41189 1

-1 -17 -4 -0.3449 1.96223 2

-1 -17 -4 4.80830 2.17105 5

1 17 4 -0.1263 2.74947 1

-2 -17 -4 5.10098 2.37416 2

-2 -17 -4 6.79074 2.32383 5

2 17 4 0.67791 2.69437 1

-3 -17 -4 11.4884 3.12061 5

3 17 4 12.7297 3.01197 1

-4 -17 -4 0.65446 2.64415 5

4 17 4 0.83583 2.57391 1

-5 -17 -4 -1.0656 2.90952 5

5 17 4 2.68755 2.60579 1

-4 18 4 -1.2305 4.01424 1

3 -18 -4 0.59352 2.30897 2

-3 18 4 -0.1801 3.66820 1

2 -18 -4 16.7063 2.70813 2

-2 18 4 18.5517 3.88702 1

1 -18 -4 2.99623 2.35340 2

1 -18 -4 6.30057 3.17656 5

-1 18 4 0.41028 3.33986 1

0 -18 -4 0.94415 2.27350 2

0 -18 -4 2.93987 2.14522 5

0 18 4 -6.7938 3.12458 1

-1 -18 -4 2.18665 2.31731 5

1 18 4 -3.3932 2.92972 1

-2 -18 -4 8.98687 2.74033 2

-2 -18 -4 10.5228 2.62268 5

2 18 4 7.21114 3.29590 1

-3 -18 -4 -0.1026 2.68874 5

3 18 4 -2.2769 2.96524 1

-4 -18 5 2.30313 2.67370 2

-3 -18 5 -0.6436 2.65838 2

-2 -18 5 3.14070 2.91901 2

1 18 -5 4.09166 2.69294 1

-1 -18 5 -2.0631 2.61157 2

0 18 -5 -1.8546 2.76454 1

0 -18 5 0.09263 2.65263 2

-1 18 -5 -1.1179 2.91702 1

1 -18 5 2.85184 2.78004 2

-2 18 -5 0.01910 3.08065 1

2 -18 5 2.90472 2.84769 2

-3 18 -5 7.42814 3.39388 1

3 -18 5 7.32020 3.07801 2

-6 -17 5 0.50301 2.49787 5

-6 -17 5 0.65483 3.03293 2

-5 -17 5 -1.9485 2.29830 5

-5 -17 5 2.18001 2.80041 2

-4 -17 5 0.18259 1.70836 5

-4 -17 5 -1.0901 2.35371 2

-3 -17 5 4.89601 2.50170 2

-3 -17 5 -0.1898 2.37578 5

-2 -17 5 0.17184 2.52298 2

1 17 -5 4.29131 2.37336 1

-1 -17 5 5.30867 2.73085 2

0 17 -5 3.36052 2.94901 1

0 -17 5 5.33095 2.43613 2

-1 17 -5 2.41091 2.63127 1

1 -17 5 -0.1864 2.62733 2

-2 17 -5 5.74594 2.76427 1

2 -17 5 6.59143 2.64880 2

-3 17 -5 1.49557 2.84374 1

3 -17 5 4.65036 2.68109 2

-4 17 -5 8.16397 3.16563 1

4 -17 5 4.21558 2.92195 2

-5 17 -5 1.28079 3.38111 1

5 -17 5 3.97447 2.86834 2

-7 -16 5 1.48235 2.66769 5

-6 -16 5 7.42492 2.63570 2

-6 -16 5 4.48559 3.36999 5

-5 -16 5 21.1226 3.09701 2

-5 -16 5 23.1223 2.49363 5

-4 -16 5 2.28439 2.36222 2

-4 -16 5 0.18284 1.72562 5

-3 -16 5 -1.1017 1.52631 5

-3 -16 5 0.04226 2.26148 2

2 16 -5 -1.3600 1.89955 1

-2 -16 5 -0.5143 1.27132 5

1 16 -5 -1.8176 1.96137 1

-1 -16 5 -1.1905 1.97336 2

0 16 -5 49.1863 4.21492 1

0 -16 5 46.4838 4.31368 2

-1 16 -5 6.58469 2.47019 1

1 -16 5 5.59326 2.39100 2

-2 16 -5 2.97518 2.99330 1

2 -16 5 6.22500 2.54630 2

-3 16 -5 4.37739 2.70696 1

3 -16 5 2.22788 3.20990 2

-4 16 -5 4.02168 2.77201 1

4 -16 5 1.66533 2.46142 2

-5 16 -5 1.37317 2.94675 1

5 -16 5 0.09550 2.40305 2

-6 16 -5 -0.0068 3.08510 1

6 -16 5 -0.9978 2.61998 2

-8 -15 5 2.17740 2.79486 5

-7 -15 5 8.30955 2.55820 5

-6 -15 5 0.24193 2.08691 2

-6 -15 5 1.50043 2.13109 5

-5 -15 5 12.7695 2.26301 5

-5 -15 5 14.5005 2.48954 2

-4 -15 5 2.79415 1.69989 5

-4 -15 5 3.93274 2.20510 2

-3 -15 5 3.42148 1.52387 5

-3 -15 5 5.06788 2.08126 2

2 15 -5 21.8146 2.88549 1

-2 -15 5 19.9492 2.53015 2

-2 -15 5 17.6927 2.20802 5

1 15 -5 3.52956 1.88991 1

-1 -15 5 6.40809 2.10345 2

0 15 -5 2.36980 2.00744 1

0 -15 5 3.13581 2.15863 2

-1 15 -5 1.81132 2.05381 1

1 -15 5 -2.3842 1.76406 2

-2 15 -5 34.8984 3.99311 1

2 -15 5 33.6772 3.74418 2

-3 15 -5 -0.1047 2.32801 1

3 -15 5 -0.7262 2.05203 2

-4 15 -5 -1.7790 3.16479 1

4 -15 5 4.31242 2.24141 2

-5 15 -5 7.90417 2.79649 1

-6 15 -5 5.23064 2.79110 1

6 -15 5 -0.6844 2.23980 2

-7 15 -5 -1.6910 2.97857 1

7 -15 5 1.89625 2.45659 2

9 14 -5 2.31881 2.41777 3

-9 -14 5 -0.1208 2.93522 5

-8 -14 5 -0.0667 2.55052 5

7 14 -5 18.6910 2.80713 6

-7 -14 5 21.0896 2.80346 5

6 14 -5 28.3741 3.90927 6

-6 -14 5 36.4584 3.54738 5

-5 -14 5 11.5304 2.32044 2

-5 -14 5 12.4512 2.12378 5

-4 -14 5 17.9543 2.01924 5

-4 -14 5 14.3315 2.31663 2

-3 -14 5 72.5155 4.18206 2

-3 -14 5 79.8835 3.62809 5

2 14 -5 35.4343 2.81891 1

-2 -14 5 36.1792 3.26580 2

-2 -14 5 31.9691 2.35973 5

1 14 -5 -1.5790 1.61508 1

-1 -14 5 0.76110 1.83679 2

0 14 -5 2.55772 1.73384 1

0 -14 5 2.20454 1.82978 2

-1 14 -5 56.7344 3.79523 1

1 -14 5 53.2565 3.69287 2

-2 14 -5 7.09238 2.02091 1

2 -14 5 8.01072 1.98148 2

-3 14 -5 16.6581 3.19731 4

-3 14 -5 12.6783 2.31685 1

3 -14 5 16.1584 2.28289 2

-4 14 -5 9.67255 2.35911 1

-4 14 -5 11.5874 3.20086 4

4 -14 5 7.97813 2.19063 2

-5 14 -5 5.78392 3.41957 4

-5 14 -5 6.04945 3.76366 1

5 -14 5 4.31120 2.07854 2

5 -14 5 5.17341 1.29901 3

-6 14 -5 3.95066 2.52635 1

6 -14 5 -0.1632 2.42446 2

-7 14 -5 8.88605 2.97912 1

7 -14 5 5.83777 2.48566 2

7 -14 5 10.1488 2.11199 3

-8 14 -5 1.11645 2.94487 1

10 13 -5 2.36556 2.47429 3

-10 -13 5 0.71724 3.14279 5

9 13 -5 6.90426 2.92968 3

-9 -13 5 5.03653 2.86167 5

-7 -13 5 24.2979 3.46869 5

-7 -13 5 27.4373 4.24219 2

-6 -13 5 55.5018 3.74035 5

-6 -13 5 51.8667 3.75023 2

5 13 -5 29.1233 4.16863 6

-5 -13 5 41.8215 3.17069 5

-5 -13 5 39.9481 3.24843 2

-4 -13 5 28.3900 2.98149 2

-4 -13 5 35.8199 2.70928 5

-3 -13 5 -0.6574 1.24614 5

-3 -13 5 -2.0244 1.30887 2

2 13 -5 14.1056 2.28101 1

-2 -13 5 17.7676 1.88245 5

-2 -13 5 18.1980 2.16005 2

1 13 -5 107.701 4.54411 1

-1 -13 5 109.950 4.84750 2

0 13 -5 19.1265 2.77247 1

0 -13 5 19.5013 2.78203 2

-1 13 -5 91.5509 4.38997 1

1 -13 5 91.6485 4.36658 2

-2 13 -5 1.62116 1.57308 1

2 -13 5 -0.7414 1.55388 2

-3 13 -5 2.06684 1.76117 1

-3 13 -5 0.81572 2.58024 4

3 -13 5 0.16643 1.64122 2

-4 13 -5 -2.0066 2.66513 4

-4 13 -5 2.71815 1.92609 1

4 -13 5 1.10347 1.75258 2

-5 13 -5 3.16852 2.10095 1

-5 13 -5 5.30179 3.16283 4

5 -13 5 3.48016 1.88893 2

5 -13 5 4.38451 1.29164 3

-6 13 -5 37.8692 4.52435 4

-6 13 -5 44.0096 4.37097 1

6 -13 5 36.0546 4.60831 1

6 -13 5 37.1063 3.33237 3

-7 13 -5 15.0456 2.92318 1

7 -13 5 10.3574 1.92531 3

7 -13 5 9.91954 2.81973 1

-8 13 -5 0.34349 2.82536 1

8 -13 5 0.96313 1.90647 3

8 -13 5 -1.1214 2.86353 1

-9 13 -5 5.39021 3.33301 1

9 -13 5 0.73235 3.23802 3

9 -13 5 0.97699 3.39263 1

10 12 -5 -0.0335 2.26419 3

-10 -12 5 2.01677 2.94404 5

9 12 -5 0.72209 1.87200 3

-9 -12 5 1.53742 2.55048 5

-8 -12 5 15.5824 2.50199 5

-7 -12 5 1.29330 1.75740 2

-7 -12 5 -0.6212 1.89565 5

-6 -12 5 4.08421 1.75977 5

-6 -12 5 3.68553 1.78056 2

-5 -12 5 0.73321 1.58835 2

-5 -12 5 1.15541 1.58267 5

-4 -12 5 75.6775 3.52845 5

-4 -12 5 74.1567 3.78944 2

-3 -12 5 -1.5664 1.19926 2

-3 -12 5 0.48811 1.14362 5

2 12 -5 51.7728 2.71351 1

-2 -12 5 46.7055 3.18521 2

-2 -12 5 54.6683 2.56135 5

1 12 -5 73.9223 3.39194 1

1 12 -5 67.4238 3.08713 1

-1 -12 5 72.3184 3.64852 2

0 12 -5 0.64386 1.11673 1

0 12 -5 0.98201 1.30243 1

0 -12 5 1.96034 1.50670 2

-1 12 -5 26.4136 2.14014 1

-1 12 -5 30.2088 2.57161 1

-1 12 -5 22.3474 1.72811 3

1 -12 5 26.6703 2.68383 2

-2 12 -5 4.04077 1.60409 1

-2 12 -5 7.45495 2.23655 4

2 -12 5 3.42161 1.50874 2

-3 12 -5 22.9479 3.18832 4

-3 12 -5 27.2017 2.93449 1

3 -12 5 18.6785 2.66924 2

-4 12 -5 33.4792 3.70320 4

4 -12 5 39.3856 2.58455 3

4 -12 5 34.2297 3.41347 1

4 -12 5 34.0918 2.97781 2

-5 12 -5 1.17563 2.84320 4

5 -12 5 3.16645 1.24964 3

5 -12 5 0.31032 1.68972 1

-6 12 -5 17.4653 3.77292 4

6 -12 5 8.75145 1.61772 3

6 -12 5 12.2456 2.33664 1

-7 12 -5 6.52928 3.74951 4

7 -12 5 2.82978 2.21726 1

7 -12 5 3.06819 1.68550 3

-8 12 -5 41.8823 4.73862 1

8 -12 5 31.8946 4.92808 1

8 -12 5 25.4160 3.98245 3

-9 12 -5 2.74104 2.93966 1

9 -12 5 0.19820 2.99177 1

9 -12 5 -0.2538 2.11379 3

-10 12 -5 3.58006 3.34329 1

10 -12 5 -1.4239 3.14791 1

11 11 -5 -0.9113 2.44624 3

-11 -11 5 -1.0728 3.18223 5

10 11 -5 1.02612 2.33158 3

-10 -11 5 5.40710 2.85936 5

9 11 -5 0.07848 1.82452 3

-9 -11 5 1.05307 3.34791 5

-8 -11 5 3.42915 2.02738 5

-7 -11 5 -3.7085 1.67981 5

-6 -11 5 42.2861 3.07534 2

-6 -11 5 42.8665 3.06416 5

-5 -11 5 36.3618 2.78868 2

-5 -11 5 40.6878 2.76876 5

4 11 -5 162.950 5.83153 6

4 11 -5 174.260 5.89965 6

-4 -11 5 178.223 6.20369 5

-4 -11 5 176.942 6.45977 2

-3 -11 5 29.5246 2.49445 2

-3 -11 5 30.3343 2.13440 5

2 11 -5 101.728 3.94855 1

-2 -11 5 103.726 4.34680 2

-2 -11 5 105.220 3.92448 5

1 11 -5 1.35226 0.92971 1

1 11 -5 2.66742 1.14156 1

1 11 -5 2.64897 0.96722 3

-1 -11 5 4.24094 1.01246 5

-1 -11 5 2.56360 1.36489 2

0 11 -5 30.3784 2.08701 3

0 11 -5 35.4408 2.37589 1

0 11 -5 35.4868 2.12702 1

0 -11 5 38.0705 2.59942 2

-1 11 -5 6.59778 1.36216 1

-1 11 -5 4.51326 0.86916 3

-1 11 -5 7.92097 1.19598 1

1 -11 5 7.72913 1.58064 2

-2 11 -5 1.99190 0.75380 3

-2 11 -5 4.32622 1.24712 1

-2 11 -5 2.92372 2.14824 4

2 -11 5 3.93201 1.34550 2

-3 11 -5 81.2972 3.35195 3

-3 11 -5 84.9315 4.36477 4

3 -11 5 87.4889 3.99113 2

-4 11 -5 70.1847 4.17076 4

4 -11 5 66.4525 3.84975 1

4 -11 5 65.4179 3.31356 3

-5 11 -5 1.82551 2.74272 4

5 -11 5 2.12302 1.64883 1

5 -11 5 1.70075 1.19059 3

-6 11 -5 -0.2289 2.90037 4

6 -11 5 0.96045 1.64976 1

6 -11 5 1.52899 1.38376 3

-7 11 -5 5.76566 3.57456 4

7 -11 5 4.39783 1.70454 3

7 -11 5 5.55246 2.00347 1

8 -11 5 7.38029 1.99462 3

8 -11 5 9.02689 2.46270 1

9 -11 5 -1.5625 2.24792 3

9 -11 5 1.12212 2.44819 1

-10 11 -5 1.38078 3.04917 1

10 -11 5 -2.3212 2.59925 3

10 -11 5 2.86449 3.07109 1

12 10 -5 -1.3734 2.45121 3

11 10 -5 -0.4245 2.15348 3

-11 -10 5 0.62650 3.04893 5

10 10 -5 0.46278 1.90027 3

-10 -10 5 0.39193 2.68549 5

9 10 -5 12.8149 2.04530 3

-9 -10 5 20.5961 2.78060 5

8 10 -5 10.4780 1.71847 3

-8 -10 5 11.7349 2.14223 5

-7 -10 5 5.07984 1.73665 5

-6 -10 5 47.1129 3.01202 2

-6 -10 5 43.7596 2.96656 5

-5 -10 5 8.86069 1.49421 2

-5 -10 5 11.3735 1.61934 5

4 10 -5 26.3079 1.70654 6

-4 -10 5 26.9201 2.36765 2

-4 -10 5 24.5833 2.06543 5

-3 -10 5 69.7861 3.34719 2

-3 -10 5 75.4223 3.06336 5

2 10 -5 32.7174 1.95786 1

2 10 -5 31.7158 1.76100 1

2 10 -5 36.1047 2.04240 3

-2 -10 5 34.2149 2.43808 2

1 10 -5 3.52596 0.97304 1

1 10 -5 1.76582 0.83557 3

-1 -10 5 4.00745 0.89678 5

-1 -10 5 2.56699 1.26080 2

0 10 -5 44.1665 2.36971 1

0 10 -5 37.3235 2.07483 3

0 10 -5 38.2853 2.12390 1

0 -10 5 41.6953 2.55315 2

-1 10 -5 14.3097 2.52364 4

-1 10 -5 17.5197 1.44276 3

-1 10 -5 16.6679 1.76242 1

1 -10 5 18.8393 2.67134 4

1 -10 5 19.2442 2.13282 2

-2 10 -5 78.9245 3.46412 1

-2 10 -5 77.0503 2.99218 3

-2 10 -5 79.5334 4.01667 4

2 -10 5 83.4113 3.66335 1

2 -10 5 87.8045 3.68400 2

-3 10 -5 1.21702 0.69440 3

-3 10 -5 -0.3973 2.06992 4

3 -10 5 1.57819 1.30121 1

3 -10 5 1.26670 0.85040 3

-4 10 -5 30.8253 3.33006 4

4 -10 5 34.4288 2.90461 3

4 -10 5 35.2343 2.78083 1

-5 10 -5 176.089 7.14289 4

5 -10 5 171.571 6.40303 3

5 -10 5 176.980 6.75642 1

-6 10 -5 52.5434 4.34013 4

6 -10 5 59.9241 3.76900 1

6 -10 5 52.9787 3.39600 3

-7 10 -5 2.97473 3.01881 4

7 -10 5 -0.4508 1.57795 3

7 -10 5 0.68612 1.79562 1

-8 10 -5 10.5971 3.90278 4

8 -10 5 5.38495 1.97785 3

8 -10 5 9.83350 2.17099 1

9 -10 5 7.91555 2.42680 1

9 -10 5 4.66975 2.32086 3

10 -10 5 -4.6941 2.46523 3

10 -10 5 1.40832 2.56179 1

-11 10 -5 7.16750 3.49952 1

11 -10 5 -5.1666 3.07679 3

11 -10 5 0.31973 2.98734 1

12 9 -5 2.56221 2.29709 3

-12 -9 5 -0.2686 3.45507 5

11 9 -5 2.69198 2.03201 3

-11 -9 5 5.48561 3.06331 5

10 9 -5 -2.2048 1.79802 3

-10 -9 5 -0.2548 4.54050 5

9 9 -5 -2.5407 1.44876 3

-9 -9 5 -0.2823 2.08466 5

8 9 -5 11.8023 1.64916 3

-8 -9 5 13.2501 2.01014 5

-7 -9 5 11.9081 1.87181 5

-6 -9 5 29.4707 2.45023 2

-6 -9 5 25.6018 2.49217 5

-5 -9 5 1.47096 1.25307 2

-5 -9 5 -2.1864 1.21819 5

-4 -9 5 103.977 4.17914 2

-4 -9 5 105.483 4.05365 5

3 9 -5 16.3002 1.51049 3

3 9 -5 11.5312 1.27195 6

-3 -9 5 12.7862 1.45914 2

-3 -9 5 12.4813 1.59579 5

2 9 -5 41.6933 1.95575 3

2 9 -5 33.5407 2.04106 1

2 9 -5 38.6849 1.96702 5

-2 -9 5 36.8722 2.43788 2

1 9 -5 33.0284 1.75779 5

1 9 -5 36.2470 1.94965 1

1 9 -5 35.8989 1.79698 1

1 9 -5 36.9392 1.89312 3

1 9 -5 29.2497 2.41428 4

-1 -9 5 35.9803 1.74281 5

-1 -9 5 32.6974 2.28446 2

0 9 -5 42.0040 2.05660 1

0 9 -5 35.0658 2.59318 4

0 9 -5 43.2657 1.96744 3

0 -9 5 40.4045 2.33492 2

0 -9 5 39.4532 2.89245 4

-1 9 -5 4.69566 0.68450 3

-1 9 -5 5.18472 1.03387 1

1 -9 5 4.03227 2.17039 4

1 -9 5 5.82965 1.03725 1

1 -9 5 3.67637 1.25060 2

-2 9 -5 4.60977 0.62077 3

-2 9 -5 4.59011 1.18221 1

2 -9 5 5.37101 1.33488 2

2 -9 5 4.89125 0.79466 3

2 -9 5 1.79813 1.10983 1

-3 9 -5 1.45026 0.63501 3

-3 9 -5 -1.8469 1.82105 4

3 -9 5 1.33038 1.30255 1

3 -9 5 1.10353 0.81990 3

-4 9 -5 14.0376 1.55751 3

-4 9 -5 18.9727 2.64884 4

4 -9 5 12.5195 1.61990 1

4 -9 5 16.6879 2.12859 3

-5 9 -5 6.49599 2.53652 4

5 -9 5 6.89991 1.39870 1

5 -9 5 6.35189 1.29662 3

-6 9 -5 23.3812 3.68671 4

6 -9 5 28.8975 2.76870 1

6 -9 5 21.6940 2.85596 3

-7 9 -5 16.4465 3.31733 4

7 -9 5 11.1559 1.79880 3

7 -9 5 10.5149 1.85415 1

-8 9 -5 2.39115 3.33517 4

8 -9 5 1.82181 1.72636 1

8 -9 5 -1.2542 1.74158 3

9 -9 5 1.50168 1.97457 1

9 -9 5 -0.0336 2.06148 3

10 -9 5 -0.7984 2.41472 3

10 -9 5 0.41913 2.53175 1

11 -9 5 -6.9338 3.13767 3

11 -9 5 0.00343 2.59518 1

12 8 -5 0.39225 2.19109 3

-12 -8 5 1.96775 3.36866 5

11 8 -5 0.29174 3.97337 3

-11 -8 5 1.32947 2.73392 5

10 8 -5 5.15134 1.79750 3

-10 -8 5 7.87295 2.25227 5

9 8 -5 0.66366 1.34681 3

-9 -8 5 1.59922 1.90714 5

8 8 -5 8.83401 1.52437 3

-8 -8 5 15.0902 2.06733 5

7 8 -5 2.32168 1.06323 3

-7 -8 5 -0.6568 1.45235 5

-6 -8 5 14.5302 1.73995 5

-6 -8 5 10.9198 1.52599 2

-5 -8 5 -0.1549 0.96507 2

-5 -8 5 0.96750 1.21563 5

4 8 -5 395.601 12.8828 4

-4 -8 5 390.699 12.7234 5

-4 -8 5 387.437 12.7584 2

3 8 -5 166.297 5.67455 4

-3 -8 5 137.287 5.53990 5

-3 -8 5 159.465 5.52052 2

2 8 -5 0.90029 0.56371 1

2 8 -5 0.65157 0.75946 5

2 8 -5 0.27044 0.60195 1

2 8 -5 0.88811 0.57876 3

-2 -8 5 -0.4278 0.85729 2

-2 -8 5 1.26706 0.87427 5

1 8 -5 33.6573 1.64403 3

1 8 -5 30.2905 1.49046 5

1 8 -5 27.5756 2.17156 4

1 8 -5 29.4429 1.58707 1

-1 -8 5 33.4418 1.56749 5

-1 -8 5 30.2886 2.01142 2

0 8 -5 192.134 6.87242 4

0 8 -5 198.130 6.48111 3

0 8 -5 206.308 6.61887 1

0 -8 5 198.577 6.59341 1

0 -8 5 199.287 7.05458 4

0 -8 5 197.803 6.80739 2

-1 8 -5 52.4634 3.04935 4

-1 8 -5 68.1271 2.64827 1

-1 8 -5 65.9798 2.32770 3

1 -8 5 62.0175 2.86137 2

1 -8 5 59.0200 3.37704 4

1 -8 5 62.5947 2.65008 1

-2 8 -5 619.078 18.5496 1

-2 8 -5 572.004 18.2292 3

2 -8 5 589.820 19.2781 4

2 -8 5 566.822 18.3158 3

2 -8 5 552.051 18.5211 1

-3 8 -5 43.2725 2.43952 1

-3 8 -5 38.7029 2.94942 4

-3 8 -5 44.5108 1.95087 3

3 -8 5 40.2405 2.24038 3

3 -8 5 43.8122 2.40353 1

-4 8 -5 5.45139 0.82488 3

-4 8 -5 9.88851 2.27158 4

4 -8 5 6.47106 1.11243 3

4 -8 5 6.16940 1.23644 1

-5 8 -5 71.8288 4.10981 4

5 -8 5 82.6565 3.50867 1

5 -8 5 71.0250 2.96890 6

5 -8 5 71.6156 3.45788 3

-6 8 -5 157.515 6.60553 4

6 -8 5 155.652 6.01539 3

6 -8 5 156.934 6.02829 1

-7 8 -5 1.06290 2.96761 4

7 -8 5 3.46495 1.49206 1

7 -8 5 2.74356 1.48461 3

-8 8 -5 -0.2204 3.06696 4

8 -8 5 -2.7351 1.52721 3

8 -8 5 1.33757 1.58336 1

9 -8 5 3.40634 1.95506 3

9 -8 5 2.40977 1.79181 1

10 -8 5 -1.9595 2.13228 3

10 -8 5 0.96802 2.29498 1

11 -8 5 -4.8681 3.34970 3

11 -8 5 9.17216 2.62944 1

12 -8 5 -3.1283 3.24476 3

12 -8 5 -2.0420 2.89568 1

13 7 -5 2.81725 2.90773 3

12 7 -5 2.49251 1.94511 3

-12 -7 5 1.97272 2.95555 5

11 7 -5 0.95536 1.66545 3

-11 -7 5 1.34631 2.56454 5

10 7 -5 4.24272 1.65563 3

-10 -7 5 7.09635 2.23258 5

9 7 -5 27.7991 2.82545 3

-9 -7 5 25.0452 3.02308 5

8 7 -5 -0.9058 1.09889 3

-8 -7 5 -0.8407 1.39922 5

7 7 -5 79.1087 3.50411 3

-7 -7 5 76.1870 3.57211 5

-6 -7 5 34.0276 2.27173 2

-6 -7 5 30.6805 2.29970 5

-5 -7 5 182.391 6.37399 5

-5 -7 5 180.131 6.31805 2

-4 -7 5 811.091 25.8570 5

-4 -7 5 822.856 25.8525 2

3 7 -5 865.558 26.7827 5

3 7 -5 861.948 26.9764 4

-3 -7 5 845.560 26.9013 2

-3 -7 5 835.362 26.8827 5

2 7 -5 648.901 20.9742 4

2 7 -5 663.166 20.6667 6

2 7 -5 665.204 20.7326 5

-2 -7 5 655.759 20.9051 2

-2 -7 5 663.241 20.7959 5

1 7 -5 600.227 18.5300 3

1 7 -5 591.735 18.5518 1

1 7 -5 586.206 18.5130 5

1 7 -5 579.289 18.8106 4

-1 -7 5 582.398 18.7535 2

-1 -7 5 600.274 18.5642 5

0 7 -5 95.2988 3.89409 4

0 7 -5 103.210 3.54657 1

0 -7 5 100.538 3.48868 1

0 -7 5 100.112 3.72232 2

0 -7 5 99.1382 4.05214 4

-1 7 -5 105.877 4.26845 4

-1 7 -5 117.934 3.99487 1

-1 7 -5 111.216 3.66385 3

1 -7 5 112.218 4.08334 2

1 -7 5 104.922 3.93271 1

1 -7 5 110.021 4.47818 4

-2 7 -5 150.594 5.73926 4

-2 7 -5 153.476 5.14450 1

-2 7 -5 153.110 4.81253 3

2 -7 5 141.234 5.11770 1

2 -7 5 142.254 4.90680 3

2 -7 5 136.723 5.55163 4

-3 7 -5 124.665 5.15101 4

-3 7 -5 132.901 4.83870 1

-3 7 -5 137.833 4.46420 3

3 -7 5 130.247 4.80338 1

3 -7 5 128.979 4.64585 3

-4 7 -5 427.714 14.1897 4

-4 7 -5 439.164 13.6136 3

4 -7 5 429.583 13.9231 1

4 -7 5 422.016 13.7070 3

4 -7 5 408.128 13.5365 6

-5 7 -5 10.0498 2.29283 4

5 -7 5 10.3255 1.49518 6

5 -7 5 7.74711 1.24914 1

-6 7 -5 24.0819 3.13052 4

6 -7 5 29.8703 2.32234 1

6 -7 5 25.6679 2.40190 3

-7 7 -5 14.1936 3.09325 4

7 -7 5 10.1179 1.62543 1

7 -7 5 18.1092 2.71939 3

-8 7 -5 19.7138 3.52322 4

8 -7 5 19.2311 2.67135 1

8 -7 5 13.8675 1.82768 3

-9 7 -5 0.85039 3.49926 4

9 -7 5 0.96081 1.65921 1

9 -7 5 0.92849 1.76610 3

10 -7 5 3.61977 2.09239 3

10 -7 5 3.28915 1.92170 1

11 -7 5 -1.3241 2.44263 3

11 -7 5 -1.1827 2.23147 1

12 -7 5 -1.2353 2.52217 1

13 6 -5 -2.2884 2.68363 3

12 6 -5 0.36126 2.18065 3

12 6 -5 -1.0092 2.03603 1

-12 -6 5 -1.9976 2.99508 5

11 6 -5 3.21359 1.69762 3

11 6 -5 1.02609 1.62089 1

-11 -6 5 5.09419 2.40990 5

10 6 -5 -2.2794 1.22276 3

10 6 -5 -0.5561 1.22685 1

-10 -6 5 -0.1087 1.84585 5

9 6 -5 4.16173 1.08203 1

9 6 -5 3.11482 1.33781 3

-9 -6 5 7.79553 1.87264 5

8 6 -5 11.0305 1.42153 3

-8 -6 5 9.69734 1.72230 5

7 6 -5 0.70511 0.99026 3

-7 -6 5 1.99484 1.34370 5

6 6 -5 91.5506 3.49218 3

-6 -6 5 84.4312 3.50848 2

-6 -6 5 80.5223 3.56871 5

-5 -6 5 389.064 12.3627 2

-5 -6 5 371.482 12.4005 5

-4 -6 5 173.111 5.79844 2

-4 -6 5 163.692 5.81882 5

3 6 -5 115.829 4.50129 4

3 6 -5 120.570 4.07145 5

-3 -6 5 112.954 4.14311 5

-3 -6 5 118.163 4.19938 2

2 6 -5 839.662 25.6588 6

2 6 -5 828.086 25.6810 5

2 6 -5 826.086 25.8135 2

2 6 -5 794.021 25.8938 4

-2 -6 5 812.390 25.7434 5

1 6 -5 1738.96 54.7092 1

1 6 -5 1746.72 55.0530 4

1 6 -5 1753.07 54.6340 5

-1 -6 5 1668.34 54.6997 2

-1 -6 5 1852.51 55.0078 5

0 6 -5 140.419 5.04456 1

0 -6 5 147.666 5.39266 4

0 -6 5 148.223 4.91610 1

0 -6 5 158.165 5.31014 2

-1 6 -5 43.3207 2.40060 4

-1 6 -5 44.8555 1.99838 1

1 -6 5 47.5352 2.57005 4

1 -6 5 42.4071 1.91133 1

-2 6 -5 -0.5973 1.30583 4

-2 6 -5 0.49933 0.77436 1

-2 6 -5 1.10944 0.41713 3

2 -6 5 1.75058 0.69360 1

2 -6 5 1.01525 0.54358 3

2 -6 5 2.58174 1.56060 4

-3 6 -5 455.433 16.0459 4

-3 6 -5 501.014 15.6384 1

-3 6 -5 512.386 15.3740 3

3 -6 5 492.012 15.6091 1

3 -6 5 484.745 15.3523 6

3 -6 5 468.568 15.3977 3

4 -6 5 33.2406 1.78725 6

4 -6 5 37.7608 2.03145 3

-5 6 -5 27.5888 2.95882 4

5 -6 5 30.9622 2.18993 1

5 -6 5 30.9585 1.87566 6

5 -6 5 31.2762 2.13054 3

-6 6 -5 63.1807 3.97100 4

6 -6 5 68.4017 3.23510 1

6 -6 5 66.3832 3.16064 3

-7 6 -5 5.62186 2.55694 4

7 -6 5 4.38411 1.31851 1

7 -6 5 5.66932 1.17836 3

-8 6 -5 31.3351 4.07725 4

8 -6 5 33.8382 3.15579 3

8 -6 5 37.0384 2.96950 1

-9 6 -5 0.56018 3.24815 4

9 -6 5 2.18006 1.64276 1

9 -6 5 0.00706 1.52979 3

10 -6 5 1.65154 1.75000 1

10 -6 5 1.02293 1.89070 3

11 -6 5 1.96267 2.08347 1

11 -6 5 -1.8901 2.35368 3

12 -6 5 1.87370 2.43558 1

12 -6 5 -4.4075 2.81754 3

13 5 -5 1.16451 2.64782 3

12 5 -5 -2.3356 1.97797 1

12 5 -5 -3.0867 2.09180 3

11 5 -5 10.5004 1.80538 3

11 5 -5 12.5892 1.88011 1

-11 -5 5 14.7803 2.70306 5

10 5 -5 91.4979 4.49447 3

10 5 -5 93.3408 4.12545 1

-10 -5 5 103.542 4.60469 5

9 5 -5 3.79877 1.30613 3

9 5 -5 7.04558 1.16374 1

-9 -5 5 6.15480 1.76948 5

8 5 -5 29.6530 2.39546 3

8 5 -5 27.4290 1.87656 1

-8 -5 5 26.6738 2.38648 5

7 5 -5 120.631 4.54119 3

-7 -5 5 114.084 4.59447 5

6 5 -5 461.619 14.5847 3

-6 -5 5 439.155 14.6342 5

-5 -5 5 267.360 9.37120 5

-5 -5 5 299.057 9.31741 2

4 5 -5 327.899 10.6111 5

-4 -5 5 321.530 10.5071 2

3 5 -5 114.035 3.95750 5

3 5 -5 115.279 4.00851 2

-3 -5 5 109.536 4.02486 5

-3 -5 5 122.301 4.23544 2

2 5 -5 32.8796 1.42982 5

2 5 -5 33.9910 1.59853 2

2 5 -5 37.9135 1.46167 6

2 5 -5 33.2267 1.99085 4

-2 -5 5 31.5458 1.62518 2

-2 -5 5 33.5872 1.51895 5

1 5 -5 1675.70 51.9325 5

1 5 -5 1641.37 52.0770 6

1 5 -5 1714.95 52.0372 1

1 5 -5 1569.82 52.4051 4

-1 -5 5 1722.37 52.2999 5

0 -5 5 248.608 7.97252 4

0 -5 5 233.781 7.72672 2

0 -5 5 227.783 7.58582 1

-1 5 -5 1224.76 38.6481 1

-1 5 -5 1230.34 38.8182 4

1 -5 5 1205.80 38.6235 1

1 -5 5 1282.93 38.8953 4

-2 5 -5 188.812 6.43438 1

-2 5 -5 197.483 6.67809 4

2 -5 5 182.267 6.17359 3

2 -5 5 192.692 6.74313 4

2 -5 5 195.960 6.40440 1

2 -5 5 192.340 6.21117 6

-3 5 -5 337.087 10.7349 1

-3 5 -5 307.935 11.3606 4

3 -5 5 319.893 10.4586 3

3 -5 5 325.506 11.0165 4

3 -5 5 339.043 10.6896 1

3 -5 5 337.411 10.4999 6

-4 5 -5 27.7526 2.44563 4

4 -5 5 33.6379 1.72560 3

4 -5 5 29.3208 1.64612 6

4 -5 5 32.0538 2.85712 1

-5 5 -5 320.951 11.2751 4

5 -5 5 307.833 10.6994 6

5 -5 5 318.397 10.8465 1

5 -5 5 366.316 11.5054 3

-6 5 -5 18.0129 2.92076 4

6 -5 5 26.4623 1.93936 3

6 -5 5 21.4944 2.00219 1

-7 5 -5 9.34057 2.57117 4

7 -5 5 7.26672 1.29758 1

7 -5 5 6.89361 1.12592 3

-8 5 -5 10.4117 2.93134 4

8 -5 5 6.42834 1.52436 1

8 -5 5 7.68111 1.40428 3

-9 5 -5 5.25474 3.23966 4

9 -5 5 4.08790 1.53321 3

9 -5 5 6.15221 1.65456 1

10 -5 5 -0.7606 1.82920 1

10 -5 5 1.31832 1.75117 3

11 -5 5 -0.1142 1.82872 1

11 -5 5 1.94188 2.29091 3

12 -5 5 -0.5868 2.24280 1

12 -5 5 -1.1219 2.67543 3

13 4 -5 -0.6767 2.53130 1

13 4 -5 -0.4992 2.65524 3

12 4 -5 15.1239 2.38065 1

12 4 -5 13.0338 2.30892 3

11 4 -5 0.61971 1.63911 1

11 4 -5 0.99809 1.73067 3

-11 -4 5 2.30091 2.05180 5

10 4 -5 0.73276 1.38463 1

10 4 -5 1.64050 1.18946 3

-10 -4 5 1.85588 1.68207 5

9 4 -5 22.9143 2.18899 1

9 4 -5 19.8928 2.40582 3

-9 -4 5 23.9991 2.40728 5

8 4 -5 152.410 5.79549 3

8 4 -5 154.254 5.41178 1

-8 -4 5 157.876 5.81641 5

7 4 -5 27.5878 1.64784 1

7 4 -5 29.6132 2.19115 3

-7 -4 5 24.9314 2.08220 5

6 4 -5 136.255 4.55572 1

6 4 -5 140.182 4.93681 3

-6 -4 5 129.178 4.94678 5

5 4 -5 60.9646 2.51153 3

-5 -4 5 59.6873 2.46583 2

-5 -4 5 52.7200 2.59853 5

4 4 -5 42.8269 1.87032 3

4 4 -5 39.4521 1.89834 5

4 4 -5 39.9315 1.85279 2

-4 -4 5 42.9612 2.07358 2

3 4 -5 1785.55 54.6526 2

3 4 -5 1767.01 54.6547 5

-3 -4 5 1654.70 55.3721 5

-3 -4 5 1806.22 55.0621 2

2 4 -5 1690.09 55.0287 4

2 4 -5 1743.42 54.7659 6

2 4 -5 1735.80 54.6571 5

2 4 -5 1805.04 54.5635 2

-2 -4 5 1731.00 55.0071 5

-2 -4 5 1746.88 54.9905 2

1 4 -5 174.084 6.08550 4

1 4 -5 175.734 5.81728 6

1 4 -5 182.530 5.77229 5

1 4 -5 182.774 5.74660 1

-1 -4 5 186.564 5.80216 5

0 4 -5 1.55004 0.43763 1

0 4 -5 1.37771 1.08488 4

0 -4 5 2.87938 0.54780 2

0 -4 5 1.22323 1.09758 4

0 -4 5 1.97067 0.40810 1

-1 4 -5 533.951 16.3379 1

-1 4 -5 500.492 16.1712 6

1 -4 5 528.551 16.5738 4

1 -4 5 510.847 16.2226 6

1 -4 5 509.601 16.3116 1

-2 4 -5 8.20021 1.11669 1

2 -4 5 7.65742 0.99648 1

2 -4 5 5.45241 0.74288 6

2 -4 5 2.93728 1.44016 4

2 -4 5 4.68641 0.68244 3

-3 4 -5 849.972 27.8766 1

3 -4 5 926.310 27.7575 6

3 -4 5 901.789 28.1494 4

3 -4 5 872.575 27.8387 1

3 -4 5 869.698 27.6392 3

-4 4 -5 11.4742 2.05018 4

-4 4 -5 11.6183 1.49777 1

4 -4 5 13.3506 1.17065 3

4 -4 5 13.2251 1.54773 1

4 -4 5 14.5853 1.28568 6

-5 4 -5 10.3245 2.07772 4

5 -4 5 6.85051 1.14599 6

-6 4 -5 1.05402 2.05564 4

6 -4 5 2.29052 0.79715 3

6 -4 5 2.48838 1.03209 1

-7 4 -5 63.1719 3.99903 4

7 -4 5 61.9229 3.17848 1

7 -4 5 66.5580 2.99024 3

-8 4 -5 -0.9928 2.46587 4

8 -4 5 -0.5974 1.11991 1

8 -4 5 -1.1679 0.95283 3

-9 4 -5 -1.1029 3.01067 4

9 -4 5 1.46196 1.25626 3

9 -4 5 2.68944 1.44865 1

10 -4 5 3.15498 1.79179 1

10 -4 5 1.82314 1.60304 3

11 -4 5 1.92601 2.13859 3

11 -4 5 5.48846 3.89071 1

12 -4 5 2.33427 2.14836 1

12 -4 5 -1.4639 2.56203 3

13 -4 5 2.42105 2.66191 1

13 -4 5 -2.3418 3.33765 3

13 3 -5 6.94893 2.99840 3

13 3 -5 5.20667 2.68869 1

12 3 -5 3.94268 2.08005 1

12 3 -5 0.01381 2.24314 3

11 3 -5 19.7114 3.09188 1

11 3 -5 22.2311 3.42455 3

10 3 -5 8.36772 1.67134 3

10 3 -5 9.31444 1.59444 1

-10 -3 5 14.3221 2.07129 5

9 3 -5 16.8573 2.26564 3

9 3 -5 16.1873 2.10870 1

-9 -3 5 17.8776 2.30874 5

-9 -3 5 13.6238 1.86299 1

8 3 -5 186.030 6.06681 1

8 3 -5 181.380 6.36162 3

-8 -3 5 178.239 6.31397 5

-8 -3 5 148.614 6.32727 1

7 3 -5 282.930 9.20307 1

7 3 -5 286.863 9.52437 3

-7 -3 5 278.009 9.50525 5

6 3 -5 72.0042 2.78476 1

6 3 -5 70.0182 3.03159 3

-6 -3 5 67.4739 2.56134 1

5 3 -5 250.917 8.13997 2

5 3 -5 261.354 7.99031 1

-5 -3 5 236.486 8.26211 5

4 3 -5 25.7993 1.48687 5

4 3 -5 33.7302 1.58219 3

4 3 -5 26.1720 1.42922 2

-4 -3 5 27.2613 1.44864 2

3 3 -5 129.912 4.41344 5

3 3 -5 140.840 4.43140 3

3 3 -5 136.073 4.40296 2

-3 -3 5 122.302 4.45789 5

-3 -3 5 130.217 4.41334 2

2 3 -5 381.365 11.9850 3

2 3 -5 391.518 12.0361 2

2 3 -5 399.228 12.0650 6

2 3 -5 372.878 12.0140 5

-2 -3 5 369.920 12.0459 5

-2 -3 5 367.492 12.0337 2

1 3 -5 73.3008 2.56085 6

1 3 -5 67.7861 2.42253 1

1 3 -5 72.4536 2.44040 5

1 3 -5 72.4873 2.89118 4

-1 -3 5 78.8923 2.50406 6

-1 -3 5 70.1873 2.55438 2

0 3 -5 9.73712 1.31638 4

0 3 -5 10.0223 0.73843 1

0 3 -5 8.48949 0.87477 6

0 -3 5 12.5888 0.85917 6

0 -3 5 10.9875 0.68574 1

0 -3 5 11.9255 1.37206 4

-1 3 -5 544.528 16.9778 1

-1 3 -5 530.934 17.1682 4

-1 3 -5 532.465 16.8988 6

1 -3 5 559.707 16.9554 6

1 -3 5 546.684 17.2067 4

1 -3 5 520.080 16.9394 1

-2 3 -5 3378.20 102.492 1

-2 3 -5 3221.43 103.168 4

2 -3 5 3158.95 102.722 1

2 -3 5 3216.65 102.097 3

2 -3 5 3261.51 102.686 4

2 -3 5 3399.02 102.480 6

-3 3 -5 562.555 17.9758 1

-3 3 -5 567.688 18.1758 4

3 -3 5 582.089 18.2655 4

3 -3 5 528.714 17.8943 1

3 -3 5 569.626 17.7574 3

3 -3 5 581.467 17.8963 6

-4 3 -5 157.221 6.47839 4

-4 3 -5 163.317 5.83931 1

4 -3 5 174.411 5.74831 6

4 -3 5 176.435 5.59121 3

-5 3 -5 191.411 6.80678 4

5 -3 5 176.283 6.25757 6

5 -3 5 177.522 6.34803 1

5 -3 5 185.274 5.99579 5

-6 3 -5 287.315 9.25984 5

-6 3 -5 288.708 10.1533 4

6 -3 5 287.791 9.34479 5

6 -3 5 288.326 9.69008 1

6 -3 5 291.954 9.49665 3

-7 3 -5 16.9200 1.37658 5

-7 3 -5 16.0328 2.86568 4

7 -3 5 15.4818 2.02693 1

7 -3 5 15.4013 1.66758 5

7 -3 5 15.1638 1.58655 3

-8 3 -5 0.40518 0.97755 5

-8 3 -5 -0.6636 2.46056 4

8 -3 5 0.51851 1.14945 1

8 -3 5 0.69687 0.92804 3

-9 3 -5 0.99835 1.21740 5

-9 3 -5 -0.4085 2.88446 4

9 -3 5 0.23048 1.09139 3

9 -3 5 -0.6278 1.24757 1

10 -3 5 8.84030 1.97095 1

10 -3 5 6.26852 1.56614 3

11 -3 5 -1.6821 3.17019 1

11 -3 5 -0.2889 1.96185 3

12 -3 5 1.38489 2.63297 3

12 -3 5 1.79116 2.52208 1

13 -3 5 -2.4855 2.37758 1

13 -3 5 -6.5090 2.99279 3

14 2 -5 -1.2966 2.95826 3

13 2 -5 -1.9885 2.44358 1

13 2 -5 -0.3338 2.33760 3

12 2 -5 -0.2401 2.04298 1

11 2 -5 3.91290 1.73945 1

11 2 -5 5.62766 1.91110 3

10 2 -5 7.73751 1.60340 3

10 2 -5 5.67698 1.60536 1

-10 -2 5 7.60662 1.42771 1

9 2 -5 97.9582 4.09950 1

9 2 -5 102.018 4.32357 3

-9 -2 5 92.3405 3.90958 1

8 2 -5 53.3707 2.61825 1

8 2 -5 50.1525 2.79735 3

-8 -2 5 52.4480 2.87133 5

-8 -2 5 51.2815 2.52512 1

7 2 -5 130.644 4.54165 1

7 2 -5 129.084 4.81065 3

-7 -2 5 125.503 4.47277 1

-7 -2 5 123.534 4.74878 5

6 2 -5 300.687 9.54125 3

-6 -2 5 279.088 9.51118 5

-6 -2 5 277.837 9.18178 1

-6 -2 5 290.884 9.23654 1

5 2 -5 495.728 15.4834 1

5 2 -5 514.949 15.5676 2

-5 -2 5 463.049 15.3941 1

-5 -2 5 488.492 16.0705 5

4 2 -5 130.217 4.28256 3

4 2 -5 127.166 4.15635 2

4 2 -5 128.405 4.22955 5

-4 -2 5 109.649 4.24751 5

-4 -2 5 120.729 4.00109 1

-4 -2 5 121.883 4.15571 2

3 2 -5 89.5420 3.10712 6

3 2 -5 86.1671 2.87697 1

3 2 -5 86.1603 3.01556 5

3 2 -5 87.2761 3.05475 3

3 2 -5 88.2528 2.97443 2

-3 -2 5 85.9167 2.98859 2

2 2 -5 2.73132 0.46669 2

2 2 -5 1.89261 0.39470 5

2 2 -5 2.95149 0.44142 1

2 2 -5 2.38418 0.38016 3

2 2 -5 3.88186 0.63304 6

-2 -2 5 1.29410 0.43628 2

1 2 -5 747.333 24.2824 4

1 2 -5 792.333 24.3742 6

1 2 -5 796.680 24.2134 5

1 2 -5 767.418 24.0571 1

-1 -2 5 773.205 24.1696 1

-1 -2 5 737.230 24.0813 2

-1 -2 5 784.183 24.1203 6

0 2 -5 7218.76 229.691 4

0 2 -5 7223.61 229.003 6

0 -2 5 7521.94 229.669 4

0 -2 5 7424.07 228.920 6

-1 2 -5 224.568 7.22172 6

-1 2 -5 219.145 7.46520 4

-1 2 -5 228.938 7.25195 1

1 -2 5 231.249 7.50647 4

1 -2 5 215.010 7.19926 1

1 -2 5 233.190 7.28025 6

-2 2 -5 139.230 4.56914 6

-2 2 -5 134.205 4.88383 4

-2 2 -5 141.585 4.65100 1

2 -2 5 133.670 4.58238 1

2 -2 5 143.621 4.64734 6

2 -2 5 138.693 4.93762 4

2 -2 5 137.175 4.44938 3

-3 2 -5 98.4714 3.29001 5

-3 2 -5 106.233 3.63262 1

-3 2 -5 97.6057 3.90013 4

3 -2 5 99.8307 3.99640 4

3 -2 5 99.0285 3.51940 1

3 -2 5 100.178 3.57071 6

3 -2 5 102.342 3.39023 3

-4 2 -5 954.373 30.0959 5

-4 2 -5 943.671 31.0902 4

4 -2 5 989.010 30.1580 5

4 -2 5 972.995 30.6820 4

4 -2 5 972.630 30.3859 6

4 -2 5 956.787 30.2079 3

-5 2 -5 4.38030 1.75887 4

-5 2 -5 3.39713 0.68744 5

5 -2 5 4.24701 0.84937 5

5 -2 5 5.03951 0.91161 1

-6 2 -5 7.24732 1.90438 4

6 -2 5 8.67450 1.10084 5

6 -2 5 4.95261 0.87371 3

6 -2 5 5.10737 1.07846 1

-7 2 -5 51.5793 2.33869 5

-7 2 -5 51.3305 3.54193 4

7 -2 5 50.2069 2.77383 1

7 -2 5 51.6393 2.52510 3

-8 2 -5 -0.7189 2.30959 4

-8 2 -5 0.34319 1.00322 5

8 -2 5 0.12327 0.85577 3

8 -2 5 -2.0101 1.10167 1

-9 2 -5 -0.0615 1.15751 5

-9 2 -5 0.94991 2.68049 4

9 -2 5 0.16929 1.01514 3

9 -2 5 1.33470 1.35087 1

-10 2 -5 1.01405 1.41153 5

10 -2 5 1.61391 1.59342 1

10 -2 5 -0.4859 1.19429 3

-11 2 -5 1.82155 1.74757 5

11 -2 5 1.39547 1.68523 3

11 -2 5 4.37056 2.14352 1

12 -2 5 -0.0311 2.51823 1

12 -2 5 -2.5581 2.33658 3

13 -2 5 -4.7183 3.04801 3

13 -2 5 0.51772 2.34408 1

14 1 -5 -0.0832 3.36145 3

13 1 -5 0.25697 2.64559 1

13 1 -5 -1.0625 2.64177 3

12 1 -5 1.70167 2.14744 1

12 1 -5 5.20818 3.52248 3

11 1 -5 6.00702 1.87102 1

11 1 -5 4.56861 1.77468 3

10 1 -5 8.79056 1.60894 1

10 1 -5 7.56056 1.45050 3

-10 -1 5 8.86674 1.54506 1

9 1 -5 1.04253 1.17592 1

9 1 -5 2.23171 1.15814 3

-9 -1 5 1.85175 1.18051 1

8 1 -5 2.39670 1.05879 3

8 1 -5 4.43537 1.07985 1

-8 -1 5 4.82732 1.08941 1

7 1 -5 4.33939 1.06617 3

7 1 -5 4.25376 1.00249 1

-7 -1 5 5.20138 1.00026 1

6 1 -5 31.1729 1.76228 2

6 1 -5 33.2669 2.02302 3

6 1 -5 29.8264 1.69827 1

-6 -1 5 28.2887 1.54529 1

-6 -1 5 30.8486 1.79812 1

5 1 -5 90.3042 3.54924 5

5 1 -5 105.994 3.46720 2

5 1 -5 101.312 3.46669 1

-5 -1 5 95.8693 3.44242 1

-5 -1 5 97.3254 3.42799 1

4 1 -5 324.119 9.98862 2

4 1 -5 308.047 10.1312 3

4 1 -5 308.584 10.0690 5

-4 -1 5 314.943 10.0721 3

-4 -1 5 312.419 9.95244 1

-4 -1 5 315.236 9.97358 1

3 1 -5 434.876 14.0772 3

3 1 -5 456.432 13.9866 2

3 1 -5 438.526 14.0451 5

3 1 -5 444.456 14.1540 6

3 1 -5 449.923 13.9753 1

-3 -1 5 460.777 13.9532 1

-3 -1 5 439.048 14.0356 3

-3 -1 5 429.901 13.9777 2

-3 -1 5 451.271 13.9885 1

2 1 -5 2235.53 67.4273 1

2 1 -5 2132.88 67.6727 3

2 1 -5 2226.15 67.4227 2

2 1 -5 2181.02 67.9493 6

2 1 -5 2162.05 67.5653 5

-2 -1 5 2070.12 67.3430 1

-2 -1 5 2110.58 67.3710 2

-2 -1 5 2127.46 67.5243 3

1 1 -5 155.652 4.99900 3

1 1 -5 153.131 5.31629 4

1 1 -5 159.649 5.07467 1

1 1 -5 158.900 5.16699 6

-1 -1 5 165.714 5.05443 1

-1 -1 5 159.089 5.00933 2

-1 -1 5 133.545 4.98296 3

-1 -1 5 166.277 5.12837 6

0 1 -5 2482.83 80.3962 4

0 1 -5 2474.58 79.9733 6

0 1 -5 2565.88 79.8070 1

0 -1 5 2532.00 79.9310 4

0 -1 5 2613.38 79.4751 3

0 -1 5 2614.16 79.9566 6

0 -1 5 2566.56 79.8362 1

-1 1 -5 1780.09 57.2223 4

-1 1 -5 1779.62 57.3156 6

-1 1 -5 1822.10 57.2065 1

1 -1 5 1783.54 57.2646 1

1 -1 5 1844.56 57.4878 4

1 -1 5 1909.97 57.3970 6

-2 1 -5 1196.42 39.0144 1

-2 1 -5 1228.81 39.1154 5

-2 1 -5 1287.15 39.8132 4

-2 1 -5 1253.95 39.3398 6

2 -1 5 1318.79 39.4748 6

2 -1 5 1209.37 39.2101 4

2 -1 5 1254.74 39.6604 1

-3 1 -5 184.405 6.08562 5

-3 1 -5 195.956 6.31448 1

-3 1 -5 188.196 6.54958 4

3 -1 5 183.192 6.21980 1

3 -1 5 190.859 6.61966 4

3 -1 5 197.173 6.34080 6

-4 1 -5 406.483 13.3351 5

-4 1 -5 426.471 13.6064 1

4 -1 5 439.063 13.4260 5

4 -1 5 426.901 13.9119 4

4 -1 5 415.812 13.6142 6

-5 1 -5 1.67790 1.74426 4

-5 1 -5 2.94662 0.74639 5

5 -1 5 3.44840 0.84523 1

5 -1 5 4.92509 0.90959 5

5 -1 5 1.71293 0.60563 3

-6 1 -5 46.8132 3.17868 4

-6 1 -5 55.2317 2.37308 5

6 -1 5 50.7215 2.35192 3

6 -1 5 48.1044 2.38839 5

6 -1 5 52.2860 2.46832 1

-7 1 -5 156.309 5.20911 5

-7 1 -5 146.945 6.09335 4

7 -1 5 145.414 5.51831 1

7 -1 5 145.552 5.32688 3

-8 1 -5 9.03861 1.39269 5

-8 1 -5 7.31964 2.51829 4

8 -1 5 4.40086 0.96453 3

8 -1 5 4.71074 1.31525 1

-9 1 -5 39.4626 2.41949 5

9 -1 5 32.3334 2.41401 3

9 -1 5 35.4553 2.94365 1

-10 1 -5 18.9270 2.48456 5

10 -1 5 15.1566 2.11804 1

10 -1 5 15.5888 2.27997 3

-11 1 -5 7.79566 1.93685 5

11 -1 5 3.65104 1.80988 3

11 -1 5 5.15367 2.01107 1

-12 1 -5 1.56391 2.30596 5

12 -1 5 1.20537 2.40186 1

12 -1 5 1.30497 2.06793 3

13 -1 5 -0.3757 3.11635 3

13 -1 5 8.37045 3.18912 1

14 0 -5 -3.4683 3.12606 3

13 0 -5 0.07653 2.40708 3

13 0 -5 2.52510 2.78220 1

12 0 -5 -5.2195 4.10460 3

12 0 -5 1.35227 2.04276 1

11 0 -5 0.15198 1.71833 3

11 0 -5 1.73394 1.74874 1

-11 0 5 -0.7533 1.61135 1

10 0 -5 -0.9626 1.37775 1

10 0 -5 -0.7318 1.21463 3

-10 0 5 -0.8307 1.39880 1

9 0 -5 0.39811 1.18011 3

9 0 -5 0.30862 1.21636 1

-9 0 5 -0.7091 1.27675 1

8 0 -5 0.39320 0.95116 3

8 0 -5 0.22021 0.99158 1

-8 0 5 -0.5509 1.05577 1

7 0 -5 1.64836 0.95263 3

6 0 -5 1.91102 0.79180 3

6 0 -5 -1.0994 0.83090 1

6 0 -5 1.61785 0.78231 2

-6 0 5 0.89324 0.87639 1

5 0 -5 1.02744 0.65045 5

5 0 -5 1.91216 0.74994 3

5 0 -5 0.93471 0.58821 2

-5 0 5 0.83593 0.56961 1

-5 0 5 1.26939 0.58494 3

-5 0 5 0.83161 0.68746 1

4 0 -5 1.16291 0.44632 2

4 0 -5 0.50617 0.48036 5

4 0 -5 0.06999 0.60597 3

-4 0 5 0.39956 0.40395 1

-4 0 5 0.04425 0.58388 1

3 0 -5 3.31570 0.44659 2

3 0 -5 2.79863 0.54003 1

3 0 -5 3.16966 0.60704 6

-3 0 5 3.65382 0.44424 1

-3 0 5 3.13875 0.61602 1

2 0 -5 4.45018 0.49778 2

2 0 -5 5.52450 0.80126 3

2 0 -5 4.67383 0.78382 6

-2 0 5 4.53423 0.63319 3

-2 0 5 5.98110 0.86187 1

-2 0 5 4.74822 0.67588 6

-2 0 5 4.02260 0.50203 2

1 0 -5 0.79422 0.38764 3

1 0 -5 0.61034 0.51460 1

1 0 -5 1.38807 0.72004 6

-1 0 5 1.05147 0.27292 2

-1 0 5 0.54389 0.53261 6

-1 0 5 0.66362 0.30072 3

-1 0 5 0.85700 0.46967 1

0 0 -5 1.33346 0.95188 4

0 0 -5 0.28235 0.64693 6

0 0 -5 0.87867 0.47075 1

0 0 5 0.26528 0.33544 3

0 0 5 1.05015 0.84520 4

0 0 5 1.01167 0.58860 6

0 0 5 1.40464 0.46007 1

-1 0 -5 1.18173 0.51107 1

-1 0 -5 -0.6674 0.91067 4

-1 0 -5 -0.6332 0.52378 6

1 0 5 0.44923 0.88903 4

1 0 5 0.83059 0.61394 6

1 0 5 0.35421 0.43626 1

-2 0 -5 1.21393 0.50127 6

-2 0 -5 1.13644 0.49340 5

-2 0 -5 1.33988 0.53474 1

-2 0 -5 1.49652 1.02093 4

2 0 5 0.57885 0.53637 6

2 0 5 0.66441 0.45385 1

2 0 5 1.46857 1.02073 4

-3 0 -5 -1.6879 1.03745 4

-3 0 -5 1.32231 0.60562 1

3 0 5 -0.8298 1.14442 4

3 0 5 -0.0243 0.66691 6

3 0 5 -0.0079 0.49232 1

-4 0 -5 1.19409 0.76878 1

-4 0 -5 1.42965 0.61147 5

-4 0 -5 2.31380 1.28504 4

4 0 5 0.81148 0.77582 6

4 0 5 3.33406 0.75623 5

4 0 5 1.32482 1.37467 4

-5 0 -5 0.03562 1.55815 4

-5 0 -5 0.83512 0.75473 5

5 0 5 2.60757 0.95115 5

5 0 5 1.20652 0.72953 1

-6 0 -5 3.48912 1.90087 4

-6 0 -5 0.19615 1.00186 5

6 0 5 1.64735 0.69305 3

6 0 5 1.60366 0.84227 1

-7 0 -5 -1.4434 0.91458 5

-7 0 -5 1.83394 2.01363 4

7 0 5 -0.0384 0.92556 1

7 0 5 -1.0721 0.74116 3

-8 0 -5 0.53959 2.25824 4

-8 0 -5 -0.8753 1.11601 5

8 0 5 0.49320 1.16635 1

8 0 5 0.35549 0.88481 3

-9 0 -5 -1.1158 1.30830 5

9 0 5 -1.0010 0.97800 3

9 0 5 -1.5201 1.32764 1

-10 0 -5 1.58189 1.47620 5

10 0 5 0.16286 1.04472 3

10 0 5 0.03507 1.57731 1

-11 0 -5 -1.5039 1.76388 5

11 0 5 -0.2490 1.88433 1

11 0 5 -1.7029 1.13331 3

-12 0 -5 -2.6552 2.26353 5

12 0 5 -2.2846 2.07252 3

12 0 5 -0.6836 2.43258 1

13 0 5 -1.4655 2.80179 3

13 0 5 1.12988 3.03104 1

14 -1 -5 0.90416 2.80676 3

14 -1 -5 6.39834 3.35838 6

13 -1 -5 -2.2469 2.82820 3

13 -1 -5 2.11477 2.66851 1

12 -1 -5 0.17292 2.05882 1

11 -1 -5 3.25002 1.69992 3

11 -1 -5 3.98432 1.83982 1

-11 1 5 2.47290 1.80733 1

10 -1 -5 6.19321 1.67087 1

10 -1 -5 7.91011 1.56050 3

-10 1 5 8.15833 2.40903 1

9 -1 -5 2.19750 1.30681 1

9 -1 -5 1.44740 1.28312 3

-9 1 5 1.70600 1.43820 1

8 -1 -5 1.88075 1.05878 3

8 -1 -5 2.87773 1.17178 1

-8 1 5 3.63079 1.28814 1

7 -1 -5 4.99425 1.03457 3

7 -1 -5 6.55832 1.18201 1

-7 1 5 5.43473 1.21979 1

6 -1 -5 24.3073 1.50912 2

6 -1 -5 26.6546 1.99994 3

6 -1 -5 23.9439 1.80271 1

-6 1 5 20.9612 1.88636 1

5 -1 -5 87.3136 3.72304 3

5 -1 -5 100.128 3.48021 1

5 -1 -5 94.9413 3.22599 2

-5 1 5 89.0512 3.39697 3

-5 1 5 94.3487 3.45731 1

-5 1 5 90.3601 3.30777 1

4 -1 -5 324.889 10.7614 3

4 -1 -5 324.847 10.6252 5

4 -1 -5 343.852 10.5292 2

4 -1 -5 331.513 10.8330 6

-4 1 5 335.873 10.6976 3

-4 1 5 338.449 10.6185 1

-4 1 5 331.856 10.6718 1

3 -1 -5 410.151 12.8108 2

3 -1 -5 418.460 12.9455 1

3 -1 -5 408.254 13.0723 6

3 -1 -5 394.400 13.0117 3

-3 1 5 414.818 12.9248 1

-3 1 5 414.295 12.9655 3

-3 1 5 403.048 12.9737 1

2 -1 -5 2100.64 67.2340 6

2 -1 -5 2179.19 66.5782 1

2 -1 -5 2078.91 67.0425 3

-2 1 5 2111.55 66.7708 3

-2 1 5 2273.06 67.0507 6

-2 1 5 1998.35 66.5418 1

1 -1 -5 191.382 6.49643 3

1 -1 -5 207.975 6.52442 1

1 -1 -5 199.579 6.60685 6

-1 1 5 206.944 6.56069 6

-1 1 5 197.360 6.47619 3

-1 1 5 204.992 6.54388 1

0 -1 -5 2844.04 87.4526 1

0 -1 -5 2674.85 87.3748 3

0 -1 -5 2728.59 87.7329 4

0 -1 -5 2780.23 87.7477 6

0 1 5 2790.22 87.5682 1

0 1 5 2882.14 87.6493 6

0 1 5 2817.37 87.4331 3

-1 -1 -5 1886.27 60.9265 6

-1 -1 -5 1935.21 60.7046 1

-1 -1 -5 1898.91 60.9054 4

1 1 5 1955.75 60.6584 1

1 1 5 1962.52 60.8912 4

1 1 5 1918.05 60.6687 6

-2 -1 -5 1248.27 41.7377 1

-2 -1 -5 1389.94 42.6115 6

-2 -1 -5 1321.58 42.0483 5

-2 -1 -5 1362.98 42.4282 4

2 1 5 1300.32 41.8097 6

2 1 5 1323.78 42.2220 1

2 1 5 1415.52 42.5059 4

-3 -1 -5 202.383 6.66941 5

-3 -1 -5 205.346 6.94145 4

-3 -1 -5 210.463 6.79267 1

3 1 5 206.424 7.02474 4

3 1 5 202.602 6.74234 1

3 1 5 207.933 6.86682 6

-4 -1 -5 499.133 15.9508 4

-4 -1 -5 487.467 15.6190 5

-4 -1 -5 491.621 15.7631 1

4 1 5 502.235 15.7090 5

4 1 5 510.692 16.0522 4

4 1 5 461.886 15.8354 6

4 1 5 504.504 15.8629 1

-5 -1 -5 3.33648 0.89334 5

5 1 5 3.48079 0.80420 1

-6 -1 -5 50.3302 2.28995 5

-6 -1 -5 50.0133 3.09181 4

6 1 5 49.8774 2.41060 1

-7 -1 -5 146.177 5.15802 5

-7 -1 -5 143.865 5.85153 4

7 1 5 141.527 5.22970 1

-8 -1 -5 8.71763 1.39778 5

-8 -1 -5 9.47280 2.62037 4

8 1 5 6.69906 1.25032 1

8 1 5 6.29807 1.07497 3

-9 -1 -5 42.0212 2.75206 5

9 1 5 35.3872 2.51028 3

9 1 5 38.6672 2.95843 1

-10 -1 -5 23.2249 2.73627 5

10 1 5 18.4781 2.43973 3

10 1 5 16.8175 2.13478 1

-11 -1 -5 5.88501 2.08486 5

11 1 5 3.87311 2.06536 1

11 1 5 3.05201 1.31764 3

-12 -1 -5 -0.3195 2.36991 5

12 1 5 -0.3807 2.41817 1

12 1 5 0.72591 1.44695 3

-13 -1 -5 3.70345 3.00015 5

13 1 5 0.61746 2.88169 3

13 1 5 2.18886 3.02070 1

14 -2 -5 -5.2481 3.01547 3

14 -2 -5 1.27067 3.59435 6

13 -2 -5 -4.3425 2.65842 1

13 -2 -5 -3.0887 2.42443 3

12 -2 -5 0.52429 2.56131 3

-12 2 5 2.37953 2.69921 1

11 -2 -5 5.44349 1.96192 3

11 -2 -5 5.51463 1.92667 1

-11 2 5 8.20805 2.00935 1

10 -2 -5 4.59594 1.71110 1

10 -2 -5 7.54681 1.64989 3

-10 2 5 3.90113 1.90095 1

9 -2 -5 94.4781 4.49121 1

9 -2 -5 107.895 4.63219 3

8 -2 -5 56.9220 3.18268 1

8 -2 -5 66.3037 3.24991 3

-8 2 5 60.9015 3.65506 1

7 -2 -5 121.415 4.72778 1

7 -2 -5 121.657 4.74225 3

-7 2 5 121.971 4.73221 1

6 -2 -5 307.561 10.2347 3

6 -2 -5 309.544 10.2221 1

-6 2 5 304.876 10.1643 1

5 -2 -5 512.626 16.7434 5

-5 2 5 545.196 16.8745 1

4 -2 -5 91.9179 3.40781 3

4 -2 -5 87.3976 3.40137 6

-4 2 5 90.1019 3.23427 3

-4 2 5 90.8607 3.16440 1

-4 2 5 87.6902 3.32036 1

3 -2 -5 87.4968 3.31190 3

3 -2 -5 90.0059 3.29628 6

3 -2 -5 93.8323 3.17436 1

-3 2 5 90.7801 3.12989 1

-3 2 5 88.1359 3.25367 1

-3 2 5 93.0768 3.16996 3

2 -2 -5 4.31538 0.73863 6

2 -2 -5 2.27063 0.58869 3

2 -2 -5 2.26756 0.55733 1

-2 2 5 1.23676 0.63492 1

-2 2 5 2.42007 0.49417 6

1 -2 -5 739.198 24.1439 6

1 -2 -5 796.849 24.3517 3

1 -2 -5 765.300 24.0966 1

-1 2 5 768.250 24.0874 3

-1 2 5 754.181 24.1145 1

-1 2 5 787.697 24.1310 6

0 -2 -5 7767.69 245.103 6

0 -2 -5 7814.86 244.892 4

0 -2 -5 7844.10 244.761 1

0 2 5 7809.45 244.941 1

0 2 5 7914.82 244.742 3

0 2 5 7929.27 245.007 6

-1 -2 -5 210.501 7.03079 6

-1 -2 -5 210.434 7.05429 4

-1 -2 -5 213.278 7.01981 1

1 2 5 219.422 6.97077 3

1 2 5 220.569 7.05426 4

1 2 5 212.318 7.03166 1

1 2 5 223.332 7.08423 6

-2 -2 -5 182.426 6.00219 6

-2 -2 -5 179.396 6.04095 4

-2 -2 -5 182.591 5.98781 1

-2 -2 -5 177.502 5.91014 5

2 2 5 179.258 5.98401 1

2 2 5 182.290 6.05452 4

2 2 5 182.297 6.03814 6

-3 -2 -5 103.085 3.63357 1

-3 -2 -5 101.681 3.74841 4

-3 -2 -5 101.183 3.52593 5

3 2 5 99.5371 3.57611 1

3 2 5 101.909 3.84452 4

3 2 5 100.323 3.68165 6

-4 -2 -5 1041.60 31.8992 5

-4 -2 -5 1022.28 32.1014 4

-4 -2 -5 1024.08 31.9853 1

4 2 5 1058.45 32.2185 4

4 2 5 954.353 32.0862 6

4 2 5 980.585 32.0873 1

4 2 5 1043.12 31.9816 5

-5 -2 -5 3.82018 1.49996 4

-5 -2 -5 3.20713 0.86792 5

5 2 5 4.86619 1.68491 4

5 2 5 2.58753 0.81133 1

-6 -2 -5 13.5309 2.22058 4

-6 -2 -5 12.5514 1.60306 5

6 2 5 10.0334 1.49962 1

-7 -2 -5 54.5172 3.46746 4

-7 -2 -5 59.3354 2.77702 5

7 2 5 59.8547 2.79611 1

-8 -2 -5 0.76545 1.21871 5

8 2 5 0.84246 0.85406 3

8 2 5 1.67348 1.07322 1

-9 -2 -5 1.19010 1.44946 5

9 2 5 -1.0540 1.25298 1

9 2 5 0.17253 1.06559 3

-10 -2 -5 -0.7538 1.75444 5

10 2 5 1.35826 1.65881 1

10 2 5 0.56678 1.20259 3

-11 -2 -5 4.46590 2.13710 5

11 2 5 1.64100 1.27484 3

11 2 5 1.44813 2.05386 1

-12 -2 -5 -3.7256 3.87709 5

12 2 5 0.33332 1.33836 3

12 2 5 -0.1021 2.41609 1

-13 -2 -5 2.21596 3.01993 5

13 2 5 -0.7411 2.91703 1

13 2 5 -0.5998 1.56667 3

13 -3 -5 4.54901 3.15691 6

13 -3 -5 1.93315 2.77359 1

13 -3 -5 2.64893 2.78155 3

12 -3 -5 1.37308 2.09868 3

12 -3 -5 1.97084 2.42243 1

-12 3 5 5.22997 2.40413 1

11 -3 -5 9.58099 2.18404 1

11 -3 -5 12.2034 2.27702 3

-11 3 5 13.0032 2.29786 1

10 -3 -5 9.05704 1.80907 3

10 -3 -5 6.44489 1.79004 1

-10 3 5 10.8005 2.06487 1

9 -3 -5 10.4055 1.74983 1

9 -3 -5 11.3536 1.54682 3

-9 3 5 11.9997 1.96758 1

8 -3 -5 167.084 6.25696 3

8 -3 -5 154.869 6.24587 1

-8 3 5 170.496 6.29879 1

7 -3 -5 245.505 8.99967 1

7 -3 -5 266.338 9.03308 3

-7 3 5 273.533 9.52840 1

6 -3 -5 68.0715 3.08724 3

6 -3 -5 60.7796 3.01478 1

-6 3 5 60.9502 3.11589 1

5 -3 -5 221.495 7.53016 3

5 -3 -5 215.794 7.21723 5

-5 3 5 224.134 7.46145 1

4 -3 -5 23.7160 2.06688 3

4 -3 -5 27.4030 1.67603 6

-4 3 5 27.1385 1.47101 3

-4 3 5 23.9431 1.73528 1

-4 3 5 26.7581 1.41817 1

3 -3 -5 119.425 4.04523 1

3 -3 -5 114.238 4.13268 6

3 -3 -5 115.071 4.20412 3

-3 3 5 121.239 4.04904 3

-3 3 5 112.783 4.13256 1

-3 3 5 120.212 4.00676 1

2 -3 -5 414.220 13.6681 6

2 -3 -5 435.745 13.6060 1

2 -3 -5 419.494 13.6785 3

-2 3 5 440.429 13.6081 6

-2 3 5 432.077 13.6560 1

-2 3 5 429.809 13.6222 3

1 -3 -5 60.4902 2.43824 6

1 -3 -5 59.8179 2.29772 1

1 -3 -5 58.3294 2.33931 3

-1 3 5 62.7880 2.34003 6

-1 3 5 63.2303 2.35245 3

-1 3 5 58.7175 2.42631 1

0 -3 -5 6.54824 0.91618 1

0 -3 -5 6.07088 1.11316 4

0 -3 -5 7.35997 0.91442 6

0 -3 -5 5.61939 0.82980 3

0 3 5 8.53796 1.06542 6

0 3 5 8.96118 0.91504 3

0 3 5 6.89938 0.45538 2

0 3 5 7.32796 1.11033 1

-1 -3 -5 596.721 18.6109 1

-1 -3 -5 577.046 18.6157 6

-1 -3 -5 586.669 18.5602 4

1 3 5 580.213 18.4205 2

1 3 5 610.506 18.6653 6

1 3 5 588.725 18.5510 4

1 3 5 592.500 18.5906 3

1 3 5 584.201 18.6321 1

-2 -3 -5 3778.06 118.235 4

-2 -3 -5 3769.84 118.298 5

-2 -3 -5 3767.50 117.809 1

-2 -3 -5 3745.64 118.714 6

2 3 5 3820.21 118.376 6

2 3 5 3761.89 118.015 4

2 3 5 3760.39 118.278 1

-3 -3 -5 652.552 20.8090 5

-3 -3 -5 669.472 20.8787 1

-3 -3 -5 654.734 20.8902 4

3 3 5 640.580 20.8449 1

3 3 5 653.318 20.9504 6

3 3 5 690.018 20.9649 4

-4 -3 -5 189.182 6.49910 4

4 3 5 188.076 6.63785 4

-5 -3 -5 208.039 6.97393 5

-5 -3 -5 217.416 8.34636 4

5 3 5 201.154 7.42030 4

5 3 5 206.527 6.97509 1

-6 -3 -5 330.098 11.0654 4

-6 -3 -5 330.320 10.7588 5

6 3 5 324.979 10.7434 1

-7 -3 -5 23.2449 2.65014 4

-7 -3 -5 23.1600 1.94096 5

7 3 5 23.7046 1.96665 1

-8 -3 -5 -0.0086 1.23715 5

8 3 5 0.21214 1.09072 1

-9 -3 -5 1.17620 1.52833 5

9 3 5 0.33376 1.18412 1

9 3 5 -0.3757 0.84255 3

-10 -3 -5 7.99618 1.95834 5

10 3 5 5.08745 1.34393 3

10 3 5 5.85718 1.85401 1

-11 -3 -5 -0.2182 2.12721 5

11 3 5 -0.1662 1.37565 3

11 3 5 2.71663 2.10591 1

-12 -3 -5 2.53102 2.58954 5

12 3 5 -0.9214 1.48554 3

12 3 5 2.58246 2.55152 1

-13 -3 -5 -2.3604 3.01333 5

13 3 5 -1.1526 1.73015 3

13 3 5 2.12101 3.01187 1

13 -4 -5 -2.9969 2.51173 3

13 -4 -5 -1.4631 3.03941 6

12 -4 -5 8.13247 2.38053 3

12 -4 -5 9.62696 2.63108 1

-12 4 5 10.1964 2.70647 1

11 -4 -5 0.16083 1.74796 3

11 -4 -5 2.43254 2.04204 1

11 -4 -5 1.88834 2.39558 6

-11 4 5 2.22626 2.26634 1

10 -4 -5 -0.4444 1.70130 1

10 -4 -5 2.37358 1.89613 6

10 -4 -5 1.89246 1.54714 3

-10 4 5 2.49457 1.97180 1

9 -4 -5 29.4870 3.05448 3

9 -4 -5 22.6604 2.82265 1

9 -4 -5 26.3334 2.92238 6

-9 4 5 22.5324 3.30010 1

8 -4 -5 129.851 5.60638 1

8 -4 -5 129.445 5.67184 6

8 -4 -5 147.296 5.64795 3

-8 4 5 148.668 5.75878 1

7 -4 -5 21.3444 2.36058 1

7 -4 -5 21.5411 2.34697 6

7 -4 -5 24.5516 2.47610 3

-7 4 5 24.1248 2.65346 1

6 -4 -5 128.507 5.12190 6

6 -4 -5 135.519 5.13779 1

6 -4 -5 135.178 5.15876 3

5 -4 -5 81.3703 3.54037 3

-5 4 5 78.2165 3.45712 1

4 -4 -5 54.8871 2.53993 6

-4 4 5 53.3246 2.60840 1

-4 4 5 56.4221 2.24529 1

-4 4 5 56.7788 2.36666 3

3 -4 -5 1777.69 54.5157 3

3 -4 -5 1727.76 53.5004 1

3 -4 -5 1741.79 54.1734 6

-3 4 5 1697.02 53.4871 1

-3 4 5 1654.41 53.5569 1

-3 4 5 1688.35 53.5123 3

2 -4 -5 1785.88 53.9609 6

2 -4 -5 1770.21 53.4223 1

2 -4 -5 1693.58 53.4489 3

-2 4 5 1672.66 53.1173 3

-2 4 5 1582.07 53.1279 1

-2 4 5 1709.16 53.0760 6

1 -4 -5 184.873 5.91078 1

1 -4 -5 175.010 5.99343 6

1 -4 -5 169.048 5.94724 3

-1 4 5 182.357 5.92414 6

-1 4 5 177.228 5.98462 1

-1 4 5 181.647 5.96216 3

0 -4 -5 3.21446 0.64074 1

0 -4 -5 5.75061 0.87062 3

0 -4 -5 3.11582 0.76804 6

0 4 5 3.67280 0.58067 3

0 4 5 4.65255 0.48675 2

0 4 5 5.66713 0.83879 6

-1 -4 -5 485.899 15.7751 6

-1 -4 -5 509.302 15.7754 1

1 4 5 489.612 15.8121 1

1 4 5 493.031 15.7700 3

1 4 5 512.385 15.8291 6

1 4 5 491.535 15.5704 2

-2 -4 -5 -0.4884 0.69811 5

-2 -4 -5 1.55892 0.70599 1

2 4 5 0.11181 0.75955 6

2 4 5 1.24860 0.75766 1

-3 -4 -5 1011.68 33.1766 4

-3 -4 -5 1060.73 33.2536 1

-3 -4 -5 1088.61 33.3506 5

3 4 5 1039.98 33.3384 6

3 4 5 1088.31 33.1381 2

3 4 5 1065.16 33.2358 4

-4 -4 -5 15.8771 1.46426 5

-4 -4 -5 15.9327 1.61947 4

4 4 5 18.8353 1.51927 1

4 4 5 17.2977 1.84759 4

-5 -4 -5 5.42916 1.54429 4

-5 -4 -5 3.53839 1.08743 5

5 4 5 1.35830 0.88528 1

5 4 5 3.06851 1.77343 4

-6 -4 -5 2.16153 1.23029 5

-6 -4 -5 3.81192 1.71500 4

6 4 5 2.95517 0.96245 1

-7 -4 -5 68.8750 3.10871 5

7 4 5 66.4739 3.05991 1

-8 -4 -5 0.89256 1.33324 5

8 4 5 0.33732 1.13674 1

-9 -4 -5 1.69781 1.59004 5

9 4 5 -0.3354 1.30430 1

-10 -4 -5 5.83403 2.07210 5

10 4 5 1.32888 1.11181 3

10 4 5 3.91345 1.84157 1

-11 -4 -5 1.76665 2.36662 5

11 4 5 0.72226 2.19202 1

-12 -4 -5 1.36913 2.66660 5

12 4 5 3.24343 2.62902 1

12 4 5 0.13550 1.66195 3

-13 -4 -5 -1.2058 3.19847 5

13 4 5 5.64288 3.10044 1

13 4 5 0.23306 1.85279 3

13 -5 -5 2.56463 3.38371 6

13 -5 -5 -1.0586 3.09474 3

12 -5 -5 1.29466 2.50757 3

12 -5 -5 -0.2603 3.10171 6

-12 5 5 -2.0727 2.76311 1

11 -5 -5 10.0082 2.65061 6

11 -5 -5 10.6145 2.43569 3

11 -5 -5 6.11042 2.31071 1

-11 5 5 9.41492 2.64018 1

10 -5 -5 73.1208 4.50590 6

10 -5 -5 84.6264 4.82451 3

10 -5 -5 62.3273 4.50199 1

-10 5 5 79.8842 4.95719 1

9 -5 -5 4.88592 1.86099 6

9 -5 -5 -2.2142 1.60607 1

9 -5 -5 2.80678 1.49477 3

-9 5 5 1.97524 1.88209 1

8 -5 -5 24.0999 2.67130 6

8 -5 -5 26.8341 2.99994 3

8 -5 -5 22.4811 2.70036 1

-8 5 5 22.9715 3.09081 1

7 -5 -5 98.7835 4.58324 1

7 -5 -5 100.523 4.52639 6

7 -5 -5 111.497 4.69060 3

-7 5 5 113.825 4.77601 1

6 -5 -5 410.130 13.6122 3

6 -5 -5 397.646 13.5569 6

-6 5 5 419.527 13.5722 1

5 -5 -5 287.354 9.65529 3

5 -5 -5 267.377 9.79571 6

-5 5 5 295.676 9.73906 1

4 -5 -5 316.854 10.5539 6

4 -5 -5 323.877 10.6705 3

4 -5 -5 322.160 10.5442 4

-4 5 5 318.222 10.6453 1

3 -5 -5 98.9473 3.86747 3

3 -5 -5 100.454 3.77925 6

3 -5 -5 97.8939 3.56038 1

-3 5 5 94.7589 3.59767 3

-3 5 5 101.667 3.57905 1

-3 5 5 99.2222 3.78910 1

2 -5 -5 42.0030 2.03152 6

2 -5 -5 38.0982 2.08633 3

2 -5 -5 38.1886 1.77343 1

-2 5 5 35.3961 2.04277 1

-2 5 5 41.7803 1.74918 6

-2 5 5 38.7970 1.91939 3

1 -5 -5 1661.88 50.5169 2

1 -5 -5 1625.58 50.5164 1

1 -5 -5 1660.27 51.0970 6

1 -5 -5 1538.00 50.5702 3

-1 5 5 1616.00 50.5643 3

-1 5 5 1656.55 50.5092 6

-1 5 5 1567.41 50.5761 1

0 -5 -5 207.892 6.93875 3

0 -5 -5 204.450 6.91371 6

0 -5 -5 202.419 6.64612 2

0 -5 -5 209.567 6.90082 1

0 5 5 200.643 7.01159 1

0 5 5 217.916 6.95174 6

0 5 5 216.079 6.97266 3

0 5 5 213.689 6.65264 2

-1 -5 -5 1393.36 44.7326 6

-1 -5 -5 1449.34 44.7259 1

1 5 5 1442.71 44.8029 1

1 5 5 1390.38 44.7572 3

1 5 5 1446.61 44.5518 2

1 5 5 1463.36 44.7865 6

-2 -5 -5 219.840 7.47222 6

-2 -5 -5 248.677 8.23418 5

-2 -5 -5 221.661 7.48436 1

2 5 5 226.697 7.30708 2

2 5 5 219.566 7.53472 6

2 5 5 224.511 7.51623 3

-3 -5 -5 373.353 11.9174 1

-3 -5 -5 357.762 11.7212 5

3 5 5 352.906 11.8301 6

3 5 5 370.373 11.6228 2

-4 -5 -5 26.9607 1.76531 5

4 5 5 24.3791 1.85094 1

-5 -5 -5 391.402 12.3913 4

-5 -5 -5 380.983 12.3593 5

5 5 5 378.485 12.3267 1

5 5 5 367.238 12.5653 4

-6 -5 -5 29.3563 2.28149 5

6 5 5 35.9670 2.18504 1

-7 -5 -5 11.2051 1.56718 5

7 5 5 8.75432 1.25857 1

-8 -5 -5 9.67389 1.76866 5

8 5 5 8.45742 1.40972 1

-9 -5 -5 4.34229 1.78772 5

9 5 5 5.80103 1.55626 1

-10 -5 -5 0.15005 2.01261 5

10 5 5 0.39188 1.32489 3

10 5 5 1.75403 1.72744 1

-11 -5 -5 0.01099 4.71818 5

11 5 5 0.31773 1.39532 3

-12 -5 -5 2.35312 2.93405 5

12 5 5 0.62264 1.70079 3

12 5 5 -1.6251 2.56101 1

13 -6 -5 -4.8508 3.48418 3

13 -6 -5 -5.3115 3.52926 6

-13 6 5 5.20189 5.69172 1

12 -6 -5 0.81351 3.32790 6

12 -6 -5 -0.0264 2.94071 3

-12 6 5 -1.2356 2.98779 1

11 -6 -5 4.11738 2.53009 6

11 -6 -5 4.15978 2.60862 3

-11 6 5 1.88135 2.69774 1

10 -6 -5 -0.3366 2.17461 6

10 -6 -5 0.91498 2.10002 3

-10 6 5 -2.0785 2.19874 1

9 -6 -5 4.26596 1.65558 3

9 -6 -5 4.01189 2.11718 6

-9 6 5 2.42403 2.06463 1

8 -6 -5 11.9089 2.07860 6

8 -6 -5 12.4673 1.86690 3

-8 6 5 7.07870 1.91787 1

7 -6 -5 2.56558 1.60427 6

7 -6 -5 1.16674 1.31278 3

-7 6 5 1.31043 1.56123 1

6 -6 -5 73.5701 3.54904 6

6 -6 -5 74.5757 3.81453 3

-6 6 5 73.7351 3.83344 1

5 -6 -5 328.476 11.2951 6

5 -6 -5 340.174 11.4659 3

-5 6 5 351.758 11.4655 1

4 -6 -5 178.311 6.40417 3

4 -6 -5 169.765 6.22438 6

4 -6 -5 169.654 6.12947 4

-4 6 5 183.701 6.33946 1

3 -6 -5 119.883 4.29038 4

3 -6 -5 120.682 4.44772 6

3 -6 -5 115.639 4.54845 3

-3 6 5 118.087 4.19675 1

-3 6 5 115.831 4.75652 1

2 -6 -5 850.283 27.4510 6

2 -6 -5 885.116 27.4580 1

2 -6 -5 825.793 27.1397 2

-2 6 5 850.609 27.4612 3

-2 6 5 893.732 27.3417 6

-2 6 5 917.139 27.4514 1

1 -6 -5 1845.15 56.1954 6

1 -6 -5 1783.15 55.6405 2

1 -6 -5 1676.17 55.5840 1

1 -6 -5 1900.51 57.6828 3

-1 6 5 1719.41 55.8730 1

-1 6 5 1739.67 55.6749 3

-1 6 5 1808.57 55.5894 6

0 -6 -5 155.787 5.21778 2

0 -6 -5 176.582 5.98817 3

0 -6 -5 159.003 5.45816 1

0 -6 -5 149.285 5.45279 6

0 6 5 159.283 5.51682 6

0 6 5 159.908 5.58898 3

0 6 5 159.580 5.21761 2

0 6 5 169.759 5.87466 1

-1 -6 -5 41.2083 2.03697 1

-1 -6 -5 40.0791 1.71267 2

-1 -6 -5 44.0142 1.96002 6

-1 -6 -5 43.8732 2.23001 5

1 6 5 47.7291 2.20654 6

1 6 5 41.4863 2.43390 1

1 6 5 43.6308 2.09891 3

1 6 5 41.5379 1.72375 2

-2 -6 -5 -0.1840 0.74223 6

-2 -6 -5 -0.2314 0.76566 5

2 6 5 0.06153 0.58436 2

2 6 5 0.52727 0.98410 6

-3 -6 -5 529.466 17.0737 5

3 6 5 526.595 16.9406 2

3 6 5 549.082 17.1480 1

-4 -6 -5 24.9285 1.98228 5

4 6 5 24.9207 2.08966 1

-5 -6 -5 44.8696 2.51702 5

5 6 5 45.2966 2.47476 1

-6 -6 -5 65.0497 3.17107 5

6 6 5 71.4202 3.07631 1

6 6 5 63.0086 3.60807 4

-7 -6 -5 14.5030 1.75322 5

7 6 5 8.72095 1.35496 1

-8 -6 -5 32.3992 2.90198 5

8 6 5 32.3440 2.62924 1

-9 -6 -5 2.96414 1.78230 5

9 6 5 1.10140 1.43795 1

-10 -6 -5 0.63511 2.20404 5

10 6 5 -1.4982 1.68227 1

-11 -6 -5 -2.5852 2.41665 5

11 6 5 -1.4651 1.48336 3

11 6 5 -1.9358 2.85801 1

-12 -6 -5 3.08325 3.25849 5

12 6 5 1.96559 1.69639 3

12 6 5 3.05952 2.75452 1

13 -7 -5 -2.9267 3.40605 3

13 -7 -5 -1.7577 3.67401 6

-13 7 5 7.68203 8.41240 1

12 -7 -5 0.52012 3.29181 6

12 -7 -5 -2.3877 2.87948 3

-12 7 5 -1.4648 3.25027 1

11 -7 -5 1.92770 2.99731 6

-11 7 5 0.90450 2.90343 1

10 -7 -5 1.08654 2.09816 3

10 -7 -5 4.62145 2.69675 6

-10 7 5 4.39885 2.65982 1

9 -7 -5 20.1737 2.73336 6

9 -7 -5 19.6279 2.31979 3

-9 7 5 17.2417 2.74382 1

8 -7 -5 0.64629 1.67370 3

8 -7 -5 -1.0585 1.98323 6

-8 7 5 -1.7466 1.94338 1

7 -7 -5 55.0712 3.40949 6

7 -7 -5 57.1345 3.69332 3

-7 7 5 58.6378 3.86148 1

6 -7 -5 31.7054 2.64645 6

6 -7 -5 36.8927 3.06015 3

-6 7 5 35.5070 3.20167 1

5 -7 -5 175.932 6.62502 3

5 -7 -5 175.873 6.40034 6

-5 7 5 172.076 6.59001 1

4 -7 -5 756.526 24.6626 3

4 -7 -5 755.361 24.4725 4

-4 7 5 798.956 24.6692 1

3 -7 -5 803.974 24.7834 4

3 -7 -5 782.937 24.9705 3

3 -7 -5 760.636 24.5102 2

3 -7 -5 803.310 24.8580 6

-3 7 5 762.665 24.9316 1

2 -7 -5 719.608 22.3961 4

2 -7 -5 696.079 22.6530 3

2 -7 -5 684.093 22.2288 2

2 -7 -5 706.303 22.5437 6

-2 7 5 728.052 22.4014 6

-2 7 5 727.054 22.6606 1

1 -7 -5 589.223 19.0096 2

1 -7 -5 599.507 19.3832 3

1 -7 -5 601.583 19.2602 6

-1 7 5 611.244 19.4021 1

-1 7 5 629.884 19.2191 6

0 -7 -5 106.448 3.69019 2

0 -7 -5 106.179 3.92652 6

0 7 5 111.349 4.02926 6

0 7 5 109.661 3.68146 2

0 7 5 109.340 4.20631 1

-1 -7 -5 102.891 3.74063 6

-1 -7 -5 102.166 3.82480 5

-1 -7 -5 100.394 3.57426 2

1 7 5 103.163 4.04074 1

1 7 5 107.593 3.95159 6

1 7 5 100.864 3.55035 2

-2 -7 -5 157.016 6.06493 6

-2 -7 -5 162.708 5.60292 5

2 7 5 162.939 5.69956 6

2 7 5 160.885 5.37971 2

2 7 5 160.191 5.75406 1

-3 -7 -5 142.856 5.11713 5

3 7 5 143.051 5.24600 1

-4 -7 -5 463.642 14.9976 5

4 7 5 464.383 15.0202 1

-5 -7 -5 15.6275 1.99416 5

5 7 5 11.3846 1.40395 1

-6 -7 -5 24.0887 2.25865 5

6 7 5 26.6358 2.12515 1

-7 -7 -5 18.3458 2.57484 5

7 7 5 17.5430 2.32219 1

-8 -7 -5 21.0437 2.35181 5

8 7 5 20.6458 2.51956 1

-9 -7 -5 2.16320 1.95620 5

9 7 5 1.53151 1.62587 1

-10 -7 -5 4.80780 2.39862 5

10 7 5 0.96999 1.91179 1

-11 -7 -5 2.98098 2.90066 5

11 7 5 0.81445 2.42253 1

11 7 5 -0.0544 1.71825 3

-12 -7 -5 0.41597 3.34005 5

12 7 5 -1.7294 2.75319 1

12 7 5 -1.2533 1.80861 3

12 -8 -5 3.28184 3.53529 6

12 -8 -5 0.13621 3.06758 3

-12 8 5 4.49507 3.98499 1

11 -8 -5 1.16945 2.79147 3

11 -8 -5 2.18488 3.03728 6

-11 8 5 6.94553 3.25474 1

10 -8 -5 2.53794 2.75286 6

10 -8 -5 -1.0791 2.39654 3

-10 8 5 -1.0637 2.77229 1

9 -8 -5 1.53174 2.28242 6

9 -8 -5 2.66553 2.01699 3

-9 8 5 0.77629 2.51893 1

8 -8 -5 7.00325 1.85382 3

8 -8 -5 10.9986 2.28415 6

-8 8 5 5.47736 2.32429 1

7 -8 -5 0.82064 1.89565 6

7 -8 -5 1.32641 1.66920 3

-7 8 5 -0.2815 1.95347 1

6 -8 -5 9.77187 1.78924 3

6 -8 -5 11.3284 1.97008 6

-6 8 5 8.02671 2.00202 1

5 -8 -5 1.07353 1.59763 6

5 -8 -5 -0.7035 1.23327 3

-5 8 5 -1.6238 1.45183 1

4 -8 -5 381.804 12.6656 6

4 -8 -5 375.459 12.5930 3

-4 8 5 367.617 12.5791 1

3 -8 -5 138.054 5.17904 4

3 -8 -5 143.667 5.51154 3

3 -8 -5 145.025 4.85445 2

3 -8 -5 146.225 5.35030 6

-3 8 5 145.818 5.52230 1

2 -8 -5 0.31834 0.65172 2

2 -8 -5 -0.7425 0.99504 3

2 -8 -5 -0.5482 1.21710 6

-2 8 5 1.63633 0.84162 6

-2 8 5 -0.6587 1.22159 1

1 -8 -5 29.4700 2.09865 3

1 -8 -5 31.0034 1.53218 2

1 -8 -5 28.0669 1.94287 6

-1 8 5 27.5705 2.32080 1

-1 8 5 33.4796 1.85870 6

0 -8 -5 189.404 6.33061 2

0 -8 -5 192.190 6.51127 6

0 8 5 195.057 6.34599 2

0 8 5 198.708 6.60634 6

0 8 5 190.480 6.81742 1

-1 -8 -5 71.4485 2.84157 2

-1 -8 -5 76.5215 3.00174 6

-1 -8 -5 75.2936 3.14864 5

1 8 5 74.0947 3.23481 6

1 8 5 74.9337 2.84507 2

1 8 5 78.0493 3.46507 1

-2 -8 -5 603.512 19.4952 5

-2 -8 -5 598.585 19.6573 6

2 8 5 634.051 19.4463 2

2 8 5 599.519 19.5420 6

2 8 5 620.578 19.6513 1

-3 -8 -5 52.1911 2.62562 5

3 8 5 51.1491 2.98085 1

-4 -8 -5 7.61607 1.29603 5

4 8 5 7.50018 1.39717 1

-5 -8 -5 82.6624 3.68966 5

5 8 5 84.6583 3.64433 1

-6 -8 -5 162.804 6.07550 5

6 8 5 162.712 5.96221 1

-7 -8 -5 5.16240 1.78536 5

7 8 5 8.43470 1.52501 1

-8 -8 -5 1.81605 1.67880 5

8 8 5 0.21653 1.38820 1

-9 -8 -5 4.23141 2.14161 5

9 8 5 2.81171 1.75344 1

-10 -8 -5 -1.5065 2.34723 5

10 8 5 -0.4541 2.03555 1

-11 -8 -5 7.55739 3.06701 5

11 8 5 4.08916 1.82592 3

11 8 5 4.27302 2.54822 1

-12 -8 -5 3.55649 3.83396 5

12 8 5 0.90040 2.03010 3

12 8 5 1.59035 2.80954 1

12 -9 -5 -4.1473 3.62492 6

12 -9 -5 -3.6495 3.11759 3

-12 9 5 3.14067 3.93184 1

11 -9 -5 -1.7925 2.75203 3

11 -9 -5 -6.7947 3.11327 6

-11 9 5 1.03467 3.43287 1

10 -9 -5 4.73160 2.65827 3

10 -9 -5 -0.4896 2.74324 6

-10 9 5 -1.7357 3.06968 1

9 -9 -5 -3.6462 2.37259 6

9 -9 -5 -0.0257 2.12812 3

-9 9 5 -3.2168 2.67341 1

8 -9 -5 10.9849 2.46265 6

8 -9 -5 6.91233 2.15929 3

-8 9 5 6.79798 2.52750 1

7 -9 -5 5.24106 1.97322 3

7 -9 -5 8.11319 2.17157 6

-7 9 5 4.14834 2.30014 1

6 -9 -5 21.0961 3.10692 3

6 -9 -5 20.6249 2.50535 6

-6 9 5 15.5285 2.40767 1

5 -9 -5 2.60779 1.46125 3

5 -9 -5 2.52642 1.68644 6

-5 9 5 -1.4480 1.66097 1

4 -9 -5 84.8864 4.30351 6

4 -9 -5 86.9610 4.21442 3

4 -9 -5 90.1156 3.39171 2

-4 9 5 93.0599 4.31186 1

3 -9 -5 7.52399 1.39732 3

3 -9 -5 9.76593 1.34506 2

3 -9 -5 11.7086 1.67255 6

-3 9 5 8.39574 1.65041 1

2 -9 -5 28.9125 2.18701 6

2 -9 -5 30.0481 1.69283 2

2 -9 -5 28.8410 2.44004 3

-2 9 5 23.9996 2.74009 1

-2 9 5 30.2726 1.76497 6

1 -9 -5 47.7516 2.12722 2

1 -9 -5 46.3428 2.35424 6

1 -9 -5 50.9778 2.70898 3

-1 9 5 48.1594 2.47166 1

-1 9 5 48.2295 2.40323 6

-1 9 5 45.2539 2.94039 1

0 -9 -5 44.8081 2.09480 2

0 -9 -5 49.7253 2.45190 5

0 -9 -5 45.0680 2.27005 6

0 9 5 47.0382 2.87049 1

0 9 5 46.7961 2.55854 6

-1 -9 -5 7.89282 1.15761 6

-1 -9 -5 5.48296 1.26844 5

1 9 5 6.38513 0.93775 2

1 9 5 5.33157 1.54844 6

1 9 5 4.98373 1.49327 1

-2 -9 -5 2.39017 1.08743 5

-2 -9 -5 1.63007 0.88823 2

2 9 5 3.47666 1.41837 1

-3 -9 -5 1.17719 1.17000 5

3 9 5 -0.7780 1.30809 1

-4 -9 -5 15.1482 1.70819 5

4 9 5 15.8364 2.39975 1

-5 -9 -5 12.1193 1.74608 5

5 9 5 10.9179 1.60942 1

-6 -9 -5 29.9737 2.70386 5

6 9 5 27.1502 2.62292 1

-7 -9 -5 17.3597 2.23769 5

7 9 5 14.0754 1.73610 1
[truncated: 875,700 more chars]
