# Supplementary material for: A microporous polymer based on nonconjugated hindered biphenyls that emits blue light
Source: Sci Rep. 2024 Jun 28;14:14923. doi: 10.1038/s41598-024-65743-5 (PMC11213909; doi:10.1038/s41598-024-65743-5)

checkCIF/PLATON report

You have not supplied any structure factors. As a result the full set of tests cannot be run.

THIS REPORT IS FOR GUIDANCE ONLY. IF USED AS PART OF A REVIEW PROCEDURE FOR PUBLICATION, IT SHOULD NOT REPLACE THE EXPERTISE OF AN EXPERIENCED CRYSTALLOGRAPHIC REFEREE.

No syntax errors found.   CIF dictionary   Interpreting this report

Datablock: 3,3',5,5'-tetraiodo-2,2'-6,6'-tetramethylbipheny

```
Bond precision:  C-C = 0.0088 A          Wavelength=0.71073

Cell:            a=9.0436(5)     b=12.1873(6)       c=17.4292(9)
                 alpha=90        beta=98.137(2)     gamma=90
Temperature:     293 K

                Calculated                 Reported
Volume          1901.65(17)                1901.65(17)
Space group     P 21/c                     P 21/c
Hall group      -P 2ybc                    -P 2ybc
Moiety formula  C16 H14 I4                 ?
Sum formula     C16 H14 I4                 C16 H14 I4
Mr              713.87                     713.87
Dx,g cm-3       2.493                      2.493
Z               4                          4
Mu (mm-1)       6.544                      6.544
F000            1288.0                     1288.0
F000'           1280.91
h,k,lmax        11,15,21                   11,15,21
Nref            3883                       3880
Tmin,Tmax       0.028,0.038                0.508,0.747
Tmin'           0.001

Correction method= # Reported T Limits: Tmin=0.508 Tmax=0.747
AbsCorr = MULTI-SCAN

Data completeness= 0.999          Theta(max)= 26.371

R(reflections)= 0.0403( 3401)     wR2(reflections)= 0.1299( 3880)

S = 1.050                  Npar= 185
```

The following ALERTS were generated. Each ALERT has the format
test-name_ALERT_alert-type_alert-level.
Click on the hyperlinks for more details of the test.

Alert level A

```
PLAT138_ALERT_1_A Monoclinic: More than one Angle Unequal 90.0 ...     Please Check
```

Author Response: The angles alpha and gamma taken from the diffractometer deviate sligh

---

Alert level B

```
SYMMS02_ALERT_1_B  Two angles should be 90 for a monoclinic cell
  Cell        9.0436   12.1873   17.4292
  Angles    90.0030   98.1370   89.9130
SYMMS02_ALERT_1_B  Two angles should be 90 for a monoclinic cell
  Cell        9.0436   12.1873   17.4292
  Angles    90.0030   98.1370   89.9130
SYMMS02_ALERT_1_B  Two angles should be 90 for a monoclinic cell
  Cell        9.0436   12.1873   17.4292
  Angles    90.0030   98.1370   89.9130
```

Alert level C

```
PLAT048_ALERT_1_C MoietyFormula Not Given (or Incomplete) ........     Please Check
PLAT342_ALERT_3_C Low Bond Precision on  C-C Bonds ...............    0.00882 Ang.
```

Alert level G

```
PLAT063_ALERT_4_G Crystal Size Possibly too Large for Beam Size ..       1.00 mm
PLAT083_ALERT_2_G SHELXL Second Parameter in WGHT  Unusually Large       6.96 Why ?
PLAT199_ALERT_1_G Reported _cell_measurement_temperature ..... (K)        293 Check
PLAT200_ALERT_1_G Reported   _diffrn_ambient_temperature ..... (K)        293 Check
PLAT802_ALERT_4_G CIF Input Record(s) with more than 80 Characters          2 Info
PLAT883_ALERT_1_G No Info/Value for _atom_sites_solution_primary .     Please Do !
PLAT965_ALERT_2_G The SHELXL WEIGHT Optimisation has not Converged     Please Check
```

---

1 **ALERT level A** = Most likely a serious problem - resolve or explain
3 **ALERT level B** = A potentially serious problem, consider carefully
2 **ALERT level C** = Check. Ensure it is not caused by an omission or oversight
7 **ALERT level G** = General information/check it is not something unexpected

8 ALERT type 1 CIF construction/syntax error, inconsistent or missing data
2 ALERT type 2 Indicator that the structure model may be wrong or deficient
1 ALERT type 3 Indicator that the structure quality may be low
2 ALERT type 4 Improvement, methodology, query or suggestion
0 ALERT type 5 Informative message, check

---

checkCIF publication errors

Alert level G

```
PUBL017_ALERT_1_G The _publ_section_references section is missing or
            empty.
```

```
   0 ALERT level A = Data missing that is essential or data in wrong format
   1 ALERT level G = General alerts. Data that may be required is missing
```

Publication of your CIF

You should attempt to resolve as many as possible of the alerts in all categories. Often the minor alerts point to easily fixed oversights, errors and omissions in your CIF or refinement strategy, so attention to these fine details can be worthwhile. In order to resolve some of the more serious problems it may be necessary to carry out additional measurements or structure refinements. However, the nature of your study may justify the reported deviations from journal submission requirements and the more serious of these should be commented upon in the discussion or experimental section of a paper or in the "special_details" fields of the CIF. *checkCIF* was carefully designed to identify outliers and unusual parameters, but every test has its limitations and alerts that are not important in a particular case may appear. Conversely, the absence of alerts does not guarantee there are no aspects of the results needing attention. It is up to the individual to critically assess their own results and, if necessary, seek expert advice.

If you wish to submit your CIF for publication in Acta Crystallographica Section C or E, you should upload your CIF via the web. If you wish to submit your CIF for publication in IUCrData, you should upload your CIF via the web. If your CIF is to form part of a submission to another IUCr journal, you will be asked, either during electronic submission or by the Co-editor handling your paper, to upload your CIF via our web site.

PLATON version of 22/03/2021; check.def file version of 19/03/2021

Datablock 3,3',5,5'-tetraiodo-2,2'-6,6'-tetramethylbiphenyl - ellipsoid plot

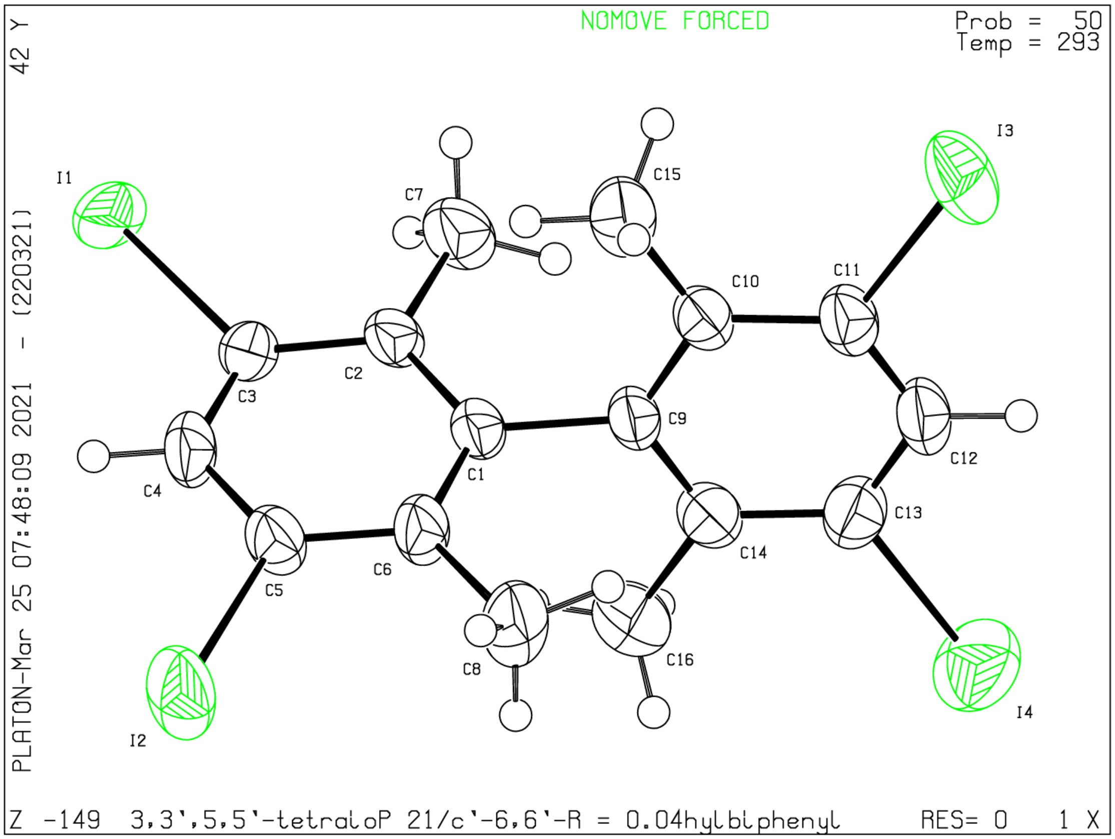

Supplement: Supplementary file 1 — Supplementary Information 1. [file 41598_2024_65743_MOESM1_ESM.pdf]
